# New Class of Hsp90 C-Terminal Domain Inhibitors with Anti-tumor Properties against Triple-Negative Breast Cancer

**DOI:** 10.1021/acs.jmedchem.4c00932

**Published:** 2024-07-23

**Authors:** Živa Zajec, Jaka Dernovšek, Jernej Cingl, Iza Ogris, Marius Gedgaudas, Asta Zubrienė, Ana Mitrović, Simona Golič Grdadolnik, Martina Gobec, Tihomir Tomašič

**Affiliations:** † Faculty of Pharmacy, University of Ljubljana, Aškerčeva cesta 7, 1000 Ljubljana, Slovenia; ‡ Laboratory for Molecular Structural Dynamics, Theory Department, National Institute of Chemistry, Hajdrihova 19, 1001 Ljubljana, Slovenia; § Department of Biothermodynamics and Drug Design, Institute of Biotechnology, Life Sciences Center, Vilnius University, Saulėtekio al. 7, LT-10257 Vilnius, Lithuania; ∥ Department of Biotechnology, Jožef Stefan Institute, Jamova 39, 1000 Ljubljana, Slovenia

## Abstract

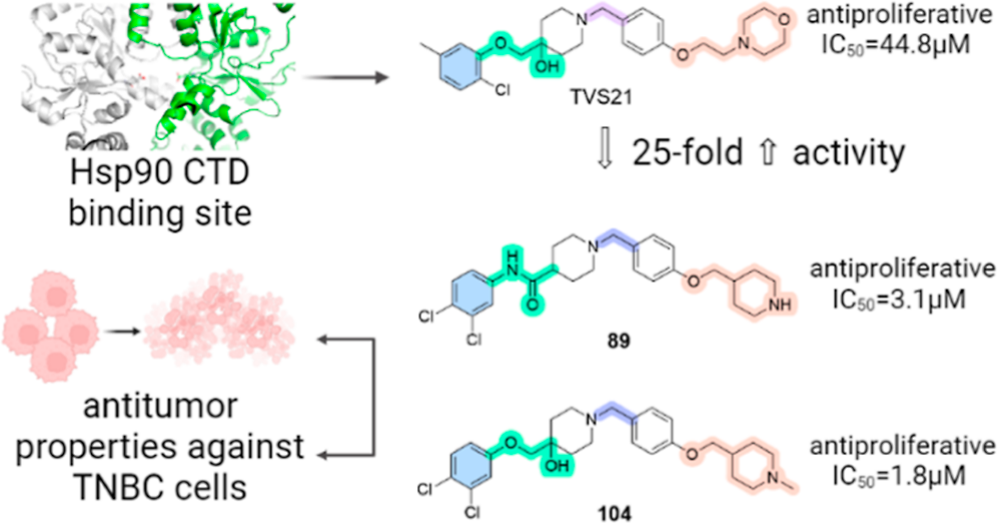

Triple-negative breast cancer (TNBC) remains a treatment challenge and requires innovative therapies. Hsp90, crucial for the stability of numerous oncogenic proteins, has emerged as a promising therapeutic target. In this study, we present the optimization of the Hsp90 C-terminal domain (CTD) inhibitor **TVS21**. Biochemical methods, NMR binding studies, and molecular modeling were employed to investigate the binding of representative analogs to Hsp90. The newly synthesized analogs showed increased antiproliferative activity in breast cancer cell lines, including the MDA-MB-231 TNBC cell line. Compounds **89** and **104** proved to be the most effective, inducing apoptosis, slowing proliferation, and degrading key oncogenic proteins without inducing a heat shock response. In vivo, compound **89** showed comparable efficacy to the clinical candidate AUY922 and a better safety profile in a TNBC xenograft model. These results highlight the promise of Hsp90 CTD inhibitors for TNBC therapy, potentially filling a significant treatment gap.

## Introduction

Breast cancer is one of the three most common malignancies and the most common cancer in women. The 5 year survival rate of breast cancer patients depends on several factors, such as the type of cancer and stage of the cancer at diagnosis and can vary from 90 to 25%.^1^ At the molecular level, breast cancer is a highly heterogeneous disease and can be classified into three main subtypes based on the presence or absence of specific molecular markers. Treatment includes surgery, radiation therapy, and systemic therapy, which is highly dependent on the subtype of breast cancer.^2,3^ The most common subtype is hormone-positive (positive for estrogen or progesterone receptor) breast cancer, which is usually treated with endocrine therapies such as aromatase inhibitors or estrogen receptor antagonist tamoxifen. The second most common is human epidermal growth factor receptor 2 (HER2)-positive breast cancer. With the development of trastuzumab, a monoclonal antibody against HER2, major advances have been made in systemic therapy for this breast cancer subtype.^1,4−6^ The remaining 10–15% of breast cancers are defined by the absence of these three markers and are therefore referred to as triple-negative breast cancer (TNBC), which has the most unfavorable prognosis and an increased risk of recurrence. Unlike hormone-positive and HER2-positive breast cancers, there are no targeted therapies for TNBC, so the standard treatment is cytotoxic chemotherapy. Numerous clinical trials of TNBC therapy have been conducted, but so far none have been successful,^7−9^ therefore new therapies are urgently needed.^10^

Heat shock protein 90 (Hsp90) is a chaperone that belongs to the heat shock protein family, a group of proteins that are induced in response to stress or cellular damage.^11^ It stabilizes proteins that are in an incompletely folded or unstable state, helps to guide them through the folding process, and is involved in controlling protein quality in the cell. Hsp90 maintains the stability and function of a number of proteins involved in various cellular processes, including cell cycle regulation, signaling pathways, and protein degradation.^12^ Hsp90 also plays a role in the activation and regulation of signaling proteins, such as receptor tyrosine kinases, by altering their conformations.^13^ The functional form of Hsp90 consists of two identical monomers, each with three characteristic domains: the N-terminal ATP-binding domain (NTD), a middle domain, and the C-terminal domain, which is important for dimerization of Hsp90.^14^ Dysregulation of Hsp90 function has been associated with a number of diseases, including cancer, neurodegenerative disorders, infectious diseases, and cardiovascular diseases.^15^ As a result, Hsp90 has been the subject of intense research and is being investigated as a potential therapeutic target. In cancer, Hsp90 has been shown to play a key role in the stabilization and activation of various oncogenic proteins such as kinases (e.g., AKT, MEK, STAT3, and mTOR) and transcription factors (e.g., estrogen receptor, androgen receptor) involved in all hallmarks of cancer.^11,16−19^ Since many tumor-promoting factors depend on Hsp90, proliferation of cancer cells is highly dependent on Hsp90 function, leading to their addiction to Hsp90. Moreover, malignant transformation is associated with proteotoxic and nutritional stress, which increases Hsp90 levels.^20^ Inhibition of Hsp90 simultaneously affects multiple oncogenic proteins and cancer pathways, making Hsp90 an attractive target for anticancer drug development, including TNBC.^21^

Most of the Hsp90 inhibitors developed and studied in clinical trials bind to the Hsp90 NTD, but unfortunately, most of them have not achieved clinical relevance due to off-target effects and lack of efficacy. Another shortcoming of Hsp90 NTD inhibitors is the induction of heat shock response (HSR), which causes upregulation of Hsp27,^22^ Hsp70, and Hsp90, leading to suppression of apoptosis and promoting cancer cell survival.^23−26^ The drawbacks associated with Hsp90 NTD inhibition can be circumvented by the use of novel strategies for Hsp90 inhibition, such as isoform-selective NTD inhibitors,^27−30^ allosteric CTD inhibitors,^31,32^ or targeting protein–protein interactions between Hsp90 and its cochaperones or substrates.^33^ To date, pimitespib is the only Hsp90α/β-selective NTD inhibitor approved for the treatment of cancer, particularly for the treatment of gastrointestinal stromal tumors.^34^

**Figure 1:**
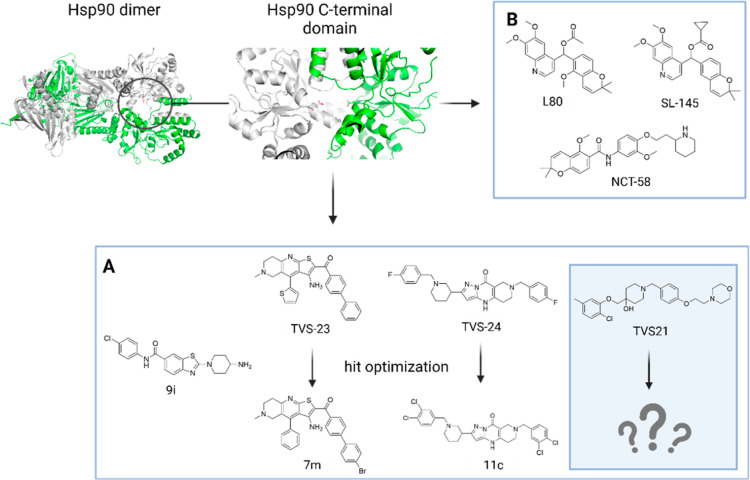
Hsp90 CTD (PDB entry: 5FWK) and its inhibitors. (A) Structures of our previously discovered and optimized structurally diverse Hsp90 CTD inhibitors; (B) Deguelin-based Hsp90 CTD inhibitors with known activity in TNBC cell lines.

Allosteric Hsp90 CTD inhibition is an attractive new strategy for targeting Hsp90 because it causes degradation of oncogenic proteins but, unlike Hsp90 NTD inhibition, does not induce HSR.^35,36^ There is no cocrystal structure of Hsp90 CTD with a noncovalent allosteric inhibitor thus far, making structure-based design difficult and leading to a lack of structural diversity. There have been several reports of Hsp90 CTD inhibitors with anticancer activity against HER2-positive breast cancer as well as TNBC,^37−40^ but most of them are analogs of known natural products such as deguelin (Figure 1).

Recently, we reported the discovery of a new structural class of Hsp90 CTD inhibitors using 3D ligand- and structure-based pharmacophore models derived from molecular dynamics (MD) simulations. In this study, we identified compound **TVS21** (Figure 1) and showed that it exerts anticancer activity against breast and liver cancer cell lines, MCF-7 and HepG2, respectively, as well as dose-dependent degradation of oncogenic proteins in MCF-7 cell line.^41^ In addition, we have used structure-based virtual screening and de novo design to identify and successfully optimize new structural classes of Hsp90 CTD inhibitors with anticancer activity.^42−46^

Building on our findings from prior work,^41^ we herein report the expansion of the structure–activity relationship (SAR) of analogs of our previously discovered Hsp90 CTD inhibitor **TVS21** by employing structure-based pharmacophore modeling and subsequent organic synthesis of prioritized compounds. Through this study, we identified a new structural class of Hsp90 CTD inhibitors with improved anticancer activity against various types of breast cancer cell lines. In our efforts, we have identified compound **89** that induces apoptosis of TNBC cells and inhibits cell proliferation in vitro, as well as slows TNBC growth in vivo.

## Results and Discussion

### Design of Novel Hsp90 CTD Inhibitors

**Figure 2:**
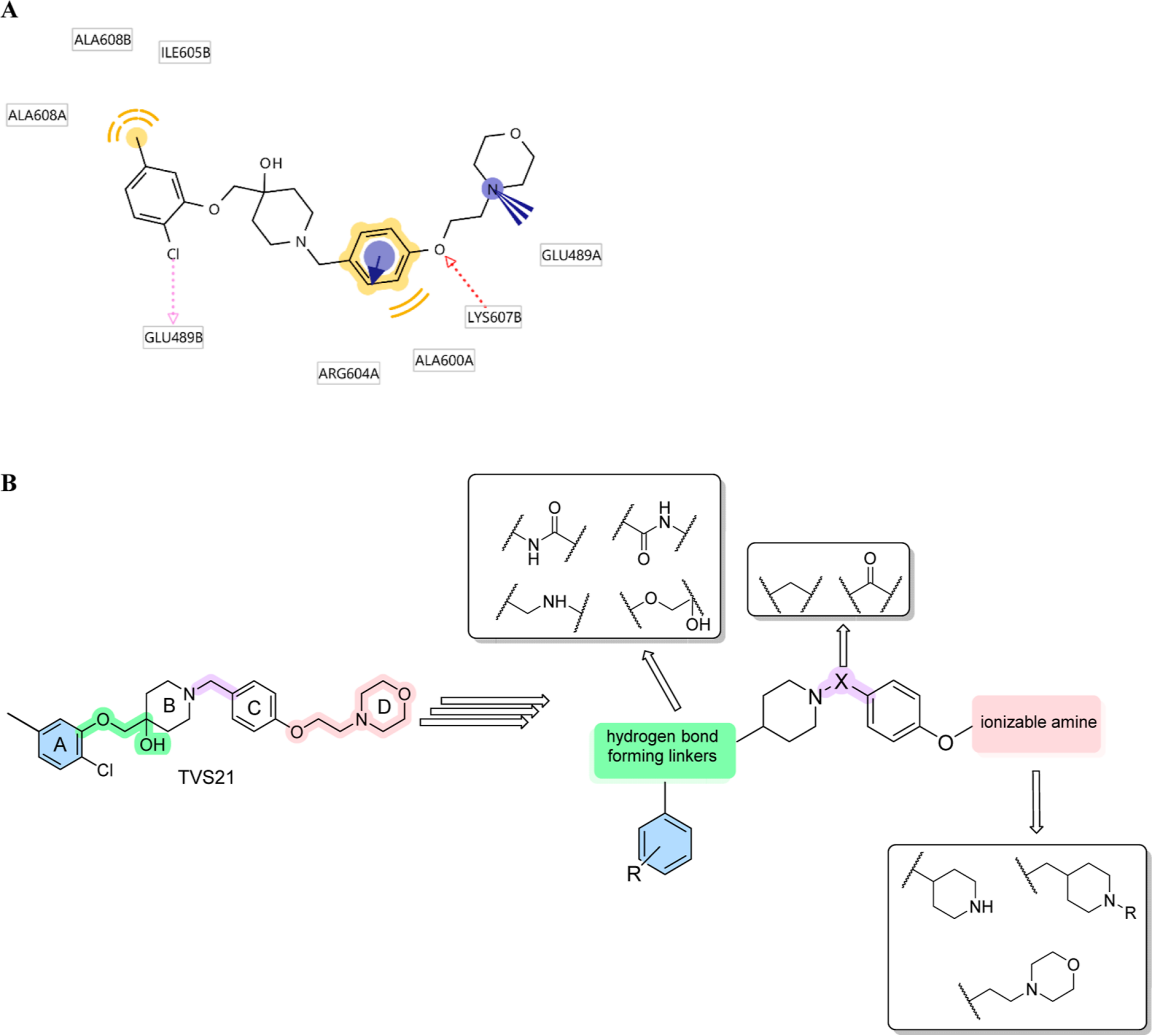
(A) MD-derived binding mode of **TVS21** in Hsp90 CTD. **TVS21** forms hydrophobic interactions with Ala600A, Ala608A, Ile605B, and Ala608B (in yellow), a cation–π stacking with Arg604A (blue circle with an arrow), an ionic interaction with Glu489A (blue lines), a hydrogen bond with Lys607B (red arrow), and a halogen bond with Glu489B backbone carbonyl (pink arrow); (B) optimization strategy and SAR investigation of **TVS21** analogs as Hsp90 CTD inhibitors.

Our starting point for SAR investigation was compound **TVS21**. The binding mode of **TVS21** in allosteric Hsp90 CTD binding site was studied by a combination of molecular docking and pharmacophore modeling. According to the calculated binding mode (Figure 2A), 2-chloro-5-methylphenyl moiety (ring A) of **TVS21** forms a network of hydrophobic contacts with Ile605B, Ala608A, and Ala608B. In addition, a halogen bond between the chlorine atom and Glu489B backbone carbonyl is predicted. Furthermore, the phenyl ring C forms a cation–π interaction with the Arg604A side chain guanidinium group and a hydrophobic interaction with Ala600A. One of the critical structural elements for binding is the morpholine basic nitrogen, which forms an ionic interaction with Glu489A side chain. In addition, the oxygen atom in the ethyloxy bridge between rings C and D forms a hydrogen bond with Lys607B.

In the present study, we systematically explored the impact of modification of rings A–D of **TVS21** (Figure 2B) on the antiproliferative activity in breast cancer cell lines. We varied different substituents on the phenyl ring A to further explore hydrophobic interactions and potential halogen bonds, introduced different hydrogen bond forming linkers between rings A and B, and studied the effect of the distance between ionizable amine in ring D and the phenyl ring A. In addition, we studied the effect of the carbonyl or methylene group as a bridge between rings B and C on antiproliferative activity.

### Synthesis of the Designed Hsp90 CTD Inhibitors

**Scheme 1:**
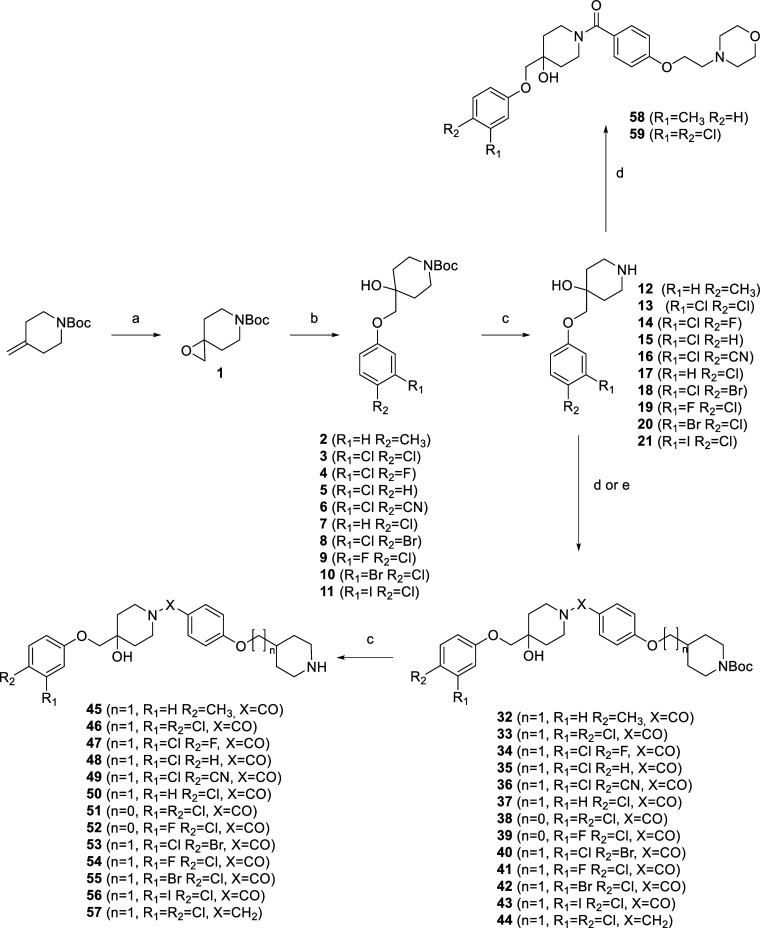
Synthesis of the Final Compounds **45**–**59** Reagents and conditions: (a) *m*CPBA, chloroform, 0 °C, 18 h; (b) for **2**: *p*-cresol, for **3**: 3,4-dichlorophenol, for **4**: 3-chloro-4-fluorophenol, for **5**: 3-chlorophenol, for **6**: 3-chloro-4-hydroxybenzonitrile, for **7**: 4-chlorophenol, for **8**: 3-chloro-4-bromophenol, for **9**: 3-fluoro-4-chlorophenol, for **10**: 3-bromo-4-chlorophenol, for **11**: 3-iodo-4-chlorophenol, K_2_CO_3_, DMF, 80 °C, 18 h; (c) CF_3_COOH, 20 °C, DCM, 18 h; (d) for **32**–**37** and **40**–**43**: **26**, for **38**, **39**: **29**, for **58**, **59**: **31**, HOBt, EDC, *N*-methylmorpholine (NMM), DMF, 18 h, (e) **24**, NaCNBH_3_, MeOH, 18 h.

**Scheme 2:**
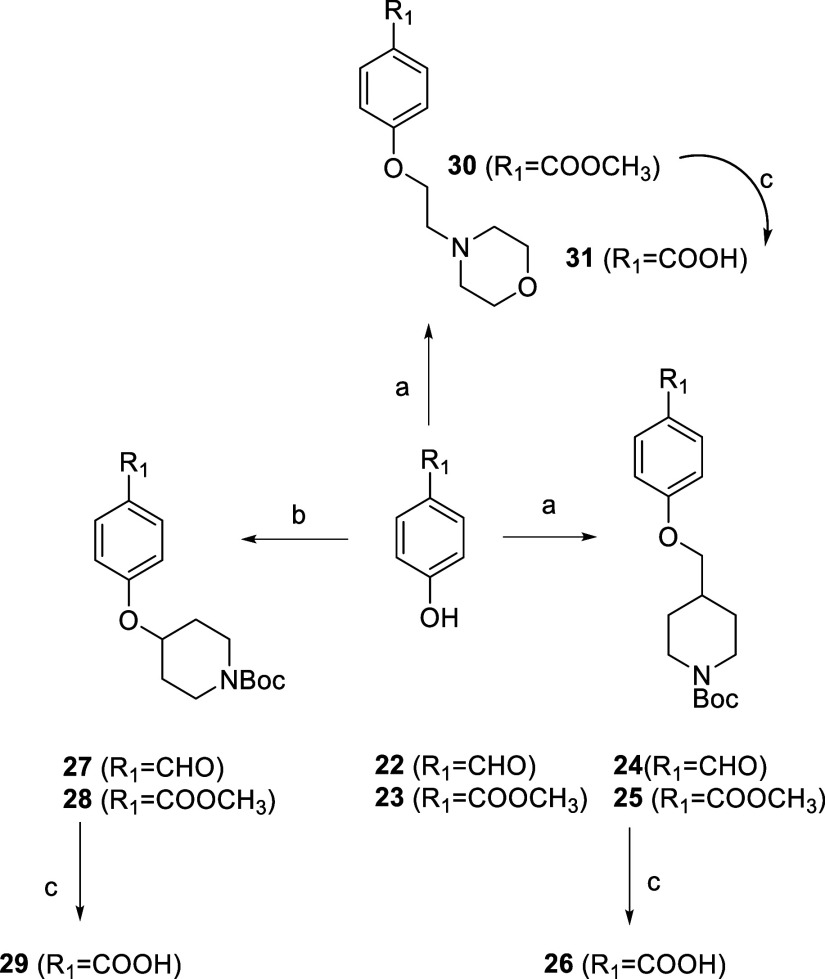
Synthesis of the Building Blocks **26**, **29,** and **31** Reagents and conditions: (a) for **24**, **25**: *tert*-butyl 4-(bromomethyl)piperidine-1-carboxylate, for **30**: *N*-(2-chloroethyl)morpholinium chloride, K_2_CO_3,_ CH_3_CN, 80 °C, 12 h; (b) (i) *ter*t-butyl 4-hydroxypiperidine-1-carboxylate, PPh_3_, THF, 20 °C, 30 min; (ii) DIAD, 0 → 20 °C, 18 h; (c) 2 M NaOH, MeOH, 18 h.

The synthesis of the final 4-hydroxypiperidines **45**–**59** is shown in Scheme 1. The first step of the synthesis was the oxidation of the double bond of 4-methylenepiperidine using *meta*-chloroperoxybenzoic acid (*m*CPBA), forming an epoxide **1**. The epoxide ring of **1** was then opened with various phenols by nucleophilic substitution, yielding compounds **2**–**11**. Subsequently, the Boc protection of intermediates **2**–**11** was removed using trifluoroacetic acid to obtain amines **12**–**21**. The latter were coupled with carboxylic acids **26**, **29,** and **31** (Scheme 2) using EDC and HOBt coupling reagents. Compound **26** was used to form **32**–**37** and **40**–**43**, compound **29** was used to synthesize **38** and **39**, and compound **31** was used to prepare the final compounds **58** and **59**. Compound **44** was synthesized by reductive amination between **13** and **24** in the presence of NaCNBH_3_. The final step in the synthesis of the final compounds **45**–**57** was the removal of the Boc protecting group using acidolysis.

The synthesis of building blocks containing phenyl ring C and piperidine ring D, that were used for preparation of final compounds **45**–**59**, is shown in Scheme 2. The starting compound was 4-hydroxybenzaldehyde (**22**) or methyl 4-hydroxybenzoate (**23**). To synthesize compounds **24**, **25**, and **30**, a nucleophilic substitution was carried out with an appropriate alkyl halide. To obtain compounds **27** and **28**, a Mitsunobu reaction was performed. The final step in the synthesis of building blocks **26**, **29,** and **31** was hydrolysis of esters **25**, **28,** and **30**, respectively.

**Scheme 3:**
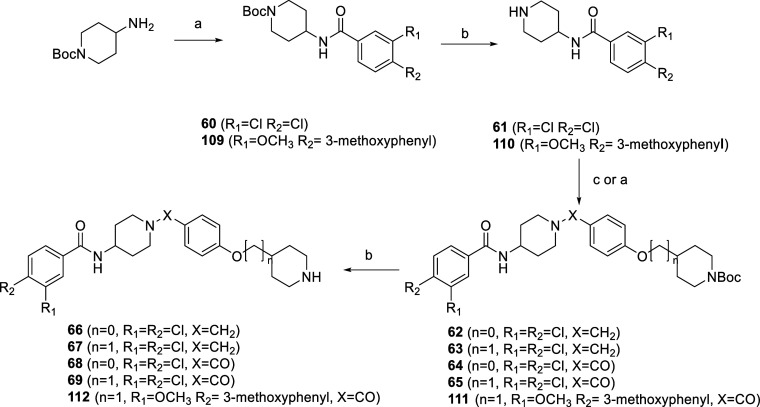
Synthesis of the Final Compounds **66–69** and **112** Reagents and conditions: (a) for **60**: 3,4-dichlorobenzoic acid, for **109**: **108**; for **64**: **29**, for **65**, **111**: **26**, EDC, HOBt, NMM, DMF, 0 °C → r.t, 18 h; (b) CF_3_COOH, DCM, 18 h; (c) for **62**: **27**, for **63**: **24**, NaCNBH_3_, MeOH, 18 h.

**Scheme 4:**
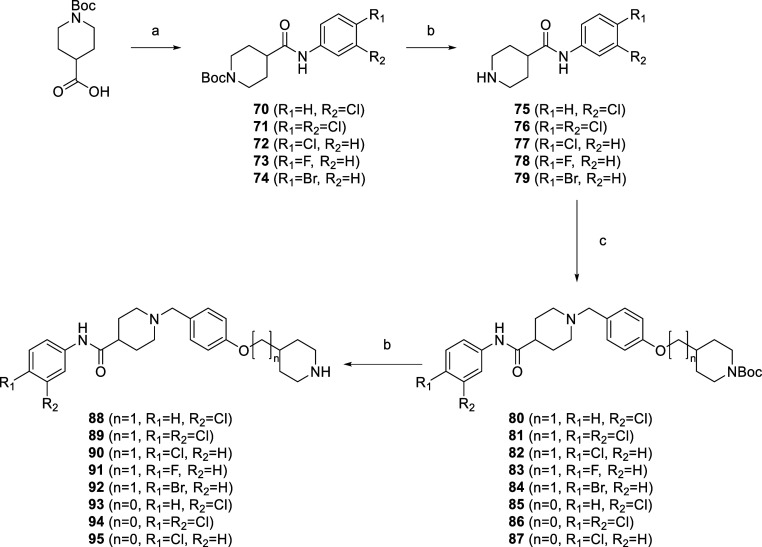
Synthesis of the Final Compounds **88–95** Reagents and conditions: (a) for **70**: 3-chlorobenzoic acid, for **71**: 3,4-dichlorobenzoic acid, for **72**: 4-chlorobenzoic acid, for **73**: 4-fluorobenzoic acid, for **74**: 4-bromobenzoic acid, EDC, HOBt, NMM, DMF, 0 °C → r.t, 18 h; (b) CF_3_COOH, DCM, 20 °C, 18 h; (c) for **80**–**84**: **24**, for **85**–**87**: **27**; NaCNBH_3_, acetic acid, MeOH, 20 °C, 18 h.

The synthesis of compounds bearing amide bond linker between phenyl ring A and piperidine ring B (**66**–**69**, **88**–**95**, and **112**) is shown in Schemes 3 and 4. The first step of the synthesis was EDC/HOBt-promoted coupling, which yielded the building blocks containing rings A and B of the final compounds (**60**, **70**–**74**, and **109**). Subsequently, the Boc protecting group was removed using trifluoroacetic acid (**61**, **110**, and **75**–**79**), followed by reductive amination to obtain compounds **62**, **63**, and **80**–**87** or amide coupling (EDC/HOBt) to give compounds **64**, **65**, and **111**. Finally, Boc deprotection of the piperidine ring was carried out to obtain the final compounds.

**Scheme 5:**
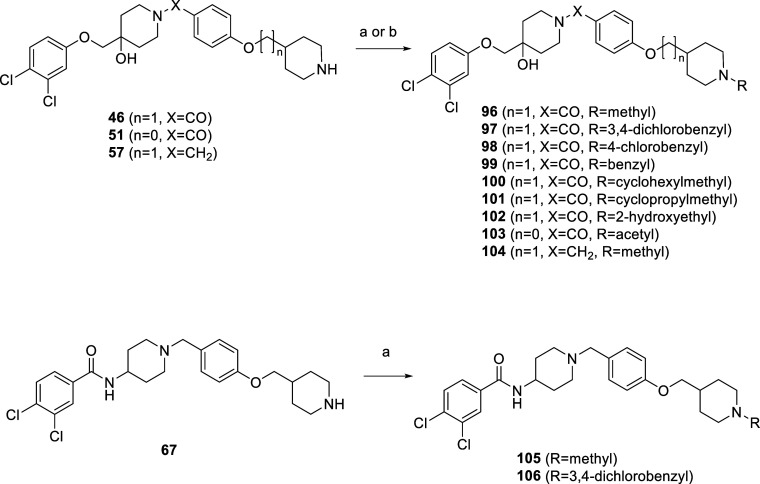
Synthesis of the Final Compounds **96**–**106** Reagents and conditions: (a) for **96**, **104**, and **105**: formaldehyde, for **97** and **106**: 3,4-dichlorobenzaldehyde, for **98**: 4-chlorobenzaldehyde, for **99**: benzaldehyde, for **100**: cyclohexanecarbaldehyde, for **101**: cyclopropanecarbaldehyde, NaCNBH_3_, CH_3_COOH, MeOH, 20 °C, 18 h; (b) for **102**: 2-chloroethanol, DIPEA, acetonitrile, 2 h, 120 °C, microwave reactor; (c) for **103**: acetanhydride, NaHCO_3_, ethyl acetate, 1 h, 20 °C.

To investigate how different substituents on the piperidine ring D affect antiproliferative activity, the final compounds **96**–**106** were synthesized, as shown in Scheme 5. Reductive amination in the presence of NaCNBH_3_ was used to prepare the final compounds **96**–**101** and **104**–**106**, while **102** was prepared by alkylation and **103** by acetylation.

**Scheme 6:**
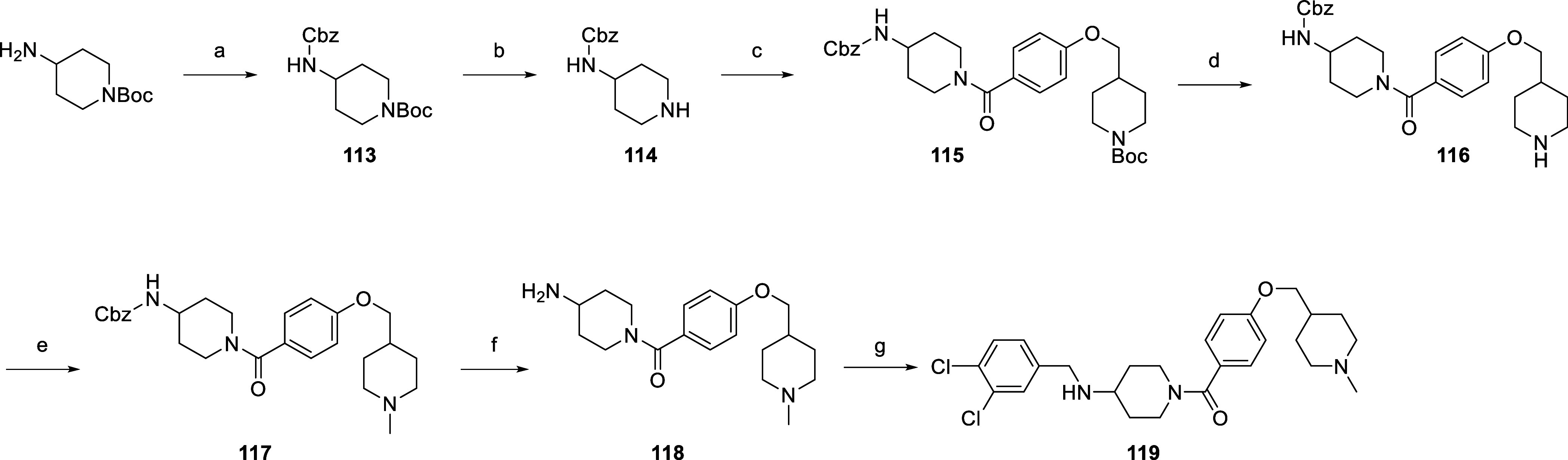
Synthesis of the Final Compound **119** Reagents and conditions: (a) benzyl chloroformate, DIPEA, DCM, 20 °C, 18 h; (b) CF_3_COOH, DCM, 20 °C, 18 h; (c) compound **18**, EDC, HOBt, NMM, DMF, 0 → 20 °C, 18 h; (d) CF_3_COOH, DCM, 20 °C, 18 h, (e) formaldehyde, NaCNBH_3_, CH_3_COOH, MeOH, 20 °C, 18 h; (f) H_2_, Pd/C, MeOH, 20 °C, 18 h; (g) 3,4-dichlorobenzaldehyde, NaCNBH_3_, CH_3_COOH, MeOH, 20 °C, 18 h.

The synthesis of the final compound **119** is shown in Scheme 6. First, the free amino group of *tert*-butyl 4-aminopiperidine-1-carboxylate was protected in the form of benzyl carbamate to give **113**, which was then Boc-deprotected (**114**) and coupled using EDC/HOBt to give compound **115**. Subsequently, the deprotected compound **116** was methylated to synthesize **117** and the Cbz protecting group was removed in the next step to obtain **118**. The final step of the synthesis of **119** was reductive amination with 3,4-dichlorobenzaldehyde.

### Exploring SAR of TVS21 Analogs

Due to the lack of appropriate biochemical assays that would enable high throughput screening, phenotypic assays are still state-of-the-art in the field of Hsp90 CTD inhibitors.^47−50^ Various factors can influence the effects of compounds on cell viability, including their desired on-target effects, potential off-target actions, cell permeability, efflux, and the sensitivity of specific cell lines to treatment. Given that the goal of this study was to enhance the in vitro anticancer activity of the starting compound, the SAR for **TVS21** analogs as Hsp90 CTD inhibitors was established based on their effects on the viability of MCF-7 breast cancer cells. In addition, selected compounds were studied with additional assays to further confirm their binding to Hsp90 CTD. The results of anticancer activity on the MCF-7 cell line of compounds **45**–**59** and **96**–**104**, which bear an ether linker between phenyl ring A and piperidine ring B, are presented in Table 1.

**Table 1:**
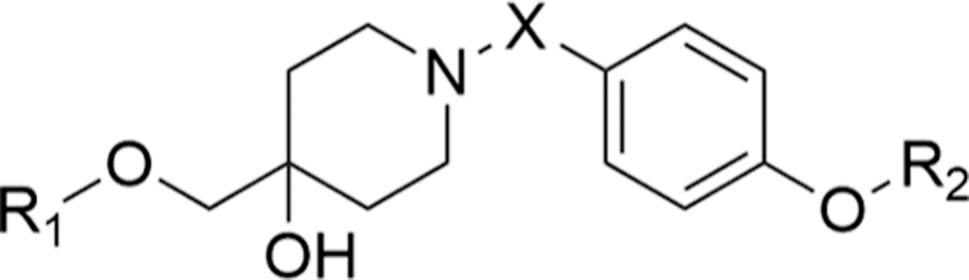

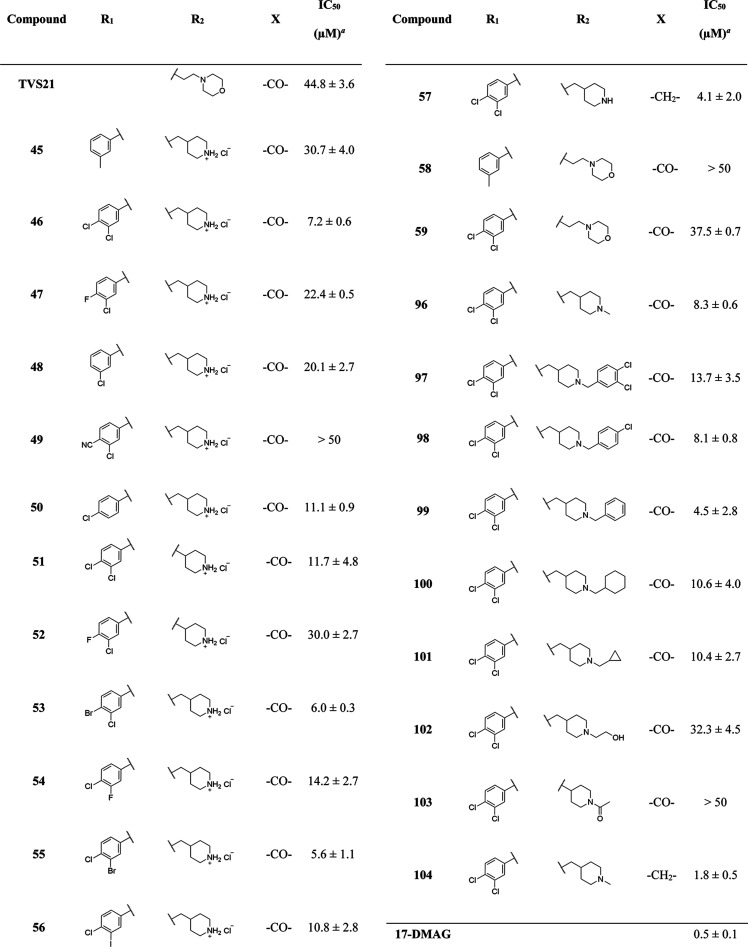
Anticancer Activity of Compounds 17-DMAG, **TVS21**, **45**–**59,** and **96**–**104** Bearing an Ether Linker on the MCF-7 Breast Cancer Cell Line^a^

a Anticancer activity was determined by MTS assay.

b Cells were treated with the Hsp90 CTD inhibitor or DMSO (vehicle) for 72 h. IC_50_ values are reported as mean ± SD of triplicates.

In an effort to determine the optimal distance between the phenyl ring C and the ionizable amine on ring D, we first increased the distance and reduced the flexibility by replacing the 4-ethylmorpholine with 4-methylpiperidine, which greatly improved the activity. Comparison of compounds **58** and **59**, which contain a morpholine ring, with the piperidine ring-bearing compounds **45** and **46** revealed that the latter compounds exhibit superior inhibitory activity. Shortening the distance by one carbon atom from 4-methylpiperidine (**46** and **47**) to piperidine (**51** and **52**) resulted in a slight decrease in potency. We then examined the effects of substitution on the phenyl ring A. Replacing the methyl group in the meta position in **45** with a chloro substituent in **48** improved the activity, while introducing an additional chlorine atom in the para position in **46** improved the activity 3-fold. Compound **49**, which has a 3-chloro-4-cyanophenyl substituent, was inactive, suggesting that polar substituents at the para position are unfavorable. We also investigated the possibility of a halogen bond between the halogen atoms on the phenyl ring A and the Hsp90 CTD binding site. Compounds **52** and **54**, which have fluorine substituents, showed lower activity than **46** with 3,4-dichloro substitution or compounds **53** and **55** with bromo substituents. However, the activity of compound **56** with iodine was lower, indicating that the halogen bond is not a significant interaction and that hydrophobic interactions have a more significant effect on the activity. We further investigated the effect of substituents of the piperidine ring D nitrogen (**96**–**104**). Our results demonstrate that the ionizable amine is a key pharmacophore feature as compound **103**, which has an amide instead of an amine, was inactive. The introduction of small aliphatic nonpolar groups, such as methyl (**96**), cyclohexylmethyl (**100**), or cyclopropylmethyl (**101**), had a modest effect on activity compared to the unsubstituted piperidine. However, the introduction of a benzyl group increased the activity 2-fold (**99**), whereas substituted benzyl rings had a minimal effect on activity, which may indicate an unfavorable steric fit. Polar groups at the piperidine NH significantly decreased activity (**102**). Notably, replacing the carbonyl group between piperidine ring B and phenyl ring C with a methylene group resulted in increased antiproliferative activity (**96** vs **104**).

The inhibitory activity of compounds with the amide bond between phenyl ring A and piperidine ring B is presented in Tables 2 and 3. The SAR found for compounds with ether linkers (Table 1) is similar to the SAR of compounds with the amide bond linkers (Tables 2 and 3). It was observed that 3,4-dichloro substitution on phenyl ring A facilitated the most favorable interactions, as can be seen when comparing compound **89** with 3,4-dichloro substitution to compound **88** with 3-chloro substitution on phenyl ring A. Compound **66** with a methylene bridge exhibited an IC_50_ in the low micromolar range, whereas compound **68** with a carbonyl bridge was inactive. A similar effect can be observed when comparing compounds **67** and **69**. Compound **67**, which contains a methylene bridge, exhibits nearly a 10-fold higher activity. This suggests that a tertiary amine in the core of the molecule is preferred over an amide group. The presence of nonpolar substituents at the N-terminus had a limited impact on activity (**105** and **106**) compared to the free amine (**67**). We also explored the possibility of halogen bond formation in compounds containing the amide bond linker. The results showed that compound **91** with the fluorine substituent on phenyl ring A displayed lower activity compared to compounds **90** and **92** with the chlorine substituent and bromine substituent, respectively. This may indicate a halogen bond, but on the other hand, compounds **90** and **92** have comparable inhibitory activities, indicating that hydrophobic interactions play a more important role in binding to Hsp90 CTD than a halogen bond, as was also observed in compounds bearing the ether linker.

**Table 2:**
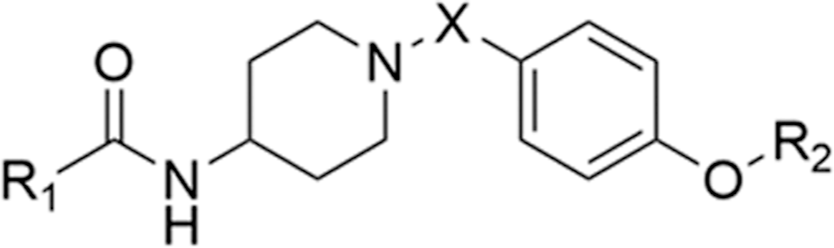

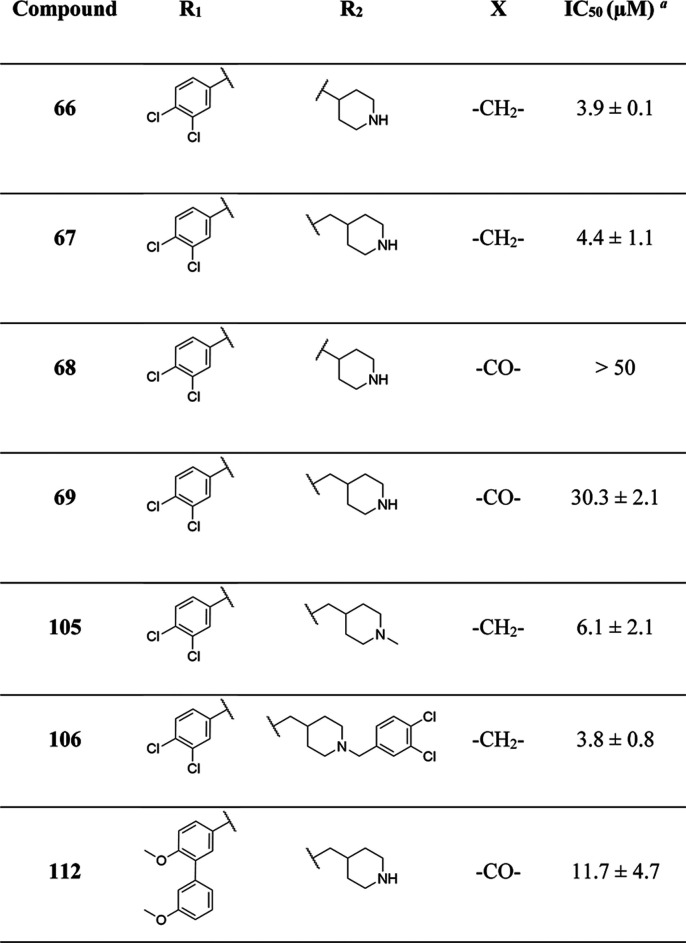
Anticancer Activity of Compounds **66**–**69**, **105**, **106,** and **112** Bearing an Amide Bond Linker on the MCF-7 Breast Cancer Cell Line^a^

a Anticancer activity was determined by MTS assay.

b Cells were treated with the Hsp90 CTD inhibitor or DMSO (vehicle) for 72 h. IC_50_ values are reported as mean ± SD of triplicates.

**Table 3:**
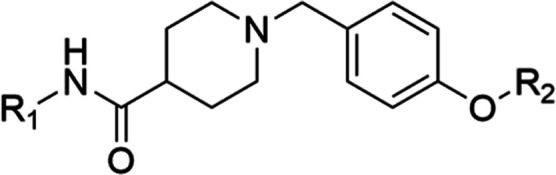

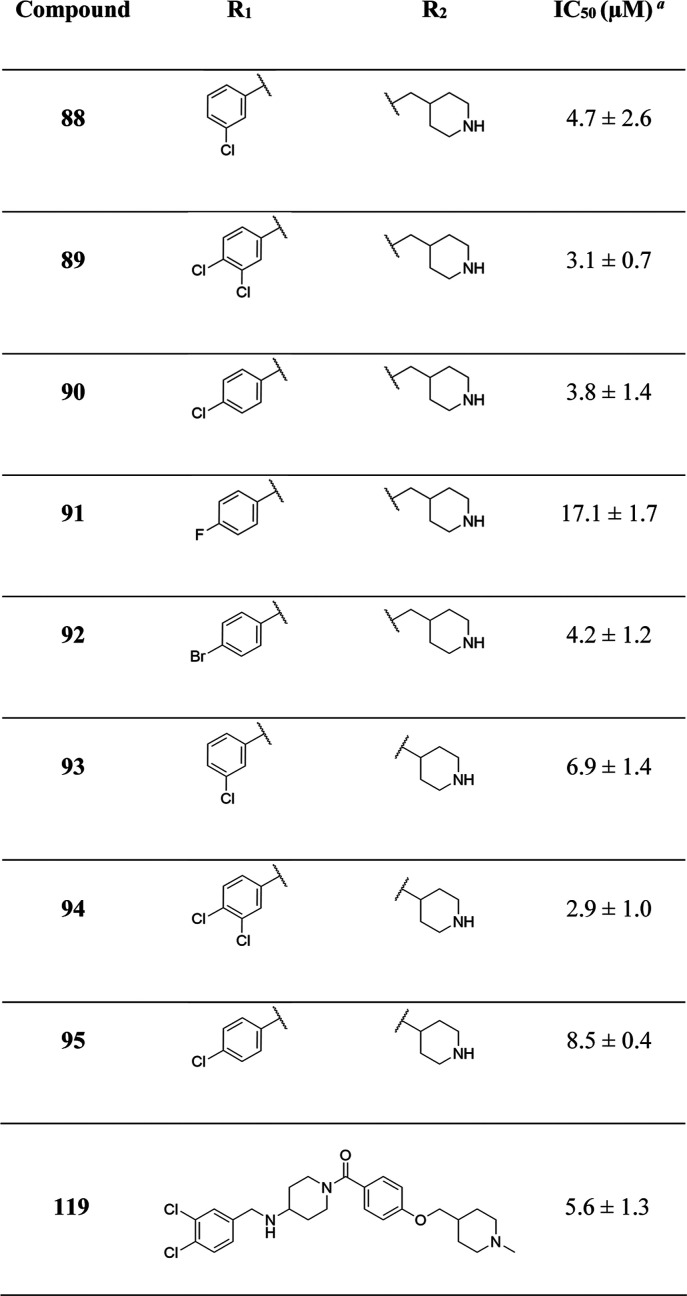
Anticancer Activity of Compounds **88**–**95** and **119** Bearing an Amide Bond Linker on the MCF-7 Breast Cancer Cell Line^a^

a Anticancer activity was determined by MTS assay.

b Cells were treated with the Hsp90 CTD inhibitor or DMSO (vehicle) for 72 h. IC_50_ values are reported as mean ± SD of triplicates.

Overall, our results suggest that a methylene linker between rings B and C is preferred over a carbonyl linker, and the linker between rings A and B must be capable of forming a hydrogen bond. The phenyl ring A forms significant hydrophobic interactions with the binding site residues, and hydrophobic substituents can enhance activity, particularly the 3,4-dichloro substitution. An ionizable amine is essential for activity and the 4-methylpiperidine moiety offers the optimal distance between the phenyl ring A and the basic center on ring D. The observed SAR is in agreement with the proposed binding mode of the starting compound **TVS21** in the Hsp90 CTD binding site (Figure 2A).

### Evaluation of Binding of TVS21 Analogs to Hsp90

**Figure 3:**
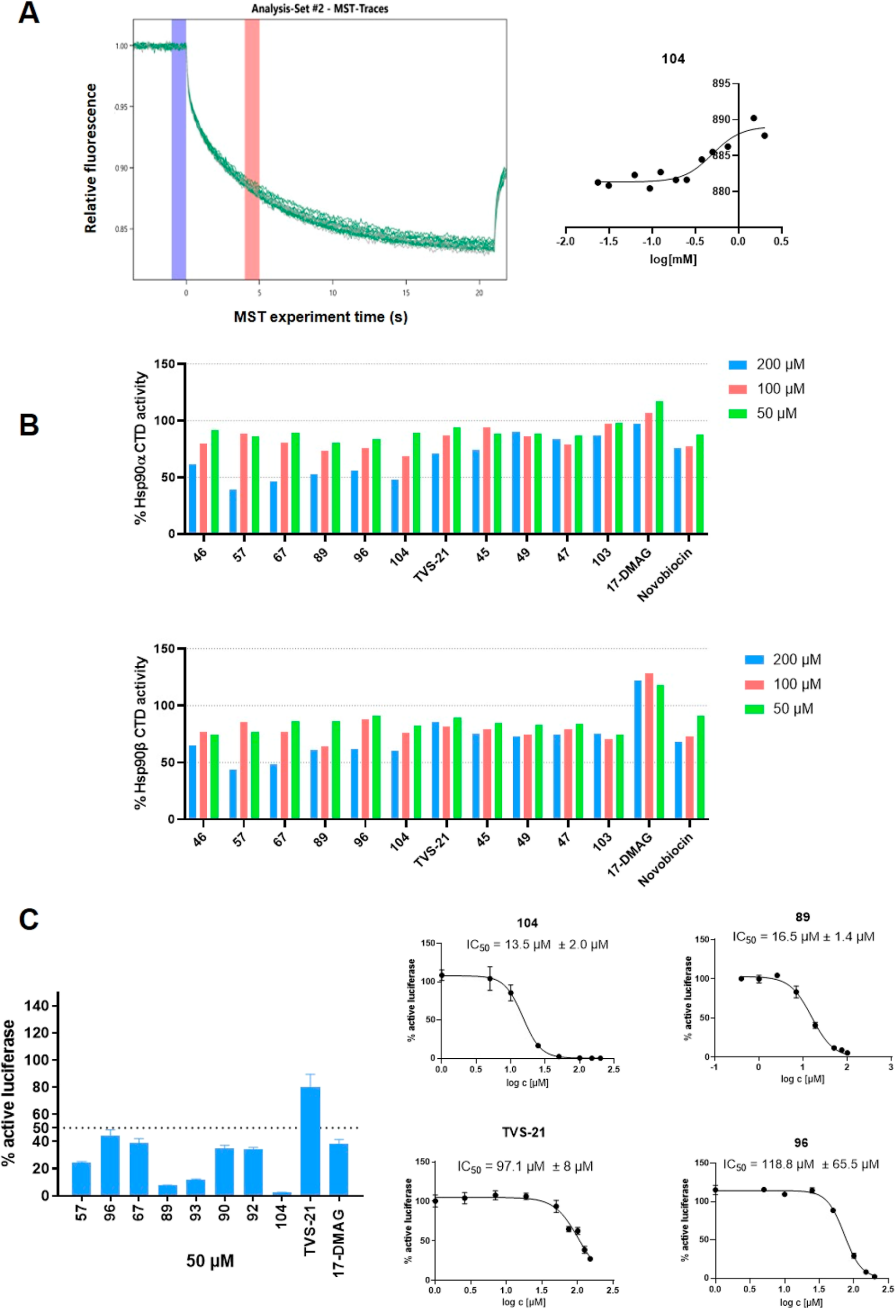
(A) Determination of the apparent *K*_d_ value of compound **104** on the full-length Hsp90β using microscale thermophoresis. Left plot shows MST curves. *K*_d_ value is a mean ± SD of two independent experiments. (B) Inhibitory effect of new Hsp90 CTD inhibitors, novobiocin (Hsp90 CTD inhibitor), and 17-DMAG (Hsp90 NTD inhibitor) at 200, 100, and 50 μM determined by TR-FRET assay. Data shown are means ± SD of two independent experiments. (C) (Left) Luciferase refolding activity of Hsp90 in PC3MM2 cells after treatment with **TVS21** analogs and 17-DMAG at 50 μM concentrations. Data are means ± SD of three independent experiments performed in triplicates. (Right) representative IC_50_ curves of luciferase refolding activity of compounds **TVS21**, **89**, **96**, and **104**. The IC_50_ values are means ± SD of two independent experiments performed in triplicates.

To confirm that **TVS21** analogs exert their biological activity through Hsp90 inhibition, several assays were performed. The binding affinities of novobiocin (positive control, *K*_d_ = 1089 ± 60 μM) and compound **104** to the full-length Hsp90β (*K*_d_ = 490 ± 10 μM) were determined using microscale thermophoresis (MST) (Figure 3A). **TVS21** analogs were designed as allosteric CTD inhibitors, which bind to the closed conformation of the Hsp90 dimer after ATP binds to the NTD.^35,36^ This may be a reason why **TVS21** analogs, such as **104**, appear to be weak binders of Hsp90 in biochemical assays in the absence of ATP. To exclude binding to the Hsp90α and Hsp90β NTD, a fluorescence-based thermal shift assay (FTSA) was performed for compounds **TVS21**, **89,** and **104**. As expected, no binding to the Hsp90α and Hsp90β NTD was detected at concentrations up to 500 μM. To confirm that our new Hsp90 inhibitors bind to the CTD, a screening assay was performed targeting both Hsp90α and Hsp90β CTDs. Inhibition of Hsp90 CTD binding to its target protein cyclophilin D (PPID) was investigated using the TR-FRET technique as previously reported.^37,38,51^ Several representative compounds were screened for their ability to inhibit the binding of PPID to Hsp90 CTD at 200, 100, and 50 μM. Compounds with more potent activity in the MCF-7 cell line (**46**, **57**, **67**, **89,** and **104**) displayed an inhibitory effect of about 50% on PPID binding to the Hsp90α or Hsp90β CTD at a concentration of 200 μM, whereas the compounds with weaker activity (**TVS21**, **45**, and **47**) displayed a lower inhibitory effect. Compound **49**, which showed no biological activity in the MTS assay, was unable to inhibit Hsp90 CTD (Figure 3B). An established Hsp90 CTD inhibitor, novobiocin, was used as a positive control, while the Hsp90 NTD inhibitor, 17-DMAG, was used as a negative control. With these assays, we confirmed that our novel Hsp90 inhibitors exert their activity by binding to the CTD and not NTD without selectivity for Hsp90α or Hsp90β.

To further evaluate the ability of **TVS21** analogs to inhibit Hsp90 chaperone function, a luciferase refolding assay in PC3MM2 cell line^52^ was carried out. Exposure of **TVS21** and its more potent analogs at 50 μM decreased the luciferase refolding ability (Figure 3C). The known Hsp90 NTD inhibitor 17-DMAG served as a positive control. A dose–response experiment was carried out for **TVS21** and its analogs **89**, **96**, and **104**. Compounds **89** and **104** showed stronger ability to inhibit luciferase refolding compared to **TVS21**, with IC_50_ values of 16.5 ± 1.4 and 13.5 ± 2.0 μM, respectively.

### NMR Binding Studies of Compounds **89**, **96**, and **104** to Full-Length Hsp90

**Figure 4:**
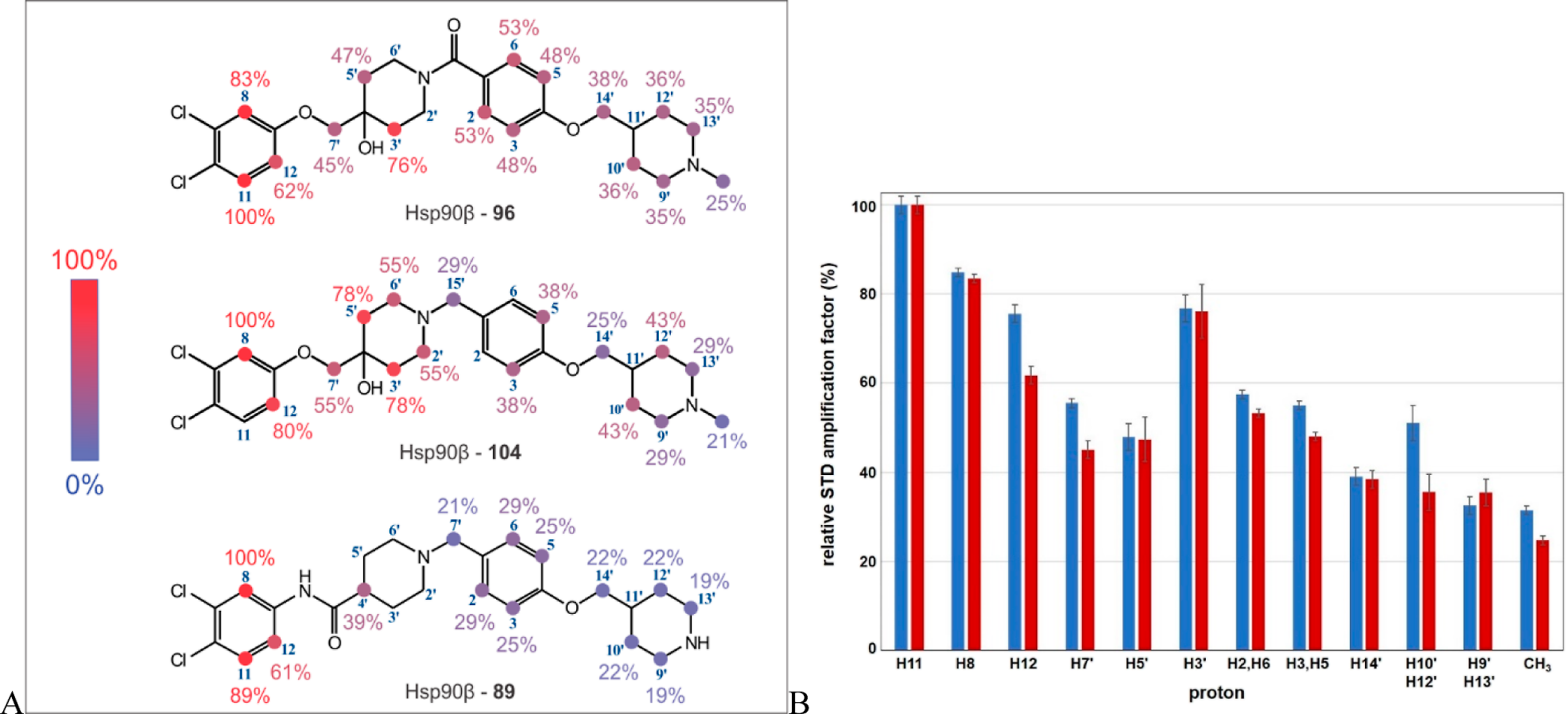
(A) Binding epitope mapping by 1D ^1^H STD NMR spectroscopy for compounds **96**, **104**, and **89** at a Hsp90β/ligand/AMP-PCP ratio of 1:200:200. Molecular structures with assignments to the proton signals are given. Relative degrees of saturation for the individual protons are presented in a gradient color. The values were normalized to the intensity of the signal with the largest STD effect. (B) Relative degrees of saturation of the individual protons of 96 in the presence of Hsp90β (in red) and Hsp90α (in blue) determined from 1D ^1^H STD spectra recorded at an Hsp90/ligand/AMP-PCP ratio of 1:200:200. The values in each molecule were normalized to the intensity of the signal with the largest STD effect. The signals of the protons not shown were overlapped with the buffer signals in the reference experiment. The proton nomenclature corresponds to the atom nomenclature shown in (A).

In the absence of the cocrystal structure of the Hsp90-CTD inhibitor complex, we aimed to obtain additional experimental data on the binding of our inhibitors to Hsp90 using ligand-based NMR methods, namely, saturation transfer difference (STD) and transferred NOESY (trNOESY) experiments. The binding of compounds **89**, **96,** and **104** to full-length Hsp90β was first investigated using STD NMR under quantitative conditions. This means that the influence of the ^1^H T_1_ relaxation times of the ligands on the STD amplification factors was suppressed (Table S2). Several approaches have been proposed to overcome this effect.^53,54^ Our previous studies have shown that for large proteins with a molecular weight greater than 45 kDa, the short saturation times proposed by Yan et al.^54^ successfully overcame the effect of T_1_ on STD amplification factors, while STD ligand epitope maps consistent with ligand binding modes were obtained.^55−57^ Since the molecular weight of Hsp90 is above 85 kDa, we used the same approach in the present study. The 1D ^1^H STD spectra of **89**, **96**, and **104** in the presence of AMP-PCP and Hsp90β or Hsp90α were obtained with a ligand/protein ratio of 200:1. The spectra are shown in Figures S2–S6 in the Supporting Information. The binding of inhibitors **89**, **96**, and **104** to Hsp90β was strongly mediated by the aromatic protons of the 3,4-dichlorophenyl moiety (ring A), which showed the greatest saturation transfer (Figure 4A), suggesting that this structural part of the inhibitors makes the strongest contact with the Hsp90 binding site. This observation is consistent with the molecular modeling studies, in which the 3,4-dichlorophenyl moiety is tightly bound and stabilized in the binding site by a network of hydrophobic interactions (Supporting Information, Figures S12 and S13). In general, the STD amplification factors decreased from ring A toward ring D and were weakest for the protons of piperidine ring D. Compounds **96** and **104**, which differ only in the linker between rings B and C, showed a comparable mapping of the binding epitopes. A similar trend was also observed for **89**, which had the lowest STD amplification factors for protons of ring D. Although the piperidine ring D seems relatively unimportant based on the STD amplification factors of its protons, it bears the basic center, which appears to be critical for the potent activity of the compounds in the MTS assay (compound **96** vs **103**) and its small modifications significantly alter the IC_50_ values in MCF-7 cell line (compound **96** vs **102**). Furthermore, the STD epitope mapping for **96** was comparable in the presence of Hsp90β or Hsp90α (Figure 4B), consistent with the results of the TR-FRET assay and molecular docking studies (see Supporting Information for further details). Moreover, the STD epitope map of **96** differed in the absence or presence of AMP-PCP (resulting in open and closed conformation of the Hsp90α dimer, respectively) (Figure S10).

**Figure 5:**
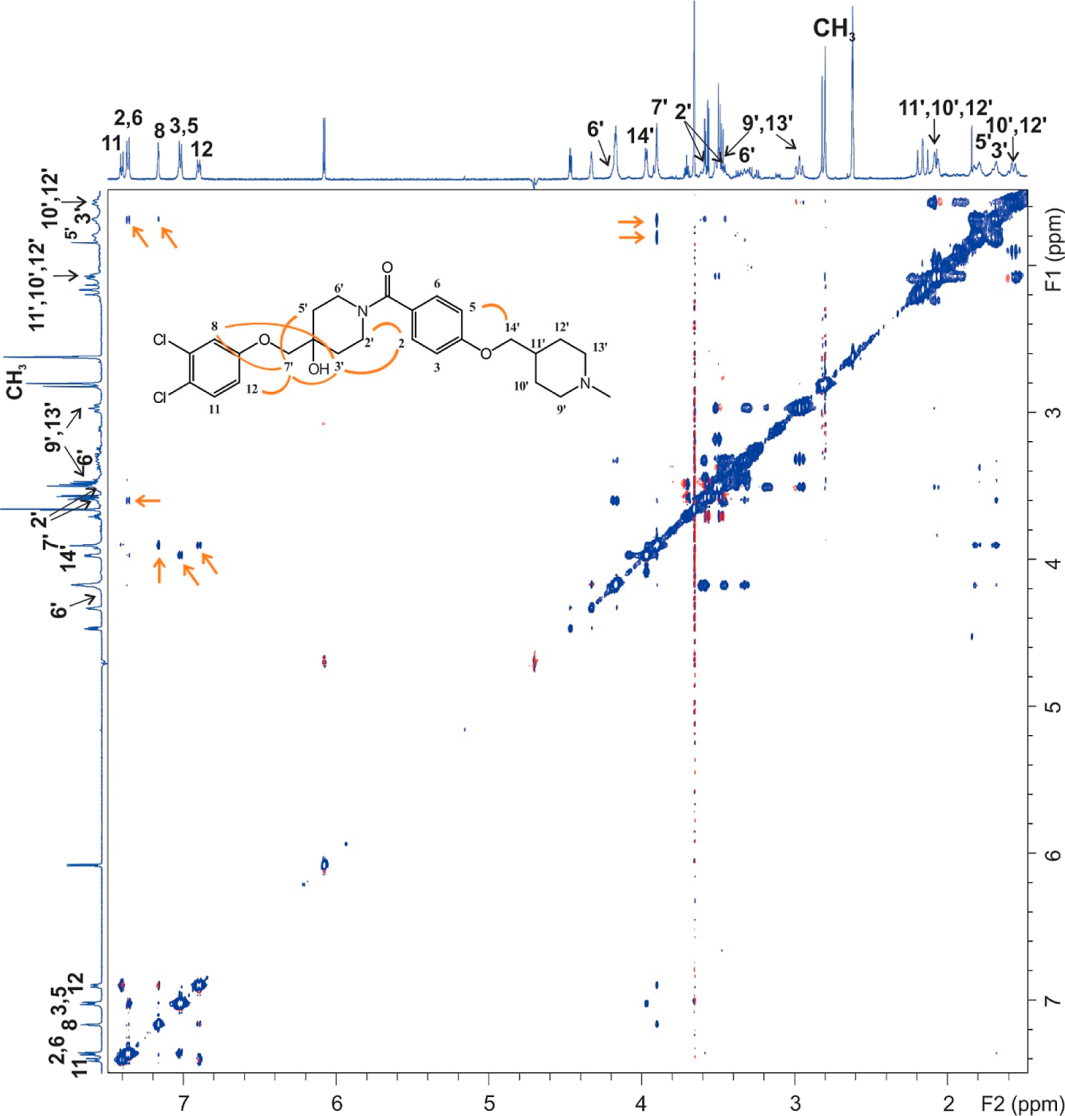
TrNOESY spectrum of **96** in the presence of Hsp90β with the molecular structure illustrating the atom nomenclature and the NOE connectivities between the protons of the different molecular segments. Corresponding NOEs are marked with arrows. Note that the NOE connectivities of the magnetic equivalent protons 2,6 and 3,5 are schematically shown only for one orientation of the corresponding aromatic ring.

**Figure 6:**
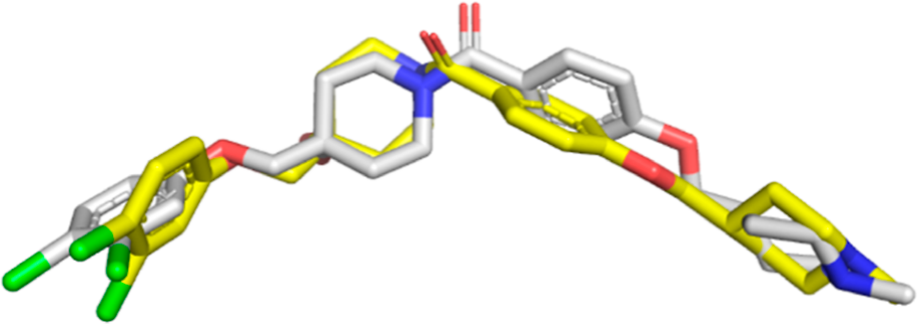
Overlay of the conformations of **96** from the most representative pharmacophore model in 500 ns MD simulation trajectory (in gray) and from trNOESY experiment (in yellow).

Binding of inhibitors **89**, **96**, and **104** to Hsp90β was further confirmed by trNOESY experiments, as negative NOEs with the same sign as the diagonal peaks were observed between the protons of each inhibitor in the presence of Hsp90β (Figures S7–S8 and S11). The NOEs were observed only between adjacent molecular segments (Figures 5 and S7 and S8), suggesting that all three inhibitors adopt an extended conformation when bound to Hsp90β, consistent with the SAR analysis and molecular modeling studies. Due to the greater overlap of the proton signals of compounds **104** and **89**, particularly ring B, the application of NOE-derived distances in molecular modeling was only feasible for compound **96**. The calculated conformation of **96** using the distance constraints from trNOESY spectrum resembles the most common conformation of **96** in complex with Hsp90β observed during the 500 ns MD simulation trajectory (Figure 6). The same NOE pattern as in the presence of Hsp90β is observed for **96** also in the presence of Hsp90α (Figure S9), confirming the observations from STD experiments. All-atom RMSD value between the MD- and trNOESY-derived conformation of **96** was 1.86 Å. The distance between the centroid of phenyl ring A and the basic nitrogen in ring D in the calculated conformation of **96**, based on the constraints of the trNOESY experiment, was 16.6 Å (Figure S14). This distance is also consistent with our previously described ligand-based pharmacophore model based on the most potent Hsp90 CTD inhibitors, in which the distance between the aromatic ring and a basic center was 16.9 Å.^41^ Moreover, the distance between the methylene group between rings A and B and the methylene group between rings C and D was 9.7 Å (Figure S14), which is in good agreement with previous work showing that the optimal distance between the *N*-methylpiperidine and the biaryl side chain of novobiocin analogs is 7.7 to 9.6 Å.^58^ We can conclude that the appropriate distance between the 3,4-dichlorophenyl moiety (ring A) and the basic nitrogen of piperidine ring D is needed for achieving potent antiproliferative activity in breast cancer cell lines.

### Effect of New Hsp90 CTD Inhibitors on Viability of Various Breast Cancer Cell Lines

**Figure 7:**
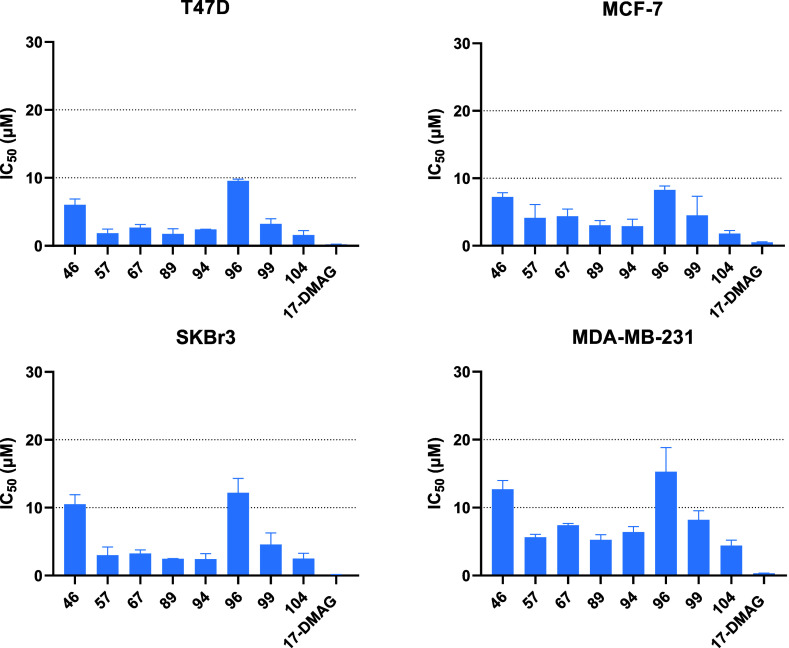
IC_50_ values of Hsp90 CTD inhibitors in hormone-dependent cell lines MCF-7 and T47D, HER2-overexpressing cell line SKBr3, and MDA-MB-231 a TNBC cell line. Hsp90 NTD inhibitor 17-DMAG was used as a positive control in all breast cancer cell lines. Data shown are means ± SD of three independent experiments.

Eight newly synthesized **TVS21** analogs were chosen as representative compounds from all three different linker types with comparable IC_50_ values on the MCF-7 cell line and evaluated for their cytotoxic activity on various breast cancer cell lines (Figure 7 and Table S1) using an MTS assay. We chose breast cancer cell lines from all three major subtypes of breast cancer, two hormone dependent breast cancer cell lines MCF-7 and T47D, a HER2-overexpressing cell line SKBr3, and a TNBC cancer cell line MDA-MB-231. Our new Hsp90 CTD inhibitors were effective against all four breast cancer cell lines in the low micromolar range, which shows an advantage of Hsp90 inhibition, as we can target different subtypes of breast cancer with the same strategy. When comparing the activity of compounds on assayed cancer cell lines, no distinct difference between hormone-dependent breast cancer cell lines and HER2-overexpressing cell line was observed. Most of our tested compounds showed slightly weaker activity in the TNBC cancer cell line, but due to the scarcity of available treatments for TNBC, this breast cancer model was chosen for further biological evaluation.

### Compounds **89** and **104** Induce Apoptosis and Inhibit Proliferation of TNBC Cells

**Figure 8:**
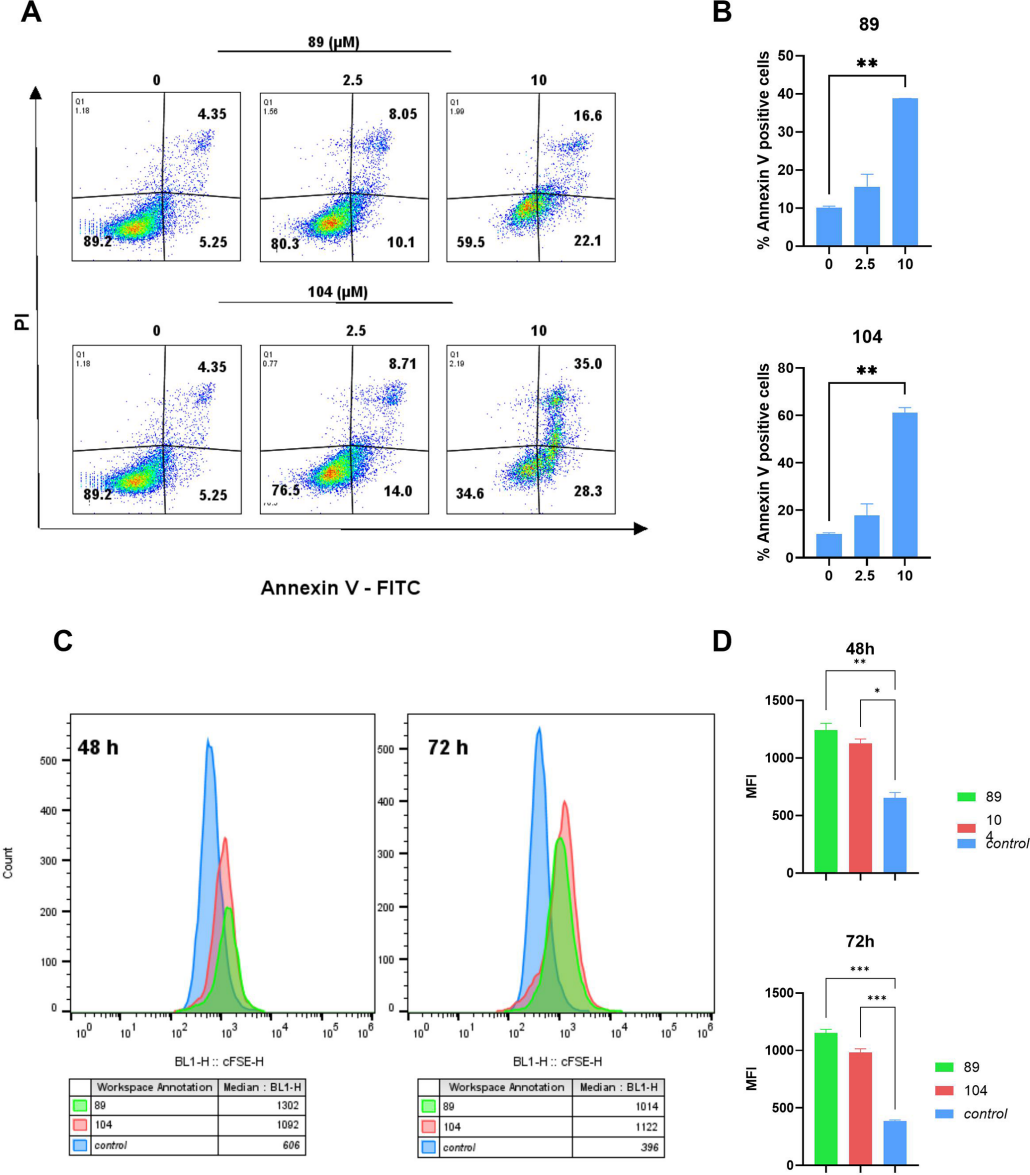
(A) MDA-MB-321 cells were treated with **89** and **104** (2.5 and 10 μM) for 72 h. Staining with Annexin V/PI was carried out to assess early and late apoptosis with flow cytometry; (B) percentages of Annexin V positive cells (right panel, Q2 and Q3) (apoptotic) are shown as bar graphs. Data shown are means ± SEM of two independent experiments. Statistical significance between treated versus control group (vehicle) was calculated using one-way ANOVA post hoc Dunnett’s test. (**p < 0.01; *p < 0.05; NS not significant); (C) MDA-MB-231 cells were treated with **89** and **104** (10 μM) for 48 and 72 h. Cells were stained with CFSE to monitor their proliferation with flow cytometry. Graph shows histograms of CFSE fluorescence of cells treated with **89** (in green), **104** (in red), and nontreated cells (in blue) as well as the medians of CFSE fluorescence (MFI); (D) MFI represented as bar graphs. Data shown are means ± SEM of two independent experiments. Statistical significance between treated versus control group (vehicle) was calculated using one-way ANOVA post hoc Dunnett’s test. (***p < 0.001; **p < 0.01; *p < 0.05; NS not significant).

Exposure of MDA-MB-231 TNBC cells to compounds **89** and **104** resulted in cytotoxicity in a dose-dependent manner observed by MTS assay. To further explore whether this effect was due to induction of apoptosis, an Annexin V/propidium iodide (PI) assay was carried out. As shown on Figure 8A,B, a significant increase in early and late apoptotic cells was observed in the presence of compounds **89** and **104** at 10 μM compared to untreated cells. These results suggest that our new Hsp90 CTD inhibitors illicit their effect by induction of apoptosis.

Additionally, we evaluated the effect of **89** and **104** on the proliferation rate of MDA-MB-231 cells with CFSE assay. As shown on Figures 8C,D and S161, proliferation of cells was significantly inhibited after treatment with compounds **89** and **104** at 10 μM. With these two assays, we confirmed that compounds **89** and **104** induce apoptosis as well as have a cytostatic effect on MDA-MB-231 TNBC cells.

### Compounds **89** and **104** Cause Degradation of Oncogenic Proteins

**Figure 9:**
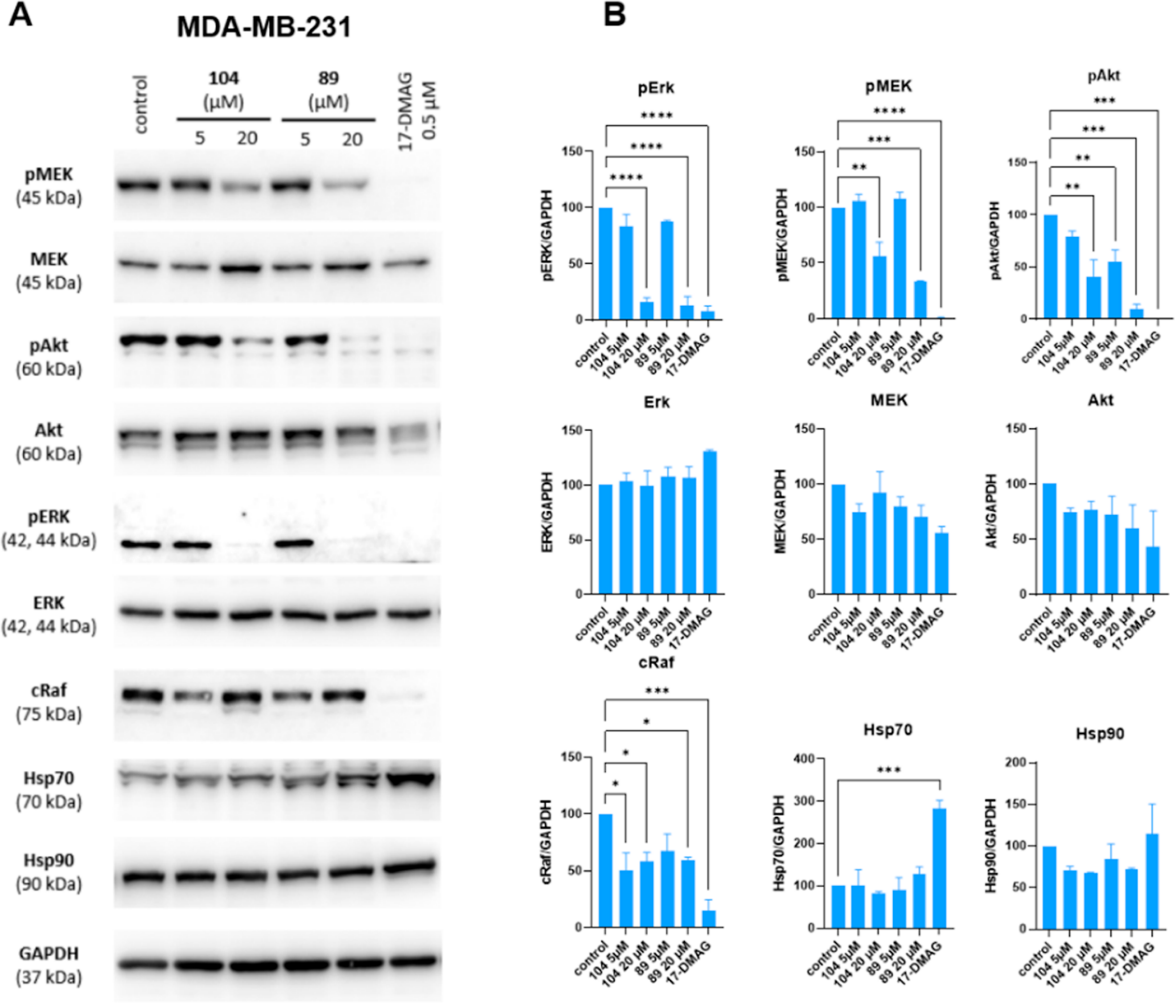

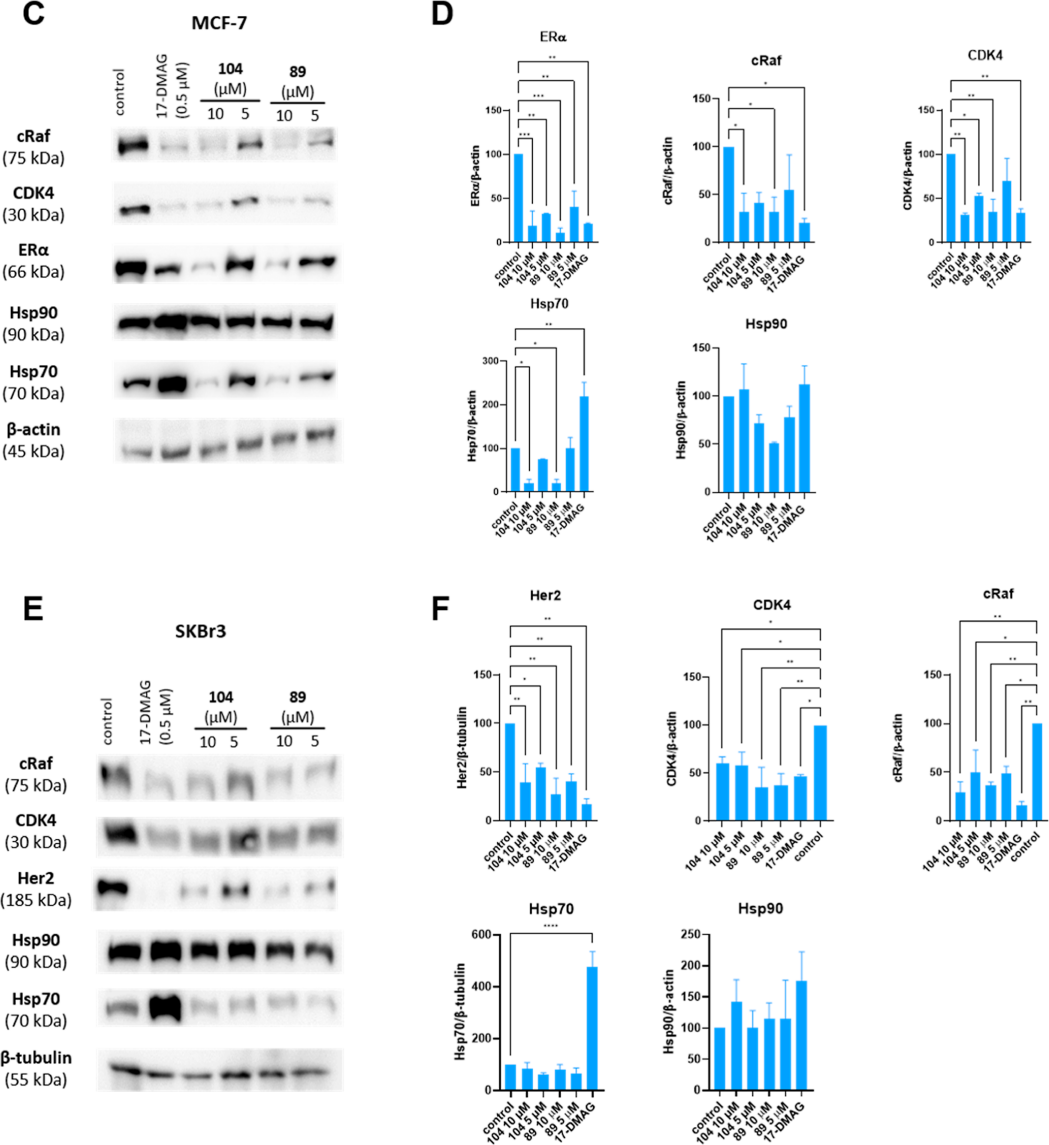
(A) Representative Western blot analyses after 24 h incubation of MDA-MB-231 cells with compounds **89**, **104**, 17-DMAG (Hsp90 NTD inhibitor), and vehicle control (0.5% DMSO); (B) quantitative graphs of protein levels represent the percentage of protein of interest to GAPDH (loading control); (C) representative Western blot analyses after 24 h incubation of MCF-7 cells with compounds **89**, **104**, 17-DMAG (Hsp90 NTD inhibitor), and vehicle control (0.5% DMSO); (D) quantitative graphs of protein levels represent the percentage of protein of interest to β-actin (loading control); (E) representative Western blot analyses after 24 h incubation of SKBr3 cells with compounds **89**, **104**, 17-DMAG (Hsp90 NTD inhibitor), and vehicle control (0.5% DMSO); and (F) quantitative graphs of protein levels represent the percentage of protein of interest normalized to β-tubulin, which was used as loading control. Data shown are means ± SEM of two independent experiments. Statistical significance between treated versus control group (vehicle) was calculated using one-way ANOVA post hoc Dunnett’s test. (****p < 0.0001, ***p < 0.001; **p < 0.01; *p < 0.05).

One of the main advantages of Hsp90 inhibition is the simultaneous down-regulation of numerous oncogenic proteins. MDA-MB-231, SKBr3, and MCF-7 cells were treated with compounds **89** and **104** and the Hsp90 NTD inhibitor 17-DMAG for 24 h. Exposure of MDA-MB-231 cells to the new Hsp90 CTD inhibitors resulted in the degradation of Hsp90 client proteins involved in various signaling pathways (Figure 9A,B). Treatment with compounds **89** and **104** significantly reduced phosphorylation of AKT, which is associated with both hyperproliferation and resistance to apoptosis.^59^ In addition, compounds **89** and **104** also decreased the concentration of oncogenic proteins involved in the RAF-MEK-ERK pathway, whose aberrant activation causes prosurvival effects as well as increased proliferation in cancer cells.^60^ In MCF-7 cells, compounds **89** and **104** markedly decreased the concentration of ERα, which is overexpressed in this cell line (Figure 9C,D). Treatment of Her2 overexpressing cell line SKBr3 with compounds **89** and **104** significantly decreased the concentration of the Her2 receptor (Figure 9E,F). Given the potential for Hsp90 CTD inhibitors to exhibit general kinase inhibition,^61^ we conducted a protein kinase panel profiling assay, which revealed no significant inhibition of the 22 kinases tested (Figure S162). The concentration levels of Hsp90 and Hsp70, which are markers for HSR, were also monitored. When the cells were treated with **89** and **104**, these levels remained unchanged. On the other hand, levels of Hsp70 were markedly increased when the cells were treated with the Hsp90 NTD inhibitor 17-DMAG, suggesting that our compounds, unlike NTD inhibitors, do not induce HSR. Interestingly, in MCF-7 cells, the levels of Hsp70 decreased when exposed to compounds **89** and **104** (Figure 9C,D).

To further assess the mechanism underlying the decreased protein concentration, we cotreated MCF-7 cells with Hsp90 CTD inhibitors and a proteasome inhibitor carfilzomib. The decrease in oncogenic protein concentration was not significant when the proteasome inhibitor was added (Figure S163), suggesting that compounds **89** and **104** inhibit the Hsp90 chaperone function, therefore preventing correct protein folding and leading to proteasomal degradation of client proteins.

### In Vivo Antitumor Efficacy of Compound **89**

**Figure 10:**
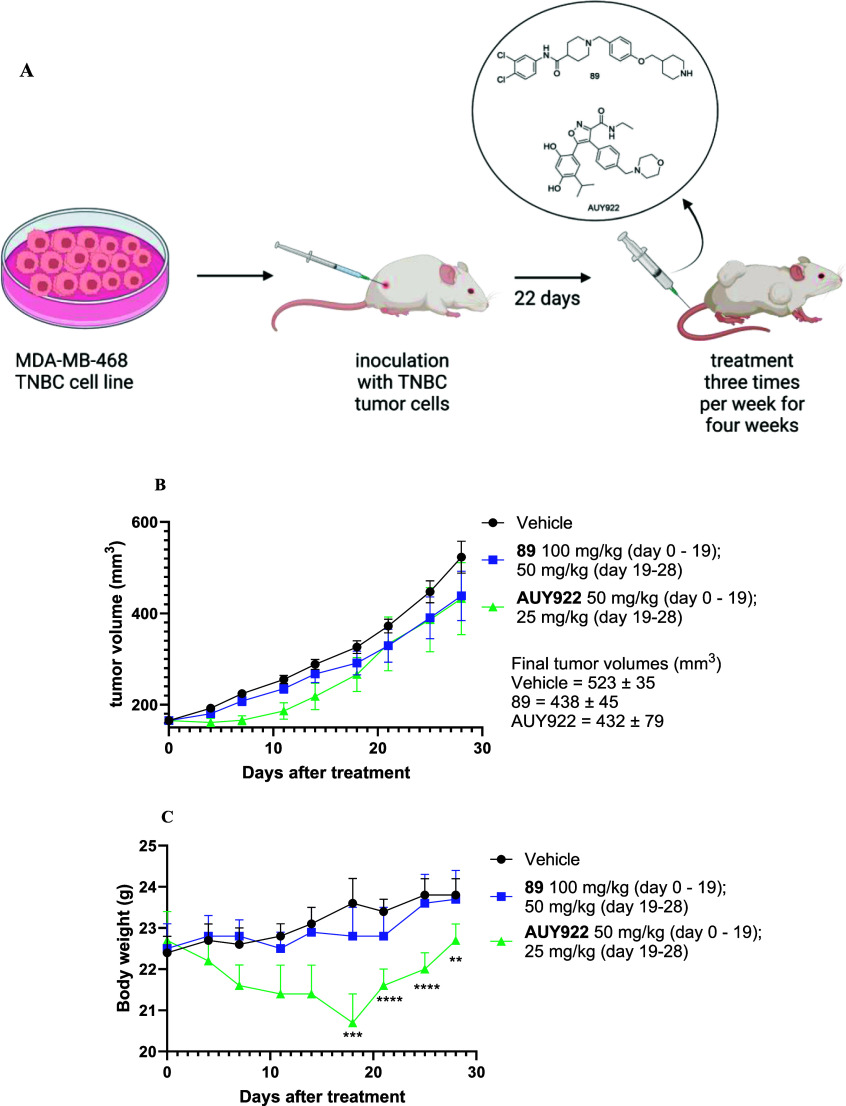
(A) BALB/c nude mice (*n* = 6 per group) were inoculated with the MDA-MB-468 TNBC cell line in the right flank. Twenty-two days after the inoculation mice were treated intravenously three times per week for 4 weeks with compound **89**, a known Hsp90 NTD inhibitor AUY922, or vehicle control; (B) tumor growth curves of different treatment groups among MDA-MB-468 bearing mice. Data points represent group mean; error bars represent standard error of the mean (SEM); (C) body weight changes of different groups in mice bearing MDA-MB-468 tumors. Data points represent group mean body weight. Error bars represent SEM.

To evaluate the in vivo relevance of in vitro findings, the impact of compound **89** on tumor growth in the TNBC MDA-MB-468 xenograft model on BALB/c nude mice was investigated (Figure 10A). Prior to selecting the xenograft model, a viability assay was conducted. In this assay, compound **89** exhibited an IC_50_ of 2.7 ± 0.1 μM in the MDA-MB-468 cell line, which is comparable to the IC_50_ value observed in our TNBC cell line MDA-MB-231 (Table S1). Before initiating the in vivo study, a formulation screening was conducted, and the most favorable formulation was determined to be a vehicle consisting of 20% sulfobutylether-β-cyclodextrin in 50 mM citrate buffer (pH = 3).

To assess safety and selective toxicity of compound **89**, a maximum tolerated dose was determined over a 4 week period. BALB/c nude mice were divided into 4 groups: vehicle, low dose (20 mg/kg), medium dose (50 mg/kg), and high dose (100 mg/kg). The mice received the vehicle or compound twice weekly for 4 weeks were observed for treatment effects such as mobility, food and water intake, and weight gain or loss. Compound **89** was found to be well tolerated at all three doses, as there were no significant changes in body weight even at 100 mg/kg (Figure S161).

The efficacy of compound **89** was compared with AUY922, a known Hsp90 NTD inhibitor, whose efficacy is well established in xenograft breast cancer models,^62^ as well as other xenograft models such as melanoma, ovarian cancer, prostate cancer, and glioblastoma.^63^ AUY922 has also advanced to clinical trials.^64^ Our study revealed comparable effects of compounds **89** and AUY922 on inhibiting tumor growth (Figure 10B). Both compounds demonstrated a reduction in tumor growth by approximately 20% (specifically, 23.86% for compound **89** and 25.32% for AUY922). The trend of tumor growth inhibition was evident for both substances in our investigation. However, it is regrettable that neither compound exhibited a statistically significant impact on inhibiting tumor growth. Notably, compound AUY922 exhibited more pronounced effects in ER-and HER2-positive breast cancer xenograft models, particularly BT-474, where it significantly slowed tumor growth at dosages 25 and 50 mg/kg.^62,63^ Considering this, there may be a possibility that our chosen xenograft model could potentially be less sensitive to treatment with Hsp90 inhibitors as single agents. Consequently, exploring compound **89** performance in vivo using an alternative xenograft breast cancer model holds promise for future investigations.

The off-target toxicity of compound **89** and AUY922 was monitored by measuring body weight loss. Mice treated with **89** experienced notably less body weight reduction than those treated with AUY922 where weight loss was statistically significant at several time points (Figure 10C). Because of the significant weight loss in the AUY922-treated group, the dose was halved in both treated groups at day 19. These findings suggest that compound **89** exhibited greater tolerance within the BALB/c nude mice model, reflecting a better safety profile than AUY922. Notably, a favorable safety profile was maintained, even though compound **89** was administered at higher dosages than AUY922. Thus, the results from this study also suggest that Hsp90 CTD inhibitors, such as compound **89**, have fewer off-target effects compared to Hsp90 NTD inhibitors, which is promising for further development of Hsp90 CTD inhibitors.

## Conclusions

In this study, we conducted a focused structure–activity relationship optimization of the previously discovered virtual screening hit **TVS21**, using our MD-derived SBPM approach. Through systematic structural modifications of **TVS21**, we synthesized a library of Hsp90 CTD inhibitors with higher anticancer activity compared to **TVS21**. In contrast to previously reported indirect evidence of inhibition, we utilized ligand-based NMR methods as well as trNOESY to unequivocally demonstrate the binding of our compounds to the Hsp90 as well as TR-FRET-based assay, MST, and FTSA to confirm binding to the Hsp90 CTD. Our compounds showed anticancer activity in different types of breast cancer cell lines. The most promising compounds **89** and **104** induced apoptosis and inhibited cell proliferation in the TNBC cell line. These compounds also downregulated the expression levels of oncogenic proteins involved in the AKT as well as RAF/MEK/ERK cancer pathways, without inducing HSR. Such a multifaceted approach targeting multiple cancer pathways promises to enhance antitumor activity, while reducing the likelihood of resistance to therapy. In addition, compound **89** demonstrated to be well tolerated in the BALB/c nude mice model with an MTD of 100 mg/kg and also exhibited a comparable trend of inhibiting tumor growth in vivo to AUY922, an established Hsp90 NTD inhibitor. These findings hold promise for the development of effective allosteric Hsp90 CTD inhibitors as potential breast cancer therapeutics.

## Experimental Section

### Chemistry

Reagents and solvents used for synthesis were purchased from Fluorochem Ltd. (Derbyshire, UK), TCI (Tokyo, Japan), Apollo Scientific Ltd. (Stockport, UK), and Sigma-Aldrich (St. Louis, MO, USA). Reagents were used without further purification. Silica gel on aluminum sheets was used for analytical thin layer chromatography (0.20 mm; 60 F254; Merck, Darmstadt, Germany). Column chromatography was performed using silica gel 60 (particle size, 230–400 mesh). ^1^H, ^13^C, and 2D NMR spectra were recorded on a Bruker AVANCE III 400 MHz NMR spectrometer (Bruker Corporation, Billerica, MA, USA). The splitting patterns were designated as follows: s, singlet; d, doublet; dd, double doublet; m, multiplet. Purity of compounds was determined using HPLC-MS on a 1260 Infinity II LC system (Agilent Technologies, Santa Clara, CA, USA), coupled to mass spectrometry (Expression CMS^L^; Advion Inc., Ithaca, NY, USA). The column used was Waters XBridge C_18_ column (3.5 μm, 4.6 mm × 150 mm), the flow rate was 1.5 mL/min, and sample injection volume was 10 μL. Compounds were detected with a UV detector at 254 nm. The mobile phase consisted of 0.1% HCOOH in double-distilled H_2_O (solvent A) and acetonitrile (solvent B). The gradient for solvent B was 0 → 1 min, 25%; 1 → 6 min, 25 → 98%; 6 → 6.5 min, 98%; 6.5 → 7 min, 98 → 25%; 7 → 10 min, 25%. Mass spectra and high-resolution mass spectra (HRMS) were obtained on Expression CMS^L^ (Advion Inc., Ithaca, NY, USA) and (Exactive Plus Orbitrap mass spectrometer; Thermo Scientific Inc., Waltham, MA, USA), respectively. The microwave-assisted reactions were performed using an Anton Paar Monowave 200 microwave reactor (Anton Paar GmbH, Graz, Austria).

All compounds used for biological assays were >95% pure, as determined by HPLC analysis (Figures S86–S122), with the exception of compounds **49**, **52,** and **112**. These three compounds were not purified further due to their poor activity in the MTS assay.

### General Synthetic Procedures

#### General Procedure A: EDC/HOBt-Mediated Coupling

The corresponding carboxylic acid (1 mmol), 1-ethyl-3-(3-(dimethylamino)propyl)carbodiimide (1.2 mmol), and 1-hydroxybenzotriazole (1.3 mmol) were dissolved in *N*,*N*-dimethylformamide (DMF) (10 mL). The mixture was stirred at 0 °C for 20 min. Then, *N*-methylmorpholine (NMM) (2 mmol) and the corresponding amine (1 mmol) were added. The mixture was stirred at room temperature for 18 h. The solvent was removed in vacuo, and the residue was dissolved in ethyl acetate and successively washed with 1 M aqueous solution of NaOH (2 × 10 mL), 1 M aqueous solution of HCl (2 × 10 mL), and brine (10 mL), dried over anhydrous Na_2_SO_4_, and filtered. The solvent was removed in vacuo.

#### General Procedure B: Reductive Amination

The corresponding amine (1 mmol) and aldehyde (1 mmol) were dissolved in methanol, and then acetic acid (1 mmol) was added. The solution was stirred at 20 °C for 2 h, then NaCNBH_3_ (1.2 mmol) was added, and the mixture was further stirred at 20 °C. Reaction was monitored by TLC, and upon completion, the solvent was removed in vacuo.

#### General Procedure C: Removal of the Boc Protecting Group

The Boc-protected compound (1 mmol) was dissolved in dichloromethane (DCM) (10 mL), and then trifluoroacetic acid (10 mmol) was added. The mixture was stirred at 20 °C for 18 h or until reaction was completed. Upon completion, pH of the reaction mixture was adjusted with 2 M NaOH to pH = 14. Phases were separated, and the aqueous phase was extracted with dichloromethane (3 × 10 mL). Organic phases were combined, dried over anhydrous Na_2_SO_4_, and filtered. The solvent was removed in vacuo.

Removal of the Boc protective group to obtain the final compounds **45**–**56** was accomplished using 1 M HCl in 1,4-dioxane. Boc-protected compounds (1 mmol) were dissolved in 1,4-dioxane, 1 M HCl in 1,4-dioxane (10 mmol) was added, and the mixture was stirred at 20 °C for 18 h. Solvent and HCl were removed in vacuo.

#### General Procedure D: Ester Hydrolysis

To a solution of the corresponding ester (1 mmol) in methanol (5 mL), 2 M NaOH (5 mmol) was added. The mixture was stirred at 50 °C for 1 day. Solvent was removed in vacuo, residue was dissolved in DCM, and pH was adjusted with 1 M HCl to pH = 1. Phases were separated, and the aqueous phase was extracted with DCM (3 × 10 mL). Organic phases were combined, dried over Na_2_SO_4_, and filtered. The solvent was removed in vacuo.

#### General Procedure E

To a solution of the starting compound (compound **1**, 4-hydroxybenzaldehyide or methyl-4-hydroxybenzoate) in DMF or acetonitrile (5 mL), the corresponding phenol (1.1 mmol) or alkyl halide (1.1 mmol) and K_2_CO_3_ (2 mmol) were added. The mixture was stirred at 80 °C for 24 h. The reaction mixture was concentrated in vacuo. Residue was dissolved in ethyl acetate and washed successively with 1% citric acid (2 × 5 mL), 1 M NaOH (2 × 5 mL), and brine. The organic phase was dried over Na_2_SO_4_ and filtered. The solvent was removed in vacuo.

##### Synthesis of *tert*-Butyl 1-Oxa-6-azaspiro[2.5]octane-6-carboxylate (**1**)

A solution of *tert*-butyl 4-methylenepiperidine-1-carboxylate (20.6 mmol, 2.00 g) in chloroform (40 mL) was cooled to 0 °C. Then *meta*-chloroperoxybenzoic acid (30.9 mmol, 2.62 g) was added. The mixture was stirred at 0 °C for 30 min and then at 20 °C for 18 h. The organic phase was washed with 10% aqueous Na_2_SO_3_ solution (2 × 10 mL) and saturated aqueous NaHCO_3_ solution (2 × 10 mL). The organic phase was dried over Na_2_SO_4_, filtered, and concentrated under reduced pressure. The residue was purified using flash column chromatography using ethyl acetate/hexane (1/4) as eluent. Yield: 75.1%; white solid; ^1^H NMR (400 MHz, chloroform-*d*): δ 3.72 (d, *J* = 12.8 Hz, 2H, CH_2_–piperidine), 3.43 (ddd, *J*_1_ = 13.2 Hz, *J*_2_ = 9.5 Hz, *J*_3_ = 3.7 Hz, 2H, CH_2_–piperidine), 2.69 (s, 2H, CH_2_), 1.80 (ddd, *J*_1_ = 13.8 Hz, *J*_2_ = 9.5 Hz, *J*_3_ = 4.5 Hz, 2H, CH_2_–piperidine), 1.47 (s, 11H, CH_2_–piperidine, 3 × CH_3_); ^13^C NMR (101 MHz, chloroform-*d*): δ 154.6, 79.6, 57.0, 53.6, 42.4, 32.9, 28.3; MS (ESI^+^) *m*/*z*: 236.3 ([M + Na]^+^).

##### Synthesis of *tert*-Butyl 4-Hydroxy-4-((*m*-tolyloxy)methyl)piperidine-1-carboxylate (**2**)

It was synthesized according to general procedure E using compound **1** (2.1 mmol, 500 mg) and *m*-cresol (2.3 mmol, 250 mg) as reagents. The crude product was purified using flash column chromatography using ethyl acetate/hexane as eluent (1:4). Yield: 52.1%; colorless oil; ^1^H NMR (400 MHz, chloroform-*d*): δ 7.17 (t, *J* = 7.6 Hz, 1H, Ar–H), 6.80 (ddt, *J*_1_ = 7.6 Hz, *J*_2_ = 1.5 Hz, *J*_3_ = 0.8 Hz, 1H, Ar–H), 6.74–6.68 (m, 2H, 2 × Ar–H), 3.90 (s, 2H, CH_2_–piperidine), 3.79 (s, 2H, CH_2_), 3.23 (t, *J* = 12.4 Hz, 2H, CH_2_–piperidine), 2.36–2.31 (m, 3H, CH_3_), 1.74 (d, *J* = 13.5 Hz, 2H, CH_2_–piperidine), 1.66–1.57 (m, 2H, CH_2_–piperidine), 1.47 (s, 9H, 3 × CH_3_), signal for OH not seen in the spectrum; MS (ESI^+^) *m*/*z*: 322.2 ([M + H]^+^).

##### Synthesis of *tert*-Butyl 4-((3,4-Dichlorophenoxy)methyl)-4-hydroxypiperidine-1-carboxylate (**3**)

It was synthesized according to general procedure E using compound **1** (5.61 mmol, 1.30 g) and 3,4-dichlorophenol (6.18 mmol, 1.00 g) as reagents. The crude product was purified using flash column chromatography using ethyl acetate/hexane as eluent (1:4). Yield: 66.9%; colorless oil; ^1^H NMR (400 MHz, chloroform-*d*): δ 7.33 (d, *J* = 8.9 Hz, 1H, Ar–H), 7.01 (d, *J* = 2.9 Hz, 1H, Ar–H), 6.77 (dd, *J*_1_ = 8.9 Hz, *J*_2_ = 2.9 Hz, 1H, Ar–H), 3.92 (s, 2H, CH_2_–piperidine), 3.78 (s, 2H, CH_2_), 3.21 (t, *J* = 12.6 Hz, 2H, CH_2_–piperidine), 2.15 (s, 1H, CH), 1.77–1.67 (m, 2H, CH_2_–piperidine), 1.60 (td, *J*_1_ = 13.3 Hz, *J*_2_ = 12.8 Hz, *J*_3_ = 4.8 Hz, 2H, CH_2_–piperidine), 1.47 (s, 9H, 3 × CH_3_), signal for OH not seen in the spectrum; ^13^C NMR (101 MHz, chloroform-*d*): δ 157.6, 154.8, 132.9, 130.7, 124.5, 116.5, 114.5, 79.6, 76.1, 69.1, 33.6, 28.5; MS (ESI+) *m*/*z*: 360.6 ([M + H]^+^).

##### Synthesis of *tert*-Butyl 4-((3-Chloro-4-fluorophenoxy)methyl)-4-hydroxypiperidine-1-carboxylate (**4**)

It was synthesized according to general procedure E using compound **1** (1.11 mmol, 236 mg) and 3-chloro-4-fluorophenol (1.24 mmol, 182 mg) as reagents. Yield: 88.7%; purple oil; ^1^H NMR (400 MHz, chloroform-*d*): δ 7.08 (t, *J* = 5.9 Hz, 1H, Ar–H), 6.97 (dd, *J*_1_ = 5.9 Hz, *J*_2_ = 3.0 Hz, 1H, Ar–H), 6.82–6.75 (m, 1H, Ar–H), 3.96 (s, 2H, CH_2_–piperidine), 3.78 (s, 2H, CH_2_), 3.23 (t, *J* = 3.0 Hz, 2H, CH_2_–piperidine), 2.10 (s, 1H, OH), 1.74 (d, *J* = 12.3 Hz, 2H, CH_2_–piperidine), 1.64 (dd, *J*_1_ = 12.3 Hz, *J*_2_ = 4.5 Hz, 2H, CH_2_–piperidine), 1.49 (s, 9H, 3 × CH_3_); MS (ESI^+^) *m*/*z*: 360.5 ([M + H]^+^).

##### Synthesis of *tert*-Butyl 4-((3-Chlorophenoxy)methyl)-4-hydroxypiperidine-1-carboxylate (**5**)

It was synthesized according to general procedure E using compound **1** (1.1 mmol, 234 mg) and 3-chlorophenol (1.22 mmol, 188 mg) as reagents. The crude product was purified using flash column chromatography using ethyl acetate/hexane (1:4) as eluent. Yield: 40.8%; white crystals; ^1^H NMR (400 MHz, chloroform-*d*): δ 7.21 (t, *J* = 8.2 Hz, 1H, Ar–H), 6.97 (ddd, *J*_1_ = 8.2 Hz, *J*_2_ = 1.9 Hz, *J*_3_ = 0.8 Hz, 1H, Ar–H), 6.91 (t, *J* = 2.2 Hz, 1H, Ar–H), 6.80 (ddd, *J*_1_ = 8.2 Hz, *J*_2_ = 2.5 Hz, *J*_3_ = 0.8 Hz, 1H, Ar–H), 3.93 (s, 2H, CH_2_–piperidine), 3.80 (s, 2H, CH_2_), 3.23 (t, *J* = 12.5 Hz, 2H, CH_2_–piperidine), 2.11 (s, 1H, OH), 1.74 (d, *J* = 13.5 Hz, 2H, CH_2_–piperidine), 1.60 (d, *J* = 5.2 Hz, 2H, CH_2_–piperidine), 1.47 (s, 9H, 3 × CH_3_); MS (ESI^+^) *m*/*z*: 342.4 ([M + H]^+^).

##### Synthesis of *tert*-Butyl 4-((3-Chloro-4-cyanophenoxy)methyl)-4-hydroxypiperidine-1-carboxylate (**6**)

It was synthesized according to general procedure E using compound **1** (1.11 mmol, 237 mg) and 2-chloro-4-hydroxybenzonitrile (1.22 mmol, 188 mg) as reagents. The crude product was purified using flash column chromatography using ethyl acetate/hexane (1:2) as eluent. Yield: 33.9%, white crystals; ^1^H NMR (400 MHz, chloroform-*d*): δ 7.60 (d, *J* = 8.7 Hz, 1H, Ar–H), 7.04 (d, *J* = 2.4 Hz, 1H, Ar–H), 6.89 (dd, *J*_1_ = 8.7 Hz, *J*_2_ = 2.5 Hz, 1H, Ar–H), 3.86 (s, 4H, 2 × CH_2_), 3.20 (t, *J* = 11.6 Hz, 2H, CH_2_–piperidine), 1.98 (s, 1H, OH), 1.64 (dd, *J* = 3.7 Hz, 2H, CH_2_–piperidine), 1.73 (d, *J* = 12.6 Hz, 2H, CH_2_–piperidine), 1.47 (s, 9H, 3 × CH_3_); MS (ESI^+^) *m*/*z*: 389.4 ([M + Na]^+^).

##### Synthesis of *tert*-Butyl 4-((4-Chlorophenoxy)methyl)-4-hydroxypiperidine-1-carboxylate (**7**)

It was synthesized according to general procedure E using compound **1** (2.35 mmol, 500 mg) and 4-chlorophenol (2.58 mmol, 330 mg) as reagents. The crude product was purified using flash column chromatography using ethyl acetate/hexane (1:3) as eluent. Yield: 52.0%; yellow oil; ^1^H NMR (400 MHz, chloroform-*d*): δ 7.26–7.22 (m, 2H, 2 × Ar–H), 6.86–6.81 (m, 2H, 2 × Ar–H), 3.91 (s, 2H, CH_2_–piperidine), 3.77 (s, 2H, CH_2_), 3.22 (t, *J* = 12.6 Hz, 2H, CH_2_–piperidine), 2.17 (s, 1H, OH), 1.73 (dt, *J*_1_ = 14.2 Hz, *J*_2_ = 2.0 Hz, 2H, CH_2_–piperidine), 1.64–1.57 (m, 2H, CH_2_–piperidine), 1.43 (s, 9H, 3 × CH_3_); MS (ESI^+^) *m*/*z*: 242.0 ([M + Na-Boc]^+^).

##### Synthesis of *tert*-Butyl 4-((4-Bromo-3-chlorophenoxy)methyl)-4-hydroxypiperidine-1-carboxylate (**8**)

It was synthesized according to general procedure E using compound **1** (2.8 mmol, 600 mg) and 4-bromo-3-chlorophenol (3.1 mmol, 427 mg) as reagents. The crude product was purified using flash column chromatography using dichloromethane/methanol (60:1) as eluent. Yield: 33.8%; red oil; ^1^H NMR (400 MHz, chloroform-*d*):: δ 7.49 (d, *J* = 8.9 Hz, 1H, Ar–H), 7.03 (d, *J* = 2.9 Hz, 1H, Ar–H), 6.71 (dd, *J*_1_ = 8.9 Hz, *J*_2_ = 2.9 Hz, 1H, Ar–H), 3.92 (s, 2H, CH_2_–piperidine), 3.78 (s, 2H, CH_2_), 3.22 (d, *J* = 11.2 Hz, 2H, CH_2_–piperidine), 1.72 (d, *J* = 12.3 Hz, 2H, CH_2_–piperidine), 1.62 (dd, *J*_1_ = 12.1 Hz, *J*_2_ = 4.7 Hz, 2H, CH_2_–piperidine), 1.47 (s, 9H, 3 × CH_3_), signal for OH not seen in the spectrum; MS (ESI^+^) *m*/*z*: 420.7 ([M + H]^+^).

##### Synthesis of *tert*-Butyl 4-((3-Fluoro-4-chlorophenoxy)methyl)-4-hydroxypiperidine-1-carboxylate (**9**)

It was synthesized according to general procedure E using compound **1** (2.8 mmol, 600 mg) and 3-fluoro-4-chlorophenol (4.5 mmol, 660 mg) as reagents. The crude product was purified using flash column chromatography using ethyl acetate/hexane (1:4) as eluent. Yield: 88.9%; yellow oil; ^1^H NMR (400 MHz, chloroform-*d*): δ 7.29 (d, *J* = 8.8 Hz, 1H, Ar–H), 6.72 (dd, *J*_1_ = 10.6 Hz, *J*_2_ = 2.8 Hz, 1H, Ar–H), 6.66 (ddd, *J*_1_ = 8.8 Hz, *J*_2_ = 2.8 Hz, *J*_3_ = 1.2 Hz, 1H, Ar–H), 3.92 (s, 2H, CH_2_–piperidine), 3.77 (s, 2H, CH_2_), 3.21 (t, *J* = 12.9 Hz, 2H, CH_2_–piperidine), 1.72 (d, *J* = 13.3 Hz, 2H, CH_2_–piperidine), 1.62 (dd, *J*_1_ = 12.5 Hz, *J*_2_ = 4.8 Hz, 2H, CH_2_–piperidine), 1.47 (s, 9H, 3 × CH_3_); MS (ESI^+^) *m*/*z*: 360.6 ([M + H]^+^).

##### Synthesis of *tert*-Butyl 4-((3-Bromo-4-chlorophenoxy)methyl)-4-hydroxypiperidine-1-carboxylate (**10**)

It was synthesized according to general procedure E using compound **1** (2.8 mmol, 600 mg) and 3-bromo-4-chlorophenol (4.5 mmol, 934 mg) as reagents. The crude product was purified using flash column chromatography using ethyl acetate/hexane (1:4) as eluent. Yield: 40.5%; red oil; ^1^H NMR (400 MHz, chloroform-*d*): δ 7.34 (d, *J* = 8.8 Hz, 1H, Ar–H), 7.18 (d, *J* = 2.9 Hz, 1H, Ar–H), 6.82 (dd, *J*_1_ = 8.9 Hz, *J*_2_ = 2.9 Hz, 1H, Ar–H), 3.92 (s, 2H, CH_2_–piperidine), 3.78 (s, 2H, CH_2_), 3.21 (t, *J* = 12.6 Hz, 2H, CH_2_–piperidine), 1.72 (d, *J* = 13.3 Hz, 2H, CH_2_–piperidine), 1.62 (dd, *J*_1_ = 12.4 Hz, *J*_2_ = 4.9 Hz, 2H, CH_2_–piperidine), 1.47 (s, 9H, 3 × CH_3_); MS (ESI^+^) *m*/*z*: 420.1 ([M + H]^+^).

##### Synthesis of *tert*-Butyl 4-((3-Iodo-4-chlorophenoxy)methyl)-4-hydroxypiperidine-1-carboxylate (**11**)

It was synthesized according to general procedure E using compound **1** (3.3 mmol, 700 mg) and 3-iodo-4-chlorophenol (5.5 mmol, 1.34 g) as reagents. The crude product was purified using flash column chromatography using ethyl acetate/hexane (1:3) as eluent. Yield: 46.2%; yellow oil; ^1^H NMR (400 MHz, chloroform-*d*): δ 7.39 (d, *J* = 2.9 Hz, 1H, Ar–H), 7.33 (d, *J* = 8.8 Hz, 1H, Ar–H), 6.85 (dd, *J*_1_ = 8.8 Hz, *J*_2_ = 2.9 Hz, 1H, Ar–H), 3.91 (s, 2H, CH_2_–piperidine), 3.76 (s, 2H, CH_2_), 3.20 (t, *J* = 13.0 Hz, 2H, CH_2_–piperidine), 1.72 (d, *J* = 13.2 Hz, 2H, CH_2_–piperidine), 1.63–1.55 (m, 2H, CH_2_–piperidine), 1.47 (s, 9H, 3 × CH_3_). MS (ESI^+^) *m*/*z*: 368.0 ([M + H-Boc]^+^).

##### Synthesis of 4-((*m*-Tolyloxy)methyl)piperidin-4-ol (**12**)

It was synthesized according to general procedure C from compound **2** (1.25 mmol, 400 mg). Yield: 87.2%; ^1^H NMR (400 MHz, chloroform-*d*): δ 7.17 (t, *J* = 7.8 Hz, 1H, Ar–H), 6.79 (ddt, *J*_1_ = 7.5 Hz, *J*_2_ = 1.7 Hz, *J*_3_ = 0.9 Hz, 1H, Ar–H), 6.78–6.68 (m, 2H, 2 × Ar–H), 3.80 (s, 2H, CH_2_), 3.06 (ddd, *J*_1_ = 12.2 Hz, *J*_2_ = 10.7 Hz, *J*_3_ = 3.4 Hz, 2H, CH_2_–piperidine), 2.33 (d, *J* = 0.8 Hz, 3H, CH_3_), 2.00 (s, 2H, CH_2_–piperidine), 1.78–1.61 (m, 4H, 2 × CH_2_–piperidine); MS (ESI^+^) *m*/*z*: 322.2 ([M + H]^+^).

##### Synthesis of 4-((3,4-Dichlorophenoxy)methyl)piperidin-4-ol (**13**)

It was synthesized according to general procedure C using compound **3** (3.89 mmol, 1.41 g) as reagent. Yield: 77.3%; colorless oil; ^1^H NMR (400 MHz, chloroform-*d*): δ 7.33 (d, *J* = 8.8 Hz, 1H, Ar–H), 7.02 (d, *J* = 2.9 Hz, 1H, Ar–H), 6.78 (dd, *J*_1_ = 8.8 Hz, *J*_2_ = 2.9 Hz, 1H, Ar–H), 3.78 (s, 2H, CH_2_), 3.03 (ddd, *J*_1_ = 12.2 Hz, *J*_2_ = 10.6 Hz, *J*_3_ = 3.5 Hz, 2H, CH_2_–piperidine), 2.89 (dt, *J*_1_ = 12.4 Hz, *J*_2_ = 4.2 Hz, 2H, CH_2_–piperidine), 1.73–1.64 (m, 4H, 2 × CH_2_–piperidine), signals for NH an OH not seen in the spectrum; ^13^C NMR (101 MHz, DMSO-*d*_6_): δ 159.0, 132.0, 131.3, 122.7, 116.9, 116.1, 76.9, 68.7, 41.8, 34.8; MS (ESI^+^) *m*/*z*: 275.9 ([M + H]^+^).

##### Synthesis of 4-((3-Chloro-4-fluorophenoxy)methyl)piperidin-4-ol (**14**)

It was synthesized according to general procedure C using compound **4** (0.89 mmol, 317 mg) as reagent. Yield: 79.6%; orange oil; ^1^H NMR (400 MHz, DMSO-*d*_6_):: δ 7.32 (t, *J* = 9.1 Hz, 1H, Ar–H), 7.16 (dd, *J*_1_ = 6.1 Hz, *J*_2_ = 3.0 Hz, 1H, Ar–H), 6.94 (ddd, *J*_1_ = 9.1 Hz, *J*_2_ = 3.8 Hz, *J*_3_ = 3.1 Hz, 1H, Ar–H), 4.49 (s, 1H, OH), 3.73 (s, 2H, CH_2_), 2.89–2.60 (m, 4H, 2 × CH_2_–piperidine), 1.60–1.38 (m, 4H, 2 × CH_2_–piperidine); signal for NH not seen in the spectrum; MS (ESI^+^) *m*/*z*: 260.2 ([M + H]^+^).

##### Synthesis of 4-((3-Chlorophenoxy)methyl)piperidin-4-ol (**15**)

It was synthesized according to general procedure C using compound **5** (0.42 mmol, 142 mg) as reagent. Yield: 87.6%; off white solid; ^1^H NMR (400 MHz, chloroform-*d*): δ 7.21 (t, *J* = 8.1 Hz, 1H, Ar–H), 6.98–6.91 (m, 2H, 2 × Ar–H), 6.81 (dd, *J*_1_ = 8.4 Hz, *J*_2_ = 2.5 Hz, 1H, Ar–H), 3.80 (s, 2H, CH_2_), 3.05 (ddd, *J*_1_ = 12.1 Hz, *J*_2_ = 10.7 Hz, *J*_3_ = 3.5 Hz, 2H, CH_2_–piperidine), 2.91 (dt, *J*_1_ = 12.3 Hz, *J*_2_ = 4.1 Hz, 2H, CH_2_–piperidine), 1.76–1.62 (m, 4H, 2 × CH_2_–piperidine), signals for OH and NH not seen in the spectrum; MS (ESI^+^) *m*/*z*: 342.4 ([M + H]^+^).

##### Synthesis of 2-Chloro-4-((4-hydroxypiperidin-4-yl)methoxy)benzonitrile (**16**)

It was synthesized according to general procedure C using compound **6** (0.33 mmol, 120 mg) as reagent. Yield: 57.3%; white solid; ^1^H NMR (400 MHz, chloroform-*d*): δ 7.59 (d, *J* = 8.7 Hz, 1H, Ar–H), 7.05 (d, *J* = 2.4 Hz, 1H, Ar–H), 6.89 (dd, *J*_1_ = 8.7 Hz, *J*_2_ = 2.4 Hz, 1H, Ar–H), 3.86 (s, 2H, CH_2_), 3.04 (td, *J*_1_ = 11.6 Hz, *J*_2_ = 11.1 Hz, *J*_3_ = 3.7 Hz, 2H, CH_2_–piperidine), 2.92 (dt, *J*_1_ = 12.1 Hz, *J*_2_ = 3.9 Hz, 2H, CH_2_–piperidine), 1.94 (s, 1H, OH), 1.71 (d, *J* = 4.6 Hz, 4H, 2 × CH_2_–piperidine), signal NH not seen in the spectrum; MS (ESI^+^) *m*/*z*: 267.2 ([M + H]^+^).

##### Synthesis of 4-((4-Chlorophenoxy)methyl)piperidin-4-ol (**17**)

It was synthesized according to general procedure C using compound **7** (1.29 mmol, 411 mg) as reagent. Yield: 80.6%; yellow solid; ^1^H NMR (400 MHz, chloroform-*d*): δ 7.24 (d, *J* = 9.0 Hz, 2H, 2 × Ar–H), 6.84 (d, *J* = 9.0 Hz, 2H, 2 × Ar–H), 3.78 (s, 2H, CH_2_), 3.04 (ddd, *J*_1_ = 12.2 Hz, *J*_2_ = 10.7 Hz, *J*_3_ = 3.4 Hz, 2H, CH_2_–piperidine), 2.89 (dt, *J*_1_ = 12.4 Hz, *J*_2_ = 4.2 Hz, 2H, CH_2_–piperidine), 1.77–1.70 (m, 2H, CH_2_–piperidine), 1.66 (td, *J*_1_ = 10.0 Hz, *J*_2_ = 9.2 Hz, *J*_3_ = 5.3 Hz, 2H, CH_2_–piperidine), signals for NH an OH not seen in the spectrum; MS (ESI+) *m*/*z*: 242.0 ([M + H]^+^).

##### Synthesis of 4-((4-Bromo-3-chlorophenoxy)methyl)piperidin-4-ol (**18**)

It was synthesized according to general procedure C using compound **8** (0.95 mmol, 400 mg) as reagent. Yield: 98.4%; white solid. Product was used in the next step without further purification. MS (ESI^+^) *m*/*z*: 320.0 ([M + H]^+^).

##### Synthesis of 4-((4-Chloro-3-fluorophenoxy)methyl)piperidin-4-ol (**19**)

It was synthesized according to general procedure C using compound **9** (2.52 mmol, 900 mg) as reagent. Yield: 81.6%; white solid; ^1^H NMR (400 MHz, chloroform-*d*): δ 7.29 (d, *J* = 8.7 Hz, 1H, Ar–H), 6.73 (dd, *J*_1_ = 10.7 Hz, *J*_2_ = 2.8 Hz, 1H, Ar–H), 6.66 (ddd, *J*_1_ = 8.9 Hz, *J*_2_ = 2.8 Hz, *J*_3_ = 1.2 Hz, 1H, Ar–H), 3.78 (s, 2H, CH_2_), 3.08–2.99 (m, 2H, CH_2_–piperidine), 2.90 (m, *J*_1_ = 12.3 Hz, *J*_2_ = 4.1 Hz, 2H, CH_2_–piperidine), 1.76–1.63 (m, 4H, 2 × CH_2_–piperidine), signals for OH and NH not seen in the spectrum; MS (ESI^+^) *m*/*z*: 260.4 ([M + H]^+^).

##### Synthesis of 4-((3-Bromo-4-chlorophenoxy)methyl)piperidin-4-ol (**20**)

It was synthesized according to general procedure C using compound **10** (1.14 mmol, 480 mg) as reagent. Yield: 82.6%; white solid; ^1^H NMR (400 MHz, chloroform-*d*): δ 7.34 (d, *J* = 8.9 Hz, 1H, Ar–H), 7.19 (d, *J* = 2.9 Hz, 1H, Ar–H), 6.82 (dd, *J*_1_ = 8.9 Hz, *J*_2_ = 2.9 Hz, 1H, Ar–H), 3.78 (s, 2H, CH_2_), 3.03 (t, *J* = 11.7 Hz, 2H, CH_2_–piperidine), 2.89 (d, *J* = 11.9 Hz, 2H, CH_2_–piperidine), 1.75–1.68 (m, 2H, CH_2_–piperidine), 1.66 (dd, *J*_1_ = 10.6 Hz, *J*_2_ = 4.4 Hz, 2H, CH_2_–piperidine), signals for OH and NH not seen in the spectrum; MS (ESI^+^) *m*/*z*: 320.4 ([M + H]^+^).

##### Synthesis of 4-((4-Chloro-3-iodophenoxy)methyl)piperidin-4-ol (**21**)

It was synthesized according to general procedure C using compound **11** (1.52 mmol, 710 mg) as reagent. Yield: 86.0%; white solid; ^1^H NMR (400 MHz, chloroform-*d*): δ 7.40 (d, *J* = 2.9 Hz, 1H, Ar–H), 7.33 (d, *J* = 8.8 Hz, 1H, Ar–H), 6.86 (dd, *J*_1_ = 8.1 Hz, *J*_2_ = 2.9 Hz, 1H, Ar–H), 3.77 (s, 2H, CH_2_), 3.04 (ddd, *J*_1_ = 12.2 Hz, *J*_2_ = 10.6 Hz, *J*_3_ = 3,6 Hz, 2H, CH_2_–piperidine), 2.91 (dt, *J*_1_ = 12.0 Hz, *J*_2_ = 4.0 Hz, 2H, CH_2_–piperidine), 1.75–1.64 (m, 4H, 2 × CH_2_–piperidine), signals for OH and NH not seen in the spectrum; MS (ESI^+^) *m*/*z*: 368.0 ([M + H]^+^).

##### Synthesis of *tert*-Butyl 4-((4-Formylphenoxy)methyl)piperidine-1-carboxylate (**24**)

It was synthesized according to general procedure E using 4-hydroxybenzaldehide (8.19 mmol, 1.00 g) and *tert*-butyl 4-(bromomethyl)piperidine-1-carboxylate (8.19 mmol, 2.27 g) as reagents. The crude product was purified using flash column chromatography using ethyl acetate/hexane as eluent (1:9). Yield: 67.8%; colorless oil; ^1^H NMR (400 MHz, chloroform-*d*): δ 9.89 (s, 1H, CHO), 7.89–7.81 (m, 2H, 2 × Ar–H), 7.02–6.96 (m, 2H, 2 × Ar–H), 4.17 (s, 2H, CH_2_–piperidine), 3.89 (d, *J* = 6.4 Hz, 2H, CH_2_), 2.76 (t, *J* = 12.9 Hz, 2H, CH_2_–piperidine), 1.99 (td, *J*_1_ = 8.1 Hz, *J*_2_ = 7.2 Hz, *J*_3_ = 4.2 Hz, 1H, CH–piperidine), 1.83 (d, *J* = 12.7 Hz, 2H, CH_2_–piperidine), 1.47 (s, 9H, 3 × CH_3_), 1.29 (qd, *J*_1_ = 12.7 Hz, *J*_2_ = 4.5 Hz, 2H, CH_2_–piperidine); ^13^C NMR (101 MHz, chloroform-*d*): δ 190.7, 164.0, 154.8, 145.3, 132.0, 129.9, 114.7, 109.1, 79.5, 72.6, 36.1, 28.5; MS (ESI^+^) *m*/*z*: 320.0 ([M + H]^+^).

##### Synthesis of *tert*-Butyl 4-((4-(Methoxycarbonyl)phenoxy)methyl)piperidine-1-carboxylate (**25**)

It was synthesized according to general procedure E using methyl 4-hydroxy benzoate (32.9 mmol, 5.00 g) and *tert*-butyl 4-(bromomethyl)piperidine-1-carboxylate (32.9 mmol, 9.14 g) as reagents. The crude product was purified using flash column chromatography using dichloromethane/methanol as eluent (60:1). Yield: 63.2%; white solid. ^1^H NMR (400 MHz, chloroform-*d*): δ 8.01–7.95 (m, 2H, 2 × Ar–H), 6.92–6.87 (m, 2H, 2 × Ar–H), 4.16 (s, 2H, CH_2_–piperidine), 3.88 (s, 3H, O–CH_3_), 3.85 (d, *J* = 6.4 Hz, 2H, CH_2_), 2.75 (t, *J* = 12.9 Hz, 2H, CH_2_–piperidine), 1.97 (td, *J*_1_ = 8.0 Hz, *J*_2_ = 7.2 Hz, *J*_3_ = 4.1 Hz, 1H, CH–piperidine), 1.82 (d, *J* = 12.9 Hz, 2H, CH_2_–piperidine), 1.47 (s, 9H, 3 × CH_3_), 1.28 (qd, *J*_1_ = 12.3, *J*_2_ = 4.4 Hz, 2H, CH_2_–piperidine); MS (ESI^+^) *m*/*z*: 350.3 ([M + H]^+^).

##### Synthesis of 4-((1-(*tert*-Butoxycarbonyl)piperidin-4-yl)methoxy)benzoic Acid (**26**)

It was synthesized according to general procedure D, using compound **25** (5.73 mmol, 2.00 g) as reagent. Yield: 79.3%; white solid; ^1^H NMR (400 MHz, DMSO-*d*_6_): δ 7.87 (d, *J* = 8.8 Hz, 2H, 2 × Ar–H), 7.01 (d, *J* = 8.9 Hz, 2H, 2 × Ar–H), 3.97 (d, *J* = 13.2 Hz, 2H, CH_2_–piperidine), 3.91 (d, *J* = 6.4 Hz, 2H, CH_2_), 2.74 (s, 2H, CH_2_–piperidine), 1.99–1.89 (m, 1H, CH–piperidine), 1.75 (dd, *J*_1_ = 13.5 Hz, *J*_2_ = 3.5 Hz, 2H, CH_2_–piperidine), 1.40 (s, 9H, 3 × CH_3_), 1.16 (tt, *J*_1_ = 12.4 Hz, *J*_2_ = 6.3 Hz, 2H, CH_2_–piperidine), signal for COOH not seen in the spectrum; ^13^C NMR (101 MHz, chloroform-*d*): δ 171.6, 163.4, 154.9, 132.3, 121.7, 114.2, 79.6, 72.5, 36.1, 28.8, 28.5; MS (ESI^–^) *m*/*z*: 334.0 ([M – H]^−^).

##### Synthesis of *tert*-Butyl 4-(4-Formylphenoxy)piperidine-1-carboxylate (**27**)

A solution of 4-hydroxybenzaldehyde (8.19 mmol, 1.00 g), *tert*-butyl 4-hydroxypiperidine-1-carboxylate (8.19 mmol, 1.65 g), and PPh_3_ (10.65 mmol, 2.81 g) in anhydrous THF was cooled to 0 °C and DIAD was added dropwise (2.1 mL, 10.65 mmol). The reaction mixture was stirred under an argon atmosphere at 0 °C for 18 h. The solvent was removed in vacuo. The crude product was purified using flash column chromatography using ethyl acetate/hexane (1:4) as eluent. Yield: 18.6%; white solid; ^1^H NMR (400 MHz, chloroform-*d*): δ 9.88 (s, 1H, CHO), 7.83 (d, *J* = 8.7 Hz, 2H, 2 × Ar–H), 7.00 (d, *J* = 8.7 Hz, 2H, 2 × Ar–H), 4.63–4.55 (m, 1H, CH–piperidine), 3.75–3.64 (m, 2H, CH_2_–piperidine), 3.42–3.32 (m, 2H, CH_2_–piperidine), 2.01–1.90 (m, 2H, CH_2_–piperidine), 1.83–1.73 (m, 2H, CH_2_–piperidine), 1.47 (s, 9H, 3 × CH_3_); MS (ESI^+^) *m*/*z*: 328.2 ([M + Na]^+^).

##### Synthesis of *tert*-Butyl 4-(4-(Methoxycarbonyl)phenoxy)piperidine-1-carboxylate (**28**)

A solution of methyl 4-hydroxybenzoate (6.57 mmol, 1.00 g), *tert*-butyl 4-hydroxypiperidine-1-carboxylate (6.57 mmol, 1.321 g), and PPh_3_ (8.65 mmol, 2.26 g) in anhydrous THF was cooled to 0 °C, and DIAD was added dropwise (1.45 mL, 8.55 mmol). The reaction mixture was stirred under an argon atmosphere at 0 °C for 18 h. Solvent was removed in vacuo. The crude product was purified using flash column chromatography using dichloromethane as eluent. Yield: 54.4%; white solid; ^1^H NMR (400 MHz, chloroform-*d*): δ 8.04–7.95 (m, 2H, 2 × Ar–H), 6.96–6.86 (m, 2H, 2 × Ar–H), 4.62–4.52 (m, 1H, CH–piperidine), 3.89 (s, 3H, CH_3_), 3.77–3.64 (m, 2H, CH_2_–piperidine), 3.42–3.31 (m, 2H, CH_2_–piperidine), 2.01–1.88 (m, 2H, CH_2_–piperidine), 1.82–1.72 (m, 2H, CH_2_–piperidine), 1.47 (s, 9H, 3 × CH_3_); MS (ESI^+^) *m*/*z*: 358.6 ([M + Na]^+^).

##### Synthesis of 4-((1-(*tert*-Butoxycarbonyl)piperidin-4-yl)oxy)benzoic Acid (**29**)

It was synthesized according to general procedure D, using compound **28** (0.417 mmol, 140 mg) as reagent. Yield: 67.1%; white crystals; ^1^H NMR (400 MHz, chloroform-*d*): δ 8.05 (d, *J* = 8.8 Hz, 2H, 2 × Ar–H), 6.94 (d, *J* = 8.8 Hz, 2H, 2 × Ar–H), 4.59 (tt, *J*_1_ = 7.1 Hz, *J*_2_ = 3.5 Hz, 1H, CH–piperidine), 3.70 (ddd, *J*_1_ = 12.0 Hz, *J*_2_ = 7.5 Hz, *J*_3_ = 3.8 Hz, 2H, CH_2_–piperidine), 3.38 (ddd, *J*_1_ = 13.5 Hz, *J*_2_ = 7.6 Hz, *J*_3_ = 3.8 Hz, 2H, CH_2_–piperidine), 1.94 (d, *J* = 8.9 Hz, 2H, CH_2_–piperidine), 1.86–1.72 (m, 2H, CH_2_–piperidine), 1.48 (s, 9H, 3 × CH_3_), signal for COOH not seen in the spectrum; MS (ESI^–^) *m*/*z*: 319.8 ([M – H]^−^).

##### Synthesis of Methyl 4-(2-Morpholinoethoxy)benzoate (**30**)

It was synthesized according to general procedure E using methyl 4-hydroxybenzoate (13.5 mmol, 2.06 g) and *N*-(2-chloroethyl)-morpholinium chloride (13.5 mmol, 2.52 g) as reagents. Yield: 50.8%; white crystals, ^1^H NMR (400 MHz, chloroform-*d*): δ 7.99 (d, *J* = 9.0 Hz, 2H, 2 × Ar–H), 6.92 (d, *J* = 9.0 Hz, 2H, 2 × Ar–H), 4.16 (t, *J* = 5.7 Hz, 2H, CH_2_) 3.88 (s, 3H, CH_3_), 3.74 (m, 4H, 2 × CH_2_–morpholine), 2.82 (t, *J* = 5.7 Hz, 2H, CH_2_) 2.58 (m, 4H, 2 × CH_2_–morpholine); HRMS (ESI^+^) for C_14_H_19_NO_4_ ([M + H]^+^): calcd, 266.1387; found, 266.1387.

##### Synthesis of 4-(2-Morpholinoethoxy)benzoic Acid (**31**)

It was synthesized according to general procedure D, using compound **30** (6.63 mmol, 1.76 g) as reagent. Yield: 26.2%; white solid; ^1^H NMR (400 MHz, DMSO-*d*_6_): δ 7.87 (d, *J* = 8.9 Hz, 2H, 2 × Ar–H), 7.02 (d, *J* = 8.9 Hz, 2H, 2 × Ar–H), 4.16 (t, *J* = 5.7 Hz, 2H, CH_2_), 3.58 (m, 4H, 2 × CH_2_–morpholine), 2.71 (t, *J* = 5.7 Hz, 2H, CH_2_), 2.47 (m, 4H, 2 × CH_2_–morpholine), signal for COOH not seen in the spectrum; HRMS (ESI^+^) za C_13_H_17_NO_4_ ([M + H]^+^): calcd, 252.1230; found, 252.1230.

##### Synthesis of *tert*-Butyl 4-((4-(4-Hydroxy-4-((*p*-tolyloxy)methyl)piperidine-1-carbonyl)phenoxy)methyl)piperidine-1-carboxylate (**32**)

It was synthesized according to general procedure A using compound **12** (0.36 mmol, 81 mg) and **26** (0.36 mmol, 123 mg) as reagents. Yield: 35.0%; ^1^H NMR (400 MHz, chloroform-*d*): δ 7.45–7.35 (m, 2H, 2 × Ar–H), 7.20 (t, *J* = 7.7 Hz, 1H, Ar–H), 6.95–6.87 (m, 2H, 2 × Ar–H), 6.83 (ddt, *J*_1_ = 7.7 Hz, *J*_2_ = 1.6 Hz, *J*_3_ = 0.8 Hz, 1H, Ar–H), 6.79–6.69 (m, 2H, 2 × Ar–H), 4.18 (s, 2H, CH_2_–piperidine), 3.85 (d, *J* = 6.0 Hz, 4H, 2 × CH_2_), 3.51 (d, *J* = 5.6 Hz, 2H, CH_2_–piperidine), 2.77 (t, *J* = 12.8 Hz, 2H, CH_2_–piperidine), 2.48–2.22 (m, 4H, CH_3_, CH–piperidine), 1.98 (ddt, *J*_1_ = 11.6 Hz, *J*_2_ = 8.2 Hz, *J*_3_ = 4.5 Hz, 1H, CH–piperidine), 1.84 (d, *J* = 13.2 Hz, 6H, 3 × CH_2_–piperidine), 1.49 (s, 9H, 3 × CH_3_), 1.29 (qd, *J*_1_ = 12.6 Hz, *J*_2_ = 4.8 Hz, 2H, CH_2_–piperidine), signal for OH not seen in the spectrum; MS (ESI^+^) *m*/*z*: 539.0 ([M + H]^+^).

##### Synthesis of *tert*-Butyl 4-((4-(4-((3,4-Dichlorophenoxy)methyl)-4-hydroxypiperidine-1-carbonyl)phenoxy)methyl)piperidine-1-carboxylate (**33**)

It was synthesized according to general procedure A, using compound **13** (0.36 mmol, 100 mg) and compound **26** (0.36 mmol, 128 mg) as reagents. Yield: 97.1%, white solid; ^1^H NMR (400 MHz, chloroform-*d*): δ 7.41–7.36 (m, 2H, 2 × Ar–H), 7.34 (d, *J* = 8.9 Hz, 1H, Ar–H), 7.02 (d, *J* = 2.8 Hz, 1H, Ar–H), 6.92–6.87 (m, 2H, 2 × Ar–H), 6.77 (dd, *J*_1_ = 8.9 Hz, *J*_2_ = 2.9 Hz, 1H, Ar–H), 4.14 (d, *J* = 7.1 Hz, 2H, CH_2_–piperidine), 3.83 (d, *J* = 6.4 Hz, 2H, CH_2_), 3.80 (s, 2H, CH_2_), 3.39 (s, 2H, CH_2_–piperidine), 2.75 (t, *J* = 12.7 Hz, 2H, CH_2_–piperidine), 2.14 (s, 1H, CH–piperidine), 2.03–1.93 (m, 1H, CH–piperidine), 1.82 (d, *J* = 13.4 Hz, 3H, CH_2_–piperidine, CH–piperidine), 1.67 (s, 4H, 2 × CH_2_–piperidine), 1.47 (s, 9H, 3 × CH_3_), 1.28 (s, 2H, CH_2_–piperidine), MS (ESI^+^) *m*/*z*: 593.0 ([M + H]^+^).

##### Synthesis of *tert*-Butyl 4-((4-(4-((3-Chloro-4-fluorophenoxy)methyl)-4-hydroxypiperidine-1-carbonyl)phenoxy)methyl)piperidine-1-carboxylate (**34**)

It was synthesized according to general procedure A, using compound **14** (0.283 mmol, 73.6 mg) and compound **26** (0.283 mmol, 95 mg) as reagents. Yield: 56.3%, off-white crystals; ^1^H NMR (400 MHz, chloroform-*d*): δ 7.38 (d, *J* = 8.6 Hz, 2H, 2 × Ar–H), 7.07 (t, *J* = 8.8 Hz, 1H, Ar–H), 6.95 (dd, *J*_1_ = 5.9 Hz, *J*_2_ = 3.0 Hz, 1H, Ar–H), 6.89 (d, *J* = 8.6 Hz, 2H, 2 × Ar–H), 6.80–6.73 (m, 1H, Ar–H), 4.51 (s, 1H, CH–piperidine), 4.12 (d, *J* = 7.1 Hz, 2H, CH_2_–piperidine), 3.83 (d, *J* = 6.3 Hz, 2H, CH_2_), 3.79 (s, 2H, CH_2_), 3.41 (s, 2H, CH_2_–piperidine), 2.75 (t, *J* = 11.5 Hz, 2H, CH_2_–piperidine), 2.16 (s, 1H, OH), 1.96 (d, *J* = 3.3 Hz, 2H, CH_2_–piperidine), 1.82 (d, *J* = 12.6 Hz, 2H, CH_2_–piperidine), 1.75 (s, 2H, CH_2_–piperidine), 1.65 (s, 2H, CH_2_–piperidine), 1.47 (s, 9H, 3 × CH_3_), 1.28 (s, 2H, CH_2_–piperidine); MS (ESI^+^) *m*/*z*: 598.9 ([M + Na]^+^).

##### Synthesis of *tert*-Butyl 4-((4-(4-((3-Chlorophenoxy)methyl)-4-hydroxypiperidine-1-carbonyl)phenoxy)methyl)piperidine-1-carboxylate (**35**)

It was synthesized according to general procedure A, using compound **15** (0.343 mmol, 83 mg) and compound **26** (0.343 mmol, 115 mg) as reagents. Yield: 82.8%; white solid; ^1^H NMR(400 MHz, chloroform-*d*): δ 7.38 (d, *J* = 8.7 Hz, 2H, 2 × Ar–H), 7.22 (t, *J* = 8.2 Hz, 1H, Ar–H), 6.98 (d, *J* = 6.7 Hz, 2H, 2 × Ar–H), 6.93–6.86 (m, 3H, 3 × Ar–H), 6.80 (dd, *J*_1_ = 8.3 Hz, *J*_2_ = 1.8 Hz, 1H, Ar–H), 4.52 (s, 1H, CH), 4.15 (s, 2H, CH_2_–piperidine), 3.83 (d, *J* = 5.2 Hz, 6H, 2 × CH_2_), 3.45 (s, 2H, CH_2_–piperidine), 2.75 (t, 2H, CH_2_–piperidine), 2.19 (s, 1H, OH), 1.97 (s, 2H, CH_2_–piperidine), 1.88–1.76 (m, 4H, 2 × CH_2_–piperidine), 1.67 (s, 2H, CH_2_–piperidine), 1.47 (s, 9H, 3 × CH_3_), 1.33 (m, 2H, CH_2_–piperidine); MS (ESI^+^) *m*/*z*: 581.0 ([M + Na]^+^).

##### Synthesis of *tert*-Butyl 4-((4-(4-((3-Chloro-4-cyanophenoxy)methyl)-4-hydroxypiperidine-1-carbonyl)phenoxy)methyl)piperidine-1-carboxylate (**36**)

It was synthesized according to general procedure A, using compound **16** (0.149 mmol, 40 mg) and compound **26** (0.149 mmol, 50 mg) as reagents. Yield: 73.1%, yellow oil; ^1^H NMR (400 MHz, chloroform-*d*): δ 7.60 (d, *J* = 8.7 Hz, 1H, Ar–H), 7.42–7.34 (m, 2H, 2 × Ar–H), 7.05 (d, *J* = 2.4 Hz, 1H, Ar–H), 6.89 (dd, *J*_1_ = 8.8 Hz, *J*_2_ = 2.5 Hz, 3H, 3 × Ar–H), 4.12 (s, 1H, CH–piperidine), 3.88 (s, 2H, CH_2_–piperidine), 3.83 (d, *J* = 6.4 Hz, 2H), 3.38 (s, 2H), 2.75 (s, 3H, CH_2_–piperidine), 2.07 (s, 1H, CH–piperidine), 2.03–1.92 (m, 1H, CH–piperidine), 1.82 (d, *J* = 13.2 Hz, 3H, CH_2_–piperidine, CH–piperidine), 1.62 (s, 2H, CH_2_–piperidine), 1.47 (s, 9H, 3 × CH_3_), 1.26 (t, *J* = 7.1 Hz, 4H, 2 × CH_2_–piperidine), signal for OH not seen in the spectrum; MS (ESI^+^) *m*/*z*: 605.9 ([M + Na]^+^).

##### Synthesis of *tert*-Butyl 4-((4-(4-((4-Chlorophenoxy)methyl)-4-hydroxypiperidine-1-carbonyl)phenoxy)methyl)piperidine-1-carboxylate (**37**)

It was synthesized according to general procedure A using compound **17** (0.91 mmol, 220 mg) and compound **26** (0.91 mmol, 305 mg) as reagents. Yield: 98.7%, white solid; ^1^H NMR (400 MHz, chloroform-*d*): δ 7.40–7.35 (m, 2H, 2 × Ar–H), 7.27–7.21 (m, 2H, 2 × Ar–H), 6.91–6.86 (m, 2H, 2 × Ar–H), 6.86–6.80 (m, 2H, 2 × Ar–H), 4.15 (s, 2H, CH_2_–piperidine), 3.82 (d, *J* = 6.4 Hz, 2H, CH_2_), 3.79 (s, 2H, CH_2_), 3.39 (s, 1H, CH–piperidine), 2.75 (t, *J* = 12.7 Hz, 2H, CH_2_–piperidine), 2.40 (s, 1H, CH–piperidine), 2.04–1.90 (m, 2H, CH_2_–piperidine), 1.91–1.73 (m, 5H, 2 × CH_2_–piperidine, CH–piperidine), 1.66 (s, 2H, CH_2_–piperidine), 1.47 (s, 9H, 3 × CH_3_), 1.35–1.27 (m, 2H, CH_2_–piperidine), signal for OH not seen in the spectrum; MS (ESI^+^) *m*/*z*: 559.2 ([M + H]^+^).

##### Synthesis of *tert*-Butyl 4-(4-(4-((3,4-Dichlorophenoxy)methyl)-4-hydroxypiperidine-1-carbonyl)phenoxy)piperidine-1-carboxylate (**38**)

It was synthesized according to general procedure A using compound **13** (0.36 mmol, 110 mg) and compound **29** (0.36 mmol, 116 mg) as reagents. Yield: 71.0%; white solid; ^1^H NMR (400 MHz, chloroform-*d*): δ 7.38 (d, *J* = 2.0 Hz, 1H, Ar–H), 7.37 (d, *J* = 2.0 Hz, 1H, Ar–H), 7.34 (d, *J* = 8.9 Hz, 1H, Ar–H), 7.01 (d, *J* = 2.8 Hz, 1H, Ar–H), 6.92 (d, *J* = 1.9 Hz, 1H, Ar–H), 6.90 (s, 1H, Ar–H), 6.77 (dd, *J*_1_ = 8.8 Hz, *J*_2_ = 2.8 Hz, 1H, Ar–H), 4.53–4.50 (m, 1H, CH–piperidine), 3.80 (s, 2H, CH_2_), 3.75–3.62 (m, 4H, 2 × CH_2_–piperidine), 3.41–3.28 (m, 4H, 2 × CH_2_–piperidine), 2.15 (s, 1H, OH), 1.96–1.90 (m, 2H, CH_2_–piperidine), 1.83–1.70 (m, 6H, 3 × CH_2_–piperidine), 1.47 (s, 9H, 3 × CH_3_); MS (ESI^+^) *m*/*z*: 600.9 ([M + Na]^+^).

##### Synthesis of *tert*-Butyl 4-(4-(4-((3-Chloro-4-fluorophenoxy)methyl)-4-hydroxypiperidine-1-carbonyl)phenoxy)piperidine-1-carboxylate (**39**)

It was synthesized according to general procedure A using compound **14** (0.284 mmol, 62 mg) and compound **29** (0.243 mmol, 78 mg) as reagents. Reaction mixture was stirred for 4 days. Yield: 89.3%; yellow solid; ^1^H NMR (400 MHz, chloroform-*d*): δ 7.42–7.35 (m, 2H, 2 × Ar–H), 7.06 (t, *J* = 8.8 Hz, 1H, Ar–H), 6.98–6.89 (m, 3H, 3 × Ar–H), 6.77 (dt, *J* = 8.7 Hz, 1H, Ar–H), 4.55–4.48 (m, 2H, CH_2_–piperidine), 3.79 (s, 2H, CH_2_), 3.74–3.65 (m, 3H, CH_2_–piperidine, CH–piperidine), 3.51–3.19 (m, 4H, 2 × CH_2_–piperidine), 2.29–2.19 (m, 1H, OH), 1.99–1.86 (m, 2H, CH_2_–piperidine), 1.84–1.70 (m, 4H, 2 × CH_2_–piperidine), 1.62 (s, 2H, CH_2_–piperidine), 1.47 (s, 9H, 3 × CH_3_); MS (ESI^+^) *m*/*z*: 585.0 ([M + Na]^+^).

##### Synthesis of *tert*-Butyl 4-((4-(4-((4-Bromo-3-chlorophenoxy)methyl)-4-hydroxypiperidine-1-carbonyl)phenoxy)methyl)piperidine-1-carboxylate (**40**)

It was synthesized according to general procedure A using compound **8** (0.94 mmol, 300 mg) and **26** (0.94 mmol, 316 mg) as reagents. Yield: 20.0%; yellow oil; ^1^H NMR (400 MHz, chloroform-*d*): δ 7.37 (d, *J* = 8.9 Hz, 1H, Ar–H), 7.31–7.24 (d, 2H, 2 × Ar–H), 6.91 (d, *J* = 2.9 Hz, 1H, Ar–H), 6.84–6.76 (d, 2H, 2 × Ar–H), 6.59 (dd, *J*_1_ = 8.9 Hz, *J*_2_ = 2.9 Hz, 1H, Ar–H), 4.07 (s, 2H, CH_2_–piperidine), 3.72 (d, *J* = 6.3 Hz, 2H, CH_2_), 3.67 (s, 2H, CH_2_), 3.37 (s, 2H, CH_2_–piperidine), 2.96 (s, 1H, CH–piperidine), 2.66 (s, 2H, CH_2_–piperidine), 1.87 (t, 1H, CH–piperidine), 1.73 (d, 2H, CH_2_–piperidine), 1.59 (d, 4H, 2 × CH_2_–piperidine), 1.38 (s, 9H, 3 × CH_3_), 1.18 (qd, *J*_1_ = 12.6 Hz, *J*_2_ = 4.2 Hz, 3H, CH_2_–piperidine, CH–piperidine), signal for OH not seen in the spectrum; MS (ESI^+^) *m*/*z*: 636.8 ([M + H]^+^).

##### Synthesis of *tert*-Butyl 4-((4-(4-((4-Chloro-3-fluorophenoxy)methyl)-4-hydroxypiperidine-1-carbonyl)phenoxy)methyl)piperidine-1-carboxylate (**41**)

It was synthesized according to general procedure A using compound **9** (2.04 mmol, 380 mg) and **26** (2.04 mmol, 685 mg) as reagents. Yield: 69.6%; white solid; ^1^H NMR (400 MHz, chloroform-*d*): δ 7.38 (d, *J* = 8.7 Hz, 2H, 2 × Ar–H), 7.30 (d, *J* = 8.7 Hz, 1H, Ar–H), 6.89 (d, *J* = 8.7 Hz, 2H, 2 × Ar–H), 6.72 (dd, *J*_1_ = 10.6 Hz, *J*_2_ = 2.8 Hz, 1H, Ar–H), 6.66 (ddd, *J*_1_ = 8.9 Hz, *J*_2_ = 2.9 Hz, *J*_3_ = 1.2 Hz, 1H, Ar–H), 4.50 (s, 1H, CH–piperidine), 4.15 (s, 3H, CH–piperidine, CH_2_–piperidine), 3.83 (d, *J* = 6.4 Hz, 2H, CH_2_), 3.80 (s, 2H, CH_2_), 3.38 (s, 3H, CH–piperidine, CH_2_–piperidine), 2.75 (s, 2H, CH_2_–piperidine), 1.97 (s, 1H, CH–piperidine), 1.82 (d, *J* = 13.3 Hz, 4H, 2 × CH_2_–piperidine), 1.47 (s, 9H, 3 × CH_3_), 1.33–1.21 (m, 3H, CH–piperidine, CH_2_–piperidine), signal for OH not seen in the spectrum; MS (ESI^+^) *m*/*z*: 577.4 ([M + H]^+^).

##### Synthesis of *tert*-Butyl 4-((4-(4-((4-Chloro-3-bromo)methyl)-4-hydroxypiperidine-1-carbonyl)phenoxy)methyl)piperidine-1-carboxylate (**42**)

It was synthesized according to general procedure A using compound **10** (1.56, 500 mg) and **26** (1.56 mmol, 523 mg) as reagents. Yield: 62.4%; white solid; ^1^H NMR (400 MHz, chloroform-*d*): δ 7.41–7.35 (m, 3H, 3 × Ar–H), 7.18 (d, *J* = 2.9 Hz, 1H, Ar–H), 6.89 (d, *J* = 8.8 Hz, 2H, 2 × Ar–H), 6.82 (dd, *J*_1_ = 8.8 Hz, *J*_2_ = 2.9 Hz, 1H, Ar–H), 4.16 (d, *J* = 8.3 Hz, 2H, CH_2_–piperidine), 3.82 (d, *J* = 6.4 Hz, 2H, CH_2_), 3.80 (s, 2H, CH_2_), 3.75–3.67 (m, 1H, CH–piperidine), 3.07 (d, *J* = 15.5 Hz, 1H, CH–piperidine), 2.75 (s, 3H, CH–piperidine, CH_2_–piperidine), 2.02–1.91 (m, 2H, CH_2_–piperidine), 1.86–1.76 (m, 4H, 2 × CH_2_–piperidine), 1.47 (s, 11H, CH_2_–piperidine, 3 × CH_3_), 1.31 (d, *J* = 6.6 Hz, 2H, CH_2_–piperidine), signal for OH not seen in the spectrum; MS (ESI^+^) *m*/*z*: 533.0 ([M-Boc + H]^+^).

##### Synthesis of *tert*-Butyl 4-((4-(4-((4-Chloro-3-iodo)methyl)-4-hydroxypiperidine-1-carbonyl)phenoxy)methyl)piperidine-1-carboxylate (**43**)

It was synthesized according to general procedure A using compound **11** (1.31, 480 mg) and **26** (1.31 mmol, 430 mg) as reagents. The product was used in the next step without purification. Yield: 95.0%; white solid; MS (ESI+) *m*/*z*: 584.0 ([M-Boc + H]^+^).

##### Synthesis of *tert*-Butyl 4-((4-((4-((3,4-Dichlorophenoxy)methyl)-4-hydroxypiperidin-1-yl)methyl)phenoxy)methyl)piperidine-1-carboxylate (**44**)

It was synthesized according to general procedure B using compound **26** (0.313 mmol, 86.4 mg) and **13** (0.313 mmol, 100 mg) as reagents. The reaction mixture was stirred for 18 h. The crude product was purified using flash column chromatography using dichloromethane/methanol (9:1) as eluent. Yield: 11.3%; yellow solid; ^1^H NMR (400 MHz, chloroform-*d*): δ 7.39–7.34 (m, 2H, 2 × Ar–H), 7.30 (d, *J* = 8.9 Hz, 1H, Ar–H), 6.98 (d, *J* = 2.9 Hz, 1H, Ar–H), 6.88 (d, *J* = 8.5 Hz, 2H, 2 × Ar–H), 6.73 (dd, *J*_1_ = 8.9 Hz, *J*_2_ = 2.9 Hz, 1H, Ar–H), 4.15 (s, 2H, CH_2_–piperidine), 3.98 (s, 2H, CH_2_), 3.83 (s, 2H, CH_2_), 3.80 (d, *J* = 6.3 Hz, 2H, CH_2_), 3.23 (d, *J* = 11.5 Hz, 2H, CH_2_–piperidine), 3.00–2.90 (m, 2H, CH_2_–piperidine), 2.73 (d, *J* = 13.2 Hz, 2H, CH_2_–piperidine), 2.18 (td, *J*_1_ = 13.4 Hz, *J*_2_ = 4.1 Hz, 2H, CH_2_–piperidine), 2.00–1.92 (m, 1H, CH–piperidine), 1.90–1.76 (m, 4H, 2 × CH_2_–piperidine), 1.46 (s, 9H, 3 × CH_3_), 1.27 (dt, *J*_1_ = 12.5 Hz, *J*_2_ = 6.2 Hz, 2H, CH_2_–piperidine), signal for OH not seen in the spectrum; MS (ESI^+^) *m*/*z*: 579.2 ([M + H]^+^).

##### Synthesis of 4-((4-(4-Hydroxy-4-((*m*-tolyloxy)methyl)piperidine-1-carbonyl)phenoxy)methyl)piperidin-1-ium Chloride (**45**)

It was synthesized according to general procedure C using compound **32** (0.128 mmol, 69 mg) as reagent. Yield: 46.0%; white solid; ^1^H NMR (400 MHz, DMSO-*d*_6_): δ 8.90 (d, *J* = 11.4 Hz, 1H, NH), 8.65–8.40 (m, 1H, NH), 7.37 (s, 1H, Ar–H), 7.15 (t, *J* = 7.8 Hz, 2H, 2 × Ar–H), 7.03–6.91 (m, 2H, 2 × Ar–H), 6.84–6.58 (m, 3H, 3 × Ar–H), 4.21 (s, 1H, CH–piperidine), 3.90 (d, *J* = 6.2 Hz, 2H, CH_2_), 3.76 (s, 2H, CH_2_), 3.54–3.46 (m, 6H, 6 × CH–piperidine), 3.29 (d, *J* = 12.6 Hz, 2H, CH_2_–piperidine), 2.90 (q, *J* = 12.0 Hz, 2H, CH_2_–piperidine), 2.27 (s, 3H), 2.15–2.02 (m, 1H, CH–piperidine), 1.92 (dd, *J*_1_ = 11.8 Hz, *J*_2_ = 4.1 Hz, 2H, CH_2_–piperidine), 1.74–1.40 (m, 4H, 2 × CH_2_–piperidine), signal for OH not seen in the spectrum; ^13^C NMR (101 MHz, DMSO-*d*_6_): δ 169.3, 159.9, 159.3, 154.3, 139.4, 129.6, 129.2, 128.7, 121.8, 115.7, 114.5, 112.0, 79.0, 75.6, 72.2, 68.7, 43.6, 35.7, 28.7, 28.6, 21.6; HRMS (ESI^+^) for C_26_H_34_N_2_O_4_ ([M + H]^+^): calcd, 438.2519; found, 439.2521; HPLC: *t*_R_ = 5.78 min (98.2% at 254 nm).

##### Synthesis of 4-((4-(4-((3,4-Dichlorophenoxy)methyl)-4-hydroxypiperidine-1-carbonyl)phenoxy)methyl)piperidin-1-ium Chloride (**46**)

It was synthesized according to general procedure C using compound **33** (0.141 mmol, 84 mg) as reagent. Yield: 53.0%; white solid; ^1^H NMR (400 MHz, DMSO-*d*_6_): δ 9.00 (s, NH), 8.67 (s, NH), 7.52 (d, *J* = 8.9 Hz, 1H, Ar–H), 7.36 (d, *J* = 8.2 Hz, 2H, 2 × Ar–H), 7.26 (d, *J* = 2.9 Hz, 1H, Ar–H), 6.99 (dd, *J*_1_ = 8.8 Hz, *J*_2_ = 3.7 Hz, 3H, 3 × Ar–H), 4.91 (s, 1H, CH–piperidine), 4.22 (s, 1H, CH–piperidine), 3.90 (d, *J* = 6.2 Hz, 2H, CH_2_), 3.84 (s, 2H, CH_2_), 3.30–3.20 (m, 3H, CH_2_–piperidine, CH–piperidine), 2.89 (t, *J* = 12.8 Hz, 2H, CH_2_–piperidine), 2.06 (d, *J* = 13.3 Hz, 1H, CH–piperidine), 1.91 (dt, *J*_1_ = 9.0 Hz, *J*_2_ = 3.7 Hz, 2H, CH_2_–piperidine), 1.78–1.35 (m, 7H, 7 × CH–piperidine), signal for OH not seen in the spectrum; ^13^C NMR (101 MHz, DMSO-*d*_6_): δ 169.3, 159.8, 158.8, 132.0, 131.4, 129.2, 128.9, 122.8, 117.0, 116.1, 114.6, 76.4, 71.7, 68.7, 66.8, 43.0, 33.5, 25.6; HRMS (ESI^+^) for C_25_H_30_Cl_2_N_2_O_4_ ([M + H]^+^): calcd, 492.1583; found, 493.1547; HPLC: *t*_R_ = 3.99 min (95.5% at 254 nm).

##### Synthesis of 4-((4-(4-((3-Chloro-4-fluorophenoxy)methyl)-4-hydroxypiperidine-1-carbonyl)phenoxy)methyl)piperidin-1-ium Chloride (**47**)

It was synthesized according to general procedure C using compound **34** (0.121 mmol, 70 mg) as reagent. The reaction mixture was stirred for 2 days. Yield: 53.0%; yellow solid; ^1^H NMR (400 MHz, DMSO-*d*_6_): δ 8.77 (s, 1H, NH), 8.46 (s, 1H, NH), 7.40–7.29 (m, 3H, 3 × Ar–H), 7.20 (dd, *J*_1_ = 6.0 Hz, *J*_2_ = 3.0 Hz, 1H, Ar–H), 7.04–6.93 (m, 3H, 3 × Ar–H), 4.89 (s, 1H, CH–piperidine), 3.91 (d, *J* = 6.1 Hz, 2H, CH_2_), 3.81 (s, 2H, CH_2_), 3.57 (s, 2H, CH_2_–piperidine), 3.29 (s, 4H, 2 × CH_2_–piperidine), 2.99–2.83 (m, 2H, CH_2_–piperidine), 2.07 (s, 1H, OH), 1.92 (d, *J* = 13.8 Hz, 2H, CH_2_–piperidine), 1.71–1.41 (m, 6H, 3 × CH_2_–piperidine); ^13^C NMR (101 MHz, DMSO-*d*_6_): δ 213.0, 169.2, 159.7, 156.0, 138.8, 138.1, 129.3, 128.9, 116.6, 114.6, 100.0, 76.7, 71.7, 68.7, 43.1, 41.1, 33.5, 25.6; HRMS (ESI^+^) for C_25_H_31_ClFN_2_O_4_ ([M + H]^+^): calcd, 477.1951; found, 477.1947; HPLC: *t*_R_ = 4.14 min (99.2% at 254 nm).

##### Synthesis of 4-((4-(4-((3-Chlorophenoxy)methyl)-4-hydroxypiperidine-1-carbonyl)phenoxy)methyl)piperidin-1-ium Chloride (**48**)

It was synthesized according to general procedure C using compound **35** (0.229 mmol, 128 mg) as reagent. Yield: 78.5%; white-yellow solid; ^1^H NMR (400 MHz, chloroform-*d*): δ 9.74 (s, 1H, NH), 9.44 (s, 1H, NH), 7.39 (d, *J* = 8.5 Hz, 2H, 2 × Ar–H), 7.22 (t, *J* = 8.2 Hz, 1H, Ar–H), 7.00–6.96 (m, 1H, Ar–H), 6.92 (t, *J* = 2.2 Hz, 1H, Ar–H), 6.89 (d, *J* = 2.3 Hz, 2H, 2 × Ar–H), 6.80 (ddd, *J*_1_ = 8.4 Hz, *J*_2_ = 1.7 Hz, 1H, Ar–H), 4.51 (s, 1H, CH–piperidine), 3.88 (d, *J* = 5.6 Hz, 2H, CH_2_), 3.82 (s, 2H, CH_2_), 3.63–3.23 (m, 4H, 2 × CH_2_–piperidine), 2.94 (s, 2H, CH_2_–piperidine), 2.19 (s, 1H, OH), 2.14–2.05 (m, 4H, 2 × CH_2_–piperidine), 1.83 (d, *J* = 12.1 Hz, 6H, 3 × CH_2_–piperidine); ^13^C NMR (101 MHz, DMSO-*d*_6_): δ 189.5, 169.3, 159.8, 134.1, 131.3, 129.3, 128.9, 121.0, 115.2, 114.6, 114.2, 94.1, 76.0, 71.7, 68.7, 43.1, 39.6, 33.5, 25.6; HRMS (ESI^+^) for C_25_H_32_ClN_2_O_4_ ([M + H]^+^): calcd, 459.2045; found, 459.2038; HPLC: *t*_R_ = 3.71 min (96.1% at 254 nm).

##### Synthesis of 4-((4-(4-((3-Chloro-4-cyanophenoxy)methyl)-4-hydroxypiperidine-1-carbonyl)phenoxy)methyl)piperidin-1-ium Chloride (**49**)

It was synthesized according to general procedure C using compound **36** (0.086 mmol, 50 mg) as reagent. Yield: 80.8%; white solid; ^1^H NMR (400 MHz, DMSO-*d*_6_): δ 8.70 (s, 1H, NH), 8.37 (s, 1H, NH), 7.89 (d, *J* = 8.8 Hz, 1H, Ar–H), 7.42–7.30 (m, 3H, 3 × Ar–H), 7.14 (dd, *J*_1_ = 8.8 Hz, *J*_2_ = 2.4 Hz, 1H, Ar–H), 6.98 (d, *J* = 8.8 Hz, 2H, 2 × Ar–H), 4.95 (s, 1H, CH–piperidine), 4.00–3.87 (m, 4H, 2 × CH_2_), 3.30 (s, 2H, CH_2_–piperidine), 2.98–2.83 (m, 2H, CH_2_–piperidine), 2.67 (s, 2H, CH_2_–piperidine), 2.08 (s, 1H, OH), 1.91 (s, 4H, 2 × CH_2_–piperidine), 1.62 (s, 4H, 2 × CH_2_–piperidine), 1.47 (d, *J* = 11.5 Hz, 2H, CH_2_–piperidine); ^13^C NMR (101 MHz, DMSO-*d*_6_): δ 169.3, 163.6, 159.8, 137.4, 136.2, 129.3, 128.9, 116.9, 116.7, 115.5, 114.6, 103.9, 100.0, 76.5, 71.7, 68.6, 43.1, 33.5, 25.6; HRMS (ESI^+^) za C_26_H_31_ClN_3_O_4_ ([M + H]^+^): calcd, 484.1998; found, 484.1992; HPLC: *t*_R_ = 3.51 min (93.5% at 254 nm).

##### Synthesis of 4-((4-(4-((4-Chlorophenoxy)methyl)-4-hydroxypiperidine-1-carbonyl)phenoxy)methyl)piperidin-1-ium Chloride (**50**)

It was synthesized according to general procedure C using compound **37** (0.90 mmol, 508 mg) as reagent. Yield: 52.1%; white solid; ^1^H NMR (400 MHz, chloroform-*d*): δ 7.38 (d, *J* = 8.7 Hz, 2H, 2 × Ar–H), 7.24 (s, 2H, 2 × Ar–H), 6.89 (d, *J* = 8.7 Hz, 2H, 2 × Ar–H), 6.84 (d, *J* = 9.0 Hz, 2H, 2 × Ar–H), 3.84–3.78 (m, 4H, 2 × CH_2_), 3.17–3.11 (m, 2H, CH_2_–piperidine), 2.66 (td, *J*_1_ = 12.2 Hz, *J*_2_ = 2.6 Hz, 2H, CH_2_–piperidine), 2.00–1.90 (m, 1H, CH–piperidine), 1.83 (d, *J* = 13.2 Hz, 3H, CH–piperidine, CH_2_–piperidine), 1.68 (s, 6H, 3 × CH_2_–piperidine), 1.35–1.24 (m, 3H, CH–piperidine, CH_2_–piperidine), signal for OH not seen in the spectrum; ^13^C NMR (101 MHz, chloroform-*d*): δ 170.4, 160.3, 157.1, 129.4, 128.9, 127.9, 126.2, 115.8, 114.2, 76.8, 75.9, 73.0, 69.2, 46.2, 36.3, 30.1; HRMS (ESI^+^) for C_25_H_31_ClN_2_O_4_ ([M + H]^+^): calcd, 459.20451; found, 459.20355; HPLC: *t*_R_ = 4.17 min (95.1% at 254 nm).

##### Synthesis of 4-(4-(4-((3,4-Dichlorophenoxy)methyl)-4-hydroxypiperidine-1-carbonyl)phenoxy)piperidin-1-ium Chloride (**51**)

It was synthesized according to general procedure C using compound **38** (0.183 mmol, 112 mg) as reagent. Yield: 92.0%; yellow oil; ^1^H NMR (400 MHz, DMSO-*d*_6_): δ 8.86 (s, 2H, 2 × NH), 7.52 (d, *J* = 8.9 Hz, 1H, Ar–H), 7.36 (d, *J* = 8.7 Hz, 2H, 2 × Ar–H), 7.26 (d, *J* = 2.9 Hz, 1H, Ar–H), 7.04 (d, *J* = 8.7 Hz, 2H, 2 × Ar–H), 6.98 (dd, *J*_1_ = 8.9 Hz, *J*_2_ = 2.9 Hz, 1H, Ar–H), 4.73–4.68 (m, 1H, CH–piperidine), 4.22 (s, 1H, OH), 3.84 (s, 2H, CH_2_), 3.29–3.16 (m, 4H, 2 × CH_2_–piperidine), 3.19–2.96 (m, 4H, 2 × CH_2_–piperidine), 2.14–2.08 (m, 2H, CH_2_–piperidine), 1.87–1.81 (m, 2H, CH_2_–piperidine), 1.66–1.54 (m, 4H, 2 × CH_2_–piperidine); ^13^C NMR (101 MHz, DMSO-*d*_6_): δ 172.5, 169.2, 158.8, 157.9, 132.0, 131.4, 129.3, 129.2, 122.8, 117.0, 116.1, 115.8, 76.4, 69.6, 68.7, 33.8, 27.5; HRMS (ESI^+^) for C_24_H_29_Cl_2_N_2_O_4_ ([M + H]^+^): calcd, 479.15099; found, 479.14893; HPLC: *t*_R_ = 3.87 min (98.0% at 254 nm).

##### Synthesis of 4-(4-(4-((3-Chloro-4-fluorophenoxy)methyl)-4-hydroxypiperidine-1-carbonyl)phenoxy)piperidin-1-ium Chloride (**52**)

It was synthesized according to general procedure C using compound **39** (0.217 mmol, 120 mg) as reagent. The reaction mixture was stirred for 2 days. Yield: 98.1%; yellow oil; ^1^H NMR (400 MHz, chloroform-*d*):: δ 9.53 (s, 2H, 2 × NH), 7.41 (s, 2H, 2 × Ar–H), 7.07 (t, *J* = 8.7 Hz, 1H, Ar–H), 6.98–6.85 (m, 3H, 3 × Ar–H), 6.77 (d, *J* = 8.6 Hz, 1H, Ar–H), 4.72 (s, 1H, CH–piperidine), 4.52 (s, 1H, OH), 3.80 (s, 2H, CH_2_), 3.71 (s, 2H, CH_2_–piperidine), 3.44–3.01 (m, 6H, 3 × CH_2_–piperidine), 2.32 (s, 2H, CH_2_–piperidine), 2.18 (s, 2H, CH_2_–piperidine), 1.77 (s, 4H, 2 × CH_2_–piperidine); ^13^C NMR (101 MHz, DMSO-*d*_6_): δ 172.5, 169.2, 157.9, 156.090, 156.0, 129.3, 129.2, 120.2, 120.0, 117.7, 117.5, 116.6, 115.7, 76.6, 69.6, 68.7, 27.5, 21.6; HRMS (ESI^+^) for C_24_H_29_ClFN_2_O_4_ ([M + H]^+^): calcd, 463.1794; found, 463.1790; HPLC: *t*_R_ = 3.64 min (84.1% at 254 nm).

##### Synthesis of 4-((4-(4-((4-Bromo-3-chlorophenoxy)methyl)-4-hydroxypiperidine-1-carbonyl)phenoxy)methyl)piperidin-1-ium Chloride (**53**)

It was synthesized according to general procedure C using compound **40** (0.188 mmol, 120 mg) as reagent. Yield: 49.4%; white solid; ^1^H NMR (400 MHz, DMSO-*d*_6_): 8.59 (s, 1H, NH), 8.28 (s, 1H, NH), 7.64 (d, *J* = 8.9 Hz, 1H, Ar–H), 7.36 (d, *J* = 8.7 Hz, 2H, 2 × Ar–H), 7.26 (d, *J* = 2.9 Hz, 1H, Ar–H), 6.98 (d, *J* = 8.7 Hz, 2H, 2 × Ar–H), 6.92 (dd, *J*_1_ = 8.9 Hz, *J*_2_ = 2.9 Hz, 1H, Ar–H), 3.91 (d, *J* = 6.2 Hz, 2H, CH_2_), 3.84 (s, 2H, CH_2_), 2.97–2.84 (m, 4H, 2 × CH_2_–piperidine), 2.09 (s, 3H, CH–piperidine, CH_2_–piperidine), 1.92 (d, *J* = 13.9 Hz, 3H, CH–piperidine, CH_2_–piperidine), 1.60 (s, 4H, 2 × CH_2_–piperidine), 1.52–1.40 (m, 3H, CH–piperidine, CH_2_–piperidine), signal for OH not seen in the spectrum; ^13^C NMR (101 MHz, DMSO-*d*_6_): δ 169.3, 159.8, 159.4, 134.5, 134.0, 129.5, 129.2, 128.9, 117.1, 116.5, 114.6, 112.20, 76.3, 71.7, 68.7, 66.8, 43.0, 33.5, 25.6; HRMS (ESI^+^) for C_25_H_30_BrClN_2_O_4_ ([M + H]^+^): calcd, 536.1077; found, 537.1137; HPLC: *t*_R_ = 4.093 min (97.9% at 254 nm).

##### Synthesis of 4-((4-(4-((3-Fluoro-4-chlorophenoxy)methyl)-4-hydroxypiperidine-1-carbonyl)phenoxy)methyl)piperidin-1-ium Chloride (**54**)

It was synthesized according to general procedure C using compound **41** (1.42 mmol, 820 mg) as reagent. Yield: 93.0%; white solid; ^1^H NMR (400 MHz, DMSO-*d*_6_): δ 9.12 (d, *J* = 11.1 Hz, 1H, NH), 8.78 (d, *J* = 11.5 Hz, 1H, NH), 7.46 (t, *J* = 8.9 Hz, 1H, Ar–H), 7.35 (d, *J* = 8.7 Hz, 2H, 2 × Ar–H), 7.09 (dd, *J*_1_ = 11.5 Hz, *J*_2_ = 2.8 Hz, 1H, Ar–H), 6.98 (d, *J* = 8.8 Hz, 2H, 2 × Ar–H), 6.85 (ddd, *J*_1_ = 9.0 Hz, *J*_2_ = 2.9 Hz, *J*_3_ = 1.2 Hz, 1H, Ar–H), 3.90 (d, *J* = 6.2 Hz, 2H, CH_2_), 3.83 (s, 2H, CH_2_), 3.28 (d, *J* = 12.6 Hz, 4H, 2 × CH_2_–piperidine), 2.96–2.81 (m, 2H, CH_2_–piperidine), 2.14–2.00 (m, 1H, CH–piperidine), 1.91 (t, *J* = 6.6 Hz, 2H, CH_2_–piperidine), 1.61 (s, 4H, 2 × CH_2_–piperidine), 1.57–1.45 (m, 3H, CH–piperidine, CH_2_–piperidine); signal for OH not seen in the spectrum; ^13^C NMR (101 MHz, DMSO-*d*_6_): δ 169.3, 159.8, 159.6, 159.5, 156.8, 131.1, 129.2, 128.9, 114.6, 112.9, 104.4, 104.1, 76.4, 71.8, 68.7, 66.8, 43.0, 33.5, 25.6; HRMS (ESI^+^) for C_25_H_30_ClFN_2_O_4_ ([M + H]^+^): calcd, 476.1878; found, 477.1938; HPLC: *t*_R_ = 3.83 min (96.7% at 254 nm).

##### Synthesis of 4-((4-(4-((3-Bromo-4-chlorophenoxy)methyl)-4-hydroxypiperidine-1-carbonyl)phenoxy)methyl)piperidin-1-ium Chloride (**55**)

It was synthesized according to general procedure C using compound **42** (0.97 mmol, 620 mg) as reagent. Yield: 95.5%; white solid; NMR (400 MHz, DMSO-*d*_6_): δ 9.03 (d, *J* = 11.4 Hz, 1H, NH), 8.69 (d, *J* = 10.6 Hz, 1H, NH), 7.51 (d, *J* = 8.9 Hz, 1H, Ar–H), 7.38–7.32 (m, 3H, 3 × Ar–H), 7.02 (dd, *J*_1_ = 8.9 Hz, *J*_2_ = 2.9 Hz, 1H, Ar–H), 7.00–6.95 (m, 2H, 2 × Ar–H), 3.90 (d, *J* = 6.3 Hz, 2H, CH_2_), 3.84 (s, 2H, CH_2_), 3.28 (d, *J* = 12.5 Hz, 3H, CH–piperidine, CH_2_–piperidine), 3.17 (s, 1H, CH–piperidine), 2.88 (dd, *J*_1_ = 15.1 Hz, *J*_2_ = 8.8 Hz, 3H, CH–piperidine, CH_2_–piperidine), 2.12–1.99 (m, 1H, CH–piperidine), 1.95–1.86 (m, 2H, CH_2_–piperidine), 1.60 (s, 3H, CH–piperidine, CH_2_–piperidine), 1.51 (q, *J*_1_ = 12.9, *J*_2_ = 11.8 Hz, 3H, CH–piperidine, CH_2_–piperidine), signal for OH not seen in the spectrum; ^13^C NMR (101 MHz, DMSO-*d*_6_): δ 169.3, 159.8, 158.7, 131.2, 129.5, 129.2, 128.9, 124.8, 122.2, 120.1, 116.6, 114.6, 76.4, 71.7, 68.7, 66.8, 43.0, 33.5, 25.6; HRMS (ESI^+^) for C_25_H_30_BrClNO_4_ ([M + H]^+^): calcd, 536.1077; found, 537.11352; HPLC: *t*_R_ = 4.087 min (94.9% at 254 nm).

##### Synthesis of 4-((4-(4-((3-Iodo-4-chlorophenoxy)methyl)-4-hydroxypiperidine-1-carbonyl)phenoxy)methyl)piperidin-1-ium Chloride (**56**)

It was synthesized according to general procedure C using compound **43** (1.34 mmol, 920 mg) as reagent. Yield: 89.1%; white solid; ^1^H NMR (400 MHz, DMSO-*d*_6_): δ 9.20 (d, *J* = 11.1 Hz, 1H, NH), 8.85 (d, *J* = 10.5 Hz, 1H, NH), 7.50 (t, *J* = 2.9 Hz, 1H, Ar–H), 7.45 (d, *J* = 8.9 Hz, 1H, Ar–H), 7.38–7.32 (m, 2H, 2 × Ar–H), 7.02 (dd, *J*_1_ = 8.8 Hz, *J*_2_ = 2.9 Hz, 1H, Ar–H), 7.00–6.94 (m, 2H, 2 × Ar–H), 3.90 (d, *J* = 6.3 Hz, 2H, CH_2_), 3.81 (s, 2H, CH_2_), 3.27 (d, *J* = 12.6 Hz, 2H, CH_2_–piperidine), 2.89 (m, 4H, 2 × CH_2_–piperidine), 2.04 (d, *J* = 18.2 Hz, 1H, CH–piperidine), 1.90 (d, *J* = 8.0 Hz, 2H, CH_2_–piperidine), 1.61 (m, 2H, CH_2_–piperidine), 1.52 (q, *J* = 11.0 Hz, 3H, CH–piperidine, CH_2_–piperidine), signal for OH not seen in the spectrum; ^13^C NMR (101 MHz, DMSO-*d*_6_): δ 169.3, 159.8, 158.3, 130.0, 129.2, 129.2, 128.9, 126.2, 117.1, 114.6, 99.6, 76.3, 71.8, 68.7, 66.9, 43.0, 34.4, 33.6, 25.5; HRMS (ESI^+^) for C_25_H_30_ClIN_2_O_6_ ([M + H]^+^): calcd, 584.0939; found, 585.09954; HPLC: *t*_R_ = 5.060 min (95.8% at 254 nm).

##### Synthesis of 4-((3,4-Dichlorophenoxy)methyl)-1-(4-(piperidin-4-ylmethoxy)benzyl)piperidin-4-ol (**57**)

It was synthesized according to general procedure C using compound **44** (0.034 mmol, 20 mg) as reagent. Yield: 100%, yellow solid; ^1^H NMR (400 MHz, DMSO-*d*_6_): δ 7.53 (d, *J* = 8.9 Hz, 1H, Ar–H), 7.48 (d, *J* = 8.3 Hz, 2H, 2 × Ar–H), 7.23 (d, *J* = 2.9 Hz, 1H, Ar–H), 7.02 (d, *J* = 8.1 Hz, 2H, 2 × Ar–H), 6.97 (dd, *J*_1_ = 9.0 Hz, *J*_2_ = 2.9 Hz, 1H, Ar–H), 4.27 (s, 2H, CH_2_), 3.89 (d, *J* = 6.2 Hz, 2H, CH_2_), 3.85 (s, 2H, CH_2_), 3.13 (t, *J* = 11.7 Hz, 4H, 2 × CH_2_–piperidine), 2.91 (d, *J* = 11.7 Hz, 4H, 2 × CH_2_–piperidine), 2.07 (s, 2H, CH_2_–piperidine), 1.93 (s, 2H, CH_2_–piperidine), 1.76 (d, *J* = 13.7 Hz, 2H, CH_2_–piperidine), 1.45 (m, 3H, CH_2_–piperidine, CH–piperidine), signal for OH not seen in the spectrum; ^13^C NMR (101 MHz, chloroform-*d*): δ 176.2, 159.6, 157.3, 154.9, 133.0, 132.1, 130.8, 124.7, 123.2, 116.6, 114.7, 114.5, 79.5, 75.5, 72.3, 67.7, 60.6, 47.4, 36.1, 31.6, 28.9, 22.3; HRMS (ESI^+^) for C_25_H_32_Cl_2_N_2_O_3_ ([M + H]^+^): calcd, 479.18627; found, 480.18544; HPLC: *t*_R_ = 3.4 min (100% at 254 nm).

##### Synthesis of (4-Hydroxy-4-((*m*-tolyloxy)methyl)piperidin-1-yl)(4-(2-morpholinoethyl)phenyl)methanone (**58**)

It was synthesized according to general procedure A using compound **12** (0.338 mmol, 75 mg) and compound **31** (0.338 mmol, 85 mg) as reagents. Yield: 67.3%; white solid; ^1^H NMR (400 MHz, chloroform-*d*): δ 7.43–7.36 (m, 2H, 2 × Ar–H), 7.19 (t, *J* = 7.8 Hz, 1H, Ar–H), 6.96–6.89 (m, 1H, Ar–H), 6.86–6.78 (m, 1H, Ar–H), 6.78–6.68 (m, 2H, 2 × Ar–H), 4.51 (s, 1H, CH), 4.15 (t, *J* = 5.7 Hz, 2H, CH_2_), 3.82 (d, *J* = 3.1 Hz, 2H, CH_2_), 3.79–3.70 (m, 4H, 2 × CH_2_), 3.60–3.25 (m, 2H, CH_2_), 2.83 (t, *J* = 5.7 Hz, 2H, CH_2_), 2.64–2.51 (m, 4H, 2 × CH_2_), 2.35 (s, 3H, CH_3_), 1.79 (s, 4H, 2 × CH_2_); ^13^C NMR (101 MHz, DMSO-*d*_6_): δ 169.3, 159.7, 159.3, 139.4, 129.6, 129.2, 128.8, 121.8, 115.7, 115.3, 114.6, 112.0, 75.6, 68.7, 66.6, 65.9, 57.4, 54.1, 33.9, 21.6; HRMS (ESI^+^) for C_26_H_34_N_2_O_4_ ([M + H]^+^): calcd, 438.2519; found, 439.25691; HPLC: *t*_R_ = 3.43 min (95.4% at 254 nm).

##### Synthesis of (4-((3,4-Dichlorophenoxy)methyl)-4-hydroxypiperidin-1-yl)(4-(2-morpholinoethyl)phenyl)methanone (**59**)

It was synthesized according to general procedure A using compound **13** (0.271 mmol, 75 mg) and compound **31** (0.271 mmol, 68 mg) as reagents. Yield: 54.3%; white solid; ^1^H NMR (400 MHz, chloroform-*d*): δ 7.44–7.31 (m, 3H, 3 × Ar–H), 7.04 (d, *J* = 2.9 Hz, 1H, Ar–H), 6.97–6.91 (m, 2H, 2 × Ar–H), 6.79 (dd, *J*_1_ = 8.9 Hz, *J*_2_ = 2.9 Hz, 1H, Ar–H), 4.16 (t, *J* = 5.7 Hz, 2H, CH_2_), 3.82 (s, 2H, CH_2_), 3.80–3.73 (m, 4H, 2 × CH_2_–morpholine), 2.84 (t, *J* = 5.7 Hz, 2H, CH_2_), 2.62–2.59 (m, 5H, 5 × CH), 2.18 (s, 1H, CH), 1.80 (s, 4H, 2 × CH_2_–morpholine); ^13^C NMR (101 MHz, DMSO-*d*_6_): δ 169.3, 159.7, 158.8, 132.0, 131.4, 129.2, 128.8, 122.8, 117.0, 116.2, 114.6, 76.4, 68.7, 66.6, 65.9, 57.4, 54.1; HRMS (ESI^+^) for C_25_H_30_Cl_2_N_2_O_4_ ([M + H]^+^): calcd, 492.1583; found, 493.1561; HPLC: *t*_R_ = 3.870 min (99.4% at 254 nm).

##### Synthesis of *tert*-Butyl 4-(3,4-Dichlorobenzamido)piperidine-1-carboxylate (**60**)

It was synthesized according to general procedure A using *tert*-butyl 4-aminopiperidine-1-carboxylate (5.24 mmol, 1.05 g) and 3,4-dichlorobenzoic acid (5.24 mmol, 1.00 g) as reagents. The product was purified using flash column chromatography using dichloromethane/methanol (40:1) as eluent. Yield: 14.3%; white solid; ^1^H NMR (400 MHz, chloroform-*d*): δ 7.84 (d, *J* = 2.0 Hz, 1H, Ar–H), 7.58 (dd, *J*_1_ = 8.3 Hz, *J*_2_ = 2.1 Hz, 1H, Ar–H), 7.51 (d, *J* = 8.3 Hz, 1H, Ar–H), 5.94 (d, *J* = 7.6 Hz, 1H, CONH), 4.18–4.01 (m, 3H, CH–piperidine, CH_2_–piperidine), 2.90 (t, *J* = 12.3 Hz, 2H, CH_2_–piperidine), 2.08–1.96 (m, 2H, CH_2_–piperidine), 1.47 (s, 9H, 3 × CH_3_), 1.44–1.35 (m, 2H, CH_2_–piperidine); MS (ESI^–^) *m*/*z*: 371.1 ([M – H]^−^).

##### Synthesis of 3,4-Dichloro-*N*-(piperidin-4-yl)benzamide (**61**)

It was synthesized according to general procedure C using compound **60** (3.31 mmol, 1.235 g) as reagent. Yield: 55.2%; white crystals; ^1^H NMR (400 MHz, chloroform-*d*): δ 7.84 (d, *J* = 2.0 Hz, 1H, Ar–H), 7.58 (dd, *J*_1_ = 8.3 Hz, *J*_2_ = 2.1 Hz, 1H, Ar–H), 7.51 (d, *J* = 8.3 Hz, 1H, Ar–H), 5.92 (d, *J* = 7.0 Hz, 1H, CONH), 4.13–3.95 (m, 1H, CH–piperidine), 3.12 (dt, *J*_1_ = 12.4 Hz, *J*_2_ = 3.1 Hz, 2H, CH_2_–piperidine), 2.75 (td, *J*_1_ = 12.4 Hz, *J*_2_ = 2.5 Hz, 2H, CH_2_–piperidine), 2.03 (d, *J* = 9.4 Hz, 2H, CH_2_–piperidine), 1.41 (ddd, *J*_1_ = 23.8 Hz, *J*_2_ = 11.5 Hz, *J*_3_ = 4.0 Hz, 2H, CH_2_–piperidine); MS (ESI^+^) *m*/*z*: 273.0 ([M + H]^+^).

##### Synthesis of *tert*-Butyl 4-(4-((4-(3,4-Dichlorobenzamido)piperidin-1-yl)methyl)phenoxy)piperidine-1-carboxylate (**62**)

It was synthesized according to general procedure B using compound **61** (0.278 mmol, 76 mg) and compound **27** (0.278 mmol, 85 mg) as reagents. The crude product was purified using column flash chromatography using dichloromethane/methanol (20:1) as eluent. Yield: 74.7%; white solid; ^1^H NMR (400 MHz, chloroform-*d*): δ 7.83 (d, *J* = 2.0 Hz, 1H, Ar–H), 7.56 (dd, *J*_1_ = 8.4 Hz, *J*_2_ = 2.0 Hz, 1H, Ar–H), 7.50 (d, *J* = 8.4 Hz, 1H, Ar–H), 7.26–7.17 (m, 2H, 2 × Ar–H), 6.93–6.82 (m, 2H, 2 × Ar–H), 5.93 (s, 1H, CONH), 4.45 (tt, *J*_1_ = 7.1 Hz, *J*_2_ = 3.5 Hz, 1H, CH–piperidine), 3.97 (ddt, *J*_1_ = 15.2 Hz, *J*_2_ = 11.0 Hz, *J*_3_ = 5.6 Hz, 1H, CH–piperidine), 3.70 (ddd, *J*_1_ = 12.1 Hz, *J*_2_ = 7.4 Hz, *J*_3_ = 3.8 Hz, 2H, CH_2_–piperidine), 3.46 (s, 2H, CH_2_), 3.33 (ddd, *J*_1_ = 13.5 Hz, *J*_2_ = 7.7 Hz, *J*_2_ = 3.8 Hz, 2H, CH_2_–piperidine), 2.86 (d, *J* = 11.5 Hz, 2H, CH_2_–piperidine), 2.15 (t, *J* = 11.3 Hz, 2H, CH_2_–piperidine), 2.04–1.96 (m, 2H, CH_2_–piperidine), 1.96–1.86 (m, 2H, CH_2_–piperidine), 1.81–1.68 (m, 2H, CH_2_–piperidine), 1.63–1.49 (m, 2H, CH_2_–piperidine), 1.47 (s, 9H, 3 × CH_3_); MS (ESI^+^) *m*/*z*: 562.2 ([M + H]^+^).

##### Synthesis of *tert*-Butyl 4-((4-((4-(3,4-Dichlorobenzamido)piperidin-1-yl)methyl)phenoxy)methyl)piperidine-1-carboxylate (**63**)

It was synthesized according to general procedure B using compound **61** (0.313 mmol, 100 mg) and compound **24** (0.313 mmol, 85.5 mg) as reagents. The reaction mixture was stirred at 20 °C for 3 days. The crude product was purified using column flash chromatography using dichloromethane/methanol (20:1) as eluent. Yield: 91.9%; white solid; ^1^H NMR (400 MHz, chloroform-*d*): δ 7.92 (d, *J* = 2.0 Hz, 1H, Ar–H), 7.65 (dd, *J*_1_ = 8.4 Hz, *J*_2_ = 2.1 Hz, 1H, Ar–H), 7.46 (d, *J* = 8.4 Hz, 1H, Ar–H), 7.31 (d, *J* = 8.6 Hz, 2H, 2 × Ar–H), 7.13 (d, *J* = 8.1 Hz, 1H, CONH), 6.90 (d, *J* = 8.7 Hz, 2H, 2 × Ar–H), 4.27–4.08 (m, 3H, CH–piperidine, CH_2_–piperidine), 3.96 (s, 2H, CH_2_), 3.80 (d, *J* = 6.3 Hz, 2H, CH_2_), 3.36 (d, *J* = 12.0 Hz, 2H, CH_2_–piperidine), 2.81–2.59 (m, 4H, 2 × CH_2_–piperidine), 2.18–2.07 (m, 4H, 2 × CH_2_–piperidine), 2.05 (s, 2H, CH_2_), 1.99–1.89 (m, 1H, CH–piperidine), 1.81 (d, *J* = 12.7 Hz, 2H, CH_2_–piperidine), 1.46 (s, 9H, 3 × CH_3_), 1.32–1.20 (m, 2H, CH_2_–piperidine); MS (ESI^+^) *m*/*z*: 576.2 ([M-CH_3_COOH + H]^+^).

##### Synthesis of *tert*-Butyl 4-(4-(4-(3,4-Dichlorobenzamido)piperidine-1-carbonyl)phenoxy)piperidine-1-carboxylate (**64**)

It was synthesized according to general procedure A using compound **60** (0.36 mmol, 100 mg) and **29** (0.36 mmol, 118 mg) as reagents. Yield: 87.0%; white solid; ^1^H NMR (400 MHz, chloroform-*d*): δ 7.87 (d, *J* = 2.0 Hz, 1H, Ar–H), 7.60 (dd, *J*_1_ = 8.3 Hz, *J*_2_ = 2.1 Hz, 1H, Ar–H), 7.49 (d, *J* = 4.2 Hz, 1H, Ar–H), 7.37 (d, *J* = 1.9 Hz, 1H, Ar–H), 7.35 (d, *J* = 2.0 Hz, 1H, Ar–H), 6.91 (d, *J* = 1.9 Hz, 1H, Ar–H), 6.89 (d, *J* = 1.9 Hz, 1H, Ar–H), 6.26 (d, *J* = 7.8 Hz, 1H, CONH), 4.53–4.48 (m, 1H, CH–piperidine), 4.26–4.19 (m, 1H, CH–piperidine), 3.71–3.66 (m, 2H, CH_2_–piperidine), 3.38–3.33 (m, 2H, CH_2_–piperidine), 3.17–2.98 (m, 2H, CH_2_–piperidine), 2.12–2.06 (m, 2H, CH_2_–piperidine), 1.95–1.89 (m, 2H, CH_2_–piperidine), 1.78–1.72 (m, 2H, CH_2_–piperidine), 1.57–1.48 (m, 2H, CH_2_–piperidine), 1.47 (s, 9H, 3 × CH_3_), 1.45–1.27 (m, 2H, CH_2_–piperidine); MS (ESI^+^) *m*/*z*: 597.9 ([M + Na]^+^).

##### Synthesis of *tert*-Butyl 4-((4-(4-(3,4-Dichlorobenzamido)piperidine-1-carbonyl)phenoxy)methyl)piperidine-1-carboxylate (**65**)

It was synthesized according to general procedure A using compound **60** (0.36 mmol, 100 mg) and **26** (0.36 mmol, 123 mg) as reagents. Yield: 80.0%; white solid; ^1^H NMR (400 MHz, chloroform-*d*): δ 7.88 (d, *J* = 2.0 Hz, 1H, Ar–H), 7.60 (dd, *J*_1_ = 8.4 Hz, *J*_2_ = 2.1 Hz, 1H, Ar–H), 7.48 (d, *J* = 4.1 Hz, 1H, Ar–H), 7.37 (d, *J* = 1.9 Hz, 1H, Ar–H), 7.35 (d, *J* = 1.9 Hz, 1H, Ar–H), 6.88 (d, *J* = 1.9 Hz, 1H, Ar–H), 6.87 (d, *J* = 1.9 Hz, 1H, Ar–H), 6.33 (d, *J* = 7.8 Hz, 1H, CONH), 4.98–4.42 (m, 1H, CH–piperidine), 4.28–4.09 (m, 4H, 2 × CH_2_–piperidine), 3.81 (d, *J* = 6.3 Hz, 2H, CH_2_), 3.20–2.95 (m, 2H, CH_2_–piperidine), 2.74 (s, 2H, CH_2_–piperidine), 2.11–2.06 (m, 2H, CH_2_–piperidine), 1.99–1.92 (m, 1H, CH–piperidine), 1.84–1.78 (m, 2H, CH_2_–piperidine), 1.60–1.47 (m, 2H, CH_2_–piperidine), 1.46 (s, 9H, 3 × CH_3_), 1.36–1.26 (m, 2H, CH_2_–piperidine); MS (ESI^+^) *m*/*z*: 611.8 ([M + Na]^+^).

##### Synthesis of 3,4-Dichloro-*N*-(1-(4-(piperidin-4-yloxy)benzyl)piperidin-4-yl)benzamide (**66**)

It was synthesized according to general procedure C using compound **62** (0.165 mmol, 93 mg) as reagent. Yield: 68.0%; light brown solid; ^1^H NMR (400 MHz, chloroform-*d*): δ 7.83 (d, *J* = 2.0 Hz, 1H, Ar–H), 7.56 (dd, *J*_1_ = 8.3 Hz, *J*_2_ = 2.0 Hz, 1H, Ar–H), 7.50 (d, *J* = 8.3 Hz, 1H, Ar–H), 7.20 (d, *J* = 8.6 Hz, 2H, 2 × Ar–H), 6.86 (d, *J* = 8.6 Hz, 2H, 2 × Ar–H), 5.86 (d, *J* = 7.3 Hz, 1H, CONH), 4.38–4.31 (m, 1H, CH–piperidine), 4.02–3.92 (m, 1H, CH–piperidine), 3.45 (s, 2H, CH_2_), 3.18–3.11 (m, 2H, CH_2_–piperidine), 2.85 (d, *J* = 11.5 Hz, 2H, CH_2_–piperidine), 2.76–2.68 (m, 2H, CH_2_–piperidine), 2.15 (t, *J* = 10.9 Hz, 2H, CH_2_–piperidine), 2.01 (d, *J* = 13.1 Hz, 4H, 2 × CH_2_–piperidine), 1.70–1.61 (m, 2H, CH_2_–piperidine), 1.25 (s, 1H, CH–piperidine), 0.91–0.79 (m, 1H, CH–piperidine), signal for NH not seen in the spectrum; ^13^C NMR (101 MHz, chloroform-*d*): δ 164.7, 156.5, 135.7, 134.6, 132.9, 130.5, 130.4, 130.3, 129.2, 126.1, 115.8, 73.4, 62.4, 52.1, 47.6, 44.0, 32.5, 32.2; HRMS (ESI^+^) for C_24_H_30_Cl_2_N_3_O_2_ ([M + H]^+^): calcd, 462.17096; found 462.17041; HPLC: *t*_R_ = 1.450 min (96.2% at 254 nm).

##### Synthesis of 3,4-Dichloro-*N*-(1-(4-(piperidin-4-yloxy)benzyl)piperidin-4-yl)benzamide (**67**)

It was synthesized according to general procedure C using compound **63** (0.149 mmol, 86 mg) as reagent. Yield: 70.4%, light brown solid; ^1^H NMR (400 MHz, chloroform-*d*): δ 7.78 (d, *J* = 2.4 Hz, 1H, Ar–H), 7.39–7.28 (m, 2H, 2 Ar–H), 7.21 (d, *J* = 8.4 Hz, 2H, 2 × Ar–H), 7.14 (s, 1H, NHCO), 6.84 (d, *J* = 8.5 Hz, 2H, 2 × Ar–H), 3.78 (d, *J* = 6.4 Hz, 2H, CH_2_), 3.45 (s, 2H, CH_2_), 3.12 (d, *J* = 12.2 Hz, 2H, CH_2_–piperidine), 2.96 (d, *J* = 11.4 Hz, 3H, CH–piperidine, CH_2_–piperidine), 2.65 (td, *J* = 12.3, 2.5 Hz, 2H, CH_2_–piperidine), 2.22 (dt, *J*_1_ = 11.1 Hz, *J*_2_ = 6.0 Hz, 1H, CH–piperidine), 2.06–1.94 (m, 2H, CH_2_–piperidine), 1.91–1.75 (m, 4H, 2 × CH_2_–piperidine), 1.27 (qd, *J*_1_ = 12.2 Hz, *J*_2_ = 4.4 Hz, 4H, 2 × CH_2_–piperidine), signal for NH not seen in the spectrum; ^13^C NMR (101 MHz, chloroform-*d*): δ 164.6, 158.3, 135.8, 134.6, 133.0, 130.6, 130.3, 130.1, 129.1, 126.1, 114.1, 73.0, 62.4, 52.1, 47.5, 46.3, 36.4, 32.2, 30.2; HRMS (ESI^+^) for C_25_H_32_Cl_2_N_3_O_2_ ([M + H]^+^): calcd, 476.18661; found, 476.18632 HPLC: *t*_R_ = 2.450 min (95.1% at 254 nm).

##### Synthesis of 3,4-Dichloro-*N*-(1-(4-(piperidin-4-yloxy)benzoyl)piperidin-4-yl)benzamide (**68**)

It was synthesized according to general procedure C using compound **64** (0.264 mmol, 152 mg) as reagent. Yield: 100%; yellow oil; ^1^H NMR (400 MHz, DMSO-*d*_6_): δ 8.81 (s, 1H, NH), 8.56 (s, 1H, NH), 8.55 (d, *J* = 7.6 Hz, 1H, Ar–H), 8.10 (d, *J* = 2.0 Hz, 1H, Ar–H), 7.83 (dd, *J*_1_ = 8.4 Hz, *J*_2_ = 2.0 Hz, 1H, Ar–H), 7.76 (d, *J* = 8.4 Hz, 1H, CONH), 7.35 (d, *J* = 8.7 Hz, 2H, 2 × Ar–H), 7.06 (d, *J* = 8.7 Hz, 2H, 2 × Ar–H), 4.75–4.68 (m, 1H, CH–piperidine), 4.10–4.02 (m, 1H, CH–piperidine), 3.30–3.17 (m, 4H, 2 × CH_2_–piperidine), 3.16–2.97 (m, 4H, 2 × CH_2_–piperidine), 2.15–2.08 (m, 2H, CH_2_–piperidine), 1.89–1.77 (m, 4H, 2 × CH_2_–piperidine), 1.59–1.37 (m, 2H, CH_2_–piperidine), signal for NH not seen in the spectrum; ^13^C NMR (101 MHz, DMSO-*d*_6_): δ 169.4, 163.7, 158.0, 135.2, 134.4, 131.6, 131.1, 129.7, 129.3, 129.0, 128.2, 115.8, 69.6, 47.3, 40.9, 31.9, 27.5; HRMS (ESI^+^) for C_24_H_28_Cl_2_N_3_O_3_ ([M + H]^+^): calcd, 476.15022; found, 476.14964; HPLC: *t*_R_ = 3.710 min (98.5% at 254 nm).

##### Synthesis of 3,4-Dichloro-*N*-(1-(4-(piperidin-4-ylmethoxy)benzoyl)piperidin-4-yl)benzamide (**69**)

It was synthesized according to general procedure C using compound **65** (0.264 mmol, 152 mg) as reagent. Yield: 100%; yellow oil; ^1^H NMR (400 MHz, DMSO-*d*_6_):: δ 8.87 (s, 1H, NH), 8.58 (s, 1H, NH), 8.57 (d, *J* = 7.6 Hz, 1H, Ar–H), 8.10 (d, *J* = 1.9 Hz, 1H, Ar–H), 7.84 (dd, *J*_1_ = 8.4 Hz, *J*_2_ = 1.9 Hz, 1H, Ar–H), 7.75 (d, *J* = 8.4 Hz, 1H, CONH), 7.34 (d, *J* = 8.6 Hz, 2H, 2 × Ar–H), 6.99 (d, *J* = 8.6 Hz, 2H, 2 × Ar–H), 4.56–4.17 (m, 1H, CH–piperidine), 4.17–3.97 (m, 2H, CH_2_–piperidine), 3.90 (d, *J* = 6.2 Hz, 2H, CH_2_), 3.79–3.47 (m, 2H, CH_2_–piperidine), 3.19–2.96 (m, 2H, CH_2_–piperidine), 2.89 (t, *J* = 11.8 Hz, 2H, CH_2_–piperidine), 2.11–2.04 (m, 1H, CH–piperidine), 1.98–1.91 (m, 2H, CH_2_–piperidine), 1.88–1.75 (m, 2H, CH_2_–piperidine), 1.56–1.43 (m, 4H, 2 × CH_2_–piperidine), signal for NH not seen in the spectrum; ^13^C NMR (101 MHz, DMSO-*d*_6_): δ 169.5, 163.7, 159.9, 135.2, 134.4, 131.6, 131.1, 129.7, 129.2, 128.7, 128.2, 114.7, 71.7, 47.3, 43.1, 33.5, 31.9, 25.6; HRMS (ESI^+^) for C_25_H_30_Cl_2_N_3_O_3_ ([M + H]^+^): calcd, 490.16697; found, 490.16492; HPLC: *t*_R_ = 3.84 min (98.9% at 254 nm).

##### Synthesis of *tert*-Butyl 4-((3-Chlorophenyl)carbamoyl)piperidine-1-carboxylate (**70**)

It was synthesized according to general procedure A using 1-(*tert*-butoxycarbonyl)piperidine-4-carboxylic acid (15.68 mmol, 3.59 g) and 3-chloroaniline (15.68 mmol, 1.65 mL) as reagents. The crude product was purified using flash column chromatography using dichloromethane/methanol (9:1) as eluent. Yield: 35.9%; light pink solid; ^1^H NMR (400 MHz, chloroform-*d*): δ 7.65 (s, 1H, NHCO), 7.36–7.29 (m, 2H, 2 × Ar–H), 7.23 (t, *J* = 8.1 Hz, 1H, Ar–H), 7.08 (ddd, *J*_1_ = 8.0 Hz, *J*_2_ = 1.9 Hz, *J*_3_ = 0.9 Hz, 1H, Ar–H), 4.17 (s, 2H, CH_2_–piperidine), 2.79 (t, *J* = 11.8 Hz, 2H, CH_2_–piperidine), 2.38 (tt, *J*_1_ = 11.4 Hz, *J*_2_ = 3.8 Hz, 1H, CH–piperidine), 1.89 (d, *J* = 10.9 Hz, 2H, CH_2_–piperidine), 1.79–1.68 (m, 2H, CH_2_–piperidine), 1.47 (s, 9H, 3 × CH_3_); MS (ESI^–^) *m*/*z*: 337.1 ([M – H]^−^).

##### Synthesis of *tert*-Butyl 4-((3,4-Dichlorophenyl)carbamoyl)piperidine-1-carboxylate (**71**)

It was synthesized according to general procedure A using 1-(*tert*-butoxycarbonyl)piperidine-4-carboxylic acid (6.17 mmol, 1.42 g) and 3,4-dichloroaniline (6.17 mmol, 1.00 g) as reagents. The crude product was purified using flash column chromatography using dichloromethane/methanol (40:1) as eluent. Yield: 14.3%; white solid; ^1^H NMR (400 MHz, chloroform-*d*): δ 7.78 (d, *J* = 2.3 Hz, 1H, NHCO), 7.37 (d, *J* = 8.7 Hz, 1H, Ar–H), 7.32 (dd, *J*_1_ = 8.7 Hz, *J*_2_ = 2.4 Hz, 1H, Ar–H), 7.18 (s, 1H, Ar–H), 4.18 (s, 2H, CH_2_–piperidine), 2.79 (t, *J* = 11.8 Hz, 2H, CH_2_–piperidine), 2.37 (tt, *J*_1_ = 11.5 Hz, *J*_2_ = 3.8 Hz, 1H, CH–piperidine), 1.89 (d, *J* = 11.3 Hz, 2H, CH_2_–piperidine), 1.73 (ddd, *J*_1_ = 25.1 Hz, *J*_2_ = 12.0, *J*_3_ = 4.3 Hz, 2H, CH_2_–piperidine), 1.47 (s, 9H, 3 × CH_3_); ^13^C NMR (101 MHz, chloroform-*d*): δ 172.9, 154.7, 137.4, 132.7, 130.5, 127.5, 121.6, 119.1, 80.0, 44.2, 42.7, 28.5, 28.5; MS (ESI^–^) *m*/*z*: 371.1 ([M – H]^−^).

##### Synthesis of *tert*-Butyl 4-((4-Chlorophenyl)carbamoyl)piperidine-1-carboxylate (**72**)

It was synthesized according to general procedure A using 1-(*tert*-butoxycarbonyl)piperidine-4-carboxylic acid (15.68 mmol, 3.59 g) and 4-chloroaniline (15.68 mmol, 2.00 g) as reagents. The crude product was purified using flash column chromatography using dichloromethane and ethyl acetate as eluents. Yield: 59.8%; white solid; ^1^H NMR (400 MHz, chloroform-*d*): δ 7.47 (d, *J* = 8.8 Hz, 2H, 2 × Ar–H), 7.28 (d, *J* = 8.8 Hz, 2H, 2 × Ar–H), 7.13 (s, 1H, NH–CO), 4.27–4.13 (m, 2H, CH_2_–piperidine), 2.86–2.73 (m, 2H, CH_2_–piperidine), 2.37 (tt, *J*_1_ = 11.5 Hz, *J*_2_ = 3.8 Hz, 1H, CH–piperidine), 1.90 (d, *J* = 13.2 Hz, 2H, CH_2_–piperidine), 1.74 (ddd, *J*_1_ = 25.1 Hz, *J*_2_ = 12.0 Hz, *J*_3_ = 4.3 Hz, 2H, CH_2_–piperidine), 1.47 (s, 9H, 3 × CH_3_); MS (ESI^–^) *m*/*z*: 337.1 ([M – H]^−^).

##### Synthesis of *tert*-Butyl 4-((4-Fluorophenyl)carbamoyl)piperidine-1-carboxylate (**73**)

It was synthesized according to general procedure A using 1-(*tert*-butoxycarbonyl)piperidine-4-carboxylic acid (13.5 mmol, 3.00 g) and 4-fluoroaniline (13.5 mmol, 1.3 mL) as reagents. The product was crystallized from hexane. Yield: 65.9%; white crystals; ^1^H NMR (400 MHz, chloroform-*d*): δ 7.49–7.44 (m, 2H, 2 × Ar–H), 7.11 (s, 1H, NHCO), 7.05–6.98 (m, 2H, 2 × Ar–H), 4.25–4.11 (m, 2H, CH_2_–piperidine), 2.85–2.73 (m, 2H, CH_2_–piperidine), 2.37 (tt, *J*_1_ = 11.4 Hz, *J*_2_ = 3.8 Hz, 1H, CH–piperidine), 1.90 (d, *J* = 11.9 Hz, 2H, CH_2_–piperidine), 1.74 (ddd, *J*_1_ = 25.0 Hz, *J*_2_ = 11.9 Hz, *J*_3_ = 4.3 Hz, 2H, CH_2_–piperidine), 1.47 (s, 9H, 3 × CH_3_); MS (ESI^–^) *m*/*z*: 321.3 ([M – H]^−^).

##### Synthesis of *tert*-Butyl 4-((4-Bromophenyl)carbamoyl)piperidine-1-carboxylate (**74**)

It was synthesized according to general procedure A using 1-(*tert*-butoxycarbonyl)piperidine-4-carboxylic acid (8.72 mmol, 2.00 g) and 4-bromoaniline (8.72 mmol, 1.50 g) as reagents. The product was crystallized from ethyl acetate/hexane. Yield: 17.5%, white crystals; ^1^H NMR (400 MHz, chloroform-*d*): δ 7.46–7.39 (m, 4H, 4 × Ar–H), 7.11 (s, 1H, NHCO), 4.26–4.14 (m, 2H, CH_2_–piperidine), 2.84–2.74 (m, 2H, CH_2_–piperidine), 2.37 (tt, *J*_1_ = 11.1 Hz, *J*_2_ = 3.8 Hz, 1H, CH–piperidine), 1.90 (d, *J* = 13.9 Hz, 2H, CH_2_–piperidine), 1.73 (ddd, *J*_1_ = 25.3 Hz, *J*_2_ = 12.2 Hz, *J*_3_ = 4.5 Hz, 2H, CH_2_–piperidine), 1.47 (s, 9H, 3 × CH_3_); MS (ESI^–^) *m*/*z*: 381.2 ([M – H]^−^).

##### Synthesis of *N*-(3-Chlorophenyl)piperidine-4-carboxamide (**75**)

It was synthesized according to general procedure C using compound **70** (5.28 mmol, 1.79 g) as reagent. Yield: 28.6%; white solid; ^1^H NMR (400 MHz, chloroform-*d*): δ 7.67 (s, 1H, NHCO), 7.35 (dd, *J*_1_ = 8.2 Hz, *J*_2_ = 1.1 Hz, 1H, Ar–H), 7.29 (s, 1H, Ar–H), 7.22 (t, *J* = 8.1 Hz, 1H, Ar–H), 7.07 (ddd, *J*_1_ = 8.0 Hz, *J*_2_ = 1.9 Hz, *J*_3_ = 0.9 Hz, 1H, Ar–H), 3.18 (dt, *J*_1_ = 12.4 Hz, *J*_2_ = 3.3 Hz, 2H, CH_2_–piperidine), 2.67 (td, *J*_1_ = 12.3 Hz, *J*_2_ = 2.6 Hz, 2H, CH_2_–piperidine), 2.42–2.31 (m, 1H, CH–piperidine), 1.90 (d, *J* = 12.6 Hz, 2H, CH_2_–piperidine), 1.75 (dd, *J*_1_ = 11.9 Hz, *J*_2_ = 4.0 Hz, 2H, CH_2_–piperidine), signal for NH not seen in the spectrum; MS (ESI+) *m*/*z*: 239.1 ([M + H]^+^).

##### Synthesis of *N*-(3,4-Dichlorophenyl)piperidine-4-carboxamide (**76**)

It was synthesized according to general procedure C using compound **71** (0.667 mmol, 249 mg) as reagent. Yield: 8.6%; off-white solid; ^1^H NMR (400 MHz, chloroform-*d*): δ 7.79 (s, 2H, Ar–H, NHCO), 7.44–7.29 (m, 2H, 2 × Ar–H), 3.18 (d, *J* = 12.4 Hz, 2H, CH_2_–piperidine), 2.72–2.57 (m, 2H, CH_2_–piperidine), 2.39 (tt, *J*_1_ = 11.6 Hz, *J*_2_ = 3.7 Hz, 1H, CH–piperidine), 1.98 (s, 1H, NH), 1.88 (d, *J* = 12.3 Hz, 2H, CH_2_–piperidine), 1.71 (qd, *J*_1_ = 12.3 Hz, *J*_2_ = 3.9 Hz, 2H, CH_2_–piperidine); MS (ESI^+^) *m*/*z*: 272.9 ([M + H]^+^).

##### Synthesis of *N*-(4-Chlorophenyl)piperidine-4-carboxamide (**77**)

It was synthesized according to general procedure C using compound **72** (9.22 mmol, 3.124 g) as reagent. Yield: 34.3%; white solid; ^1^H NMR (400 MHz, chloroform-*d*): δ 7.47 (d, *J* = 8.8 Hz, 2H, 2 × Ar–H), 7.28 (d, *J* = 8.9 Hz, 2H, 2 × Ar–H), 7.17 (s, 1H, NHCO), 3.19 (dt, *J*_1_ = 12.4 Hz, *J*_2_ = 3.2 Hz, 2H, CH_2_–piperidine), 2.67 (td, *J*_1_ = 12.3 Hz, *J*_2_ = 2.5 Hz, 2H, CH_2_–piperidine), 2.36 (tt, *J*_1_ = 11.7 Hz, *J*_2_ = 3.8 Hz, 1H, CH–piperidine), 1.91 (d, *J* = 12.5 Hz, 2H, CH_2_–piperidine), 1.71 (ddd, *J*_1_ = 25.0 Hz, *J*_2_ = 12.0 Hz, *J*_3_ = 4.0 Hz, 2H, CH_2_–piperidine), signal for NH not seen in the spectrum; MS (ESI^+^) *m*/*z*: 239.0 ([M + H]^+^).

##### Synthesis of *N*-(4-Fluorophenyl)piperidine-4-carboxamide (**78**)

It was synthesized according to general procedure C using compound **73** (8.69 mmol, 2.80 g) as reagent. Yield: 83.2%; white solid; ^1^H NMR (400 MHz, chloroform-*d*): δ 7.51–7.44 (m, 2H, 2 × Ar–H), 7.12 (s, 1H, NHCO), 7.05–6.98 (m, 2H, 2 × Ar–H), 3.22–3.15 (m, 2H, CH_2_–piperidine), 2.67 (td, *J*_1_ = 12.3 Hz, *J*_2_ = 2.5 Hz, 2H, CH_2_–piperidine), 2.36 (tt, *J*_1_ = 11.8 Hz, *J*_2_ = 3.8 Hz, 1H, CH–piperidine), 1.91 (d, *J* = 13.0 Hz, 2H, CH_2_–piperidine), 1.71 (ddd, *J*_1_ = 25.0 Hz, *J*_2_ = 11.9 Hz, *J*_3_ = 4.0 Hz, 2H, CH_2_–piperidine), signal for NH not seen in the spectrum; MS (ESI^+^) *m*/*z*: 223.1 ([M + H]^+^).

##### Synthesis of *N*-(4-Bromophenyl)piperidine-4-carboxamide (**79**)

It was synthesized according to general procedure C using compound **74** (1.47 mmol; 565 mg) as reagent. Yield: 73.1%; brown solid; ^1^H NMR (400 MHz, chloroform-*d*): δ 7.43 (s, 4H, 4 × Ar–H), 7.13 (s, 1H, NHCO), 3.22–3.15 (m, 2H, CH_2_–piperidine), 2.67 (td, *J*_1_ = 12.3 Hz, *J*_2_ = 2.5 Hz, 2H, CH_2_–piperidine), 2.36 (tt, *J*_1_ = 11.7 Hz, *J*_2_ = 3.9 Hz, 1H, CH–piperidine), 1.91 (d, *J* = 12.9 Hz, 2H, CH_2_–piperidine), 1.71 (ddd, *J*_1_ = 16.1 Hz, *J*_2_ = 12.5 Hz, *J*_3_ = 4.1 Hz, 2H, CH_2_–piperidine), signal for NH not seen in the spectrum; MS (ESI^+^) *m*/*z*: 283.0 ([M + H]^+^).

##### Synthesis of *tert*-Butyl 4-((4-((4-((3-Chlorophenyl)carbamoyl)piperidin-1-yl)methyl)phenoxy)methyl)piperidine-1-carboxylate (**80**)

It was synthesized according to general procedure B using compound **24** (0.313 mmol, 100 mg) and compound **75** (0.313 mmol, 74.74 mg) as reagents. The reaction mixture was stirred for 2 days. The crude product was purified using flash column chromatography using dichloromethane/methanol (20:1) as eluent. Yield: 58.3%; white solid; ^1^H NMR (400 MHz, chloroform-*d*): δ 7.68 (s, 1H, Ar–H), 7.60–7.38 (m, 1H, NHCO), 7.35–7.30 (m, 1H, Ar–H), 7.26–7.19 (m, 3H, 3 × Ar–H), 7.10–7.05 (m, 1H, Ar–H), 6.86 (d, *J* = 8.6 Hz, 2H, 2 × Ar–H), 4.22–4.10 (m, 2H, CH_2_–piperidine), 3.80 (d, *J* = 6.3 Hz, 2H, CH_2_), 3.61 (s, 2H, CH_2_), 3.14–3.03 (m, 2H, CH_2_–piperidine), 2.75 (t, *J* = 12.3 Hz, 2H, CH_2_–piperidine), 2.47–2.18 (m, 2H, CH_2_–piperidine), 2.01–1.91 (m, 5H, CH–piperidine, 2 × CH_2_–piperidine), 1.82 (d, *J* = 12.5 Hz, 3H, CH–piperidine, CH_2_–piperidine), 1.46 (s, 9H, 3 × CH_3_), 1.33–1.20 (m, 2H, CH_2_–piperidine); MS (ESI^+^) *m*/*z*: 542.2 ([M + H]^+^).

##### Synthesis of *tert*-Butyl 4-((4-((4-((3,4-Dichlorophenyl)carbamoyl)piperidin-1-yl)methyl)phenoxy)methyl)piperidine-1-carboxylate (**81**)

It was synthesized according to general procedure B using compound **24** (0.313 mmol, 100 mg) and compound **76** (0.313 mmol, 85.52 mg) as reagents. The reaction mixture was stirred for 3 days. The crude product was purified using flash column chromatography using dichloromethane/methanol (20:1) as eluent. Yield: 58.7%; white solid; ^1^H NMR (400 MHz, DMSO-*d*_6_): δ 10.13 (s, 1H, NHCO), 8.01 (d, *J* = 2.4 Hz, 1H, Ar–H), 7.54 (d, *J* = 8.8 Hz, 1H, Ar–H), 7.48 (dd, *J*_1_ = 8.8 Hz, *J*_2_ = 2.4 Hz, 1H, Ar–H), 7.19 (d, *J* = 8.4 Hz, 2H, 2 × Ar–H), 6.87 (d, *J* = 8.4 Hz, 2H, 2 × Ar–H), 3.97 (d, *J* = 12.1 Hz, 2H, CH_2_–piperidine), 3.81 (d, *J* = 6.4 Hz, 2H, CH_2_), 3.37 (s, 2H, CH_2_), 2.87–2.70 (m, 4H, 2 × CH_2_–piperidine), 2.30–2.20 (m, 1H, CH–piperidine), 1.96–1.84 (m, 3H, CH–piperidine, CH_2_–piperidine), 1.74 (d, *J* = 12.2 Hz, 4H, 2 × CH_2_–piperidine), 1.67–1.56 (m, 2H, CH_2_–piperidine), 1.40 (s, 9H, 3 × CH_3_), 1.20–1.08 (m, 2H, CH_2_–piperidine); MS (ESI^–^) *m*/*z*: 574.3 ([M – H]^−^).

##### Synthesis of *tert*-Butyl 4-((4-((4-((4-Chlorophenyl)carbamoyl)piperidin-1-yl)methyl)phenoxy)methyl)piperidine-1-carboxylate (**82**)

It was synthesized according to general procedure B using compound **24** (0.313 mmol, 100 mg) and compound **77** (0.313 mmol, 74.74 mg) as reagents. The reaction mixture was stirred for 2 days. The crude product was purified using flash column chromatography using dichloromethane/methanol (20:1) as eluent. Yield: 64.2%; white solid; ^1^H NMR (400 MHz, chloroform-*d*): δ 7.47 (d, *J* = 8.7 Hz, 2H, 2 × Ar–H), 7.29–7.27 (m, 2H, 2 × Ar–H), 7.26–7.23 (m, 2H, 2 × Ar–H), 6.87 (d, *J* = 8.5 Hz, 2H, 2 × Ar–H), 4.21–4.10 (m, 2H, CH_2_–piperidine), 3.80 (d, *J* = 6.3 Hz, 2H, CH_2_), 3.64 (s, 2H, CH_2_), 3.17–3.07 (m, 2H, CH_2_–piperidine), 2.80–2.69 (m, 2H, CH_2_–piperidine), 2.48–2.31 (m, 2H, CH_2_–piperidine), 2.06–1.92 (m, 5H, CH–piperidine, 2 × CH_2_–piperidine), 1.82 (d, *J* = 14.4 Hz, 2H, CH_2_–piperidine), 1.46 (s, 9H, 3 × CH_3_), 1.33–1.20 (m, 3H, CH–piperidine, CH_2_–piperidine), signal for CONH not seen in the spectrum; MS (ESI^+^) *m*/*z*: 542.2 ([M + H]^+^).

##### Synthesis of *tert*-Butyl 4-((4-((4-((4-Fluorophenyl)carbamoyl)piperidin-1-yl)methyl)phenoxy)methyl)piperidine-1-carboxylate (**83**)

It was synthesized according to general procedure B using compound **24** (0.241 mmol, 77 mg) and compound **78** (0.241 mmol, 53.5 mg) as reagents. The reaction mixture was stirred for 2 days. The crude product was purified using flash column chromatography using dichloromethane/methanol (20:1) as eluent. Yield: 52.1%; white solid; ^1^H NMR (400 MHz, chloroform-*d*): δ 7.50–7.45 (m, 2H, 2 × Ar–H), 7.28 (s, 2H, 2 × Ar–H), 7.03–6.97 (m, 2H, 2 × Ar–H), 6.88 (d, *J* = 8.6 Hz, 2H, 2 × Ar–H), 4.21–4.09 (m, 2H, CH_2_–piperidine), 3.80 (d, *J* = 6.4 Hz, 2H, CH_2_), 3.70 (s, 2H, CH_2_), 3.21–3.12 (m, 2H, CH_2_–piperidine), 2.80–2.69 (m, 2H, CH_2_–piperidine), 2.52–2.36 (m, 2H, CH_2_–piperidine), 2.11–1.94 (m, 5H, CH–piperidine, 2 × CH_2_–piperidine), 1.82 (d, *J* = 12.7 Hz, 2H, CH_2_–piperidine), 1.46 (s, 9H, 3 × CH_3_), 1.33–1.21 (m, 3H, CH–piperidine, CH_2_–piperidine), signal for NHCO not seen in the spectrum; MS (ESI^+^) *m*/*z*: 526.3 ([M + H]^+^).

##### Synthesis of *tert*-Butyl 4-((4-((4-((4-Bromophenyl)carbamoyl)piperidin-1-yl)methyl)phenoxy)methyl)piperidine-1-carboxylate (**84**)

It was synthesized according to general procedure B using compound **24** (0.313 mmol, 100 mg) and compound **73** (0.313 mmol, 88.6 mg) as reagents. The reaction mixture was stirred for 2 days. The crude product was purified using flash column chromatography using dichloromethane/methanol (20:1) as eluent. Yield: 73.1%; white solid; ^1^H NMR (400 MHz, chloroform-*d*): δ 7.88 (s, 1H, NHCO), 7.42 (q, *J* = 9.0 Hz, 4H, 4 × Ar–H), 7.29 (s, 2H, 2 × Ar–H), 6.88 (d, *J* = 8.6 Hz, 2H, 2 × Ar–H), 4.27–4.06 (m, 2H, CH_2_–piperidine), 3.80 (d, *J* = 6.3 Hz, 2H, CH_2_), 3.75 (s, 2H, CH_2_), 3.25–3.14 (m, 2H, CH_2_–piperidine), 2.81–2.69 (m, 2H, CH_2_–piperidine), 2.56 (s, 3H, CH–piperidine, CH_2_–piperidine), 2.10–2.01 (m, 5H, CH–piperidine, 2 × CH_2_–piperidine), 1.82 (d, *J* = 12.5 Hz, 2H, CH_2_–piperidine), 1.46 (s, 9H, 3 × CH_3_), 1.33–1.20 (m, 2H, CH_2_–piperidine); MS (ESI^+^) *m*/*z*: 586.2 ([M + H]^+^).

##### Synthesis of *tert*-Butyl 4-(4-((4-((3-Chlorophenyl)carbamoyl)piperidin-1-yl)methyl)phenoxy)piperidine-1-carboxylate (**85**)

It was synthesized according to general procedure B using compound **27** (0.294 mmol, 90 mg) and compound **75** (0.294 mmol, 70.35 mmol) as reagents. The reaction mixture was stirred for 2 days. The crude product was purified using flash column chromatography using dichloromethane/methanol (20:1) as eluent. Yield: 53.97%; white solid; ^1^H NMR (400 MHz, chloroform-*d*): δ 7.67 (s, 1H, NHCO), 7.46–7.29 (m, 2H, 2 × Ar–H), 7.26–7.18 (m, 3H, 3 × Ar–H), 7.11–7.03 (m, 1H, Ar–H), 6.88 (d, *J* = 8.6 Hz, 2H, 2 × Ar–H), 4.50–4.41 (m, 1H, CH–piperidine), 3.74–3.65 (m, 2H, CH_2_–piperidine), 3.55 (s, 2H, CH_2_), 3.39–3.28 (m, 2H, CH_2_–piperidine), 3.11–2.95 (m, 2H, CH_2_–piperidine), 2.40–2.08 (m, 3H, CH–piperidine, CH_2_–piperidine), 1.97–1.88 (m, 5H, CH–piperidine, 2 × CH_2_–piperidine), 1.79–1.70 (m, 3H, CH–piperidine, CH_2_–piperidine), 1.47 (s, 9H, 3 × CH_3_); MS (ESI^+^) *m*/*z*: 528.2 ([M + H]^+^).

##### Synthesis of *tert*-Butyl 4-(4-((4-((3,4-Dichlorophenyl)carbamoyl)piperidin-1-yl)methyl)phenoxy)piperidine-1-carboxylate (**86**)

It was synthesized according to general procedure B using compound **27** (0.183 mmol, 55.9 mg) and compound **76** (0.183 mmol, 50 mg) as reagents. The reaction mixture was stirred for 2 days. The crude product was purified using flash column chromatography using dichloromethane/methanol (20:1) as eluent. Yield: 32.5%; white solid; ^1^H NMR (400 MHz, chloroform-*d*): δ 9.29 (s, 1H, CONH), 7.83 (d, *J* = 2.4 Hz, 1H, Ar–H), 7.40–7.23 (m, 4H, 4 × Ar–H), 6.91 (d, *J* = 8.3 Hz, 2H, 2 × Ar–H), 4.48 (tt, *J*_1_ = 7.3 Hz, *J*_2_ = 3.5 Hz, 1H, CH–piperidine), 4.06 (s, 2H), 3.73–3.62 (m, 2H, CH_2_–piperidine), 3.48–3.27 (m, 3H, CH–piperidine, CH_2_–piperidine), 2.98 (s, 2H, CH_2_–piperidine), 2.81 (t, *J* = 7.2 Hz, 1H, CH–piperidine), 2.21–2.11 (m, 4H, 2 × CH_2_–piperidine), 1.91 (ddt, *J*_1_ = 11.6 Hz, *J*_2_ = 7.5 Hz, *J*_3_ = 3.7 Hz, 2H, CH_2_–piperidine), 1.73 (dtd, *J*_1_ = 13.5 Hz, *J*_2_ = 7.3 Hz, *J*_3_ = 3.7 Hz, 2H, CH_2_–piperidine), 1.47 (s, 9H, 3 × CH_3_); MS (ESI^+^) *m*/*z*: 562.2 ([M-CH_3_COOH + H]^+^).

##### Synthesis of *tert*-Butyl 4-(4-((4-((4-Chlorophenyl)carbamoyl)piperidin-1-yl)methyl)phenoxy)piperidine-1-carboxylate (**87**)

It was synthesized according to general procedure B using compound **27** (0.294 mmol, 90 mg) and compound **77** (0.294 mmol, 70.3 mg) as reagents. The reaction mixture was stirred for 4 days. The crude product was purified using flash column chromatography using dichloromethane/methanol (20:1) as eluent. Yield: 74.5%; white solid; ^1^H NMR (400 MHz, MeOD): δ 7.59–7.54 (m, 2H, 2 × Ar–H), 7.34–7.28 (m, 4H, 4 × Ar–H), 6.98 (d, *J* = 8.5 Hz, 2H, 2 × Ar–H), 3.77–3.66 (m, 4H, CH_2_, CH_2_–piperidine), 3.19–3.10 (m, 3H, CH–piperidine, CH_2_–piperidine), 2.49–2.28 (m, 4H, 2 × CH_2_–piperidine), 2.00–1.88 (m, 6H, 3 × CH_2_–piperidine), 1.74–1.63 (m, 3H, CH–piperidine, CH_2_–piperidine), 1.49 (s, 9H, 3 × CH_3_), signal for NHCO not seen in the spectrum; MS (ESI^+^) *m*/*z*: 528.2 ([M + H]^+^).

##### Synthesis of *N*-(3-Chlorophenyl)-1-(4-(piperidin-4-ylmethoxy)benzyl)piperidine-4-carboxamide (**88**)

It was synthesized according to general procedure C using compound **80** (0.142 mmol, 77 mg) as reagent. Yield: 79.6%; light brown solid; ^1^H NMR (400 MHz, chloroform-*d*): δ 7.66 (s, 1H, NHCO), 7.33 (d, *J* = 9.1 Hz, 1H, Ar–H), 7.22 (t, *J* = 8.1 Hz, 3H, 3 × Ar–H), 7.15 (s, 1H, Ar–H), 7.07 (d, *J* = 8.6 Hz, 1H, Ar–H), 6.84 (d, *J* = 8.5 Hz, 2H, 2 × Ar–H), 3.78 (d, *J* = 6.3 Hz, 2H, CH_2_), 3.45 (s, 2H, CH_2_), 3.15 (d, *J* = 12.2 Hz, 2H, CH_2_–piperidine), 2.96 (d, *J* = 11.5 Hz, 2H, CH_2_–piperidine), 2.67 (td, *J*_1_ = 12.2 Hz, *J*_2_ = 2.5 Hz, 2H, CH_2_–piperidine), 2.27–2.18 (m, 1H, CH–piperidine), 2.05–1.95 (m, 3H, CH–piperidine, CH_2_–piperidine), 1.93–1.85 (m, 5H, CH–piperidine, 2 × CH_2_–piperidine), 1.83 (s, 1H, NH), 1.36–1.22 (m, 3H, CH–piperidine, CH_2_–piperidine); ^13^C NMR (101 MHz, chloroform-*d*): δ 173.5, 158.3, 139.1, 134.6, 130.3, 130.0, 129.9, 124.2, 119.9, 117.7, 114.2, 72.9, 62.5, 52.8, 46.1, 44.5, 36.3, 30.0, 28.9; HRMS (ESI^+^) for C_25_H_33_ClN_3_O_2_ ([M + H]^+^): calcd, 442.22558; found, 442.22533; HPLC: *t*_R_ = 3.05 min (97.9% at 254 nm).

##### Synthesis of *N*-(3,4-Dichlorophenyl)-1-(4-(piperidin-4-ylmethoxy)benzyl)piperidine-4-carboxamide (**89**)

It was synthesized according to general procedure C using compound **81** (0.149 mmol, 86 mg) as reagent. Yield: 70.4%; white solid; ^1^H NMR (400 MHz, chloroform-*d*): δ 7.78 (d, *J* = 2.4 Hz, 1H, CONH), 7.39–7.28 (m, 2H, 2× Ar–H), 7.25–7.17 (m, 3H, × Ar–H), 6.88–6.80 (m, 2H, 2 × Ar–H), 3.78 (d, *J* = 6.3 Hz, 2H, CH_2_), 3.45 (s, 2H, CH_2_), 3.12 (dt, *J*_1_ = 12.2 Hz, *J*_2_ = 3.3 Hz, 2H, CH_2_–piperidine), 2.96 (d, *J* = 11.0 Hz, 2H, CH_2_–piperidine), 2.65 (td, *J*_1_ = 12.2 Hz, *J*_2_ = 2.6 Hz, 2H, CH_2_–piperidine), 2.22 (tt, *J*_1_ = 10.2 Hz, *J*_2_ = 4.5 Hz, 1H, CH–piperidine), 2.05–1.97 (m, 2H, CH_2_–piperidine), 1.95–1.78 (m, 7H, CH–piperidine, 3 × CH_2_–piperidine), 1.34–1.19 (m, 2H, CH_2_–piperidine); ^13^C NMR (101 MHz, chloroform-*d*): δ 173.4, 158.3, 137.4, 132.7, 130.5, 130.3, 129.9, 121.5, 119.0, 114.2, 76.7, 72.8, 62.5, 52.7, 46.0, 44.5, 36.2, 29.9, 28.8; HRMS (ESI^+^) for C_25_H_32_Cl_2_N_3_O_2_ ([M + H]^+^): calcd, 476.18661; found, 476.18632; HPLC: *t*_R_ = 4.753 min (97.2% at 254 nm).

##### Synthesis of *N*-(4-Chlorophenyl)-1-(4-(piperidin-4-ylmethoxy)benzyl)piperidine-4-carboxamide (**90**)

It was synthesized according to general procedure C using compound **82** (0.170 mmol, 90 mg) as reagent. Yield: 65.8%; white solid; ^1^H NMR (400 MHz, chloroform-*d*): δ 7.46 (d, *J* = 8.8 Hz, 2H, 2 × Ar–H), 7.28 (d, *J* = 2.1 Hz, 2H, 2 × Ar–H), 7.21 (d, *J* = 8.6 Hz, 2H, 2 × Ar–H), 7.12 (s, 1H, NHCO), 6.84 (d, *J* = 8.6 Hz, 2H, 2 × Ar–H), 3.78 (d, *J* = 6.3 Hz, 2H, CH_2_), 3.45 (s, 2H, CH_2_), 3.12 (d, *J* = 11.9 Hz, 2H, CH_2_–piperidine), 2.96 (d, *J* = 11.5 Hz, 2H, CH_2_–piperidine), 2.65 (td, *J*_1_ = 12.1 Hz, *J*_2_ = 2.4 Hz, 2H, CH_2_–piperidine), 2.27–2.18 (m, 1H, CH–piperidine), 2.04–1.96 (m, 2H, CH_2_–piperidine), 1.93–1.79 (m, 6H, 3 × CH_2_–piperidine), 1.27 (m, 3H, CH–piperidine, CH_2_–piperidine), signal for NH not seen in the spectrum; ^13^C NMR (101 MHz, chloroform-*d*): δ 173.5, 158.2, 136.5, 130.3, 130.2, 129.1, 129.0, 121.1, 114.1, 72.7, 62.5, 52.8, 45.9, 44.5, 36.1, 29.5, 28.9; HRMS (ESI^+^) for C_25_H_33_ClN_3_O_2_ ([M + H]^+^): calcd, 442.22558; found, 442.22540, HPLC: *t*_R_ = 4.03 min, (98.1% at 254 nm).

##### Synthesis of *N*-(4-Fluorophenyl)-1-(4-(piperidin-4-ylmethoxy)benzyl)piperidine-4-carboxamide (**91**)

It was synthesized according to general procedure C using compound **83** (0.097 mmol, 51 mg) as reagent. Yield: 100%; light brown solid; ^1^H NMR (400 MHz, chloroform-*d*): δ 7.46 (dd, *J*_1_ = 9.0 Hz, *J*_2_ = 4.8 Hz, 2H, 2 × Ar–H), 7.21 (d, *J* = 8.6 Hz, 2H, 2 × Ar–H), 7.12 (s, 1H, NHCO), 7.00 (t, *J* = 8.7 Hz, 2H, 2 × Ar–H), 6.84 (d, *J* = 8.6 Hz, 2H, 2 × Ar–H), 3.78 (d, *J* = 6.4 Hz, 2H, CH_2_), 3.45 (s, 2H, CH_2_), 3.13 (d, *J* = 12.0 Hz, 2H, CH_2_–piperidine), 2.96 (d, *J* = 11.5 Hz, 2H, CH_2_–piperidine), 2.65 (td, *J*_1_ = 12.2 Hz, *J*_2_ = 2.5 Hz, 2H, CH_2_–piperidine), 2.27–2.18 (m, 1H, CH–piperidine), 2.04–1.96 (m, 2H, CH_2_–piperidine), 1.94–1.79 (m, 6H, 3 × CH_2_–piperidine), 1.28 (ddd, *J*_1_ = 24.7 Hz, *J*_2_ = 12.1 Hz, *J*_3_ = 4.0 Hz, 3H, CH–piperidine, CH_2_–piperidine); ^13^C NMR (101 MHz, chloroform-*d*): δ 173.3, 158.3, 130.3, 130.0, 121.7, 121.6, 115.7, 115.5, 114.1, 72.9, 62.6, 52.9, 46.3, 44.4, 36.4, 30.2, 28.9; HRMS (ESI^+^) for C_25_H_33_FN_3_O_2_ (M + H^+^): calcd, 426.25513; found, 426.25473; HPLC: *t*_R_ = 2.69 min (97.7% at 254 nm).

##### Synthesis of *N*-(4-Bromophenyl)-1-(4-(piperidin-4-ylmethoxy)benzyl)piperidine-4-carboxamide (**92**)

It was synthesized according to general procedure C using compound **84** (0.179 mmol, 105 mg) as reagent. Yield: 56.3%; light brown solid; ^1^H NMR (400 MHz, chloroform-*d*): δ 7.41 (s, 4H, 4 × Ar–H), 7.21 (d, *J* = 8.6 Hz, 2H, 2 × Ar–H), 7.13 (s, 1H, NHCO), 6.84 (d, *J* = 8.6 Hz, 2H, 2 Ar–H), 3.78 (d, *J* = 6.3 Hz, 2H, CH_2_), 3.45 (s, 2H, CH_2_), 3.15 (d, *J* = 12.1 Hz, 2H, CH_2_–piperidine), 2.96 (d, *J* = 11.6 Hz, 2H, CH_2_–piperidine), 2.67 (td, *J*_1_ = 12.3 Hz, *J*_2_ = 2.5 Hz, 2H, CH_2_–piperidine), 2.26–2.18 (m, 1H, CH–piperidine), 2.04–1.96 (m, 2H, CH_2_–piperidine), 1.94–1.79 (m, 6H, 3 × CH_2_–piperidine), 1.36–1.24 (m, 3H, CH–piperidine, CH_2_–piperidine); ^13^C NMR (101 MHz, chloroform-*d*): δ 173.4, 158.3, 137.0, 131.1, 130.3, 130.0, 121.4, 116.7, 114.1, 72.9, 62.5, 52.8, 46.1, 44.5, 36.3, 30.0, 28.9; HRMS (ESI^+^) for C_25_H_33_BrN_3_O_2_ ([M + H]^+^): calcd, 486.17507; found, 486.17482; HPLC: *t*_R_ = 4.24 (95.3% at 254 nm).

##### Synthesis of *N*-(3-Chlorophenyl)-1-(4-(piperidin-4-yloxy)benzyl)piperidine-4-carboxamide (**93**)

It was synthesized according to general procedure C using compound **85** (0.117 mmol, 62 mg) as reagent. Yield: 81.6%; white solid; ^1^H NMR (400 MHz, chloroform-*d*): δ 7.68–7.64 (m, 1H, NHCO), 7.35–7.31 (m, 1H, Ar–H), 7.22 (t, *J* = 8.8 Hz, 3H, 3 × Ar–H), 7.13 (s, 1H, Ar–H), 7.09–7.05 (m, 1H, Ar–H), 6.86 (d, *J* = 8.6 Hz, 2H, 2 × Ar–H), 4.39–4.32 (m, 1H, CH–piperidine), 3.45 (s, 2H, CH_2_), 3.19–3.12 (m, 2H, CH_2_–piperidine), 2.97 (d, *J* = 11.8 Hz, 2H, CH_2_–piperidine), 2.79–2.71 (m, 2H, CH_2_–piperidine), 2.27–2.19 (m, 1H, CH–piperidine), 2.06–1.97 (m, 4H, 2 × CH_2_–piperidine), 1.94–1.79 (m, 5H, CH–piperidine, 2 × CH_2_–piperidine), 1.73–1.65 (m, 1H, CH–piperidine), signal for NH not seen in the spectrum; ^13^C NMR (101 MHz, chloroform-*d*): δ 173.5, 156.4, 139.1, 134.6, 130.4, 130.3, 129.9, 124.2, 119.9, 117.7, 115.8, 73.0, 62.5, 52.8, 44.5, 43.7, 32.0, 28.9; HRMS (ESI^+^) for C_24_H_31_ClN_3_O_2_ ([M + H]^+^): calcd, 428.20993; found, 428.20983; HPLC: *t*_R_ = 2.93 min (98.0% at 254 nm).

##### Synthesis of *N*-(3,4-Dichlorophenyl)-1-(4-(piperidin-4-yloxy)benzyl)piperidine-4-carboxamide (**94**)

It was synthesized according to general procedure C, using compound **86** (0.048 mmol, 30 mg) as reagent. Yield: 100%; off-white solid; ^1^H NMR (400 MHz, chloroform-*d*): δ 7.79 (d, *J* = 1.9 Hz, 1H, NHCO), 7.37–7.30 (m, 3H, 3 × Ar–H), 7.21 (d, *J* = 8.5 Hz, 2H, 2 × Ar–H), 6.86 (d, *J* = 8.5 Hz, 2H, 2 × Ar–H), 4.41–4.35 (m, 1H, CH–piperidine), 3.44 (s, 2H, CH_2_), 3.20–3.13 (m, 2H, CH_2_–piperidine), 2.96 (d, *J* = 11.0 Hz, 2H, CH_2_–piperidine), 2.83–2.75 (m, 2H, CH_2_–piperidine), 1.91–1.81 (m, 6H, 3 × CH_2_–piperidine), 1.25 (s, 3H, CH–piperidine, CH_2_–piperidine), 0.91–0.80 (m, 2H, CH_2_–piperidine), signal for NH not seen in the spectrum; ^13^C NMR (101 MHz, chloroform-*d*): δ 173.5, 156.4, 137.4, 132.8, 130.4, 130.4, 130.3, 127.3, 121.5, 119.0, 115.8, 72.7, 62.5, 52.8, 44.5, 43.5, 31.8, 28.8; HRMS (ESI^+^) for C_24_H_30_Cl_2_N_3_O_2_ ([M + H]^+^): calcd, 462.17096; found, 462.17058; HPLC: *t*_R=_ 2.00 min (95.4% at 254 nm).

##### Synthesis of *N*-(4-Chlorophenyl)-1-(4-(piperidin-4-yloxy)benzyl)piperidine-4-carboxamide (**95**)

It was synthesized according to general procedure C using compound **87** (0.170 mmol, 90 mg) as reagent. Yield: 65.8%; white solid; ^1^H NMR (400 MHz, chloroform-*d*): δ 7.46 (d, *J* = 8.7 Hz, 2H, 2 × Ar–H), 7.28 (d, *J* = 2.2 Hz, 2H, 2 × Ar–H), 7.21 (d, *J* = 8.6 Hz, 2H, 2 × Ar–H), 7.11 (s, 1H, NHCO), 6.86 (d, *J* = 8.6 Hz, 2H, 2 × Ar–H), 4.38–4.30 (m, 2H, CH_2_–piperidine), 3.44 (s, 2H, CH_2_), 3.18–3.11 (m, 2H, CH_2_–piperidine), 2.99–2.94 (m, 2H, CH_2_–piperidine), 2.76–2.69 (m, 2H, CH_2_–piperidine), 2.05–1.96 (m, 4H, 2 × CH_2_–piperidine), 1.92–1.82 (m, 4H, 2 × CH_2_–piperidine), 1.70–1.63 (m, 2H, CH_2_–piperidine), signal for NH not seen in the spectrum; ^13^C NMR (101 MHz, chloroform-*d*): δ 173.7, 156.5, 136.6, 130.4, 130.2, 129.1, 128.9, 121.2, 115.8, 73.3, 62.5, 52.8, 44.4, 43.9, 32.3, 28.8; HRMS (ESI^+^) for C_24_H_31_ClN_3_O_2_ ([M + H]^+^): calcd, 428.20993; found, 428.20993; HPLC: *t*_R_ = 2.89 min (98.0% at 254 nm).

##### Synthesis of (4-((3,4-Dichlorophenoxy)methyl)-4-hydroxypiperidin-1-yl)(4-((1-methylpiperidin-4-yl)methoxy)phenyl)methanone (**96**)

It was synthesized according to general procedure B using formaldehyde (0.525 mmol, 19.2 μL) and compound **46** (0.105 mmol, 50 mg) as reagents. The solvent was removed under reduced pressure, and the crude product was dissolved in dichloromethane (30 mL). The organic phase was washed with 2 M NaOH (30 mL). Dichloromethane was removed under reduced pressure. Yield: 97.1%; white solid; ^1^H NMR (400 MHz, chloroform-*d*): δ 7.43–7.30 (m, 3H, 3 × Ar–H), 7.01 (d, *J* = 3.0 Hz, 1H, Ar–H), 6.89 (d, *J* = 8.6 Hz, 2H, 2 × Ar–H), 6.77 (dd, *J*_1_ = 8.9 Hz, *J*_2_ = 2.9 Hz, 1H, Ar–H), 4.49 (s, 1H, CH–piperidine), 3.83 (d, *J* = 6.1 Hz, 2H, CH_2_), 3.80 (s, 2H, CH_2_), 3.39 (s, 2H, CH_2_–piperidine), 2.89 (d, *J* = 11.2 Hz, 2H, CH_2_–piperidine), 2.29 (s, 3H, CH_3_), 2.21 (s, 1H, CH–piperidine), 1.96 (td, *J*_1_ = 11.8 Hz, *J*_2_ = 2.3 Hz, 2H, CH_2_–piperidine), 1.91–1.66 (m, 7H, 3 × CH_2_–piperidine, CH–piperidine), 1.44 (td, *J*_1_ = 12.1 Hz, *J*_2_ = 3.9 Hz, 2H, CH_2_–piperidine), signal for OH not seen in the spectrum; ^13^C NMR (101 MHz, chloroform-*d*): δ 170.4, 160.3, 157.5, 133.0, 130.8, 129.0, 127.9, 124.7, 116.5, 114.6, 114.2, 76.0, 72.7, 69.3, 55.4, 46.5, 35.2, 29.1; HRMS (ESI^+^) for C_26_H_32_Cl_2_N_2_O_4_ ([M + H]^+^): calcd, 507.18119; found, 507.18058; HPLC: *t*_R_ = 4.85 min (97.4% at 254 nm).

##### Synthesis of (4-((1-(3,4-Dichlorobenzyl)piperidin-4-yl)methoxy)phenyl)(4-((3,4-dichlorophenoxy)methyl)-4-hydroxypiperidin-1-yl)methanone (**97**)

It was synthesized according to general procedure B using compound **46** (0.355 mmol, 175 mg) and 3,4-dichlorobenzaldehyde (0.426 mmol, 74.6 mg) as reagents. The reaction mixture was stirred for 18 h. The crude product was purified using flash column chromatography using dichloromethane/methanol (20:1) as eluent. Yield: 45.8%; white solid; ^1^H NMR (400 MHz, chloroform-*d*): δ 7.44 (d, *J* = 2.0 Hz, 1H, Ar–H), 7.39 (s, 2H, 2 × Ar–H), 7.37–7.32 (m, 2H, 2 × Ar–H), 7.16 (d, *J* = 8.8 Hz, 1H, Ar–H), 7.02 (d, *J* = 2.9 Hz, 1H, Ar–H), 6.89 (d, *J* = 8.6 Hz, 2H, 2 × Ar–H), 6.77 (dd, *J*_1_ = 8.9 Hz, *J*_2_ = 2.9 Hz, 1H, Ar–H), 4.15 (s, 1H, CH–piperidine), 3.83 (d, *J* = 5.9 Hz, 2H, CH_2_), 3.80 (s, 2H, CH_2_), 3.45 (s, 2H, CH_2_), 2.89 (d, *J* = 11.2 Hz, 2H, CH_2_–piperidine), 2.12 (s, 1H, CH–piperidine), 2.01 (t, *J* = 11.5 Hz, 2H, CH_2_–piperidine), 1.82 (d, *J* = 11.7 Hz, 4H, 2 × CH_2_–piperidine), 1.77–1.62 (m, 4H, CH_2_–piperidine), 1.41 (m, 3H, CH–piperidine, CH_2_–piperidine), signal for OH not seen in the spectrum; HRMS (ESI^+^) for C_32_H_34_Cl_4_N_2_O_4_ ([M + H]^+^): calcd, 651.13454; found, 651.13339; HPLC: *t*_R_ = 5.83 min (96.8% at 254 nm).

##### Synthesis of (4-((1-(4-Chlorobenzyl)piperidin-4-yl)methoxy)phenyl)(4-((3,4-dichlorophenoxy)methyl)-4-hydroxypiperidin-1-yl)methanone (**98**)

It was synthesized according to general procedure B using compound **46** (0.203 mmol, 100 mg) and 4-chlorobenzaldehyde (0.243 mmol, 34 mg). The crude product was purified using flash column chromatography using dichloromethane/methanol (4:1) as eluent. Yield: 16.8%; colorless oil; ^1^H NMR (300 MHz, chloroform-*d*): δ 7.38 (d, *J* = 8.8 Hz, 2H, 2 × Ar–H), 7.35–7.30 (m, 1H, Ar–H), 7.28 (dd, *J*_1_ = 5.8 Hz, *J*_2_ = 4.7 Hz, 4H, 4 × Ar–H), 7.02 (d, *J* = 2.9 Hz, 1H, Ar–H), 6.90 (d, *J* = 8.7 Hz, 2H, 2 × Ar–H), 6.78 (dd, *J*_1_ = 8.9 Hz, *J*_2_ = 2.9 Hz, 1H, Ar–H), 4.47 (d, *J* = 13.3 Hz, 1H, OH), 3.83 (d, *J* = 6.0 Hz, 2H, CH_2_), 3.80 (d, *J* = 6.2 Hz, 2H, CH_2_), 3.49 (s, 2H, CH_2_), 3.00–2.85 (m, 2H, CH_2_–piperidine), 2.13 (s, 1H, CH–piperidine), 2.02 (t, *J* = 10.8 Hz, 2H, CH_2_–piperidine), 1.95–1.50 (m, 10H, 5 × CH_2_–piperidine), 1.50–1.25 (m, 2H, CH_2_–piperidine); ^13^C NMR (101 MHz, chloroform-*d*): δ 170.4, 168.9, 160.3, 157.4, 133.0, 132.7, 130.8, 130.5, 129.0, 128.3, 127.9, 124.8, 116.5, 116.5, 114.5, 114.2, 72.7, 69.4, 69.2, 62.6, 53.3, 42.0, 37.0, 35.8, 34.3, 33.5, 29.0, 21.5; HRMS (ESI^+^) for C_32_H_35_Cl_3_N_2_O_4_ ([M + H]^+^): calcd 617.1735; found, 617.1718; HPLC: *t*_R_ = 4.79 min (96.2% at 254 nm).

##### Synthesis of (4-((1-Benzylpiperidin-4-yl)methoxy)phenyl)(4-((3,4-dichlorophenoxy)methyl)-4-hydroxypiperidin-1-yl)methanone (**99**)

It was synthesized according to general procedure B using compound **46** (0.203 mmol, 100 mg) and benzaldehyide (0.243 mmol, 25 μL). The crude product was purified using flash column chromatography using dichloromethane/methanol (20:1) as eluent. Yield: 97.2%; colorless oil; ^1^H NMR (400 MHz, chloroform-*d*): δ 7.38 (d, *J* = 2.4 Hz, 1H, Ar–H), 7.35 (d, *J* = 2.4 Hz, 2H, 2 × Ar–H), 7.31 (dd, *J*_1_ = 9.5 Hz, *J*_2_ = 3.8 Hz, 5H, 5 × Ar–H), 7.01 (d, *J* = 2.9 Hz, 1H, Ar–H), 6.89 (t, *J* = 5.7 Hz, 2H, 2 × Ar–H), 6.77 (dd, *J*_1_ = 8.9 Hz, *J*_2_ = 2.9 Hz, 1H, Ar–H), 3.82 (d, *J* = 6.0 Hz, 2H, CH_2_), 3.80 (s, 2H, CH_2_), 3.52 (s, 2H, CH_2_), 2.94 (d, *J* = 11.4 Hz, 2H, CH_2_–piperidine), 2.19 (s, 1H, CH–piperidine), 2.01 (t, *J* = 10.8 Hz, 2H, CH_2_–piperidine), 1.85–1.76 (m, 4H, 2 × CH_2_–piperidine), 1.64 (s, 6H, 3 × CH_2_–piperidine), 1.42 (dd, *J*_1_ = 12.1 Hz, *J*_2_ = 2.7 Hz, 2H, CH_2_–piperidine); ^13^C NMR (101 MHz, chloroform-*d*): δ 170.5, 160.4, 157.5, 138.2, 132.9, 130.8, 129.3, 128.9, 128.2, 127.7, 127.0, 124.5, 116.5, 114.6, 114.2, 76.0, 72.7, 69.2, 63.4, 53.3, 43.6, 35.8, 33.9, 29.0; HRMS (ESI^+^) for C_32_H_36_Cl_2_N_2_O_4_ ([M + H]^+^): calcd, 583.2125; found, 583.2108; HPLC: *t*_R_ = 4.63 min (95.0% at 254 nm).

##### Synthesis of (4-((1-(Cyclohexylmethyl)piperidin-4-yl)methoxy)phenyl)(4-((3,4-dichlorophenoxy)methyl)-4-hydroxypiperidin-1-yl)methanone (**100**)

It was synthesized according to general procedure B using compound **46** (0.203 mmol, 100 mg) and cyclohexanecarbaldehyde (0.243 mmol, 30 μL). The crude product was purified using flash column chromatography using dichloromethane/methanol (20:1) as eluent. Yield: 67.8%; colorless oil; ^1^H NMR (400 MHz, chloroform-*d*): δ 7.37 (d, *J* = 8.4 Hz, 2H, 2 × Ar–H), 7.34 (d, *J* = 8.9 Hz, 1H, Ar–H), 7.03 (d, *J* = 2.9 Hz, 1H, Ar–H), 6.88 (d, *J* = 8.4 Hz, 2H, 2 × Ar–H), 6.78 (dd, *J*_1_ = 8.9 Hz, *J*_2_ = 2.8 Hz, 1H, Ar–H), 3.89 (s, 2H, CH_2_), 3.80 (s, 2H, CH_2_), 3.73 (d, *J* = 12.1 Hz, 2H, CH_2_), 3.47 (d, *J* = 1.6 Hz, 2H, CH_2_), 2.85 (d, *J* = 6.1 Hz, 2H, CH_2_), 2.64 (s, 2H, CH_2_), 2.58 (s, 2H, CH_2_), 2.03 (s, 4H, 2 × CH_2_), 1.87–1.76 (m, 8H, 4 × CH_2_), 1.34–1.13 (m, 5H, CH, 2 × CH_2_), 1.06 (d, *J* = 11.5 Hz, 3H, CH, CH_2_), signal for OH not seen in the spectrum; ^13^C NMR (101 MHz, chloroform-*d*): δ 170.5, 160.3, 157.5, 133.0, 130.8, 128.9, 127.9, 124.7, 116.6, 114.6, 114.3, 76.0, 72.6, 69.3, 65.9, 53.9, 50.7, 35.7, 35.0, 32.0, 28.6, 26.6, 26.1; HRMS (ESI^+^) for C_32_H_42_Cl_2_N_2_O_4_ ([M + H]^+^): calcd, 589.2594; found, 589.2577; HPLC: *t*_R_ = 4.85 min (98.1% at 254 nm).

##### Synthesis of (4-((1-(Cyclopropylmethyl)piperidin-4-yl)methoxy)phenyl)(4-((3,4-dichlorophenoxy)methyl)-4-hydroxypiperidin-1-yl)methanone (**101**)

It was synthesized according to general procedure B using compound **46** (0.203 mmol, 100 mg) and cyclopropylcarbaldehyde (0.243 mmol, 18 μL). The crude product was purified using flash column chromatography using dichloromethane/methanol (4:1) as eluent. Yield: 88.3%; colorless oil; ^1^H NMR (400 MHz, chloroform-*d*): δ 7.38 (d, *J* = 8.7 Hz, 2H, 2 × Ar–H), 7.34 (d, *J* = 8.9 Hz, 1H, Ar–H), 7.02 (d, *J* = 2.9 Hz, 1H, Ar–H), 6.89 (d, *J* = 8.8 Hz, 2H, 2 × Ar–H), 6.77 (dd, *J*_1_ = 8.9 Hz, *J*_2_ = 2.9 Hz, 1H, Ar–H), 3.90 (d, *J* = 5.8 Hz, 2H, CH_2_), 3.81 (s, 2H, CH_2_), 3.53 (d, *J* = 11.8 Hz, 2H, CH_2_), 3.49 (s, 2H, CH_2_), 2.71 (d, *J* = 7.1 Hz, 2H, CH_2_), 2.56 (t, *J* = 12.0 Hz, 2H, CH_2_), 1.91–1.81 (m, 5H, CH, 2 × CH_2_), 1.72–1.53 (m, 4H, 2 × CH_2_), 1.09 (s, 2H, CH_2_), 1.00 (dt, *J*_1_ = 8.0 Hz, *J*_2_ = 3.9 Hz, 1H, CH), 0.86 (dt, *J*_1_ = 7.4 Hz, *J*_2_ = 4.0 Hz, 2H, CH_2_), 0.74 (q, *J* = 5.9 Hz, 2H, CH_2_); ^13^C NMR (101 MHz, chloroform-*d*): δ 170.4, 159.9, 157.5, 133.0, 130.8, 128.9, 128.2, 124.6, 116.5, 114.6, 114.3, 76.0, 71.5, 69.3, 62.1, 52.1, 34.5, 26.6, 13.9, 7.8, 6.2, 4.6; HRMS (ESI^+^) for C_29_H_36_Cl_2_N_2_O_4_ ([M + H]^+^): calcd, 547.2125; found, 547.2109; HPLC: *t*_R_ = 4.41 min (100% at 254 nm).

##### Synthesis of (4-((3,4-Dichlorophenoxy)methyl)-4-hydroxypiperidin-1-yl)(4-((1-(2-hydroxyethyl)piperidin-4-yl)methoxy)phenyl)methanone (**102**)

To solution of compound **46** (0.1 mmol, 50 mg) in dry acetonitrile, DIPEA (0.152 mmol, 26 μL) and 2-chloroethanol (0.122 mmol, 8 μL) were added. The reaction mixture was heated to 120 °C in a microwave synthesizer. After 2 h, the reaction was quenched with water. The crude product was purified using flash column chromatography using dichloromethane/methanol (15:1) as eluent. Yield: 18.4%; ^1^H NMR (400 MHz, chloroform-*d*): δ 7.38 (d, *J* = 8.7 Hz, 2H, 2 × Ar–H), 7.34 (d, *J* = 8.9 Hz, 1H, Ar–H), 7.02 (d, *J* = 2.9 Hz, 1H, Ar–H), 6.89 (d, *J* = 8.8 Hz, 2H, 2 × Ar–H), 6.77 (dd, *J*_1_ = 8.9 Hz, *J*_2_ = 2.9 Hz, 1H, Ar–H), 3.87 (d, *J* = 5.9 Hz, 2H, CH_2_), 3.82 (d, *J* = 8.9 Hz, 4H, 2 × CH_2_), 3.45–3.20 (m, 4H, 2 × CH_2_), 2.84 (s, 2H, CH_2_), 2.46 (t, *J* = 11.7 Hz, 3H, CH, CH_2_), 1.97 (d, *J* = 11.0 Hz, 4H, 2 × CH_2_), 1.76 (d, *J* = 11.9 Hz, 4H, 2 × CH_2_), 1.47 (d, *J* = 6.5 Hz, 2H, CH_2_); ^13^C NMR (101 MHz, chloroform-*d*): δ 170.3, 160.0, 157.4, 133.1, 130.8, 129.0, 128.2, 124.8, 116.5, 114.5, 114.2, 77.2, 76.0, 71.9, 69.4, 60.2, 57.2, 53.5, 35.0, 27.7; HRMS (ESI^+^) for C_27_H_34_Cl_2_N_2_O_5_ ([M + H]^+^): calcd, 537.1917; found, 537.1907; HPLC: *t*_R_ = 4.03 min (100% at 254 nm).

##### Synthesis of 1-(4-(4-(4-((3,4-Dichlorophenoxy)methyl)-4-hydroxypiperidine-1-carbonyl)phenoxy)piperidin-1-yl)ethan-1-one (**103**)

To a solution of compound **51** (0.6 mmol, 285 mg) in ethyl acetate (10 mL), 5 mL aqueous solution of NaHCO_3_ (1.27 mmol, 107 mg) and acetanhydride (0.765 mmol, 72 μL) were added. The reaction mixture was stirred for 1 h at 20 °C. Phases were separated, and organic phase was washed with 10% citric acid aqueous solution (2 × 10 mL). The organic phase was dried over anhydrous Na_2_SO_4_ and filtered, and the solvent was removed in vacuo*.* Yield: 79.0%; colorless oil; ^1^H NMR (400 MHz, chloroform-*d*): δ 7.39 (d, *J* = 1.9 Hz, 1H, Ar–H), 7.38 (d, *J* = 1.9 Hz, 1H, Ar–H), 7.34 (d, *J* = 8.9 Hz, 1H, Ar–H), 7.01 (d, *J* = 2.9 Hz, 1H, Ar–H), 6.92 (d, *J* = 8.7 Hz, 2H, 2 × Ar–H), 6.77 (dd, *J*_1_ = 8.9 Hz, *J*_2_ = 2.9 Hz, 1H, Ar–H), 4.60–4.57 (m, 1H, CH–piperidine), 3.80 (s, 2H, CH_2_), 3.74–3.65 (m, 4H, 2 × CH_2_–piperidine), 3.42 (ddd, *J*_1_ = 13.5 Hz, *J*_2_ = 6.7 Hz, *J*_3_ = 3.9 Hz, 4H, 2 × CH_2_–piperidine), 2.22 (s, 1H, OH), 2.12 (s, 3H, CH_3_), 2.00–1.86 (m, 4H, 2 × CH_2_–piperidine), 1.86–1.77 (m, 4H, 2 × CH_2_–piperidine); ^13^C NMR (101 MHz, DMSO-*d*_6_): δ 169.2, 168.6, 158.8, 158.3, 132.0, 131.4, 129.3, 128.9, 122.8, 117.0, 116.2, 115.7, 76.4, 72.4, 68.7, 43.4, 38.5, 31.4, 30.7, 21.8; HRMS (ESI^+^) for C_26_H_31_Cl_2_N_2_O_5_ ([M + H]^+^): calcd, 521.16155; found, 521.15969; HPLC: *t*_R_ = 5.08 min (99.4% at 254 nm).

##### Synthesis of 4-((3,4-Dichlorophenoxy)methyl)-1-(4-((1-methylpiperidin-4-yl)methoxy)benzyl)piperidin-4-ol (**104**)

It was synthesized according to general procedure B using compound **57** (0.044 mmol, 20 mg) and formaldehyde (0.22 mmol, 6 μL) as reagents. The product was purified using reverse phase flash chromatography. Yield: 89.1%; white solid; ^1^H NMR (400 MHz, methanol-*d*_4_): δ 7.40 (d, *J* = 8.9 Hz, 1H, Ar–H), 7.30–7.22 (m, 2H, 2 × Ar–H), 7.15 (d, *J* = 2.9 Hz, 1H, Ar–H), 6.96–6.85 (m, 3H, 2 × Ar–H), 3.85 (d, *J* = 6.1 Hz, 2H, CH_2_), 3.81 (s, 2H, CH_2_), 3.52 (s, 2H, CH_2_), 2.94 (d, *J* = 11.8 Hz, 2H, CH_2_–piperidine), 2.75–2.66 (m, 2H, CH_2_–piperidine), 2.47 (td, *J*_1_ = 11.7 Hz, *J*_2_ = 3.0 Hz, 2H, CH_2_–piperidine), 2.31 (s, 3H, CH_3_), 2.09 (td, *J*_1_ = 12.0 Hz, *J*_2_ = 2.5 Hz, 2H, CH_2_–piperidine), 1.96–1.76 (m, 5H, 2 × CH_2_–piperidine, CH–piperidine), 1.71 (d, *J* = 13.6 Hz, 2H, CH_2_–piperidine), 1.53–1.38 (m, 2H, CH_2_–piperidine); ^13^C NMR (101 MHz, chloroform-*d*): δ 158.3, 157.7, 132.9, 130.7, 130.4, 124.4, 116.5, 114.6, 114.1, 77.2, 76.1, 72.6, 69.0, 62.5, 55.5, 48.7, 46.4, 35.3, 33.9, 29.1; HRMS (ESI^+^) for C_26_H_34_Cl_2_N_2_O_3_ ([M + H]^+^): calcd, 492.19465; found, 492.19672; HPLC: *t*_R_ = 3.46 min (99.1% at 254 nm).

##### Synthesis of 3,4-Dichloro-*N*-(1-(4-((1-methylpiperidin-4-yl)methoxy)benzyl)piperidin-4-yl)benzamide (**105**)

It was synthesized according to general procedure B using compound **67** (0.252 mmol, 120 mg) and formaldehyde (1.26 mmol, 35 μL) as reagents. The solvent was removed under reduced pressure, and the crude product dissolved in dichloromethane (20 mL). The organic phase was washed with 2 M NaOH (2 × 20 mL). Dichloromethane was removed under reduced pressure. Yield: 98.0%; white solid; ^1^H NMR (400 MHz, chloroform-*d*): δ 7.83 (d, *J* = 2.0 Hz, 1H, Ar–H), 7.56 (dd, *J*_1_ = 8.3 Hz, *J*_2_ = 2.0 Hz, 1H, Ar–H), 7.50 (d, *J* = 8.3 Hz, 1H, Ar–H), 7.24–7.17 (m, 2H, 2 × Ar–H), 6.87–6.81 (m, 2H, 2 × Ar–H), 5.86 (d, *J* = 7.9 Hz, 1H, CONH), 3.97 (d, *J* = 7.7 Hz, 1H, CH–piperidine), 3.79 (d, *J* = 6.3 Hz, 2H, CH_2_), 3.45 (s, 2H, CH_2_), 2.86 (dd, *J*_1_ = 20.3 Hz, *J*_2_ = 11.6 Hz, 4H, 2 × CH_2_–piperidine), 2.29 (s, 3H, CH_3_), 2.14 (t, *J* = 11.2 Hz, 2H, CH_2_–piperidine), 2.04–1.92 (m, 4H, 2 × CH_2_–piperidine), 1.84 (d, *J* = 13.8 Hz, 2H, CH_2_–piperidine), 1.55 (d, *J* = 14.6 Hz, 2H, CH_2_–piperidine), 1.49–1.35 (m, 3H, CH_2_–piperidine, CH–piperidine); ^13^C NMR (101 MHz, chloroform-*d*): δ 173.4, 158.3, 137.3, 132.8, 130.5, 130.2, 130.1, 1215, 118.9, 114.1, 100.0, 77.3, 72.7, 62.5, 55.5, 52.8, 46.5, 44.5, 35.4, 29.2, 28.9; HRMS (ESI^+^) for C_26_H_33_Cl_2_N_3_O_2_ ([M + H]^+^): calcd, 489.19498; found, 489.19399; HPLC: *t*_R_ = 3.23 min (95.0% at 254 nm).

##### Synthesis of 3,4-Dichloro-*N*-(1-(4-((1-(3,4-dichlorobenzyl)piperidin-4-yl)methoxy)benzyl)piperidin-4-yl)benzamide (**106**)

It was synthesized according to general procedure B using compound **67** (0.063 mmol, 30 mg) and 3,4-dichlorobenzaldehyde (0.076 mmol, 13.2 mg) as reagents. The reaction mixture was stirred for 2 days. The crude product was purified using flash column chromatography, using dichloromethane/methanol (9:1) as eluent. Yield: 60.0%; white solid; ^1^H NMR (400 MHz, chloroform-*d*): δ 7.93 (d, *J* = 2.0 Hz, 1H, Ar–H), 7.63 (dd, *J*_1_ = 8.4 Hz, *J*_2_ = 2.1 Hz, 1H, Ar–H), 7.50 (d, *J* = 8.4 Hz, 1H, Ar–H), 7.45 (d, *J* = 1.9 Hz, 1H, Ar–H), 7.40 (d, *J* = 8.2 Hz, 1H, Ar–H), 7.30 (d, *J* = 8.7 Hz, 2H, 2 × Ar–H), 7.19 (dd, *J*_1_ = 8.2 Hz, *J*_2_ = 1.9 Hz, 1H, Ar–H), 6.91 (d, *J* = 8.7 Hz, 2H, 2 × Ar–H), 6.66 (d, *J* = 8.4 Hz, 1H, CONH), 4.23–4.10 (m, 2H, CH_2_–piperidine), 3.88 (s, 2H, CH_2_), 3.82 (d, *J* = 5.9 Hz, 2H, CH_2_), 3.52 (s, 2H, CH_2_), 3.31 (d, *J* = 10.5 Hz, 3H, CH–piperidine, CH_2_–piperidine), 2.97 (d, *J* = 11.3 Hz, 3H, CH–piperidine, CH_2_–piperidine), 2.64–2.54 (m, 2H, CH_2_–piperidine), 2.15–2.10 (m, 3H, CH–piperidine, CH_2_–piperidine), 1.89–1.79 (m, 3H, CH–piperidine, CH_2_–piperidine), 1.53–1.41 (m, 2H, CH_2_–piperidine); ^13^C NMR (101 MHz, chloroform-*d*): δ 176.2, 159.7, 137.9, 135.9, 133.9, 132.9, 132.3, 132.0, 131.1, 130.5, 130.2, 129.4, 128.6, 126.5, 114.9, 72.5, 61.8, 60.7, 53.0, 51.4, 45.4, 35.6, 29.3, 28.6, 21.9; HRMS (ESI^+^) for C_32_H_36_Cl_4_N_3_O_2_ ([M + H]^+^): calcd, 634.15561; found, 634.15533; HPLC: *t*_R_ = 4.17 min (99.3% at 254 nm).

##### Synthesis of Methyl 3′,6-Dimethoxy-[1,1′-biphenyl]-3-carboxylate (**107**)

To a solution of methyl 3-iodo-4-methoxybenzoate (0.684 mmol, 200 mg) in THF (10 mL), 2 M aqueous K_2_CO_3_ solution (1 mL) and PdCl_2_(dppf) (3 mol %) were added. The reaction mixture was stirred at 20 °C for 30 min. Then, (3-methoxyphenyl)boronic acid (1.368 mmol, 206 mg) was added. The reaction mixture was refluxed for 18 h. The crude product was purified using column chromatography using ethyl acetate/hexane (1:9) as eluent. Yield: 83.0%; colorless oil; ^1^H NMR (400 MHz, chloroform-*d*): δ 8.05–7.99 (m, 2H, 2 × Ar–H), 7.33 (d, *J* = 8.0 Hz, 1H, Ar–H), 7.13–7.06 (m, 2H, 2 × Ar–H), 7.03–6.97 (m, 1H, Ar–H), 6.92 (s, 1H, Ar–H), 3.90 (s, 3H, CH_3_), 3.88 (s, 3H, CH_3_), 3.85 (s, 3H, CH_3_); MS (ESI^+^) *m*/*z*: 273.0 ([M + H]^+^).

##### Synthesis of 3′,6-Dimethoxy-[1,1′-biphenyl]-3-carboxylic Acid (**108**)

It was synthesized according to general procedure D using compound **107** (0.57 mmol 156 mg) as reagent. Yield: 68.0%; white solid; ^1^H NMR (400 MHz, chloroform-*d*): δ 8.14–8.07 (m, 2H, 2 × Ar–H), 7.35 (t, *J* = 7.9 Hz, 1H, Ar–H), 7.14–7.10 (m, 1H, Ar–H), 7.08 (dd, *J* = 2.6, 1.5 Hz, 1H, Ar–H), 7.05–7.00 (m, 1H, Ar–H), 6.94–6.89 (m, 1H, Ar–H), 3.90 (s, 3H, CH_3_), 3.85 (s, 3H, CH_3_), signal for COOH not seen in the spectrum; MS (ESI^+^) *m*/*z*: 258.9 ([M + H]^+^).

##### Synthesis of *tert*-Butyl 4-(3′,6-Dimethoxy-[1,1′-biphenyl]-3-carboxamido)piperidine-1-carboxylate (**109**)

It was synthesized according to general procedure A using *tert*-butyl 4-aminopiperidine-1-carboxylate (0.387 mmol, 77.5 mg) and compound **108** (0.387 mmol, 100 mg) as reagents. Yield: 77.2%, white solid; ^1^H NMR (400 MHz, chloroform-*d*): δ 7.79 (dd, *J*_1_ = 8.6 Hz, *J*_2_ = 2.4 Hz, 1H, Ar–H), 7.67 (d, *J* = 2.4 Hz, 1H, Ar–H), 7.34 (t, *J* = 7.9 Hz, 1H, Ar–H), 7.09 (dt, *J*_1_ = 7.6 Hz, *J*_2_ = 1.3 Hz, 1H, Ar–H), 7.06 (dd, *J*_1_ = 2.6 Hz, *J*_2_ = 1.6 Hz, 1H, Ar–H), 7.00 (d, *J* = 8.6 Hz, 1H, Ar–H), 6.91 (ddd, *J*_1_ = 8.3 Hz, *J*_2_ = 2.6 Hz, *J*_3_ = 1.0 Hz, 1H, Ar–H), 5.95 (d, *J* = 7.9 Hz, 1H, CONH), 4.12 (q, *J* = 7.1 Hz, 3H, CH_2_–piperidine, CH–piperidine), 3.86 (s, 3H, CH_3_), 3.85 (s, 3H, CH_3_), 2.92 (d, *J* = 13.5 Hz, 2H, CH_2_–piperidine), 2.04–1.99 (m, 2H, CH_2_–piperidine), 1.46 (s, 9H, 3 × CH_3_), 1.40 (dd, *J*_1_ = 12.0 Hz, *J*_2_ = 4.0 Hz, 2H, CH_2_–piperidine); MS (ESI^+^) *m*/*z*: 441.1 ([M + H]^+^).

##### Synthesis of 3′,6-Dimethoxy-*N*-(piperidin-4-yl)-[1,1′-biphenyl]-3-carboxamide (**110**)

It was synthesized according to general procedure C using compound **109** (0.29 mmol, 131 mg) as reagent. Yield: 100%; white solid; ^1^H NMR (400 MHz, chloroform-*d*): δ 7.80 (dd, *J*_1_ = 8.6 Hz, *J*_2_ = 2.4 Hz, 1H, Ar–H), 7.68 (d, *J* = 2.4 Hz, 1H, Ar–H), 7.34 (t, *J* = 7.9 Hz, 1H, Ar–H), 7.10 (ddd, *J*_1_ = 7.6 Hz, *J*_2_ = 1.6 Hz, *J*_3_ = 1.0 Hz, 1H, Ar–H), 7.06 (dd, *J*_1_ = 2.6 Hz, *J*_2_ = 1.5 Hz, 1H, Ar–H), 7.00 (d, *J* = 8.6 Hz, 1H, Ar–H), 6.91 (ddd, *J*_1_ = 8.2 Hz, *J*_2_ = 2.6 Hz, *J*_3_ = 1.0 Hz, 1H, Ar–H), 6.00 (d, *J* = 8.0 Hz, 1H, CONH), 4.15–4.02 (m, 1H, CH–piperidine), 3.86 (s, 3H, CH_3_), 3.85 (s, 3H, CH_3_), 3.12 (dt, *J*_1_ = 12.2 Hz, *J*_2_ = 3.1 Hz, 2H, CH_2_–piperidine), 2.76 (ddd, *J*_1_ = 12.4 Hz, *J*_2_ = 11.4 Hz, *J*_3_ = 2.6 Hz, 2H, CH_2_–piperidine), 2.08–2.02 (m, 2H, CH_2_–piperidine), 1.49–1.38 (m, 2H, CH_2_–piperidine), signal for NH not seen in the spectrum; MS (ESI^+^) *m*/*z*: 341.1 ([M + H]^+^).

##### Synthesis of *tert*-Butyl 4-((4-(4-(3′,6-Dimethoxy-[1,1′-biphenyl]-3-carboxamido)piperidine-1-carbonyl)phenoxy)methyl)piperidine-1-carboxylate (**111**)

It was synthesized according to general procedure A using compound **26** (0.293 mmol, 98 mg) and compound **110** (0.293 mmol, 100 mg) as reagents. Yield: 88.3%; colorless oil; ^1^H NMR (400 MHz, chloroform-*d*): δ 7.80 (dd, *J*_1_ = 8.6 Hz, *J*_2_ = 2.4 Hz, 1H, Ar–H), 7.68 (d, *J* = 2.4 Hz, 1H, Ar–H), 7.41–7.32 (m, 3H, 3 × Ar–H), 7.09 (ddd, *J*_1_ = 7.6 Hz, *J*_2_ = 1.6 Hz, *J*_3_ = 1.0 Hz, 1H, Ar–H), 7.06 (dd, *J*_1_ = 2.7 Hz, *J*_2_ = 1.6 Hz, 1H, Ar–H), 7.01 (d, *J* = 8.7 Hz, 1H, Ar–H), 6.93–6.86 (m, 3H, 3 × Ar–H), 6.05 (d, *J* = 7.8 Hz, 1H, CONH), 4.30–4.09 (m, 4H, 2 × CH_2_–piperidine), 3.85 (d, *J* = 5.9 Hz, 6H, 2 × CH_3_), 3.82 (d, *J* = 6.4 Hz, 2H, CH_2_), 3.14–3.00 (m, 3H, CH_2_–piperidine, CH–piperidine), 2.81–2.69 (m, 3H, CH_2_–piperidine, CH–piperidine), 2.09 (d, *J* = 10.5 Hz, 2H, CH_2_–piperidine), 2.02–1.91 (m, 1H, CH–piperidine), 1.82 (d, *J* = 13.1 Hz, 2H, CH_2_–piperidine), 1.47 (s, 9H, 3 × CH_3_), 1.33–1.22 (m, 3H, CH_2_–piperidine, CH–piperidine); MS (ESI^+^) *m*/*z*: 658.2 ([M + H]^+^).

##### Synthesis of 3′,6-Dimethoxy-*N*-(1-(4-(piperidin-4-ylmethoxy)benzoyl)piperidin-4-yl)-[1,1′-Biphenyl]-3-carboxamide (**112**)

It was synthesized according to general procedure C using compound **111** (0.26 mmol, 171 mg) as reagent. Yield: 86.3%; colorless oil; ^1^H NMR (400 MHz, chloroform-*d*): δ 7.79 (dd, *J*_1_ = 8.6 Hz, *J*_2_ = 2.4 Hz, 1H, Ar–H), 7.67 (d, *J* = 2.3 Hz, 1H, Ar–H), 7.39–7.35 (m, 2H, 2 × Ar–H), 7.33 (d, *J* = 8.0 Hz, 1H, Ar–H), 7.09 (ddd, *J*_1_ = 7.6 Hz, *J*_2_ = 1.6 Hz, *J*_3_ = 1.0 Hz, 1H, Ar–H), 7.06 (dd, *J*_1_ = 2.6 Hz, *J*_2_ = 1.5 Hz, 1H, Ar–H), 7.01 (d, *J* = 8.7 Hz, 1H, Ar–H), 6.90 (ddt, *J*_1_ = 10.7 Hz, *J*_2_ = 8.7 Hz, *J*_3_ = 2.3 Hz, 3H, 3 × Ar–H), 5.99 (d, *J* = 7.8 Hz, 1H, CONH), 4.29–4.20 (m, 1H, CH–piperidine), 3.86 (s, 3H, CH_3_), 3.85 (s, 3H, CH_3_), 3.81 (d, *J* = 6.4 Hz, 2H, CH_2_), 3.13 (d, *J* = 12.2 Hz, 2H, CH_2_–piperidine), 3.05 (s, 2H, CH_2_–piperidine), 2.66 (td, *J*_1_ = 12.2 Hz, *J*_2_ = 2.6 Hz, 2H, CH_2_–piperidine), 2.09 (d, *J* = 12.6 Hz, 2H, CH_2_–piperidine), 1.98–1.89 (m, 1H, CH–piperidine), 1.83 (d, *J* = 13.0 Hz, 2H, CH_2_–piperidine), 1.48 (s, 2H, CH_2_–piperidine), 1.29 (qd, *J*_1_ = 12.5 Hz, *J*_1_ = 4.5 Hz, 4H, 2 × CH_2_–piperidine), signal for NH not seen in the spectrum; ^13^C NMR (101 MHz, chloroform-*d*): δ 158.3, 157.7, 132.9, 130.7, 130.4, 124.4, 116.5, 114.6, 114.1, 77.3, 76.1, 72.6, 69.0, 62.5, 55.5, 48.7, 46.4, 35.3, 33.9, 29.1; HRMS (ESI^+^) for C_33_H_39_N_3_O_5_: calcd 558.29625, found: 558.29526; HPLC: *t*_R_ = 3.98 min (93.5% at 254 nm).

##### Synthesis of *tert*-Butyl 4-(((Benzyloxy)carbonyl)amino)piperidine-1-carboxylate (**113**)

To a solution of *tert*-butyl 4-aminopiperidine-1-carboxylate (1.25 mmol, 250 mg) in dichloromethane (20 mL), DIPEA (1.25 mmol, 213 μL) was added. The reaction mixture was cooled to 0 °C, and then benzyl chloroformate (1.375 mmol, 192.5 μL) was added dropwise. The reaction mixture was stirred for 18 h and then washed with 1% aqueous solution of citric acid (20 mL). The solvent was removed in vacuo. Yield: 97.6%; yellow oil; ^1^H NMR (400 MHz, chloroform-*d*): δ 7.41–7.30 (m, 5H, 5 × Ar–H), 5.09 (s, 2H, CH_2_), 4.70 (s, 1H, NH), 4.01 (s, 2H CH_2_–piperidine), 3.66 (s, 1H, CH–piperidine), 2.85 (t, *J* = 12.5 Hz, 2H, CH_2_–piperidine), 1.97–1.87 (m, 2H, CH_2_–piperidine), 1.45 (s, 9H, 3 × CH_3_), 1.37–1.21 (m, 2H, CH_2_–piperidine); MS (ESI^+^) *m*/*z*: 335.1 ([M + H]^+^).

##### Synthesis of Benzyl Piperidin-4-ylcarbamate (**114**)

It was synthesized according to general procedure C using compound **113** (1.22 mmol, 407 mg) as reagent. Yield: 85.2%; yellow oil; ^1^H NMR (400 MHz, chloroform-*d*): δ 7.42–7.29 (m, 5H, 5 × Ar–H), 5.09 (s, 2H, CH_2_), 3.63 (s, 1H, NH), 3.11 (dt, *J*_1_ = 12.9 Hz, *J*_2_ = 3.8 Hz, 2H, CH_2_–piperidine), 2.71 (t, *J* = 11.9 Hz, 2H, CH_2_–piperidine), 2.48 (s, 1H, CH–piperidine), 1.98 (d, *J* = 12.9 Hz, 2H, CH_2_–piperidine), 1.45–1.32 (m, 2H, CH_2_–piperidine), signal for NH not seen in the spectrum; MS (ESI^+^) *m*/*z*: 235.1 ([M + H]^+^).

##### Synthesis of *tert*-Butyl 4-((4-(4-(((Benzyloxy)carbonyl)amino)piperidine-1-carbonyl)phenoxy)methyl)piperidine-1-carboxylate (**115**)

It was synthesized according to general procedure A using compound **114** (1.04 mmol, 244 mg) and compound **26** (1.04 mmol, 350 mg) as reagents. The product was crystallized from ethyl acetate/hexane. Yield: 51.0%; white solid; ^1^H NMR (400 MHz, chloroform-*d*): δ 7.44–7.30 (m, 7H, 7 × Ar–H), 6.91–6.85 (m, 2H, 2 × Ar–H), 5.10 (s, 2H, CH_2_), 4.69 (s, 1H, NH), 4.16 (s, 2H, CH_2_), 3.82 (d, *J* = 6.3 Hz, 2H, CH_2_–piperidine), 3.49 (s, 2H, CH_2_–piperidine), 3.04 (s, 2H, CH_2_–piperidine), 2.75 (t, *J* = 12.7 Hz, 2H, CH_2_–piperidine), 1.99 (s, 3H, CH_2_–piperidine, CH–piperidine), 1.82 (d, *J* = 13.1 Hz, 2H, CH_2_–piperidine), 1.47 (s, 9H, 3 × CH_3_), 1.41 (s, 2H, CH_2_–piperidine), 1.28 (dd, *J*_1_ = 12.6 Hz, *J*_2_ = 8.2 Hz, 3H, CH_2_–piperidine, CH–piperidine); MS (ESI^+^) *m*/*z*: 552.1 ([M + H]^+^).

##### Synthesis of Benzyl (1-(4-(Piperidin-4-ylmethoxy)benzoyl)piperidin-4-yl)carbamate (**116**)

It was synthesized according to general procedure C using compound **115** (0.5 mmol, 285 mg) as reagent. Yield: 100%; white solid; ^1^H NMR (400 MHz, chloroform-*d*): δ 7.39–7.31 (m, 7H, 7 × Ar–H), 6.88 (d, *J* = 8.8 Hz, 2H, 2 × Ar–H), 5.10 (s, 2H, CH_2_), 4.70 (s, 1H, CH–piperidine), 3.81 (d, *J* = 6.4 Hz, 2H, CH_2_), 3.74 (s, 1H, CH–piperidine), 3.12 (dd, *J*_1_ = 12.2 Hz, *J*_2_ = 3.4 Hz, 2H, CH_2_–piperidine), 3.04 (s, 2H, CH_2_–piperidine), 2.66 (td, *J*_1_ = 12.2 Hz, *J*_2_ = 2.6 Hz, 2H, CH_2_–piperidine), 2.04–1.88 (m, 3H, CH_2_–piperidine, CH–piperidine), 1.82 (d, *J* = 13.0 Hz, 2H, CH_2_–piperidine), 1.38 (s, 2H, CH_2_–piperidine), 1.34–1.22 (m, 3H, CH_2_–piperidine, CH–piperidine), signal for NH not seen in the spectrum; MS (ESI^+^) *m*/*z*: 452.1 ([M + H]^+^).

##### Synthesis of Benzyl (1-(4-((1-Methylpiperidin-4-yl)methoxy)benzoyl)piperidin-4-yl)carbamate (**117**)

It was synthesized according to general procedure B using compound **116** (0.5 mmol, 226 mg) and formaldehyde (2.5 mmol, 68.93 μL) as reagents. The solvent was removed under reduced pressure, and the crude product was dissolved in dichloromethane (20 mL). The organic phase was washed with 2 M NaOH (2 × 20 mL). Dichloromethane was removed under reduced pressure. Yield: 70.3%; colorless oil; ^1^H NMR (400 MHz, chloroform-*d*): δ 7.34 (d, *J* = 7.4 Hz, 7H, 7 × Ar–H), 6.88 (d, *J* = 8.7 Hz, 2H, 2 × Ar–H), 5.10 (s, 2H, CH_2_), 4.69 (s, 1H, NH), 3.82 (d, *J* = 6.1 Hz, 2H, CH_2_), 3.76 (s, 1H, CH–piperidine), 3.04 (s, 2H, CH_2_–piperidine), 2.93–2.86 (m, 2H, CH_2_–piperidine), 2.29 (s, 3H, CH_3_), 2.05–1.92 (m, 4H, 2 × CH_2_–piperidine), 1.87–1.74 (m, 4H, 2 × CH_2_–piperidine), 1.48–1.32 (m, 5H, 2 × CH_2_–piperidine, CH–piperidine); MS (ESI^+^) *m*/*z*: 466.2 ([M + H]^+^).

##### Synthesis of (4-Aminopiperidin-1-yl)(4-((1-methylpiperidin-4-yl)methoxy)phenyl)methanone (**118**)

Compound **117** (0.365 mmol, 163 mg) was dissolved in methanol (20 mL) under an argon atmosphere, then Pd/C (20 mg) was added, and the reaction mixture was stirred under a hydrogen atmosphere at 20 °C for 18 h. Pd/C was filtered off, and methanol was removed in vacuo. Yield: 97.5%; colorless oil; ^1^H NMR (400 MHz, chloroform-*d*): δ 7.35 (dd, *J*_1_ = 8.8 Hz, *J*_2_ = 1.2 Hz, 2H, 2 × Ar–H), 6.90–6.85 (m, 2H, 2 × Ar–H), 3.82 (d, *J* = 6.2 Hz, 2H, CH_2_), 3.48 (s, 2H, NH_2_), 3.04–2.93 (m, 2H, CH_2_–piperidine), 2.93–2.88 (m, 2H, CH_2_–piperidine), 2.29 (s, 3H, CH_3_), 1.96 (td, *J*_1_ = 11.8 Hz, *J*_2_ = 2.4 Hz, 3H, CH_2_–piperidine, CH–piperidine), 1.84 (d, *J* = 13.3 Hz, 4H, 2 × CH_2_–piperidine), 1.80–1.72 (m, 2H, CH_2_–piperidine), 1.42 (qd, *J*_1_ = 12.0 Hz, *J*_2_ = 11.5 Hz, *J*_3_ = 3.9 Hz, 3H, CH_2_–piperidine, CH–piperidine), 1.32 (s, 2H, CH_2_–piperidine); MS (ESI^+^) *m*/*z*: 332.2 ([M + H]^+^).

##### Synthesis of (4-((3,4-Dichlorobenzyl)amino)piperidin-1-yl)(4-((1-methylpiperidin-4-yl)methoxy)phenyl)methanone (**119**)

It was synthesized according to general procedure A using compound **118** (0.356 mmol, 118 mg) and 3,4-dichlorobenzaldehyde (0.356 mmol, 62.31 mg) as reagents. The mixture was stirred at 20 °C for 2 days. The crude product was purified using flash column chromatography using dichloromethane/methanol (4:1) as eluent. Yield: 31.3%; colorless oil; ^1^H NMR (400 MHz, chloroform-*d*): δ 7.44 (d, *J* = 2.0 Hz, 1H, Ar–H), 7.39 (d, *J* = 8.2 Hz, 1H, Ar–H), 7.35 (d, *J* = 8.7 Hz, 2H, 2 × Ar–H), 7.16 (dd, *J*_1_ = 8.2 Hz, *J*_2_ = 2.0 Hz, 1H, Ar–H), 6.88 (d, *J* = 8.7 Hz, 2H, 2 × Ar–H), 3.83 (d, *J* = 6.0 Hz, 2H, CH_2_), 3.79 (s, 2H, CH_2_), 2.99 (d, *J* = 11.8 Hz, 4H, 2 × CH_2_–piperidine), 2.80–2.73 (m, 1H, CH–piperidine), 2.33 (s, 3H, CH_3_), 2.10–2.05 (m, 2H, CH_2_–piperidine), 2.04 (d, *J* = 2.2 Hz, 3H, CH_2_–piperidine, CH–piperidine), 1.86 (d, *J* = 12.7 Hz, 5H, 2 × CH_2_–piperidine, CH–piperidine), 1.54–1.45 (m, 3H, CH_2_–piperidine, CH–piperidine); ^13^C NMR (101 MHz, chloroform-*d*): δ 170.3, 160.1, 140.9, 132.4, 130.8, 130.3, 129.8, 128.9, 128.1, 127.3, 114.2, 72.3, 54.8, 54.2, 49.7, 45.5, 34.9, 28.3, 22.8; HRMS (ESI^+^) for C_26_H_33_Cl_2_N_3_O_2_ ([M + H]^+^): calcd, 490.20226; found, 490.20139; HPLC: *t*_R_ = 3.05 min (96.8% at 254 nm).

### Conformation of Compound **96** from NMR Experiments

Conformation of **96** was calculated in Schrodinger Suite using distance constraints from trNOESY experiments (2.5 ± 0.5 Å for strong NOEs). For conformational search, the systematic torsional sampling method with default settings was used. Generated conformations were compared to the ligand conformations from MD trajectory, the and rmsd value was calculated.

### Expression and Purification of the Full-Length Hsp90α and Hsp90β Proteins

The plasmids for Hsp90α/Hsp90β protein expression were a kind gift from dr. Asta Zubriené, Institute of Biotechnology, Vilnius University, Lithuania. Hsp90 with N-terminal 6× His-tag was expressed in *Escherichia coli* strain BL21 (DE3). Cells were grown in TB media at 37 °C, and protein expression was achieved by induction with 0.5 mM IPTG at OD_600_ = 0.8, followed by incubation at 18 °C for 18 h. Cells were harvested by centrifugation, resuspended in lysis buffer [40 mM potassium phosphate pH 8.0, 400 mM KCl, 10 mM imidazole, protease inhibitors (Sigma)], and lysed by sonication. After centrifugation, proteins were first purified with a Ni^2+^-affinity HisTrap column (GE Healthcare). Impurities were washed with lysis buffer containing 20–40 mM imidazole and Hsp90 was eluted with lysis buffer containing 300 mM imidazole. The second purification was performed by SEC with a Superdex-200 (16/600) column (GE Healthcare) and running buffer (50 mM Tris pH 7.5 at RT, 300 mM KCl). Fractions were checked for purity by SDS-PAGE and concentrated. Hsp90 was then dialyzed against NMR buffer (50 mM KPO_4_ pD 7.5, 100 mM KCl, 1 mM DTT (98%, D10) in D_2_O) and frozen in liquid nitrogen.

#### Expression and Purification of the Hsp90α and Hsp90β N-Terminal Domains

The N-terminal domain of Hsp90α and Hsp90β (Hsp90αN and Hsp90βN) encoding plasmids was constructed by inserting the DNA sequences encoding the N-terminal domain of human Hsp90α (corresponding to amino acids 1–241) and Hsp90β (corresponding to amino acids 1–239), respectively, into the pET21b vector (Novagen, Madison, WI, USA). All resulting protein constructs include an N-terminal 6× His-tag with a thrombin cleavage site. Hsp90β NTD was then expressed in *E. coli* BL21 (DE3) strain. Bacterial cultures transformed by the plasmid were grown in shaker flasks in LB media supplemented with ampicillin until OD_600_ of 0.6 at 37 °C. Then, temperature was reduced to 30 °C, and target protein expression was induced by the addition of 1 mM IPTG. Four hours postinduction, bacteria were centrifuged and resuspended in buffer that comprised of 25 mM Tris–HCl, 100 mM NaCl, 100 mM imidazole, pH 7.5. The bacteria were lysed by sonication. Protein was purified from the soluble fraction using a Ni-IDA immobilized metal affinity column (Cytiva), followed by Q-Sepharose anion-exchange column (Cytiva). SDS-PAGE analysis determined protein purity to be higher than 95%. Protein concentrations were determined by UV–vis spectrophotometry. Proteins were dialyzed against storage buffer (50 mM Hepes, 100 mM NaCl, pH 7.5).

#### Nuclear Magnetic Resonance

The ^1^H STD and tr-NOESY experiments were recorded on a Bruker Avance Neo 600 MHz spectrometer with a cryoprobe at 25 °C using the pulse sequences included in the Bruker TopSpin library of pulse programs. Samples contained 1.5 μM Hsp90 and 0.3 mM AMP-PCP in NMR buffer. The ligands were dissolved in DMSO-*d*_6_ and added to the samples at a ligand/Hsp90 ratio of 200:1. The final concentration of DMSO-*d*_6_ in the samples was 2%.

The ^1^H STD ligand epitope mapping experiments^65^ were performed under quantitative conditions, considering the nonuniform relaxation properties of the ligands. The inversion–recovery T_1_ experiments showed that the ^1^H T_1_ relaxation times of the ligands ranged from 2.5 s for the aromatic ring protons to 0.4 s for the CH_2_ protons. Therefore, the STD amplification factors were determined with a short saturation delay of 0.2 s to avoid the effects of the longitudinal relaxation rate on the signal intensities.^54^ Spectra were acquired with a spectral width of 5882 Hz, 16384 data points, a relaxation delay of 5 s, and 2880 scans. The on-resonance selective saturation of Hsp90 was applied at −0.83 ppm at transmitter offset referenced to 4.70 ppm. The off-resonance irradiation was applied at 30 ppm for the reference spectrum. The spectra were zero-filled twice and apodized with an exponential line-broadening function of 3 Hz. Errors in the STD amplification factor were estimated according to the formula: STD amplification factor absolute error = STD amplification factor × ((*N*_STD_/*I*_STD_)^2^ + (*N*_REF_/*I*_REF_)^2^)^1/2^.^66^*N*_STD_ and *N*_REF_ are noise levels in STD and reference spectra. *I*_STD_ and *I*_REF_ are signal intensities in STD and reference spectra.

The tr-NOESY spectra^67^ were acquired with spectral width of 5882 Hz, 4096 data points in *t*_2_, 64 scans, 200 complex points in *t*_1_, a mixing time of 150 ms, and a relaxation delay of 1.5 s. The spectra were zero-filled twice and apodized with a squared sine bell function shifted by π/2 in both dimensions. Distances were calculated from cross-peak volumes using the integrated intensity of a pair of protons H11 and H12 in the 3,4-dichlorophenyl ring assumed to have a distance of 2.5 Å.

### Hsp90 C-Terminal Domain TR-FRET Assay

The activity of the Hsp90α and Hsp90β C-terminal domains was determined using the Hsp90α and Hsp90β CTD TR-FRET kit (BPS Bioscience; San Diego, USA). Each sample consisted of a terbium-labeled donor (5 μL), a dye-labeled acceptor (5 μL), the Hsp90α or Hsp90β C-terminal domain (2 ng/μL, 3 μL) and cyclophilin D (PPID) (3 ng/μL, 5 μL), and the test compounds (2 μL). The positive control contained all reagents except the inhibitors, while the negative control lacked the target protein PPID. The maximum DMSO concentration was 1%. The reaction mixture was incubated for 2 h at room temperature. We then analyzed the interaction between the C-terminal domain of Hsp90 and PPID by TR-FRET measurement using Tecan’s Spark Multimode Microplate reader (Tecan Trading AG, Switzerland). Each sample was run in triplicate. To quantify the activity of the Hsp90 C-terminal domain, the following formula was used % activity = 100 × (FRET sample – FRET negative control)/(FRET positive control – FRET negative control), where the value FRET represents the ratio between the dye-labeled acceptor emission and the terbium-labeled donor emission.

### Cell Culture

Hormone positive breast cancer cell lines MCF-7 (ATCC-HTB-22; ATCC) and T47D (ATCC-HTB-133; ATCC) were cultured in Dulbecco’s modified Eagle’s MEM medium and RPMI-1640 medium (Sigma-Aldrich, St. Louis, MO, USA), respectively. Cell culture medium for T47D cell line was supplemented with 0.2 units/mL of insulin (Sigma-Aldrich, St. Louis, MO, USA). HER2 overexpressing cell line SKBr3 (ATCC-HTB-30; ATCC) was cultured in McCoy’s 5A medium (Sigma-Aldrich, St. Louis, MO, USA). Triple negative breast cancer cell line MDA-MB-231 (ATCC-HTB-26; ATCC) was cultured in RPMI-1640 medium (Sigma-Aldrich, St. Louis, MO, USA). The PC3MM2 cell line was cultured in Dulbecco’s modified Eagle DMEM, high glucose medium (Sigma-Aldrich, St. Louis, MO, USA). All cell culture mediums were supplemented with 10% heat inactivated fetal bovine serum (Gibco, Thermo Fisher Scientific, Waltham, MA, USA), 100 U/mL penicillin (Sigma-Aldrich, St. Louis, MO, USA), 100 μg/mL streptomycin (Sigma-Aldrich, St. Louis, MO, USA), and 2 mM l-glutamine (Sigma-Aldrich, St. Louis, MO, USA). All cell lines were incubated in a 5% CO_2_ atmosphere at 37 °C.

### Cell Viability Assay

The antiproliferative activity of the compounds was assessed against MCF-7 (ATCC-HTB-22; ATCC), SKBr3 (ATCC-HTB-30; ATCC), MDA-MB-231(ATCC-HTB-26; ATCC), and T47D (ATCC-HTB-133; ATCC) breast cancer cell lines using an MTS antiproliferation assay (Promega, Madison, WI, USA). The cells were seeded in 96-well plates (5 × 10^4^ cells/mL) in 100 μL of growth medium and allowed to attach. After incubating the cells for 72 h in the presence of the test compounds, a positive control (17-DMAG), or a vehicle control (0.5% DMSO), CellTiter96 Aqueous One Solution Reagent (Promega, Madison, WI, USA) was added to each well. Following a 3 h incubation period, the absorbance was measured at 492 nm using BioTek’s Synergy 4 Hybrid Microplate Reader (Winooski, VT, USA). Each experiment was performed in triplicates. The IC_50_ values (concentration of the inhibitor that gives a half-maximal response) are the average values from three independent repeats and were determined using GraphPad Prism 9.5.0 software (San Diego, CA, USA).

### Apoptosis Assay

Apoptosis assay was carried out using the Annexin V FITC/PI Cell apoptosis kit (Invitrogen, Thermo Fisher Scientific, Waltham, MA, USA). MDA-MB-231 cells were seeded (5 × 10^4^ cells/well) in 12-well plates and treated with compounds **89** (2.5 and 10 μM), **104** (2.5 and 10 μM), or vehicle control (0.5% DMSO) for 72 h. After the incubation period, cells were collected, washed with PBS, and centrifuged. Cell pellet was resuspended in 1× binding buffer with Annexin V FITC. After 15 min, PI (100 μg/mL) was added. Stained cells were analyzed by flow cytometry (Attune NxT flow cytometer, Thermo Fisher Scientific, Waltham, MA, USA) and FlowJo software (Tree Star Inc., Ashland, OR, USA).

### CFSE Proliferation Assay

The proliferation status of MDA-MB-231 cells was conducted using CFSE staining. MDA-MB-231 cells were stained with the 2 μM CellTrace CFSE cell proliferation kit (Invitrogen, Thermo Fisher Scientific, Waltham, MA, USA) in PBS for 20 min at 37 °C. Complete culture medium was added to the cells to remove residual CFSE. Cells were then washed and seeded (5 × 10^4^ cells/well) in 12-well plates. Cells were treated with compounds **89** (2.5 and 10 μM), **104** (2.5 and 10 μM), or vehicle control (0.5% DMSO) for 48 and 72 h. After the desired incubation period, cells were collected and analyzed by flow cytometry (Attune NxT flow cytometer, Thermo Fisher Scientific, Waltham, MA, USA) and FlowJo software (Tree Star Inc., Ashland, OR, USA).

### Western Blot

The MDA-MB-231, MCF-7, and SKBr3 cells were treated with two concentrations of compounds **89** and **104** (5 μM and 20 μM), 0.5 μM 17-DMAG, or 0.5% DMSO and incubated for 24 h. After incubation, cells were washed with 1× DPBS (Gibco, Thermo Fisher Scientific, Waltham, MA, USA), lysed with RIPA buffer (50 mM Tris–HCl pH 7.4, 150 mM NaCl, 1% NP −40, 0.5% sodium deoxycholate, 1 mM EDTA), and supplemented with 1:100 Halt Protease Inhibitor Cocktail (Thermo Fisher Scientific, Waltham, MA, USA) and 1:100 Halt Protease Phosphatase Inhibitor Cocktail (Thermo Fisher Scientific, Waltham, MA, USA). The resulting cell lysates were sonicated and then centrifuged at 15,000 rpm for 20 min at 4 °C, and the supernatants obtained were collected. For protein quantification, the DC protein assay (Bio-Rad, Hercules, California, USA) was performed and the eSDS PAGE was performed using equal amounts of protein (20 μg) on a 10 or 7% (for Her2) acrylamide/bis(acrylamide) gel. Electrophoresis was performed at 80 V for 15 min and then at 130 V for 60 min, followed by transfer to a PVDF membrane using the iBlot3 Dry Blotting System (Thermo Fisher Scientific, Waltham, MA, USA). Nonspecific binding sites were blocked with 5% BSA for 1 h at room temperature, followed by for 18 h incubation at 4 °C with primary antibodies against Hsp90 Rabbit mAb (1:1000), Hsp70 Mouse mAb (1:1000), cRAF Rabbit mAb (1:1000), GAPDH Rabbit mAb (1:2500), AKT Rabbit mAb (1:1000), phospho-AKT rabbit mAb (1:1000), phospho-MEK rabbit mAb (1:1000), MEK rabbit mAb (1:1000), phospho-ERK mouse mAb (1:1000), ERK mouse mAb (1:1000), CDK4 rabbit mAb (1:1000), ERα rabbit mAb (1:1000), Her2 rabbit mAb (1:1000), β-tubulin rabbit mAb (1:5000), and β-actin mouse mAb (1:5000) (all antibodies from Cell Signaling, Danvers, MA, USA). After washing, membranes were incubated with secondary antibodies (antirabbit IgG, HRP-linked antibody at 1:5000 dilution and antimouse IgG, HRP-linked antibody at 1:5000 dilution) for 1 h at room temperature. To visualize the membranes, the SuperSignal West Femto Maximum Sensitivity Substrate (Thermo Fisher Scientific, Waltham, MA, USA) was added and the membranes were imaged using the UVITEC Cambridge Imaging System (UVITEC, Cambridge, UK). Quantitative densitometric analysis of Western blot bands was performed using Image Lab software (Bio-Rad, Hercules, California, USA). The adjusted relative densities were calculated with respect to the loading control GAPDH, β-actin or β-tubulin.

### In Vivo Efficacy Study in Mice

In vivo efficacy evaluation of **89** in MDA-MB-468 human cancer xenograft model was carried out by Wuxi AppTec (Shanghai, China). The study included 18 (6 per group) female BALB/c nude mice (*Mus musculus*) supplied to Wuxi AppTec by Zhejiang Vital River Laboratory Animal Technology Co., Ltd. Each mouse was inoculated subcutaneously at the right flank with MDA-MB-468 tumor cells (10 × 10^6^) in 0.2 mL of PBS supplemented with Matrigel (1:1) for tumor development. Treatments were started on day 22 after tumor inoculation when the average tumor size reached approximately 165 mm^3^. The animals were assigned into groups using an Excel-based randomization software performing stratified randomization based upon their tumor volumes. Each group consisted of 6 tumor-bearing mice. Mice were administered **89** (100 mg/kg day 0 to day 18, 50 mg/kg day 19 to day 28), positive control AUY922 (50 mg/kg day 0 to day 18, 25 mg/kg day 19 to day 28) or vehicle intravenously three times per week for 4 weeks. Tumor size and body weight were measured every 2 days. All the procedures related to animal handling, care, and the treatment in the study were performed according to the guidelines approved by the Institutional Animal Care and Use Committee (IACUC) of WuXi AppTec following the guidance of the Association for Assessment and Accreditation of Laboratory Animal Care (AAALAC).

#### Microscale Thermophoresis

The full-length Hsp90β was labeled with the Monolith His-Tag Labeling Kit RED-tris-NTA according to the manufacturers labeling instructions (NanoTemper Technologies GmbH, Munich, Germany). The protein was first diluted to 8 nM concentration by the assay buffer (50 mM Tris–HCl, pH 7.4 containing 150 mM NaCl, 5% EtOH and 10 mM MgCl_2_). To determine the *K*_d_ values, the protein was mixed with the ethanol solution of the compound in question in a ratio 1:1. The final concentrations of Hsp90β (4 nM) and compound (2000–23.4 μM for **104**, 5–0.00031 mM for novobiocin) were determined. At higher concentrations of the compounds, aggregation was observed due to insufficient solubility; therefore, these measurements were disregarded (MST curves colored in gray). The compound was incubated with the protein for 15 min in the dark at room temperature. The mixtures were then inserted into Monolith NT.115 Premium Capillaries (NanoTemper Technologies GmbH, Munich, Germany). Thermophoresis of each mixture was induced at 1475 ± 15 nm and measured using a Monolith NT.115 pico instrument (NanoTemper Technologies GmbH, Munich, Germany). The temperature of the measurement was kept at ambient temperature (24–25 °C), the excitation power was set to 20%, while the MST power was set to 40% with 5 s laser on time). Two independent *K*_d_ determinations were performed. The average fluorescence responses for each concentration were then plotted against the logarithm of compound concentration using GraphPad Prism software (GraphPad Software, Inc. La Jolla, CA).

#### Fluorescence-Based Thermal Shift Assay with the Hsp90α and Hsp90β N-Terminal Domains

Compound binding to Hsp90α and Hsp90β N-terminal domains was determined by the fluorescence-based thermal shift assay (FTSA) which determines the thermal stability of the free and ligand-bound protein. The experiments were performed using Rotor-Gene Q 6-Plex spectrofluorimeter (excitation 365 nm, detection 460 nm). Solutions containing 10 μM of protein and various concentrations of ligand (0–500 μM) were heated up from 25 to 80 °C at a rate of 1 °C/min. Protein unfolding was detected using 8-anilino-1-naphthalenesulfonate fluorescent dye at 100 μM concentration. Experiments were carried out in a buffer composed of 50 mM sodium phosphate, 100 mM sodium chloride, 2% DMSO, pH 7.5. Fitting of melting curves (*T*_m_ values) were performed using Thermott.^68^

### Luciferase Refolding Assay

Luciferase refolding assay was carried out in PC3MM2luc cells expressing firefly luciferase. Cells were grown to 80% confluency and then harvested. Cell pellets were suspended in prewarmed medium (50 °C) for 2 min to induce firefly luciferase unfolding. The cells were plated in 96-well plates at a density of 50,000 cells per well in the presence of selected compounds, vehicle control (1% DMSO), or positive control (50 μM 17-DMAG). The plates were incubated for 60 min at 37 °C to allow for luciferase refolding. After incubation 100 μL of ONE-Glo Luciferase Assay System (Promega, Madison, WI, USA) was added to each well of the plate and incubated for another 5 min. Luciferase activity was determined by measuring luminescence with Tecan’s Spark Multimode Microplate reader (Tecan Trading AG, Switzerland). Independent experiments were repeated two times, each performed in triplicate. IC_50_ values (concentration of the inhibitor that gives a half-maximal response) are given as average values from the independent measurements, and were determined using GraphPad Prism 9.2.0 software (San Diego, CA, USA).

## Supporting Information Available

Representative IC_50_ curves from MTS assay; molecular modeling; proliferation of untreated MDA-MB-231 cells; 1D ^1^H STD NMR spectra; trNOESY spectra; determination of MTD in BALB/c nude mice; representative ^1^H and ^13^C NMR spectra; representative HPLC and UPLC chromatograms; Western blot images used for quantification; kinase profiling (PDF)
Structure of Hsp90β–**89** complex (PDB)
Structure of Hsp90β–**96** complex (PDB)
Structure of Hsp90β–**104** complex (PDB)
Structure of Hsp90α–**96** complex (PDB)
SMILES molecular formula strings (CSV)

## Supplementary Material

jm4c00932_si_001.pdf

jm4c00932_si_002.pdb

jm4c00932_si_003.pdb

jm4c00932_si_004.pdb

jm4c00932_si_005.pdb

## Abbreviations

AKT: protein kinase B
AMP-PCP: adenylylmethylenediphosphonate
BALB/c: Bagg albino laboratory-bred
CFSE: carboxyfluorescein succinimidyl ester
CTD: C-terminal domain
dd: double doublet
DIAD: diisopropyl azodicarboxylate
DIPEA: *N*-ethyldiisopropylamine
EDC: 1-ethyl-3-carbodiimide hydrochloride
Erα: estrogen receptor α
FITC: fluorescein isothiocyanate
FTSA: fluorescence-based thermal shift assay
GAPDH: glyceraldehyde 3-phosphate dehydrogenase
HER2: human epidermal growth factor receptor 2
HOBt: hydroxybenzotriazole
HPLC–MS: liquid chromatography–mass spectrometry
HSP27: heat shock protein 27
HSP70: heat shock protein 70
HSP90: heat shock protein 90
HSR: heat shock factor
IPTG: isopropyl β-d-1-thiogalactopyranoside
*K*_d_: dissociation constant
mAb: monoclonal antibody
MEK: mitogen-activated protein kinase
MeOH: methanol
MFI: mean fluorescence intensity
MST: microscale thermophoresis
MTD: maximum tolerated dose
mTOR: mammalian target of rapamycin
MTS: 3-(4,5-dimethylthiazol-2-yl)-5-(3-carboxymethoxyphenyl)-2-(4-sulfophenyl)-2*H*-tetrazolium
Ni-IDA: nickel ion coupled to iminodiacetic acid
NMM: *N*-methylmorpholine
NTD: N-terminal domain
PI: propidium iodide
PPID: protein cyclophilin D
RAF: serine/threonine-specific protein kinases
RIPA: radio-immunoprecipitation assay
rmsd: root-mean-square deviation
SBPM: structure-based pharmacophore model
SD: standard deviation
SDS-PAGE: sodium dodecyl sulfate–polyacrylamide gel electrophoresis
SEM: standard error of the mean
STAT3: signal transducer and activator of transcription 3
STD: saturation transfer difference
TB: terrific broth
TNBC: triple negative breast cancer
TR-FRET: time-resolved fluorescence energy transfer
trNOESY: transferred nuclear overhauser enhancement spectroscopy
