# Supplementary material for: New Class of Hsp90 C-Terminal Domain Inhibitors with Anti-tumor Properties against Triple-Negative Breast Cancer
Source: J Med Chem. 2024 Jul 23;67(15):12984–3018. doi: 10.1021/acs.jmedchem.4c00932 (PMC11320583; doi:10.1021/acs.jmedchem.4c00932)
Supplement: Supplementary file 1 — jm4c00932_si_001.pdf [file jm4c00932_si_001.pdf]

Supporting Information for

New Class of Hsp90 C-Terminal Domain Inhibitors with Anti-tumor Properties against Triple-Negative Breast Cancer

Živa Zajec,[a] Jaka Dernovšek,[a] Jernej Cingl,[a] Iza Ogris,[b] Marius Gedgaudas,[c] Asta Zubrienė,[c] Ana Mitrović,[d] Simona Golič Grdadolnik,[b] Martina Gobec,[a] and Tihomir Tomašič[a,*]

[a]*Faculty of Pharmacy, University of Ljubljana, Aškerčeva cesta 7, 1000 Ljubljana, Slovenia*

[b]*Laboratory for Molecular Structural Dynamics, Theory Department, National Institute of Chemistry, Hajdrihova 19, 1001 Ljubljana, Slovenia*

[c]*Department of Biothermodynamics and Drug Design, Institute of Biotechnology, Life Sciences Center, Vilnius University, Saulėtekio al. 7, LT-10257 Vilnius, Lithuania*

[d]*Department of Biotechnology, Jožef Stefan Institute, Jamova 39, 1000, Ljubljana, Slovenia*

*****Corresponding author email address: tihomir.tomasic@ffa.uni-lj.si

Contents

1.1 Representative IC_{50} curves ... 3

1.2 1D ^{1}H STD NMR spectra ... 4

1.3 trNOESY spectra ... 9

1.4 Studying the binding mode of representative Hsp9 CTD inhibitors by molecular modeling ... 15

1.5 Molecular Docking ... 17

1.6 Molecular dynamics simulations ... 18

1.7 Structure-based pharmacophore modeling ... 18

1.8 Representative ^{1}H and ^{13}C NMR spectra ... 19

1.9 Representative HPLC and UPLC chromatograms ... 86

1.10 WB images used for quantification ... 121

1.11 Proliferation of untreated MDA-MB-231 cells ... 142

1.12 Determination of MTD in BALB/c nude mice ... 142

1.13 Kinase profiling ... 143

1.14 Western blot analysis of MCF-7 cells when co-treated with **89** or **104** and carfilzomib ... 144

1.1 Representative IC_{50} curves

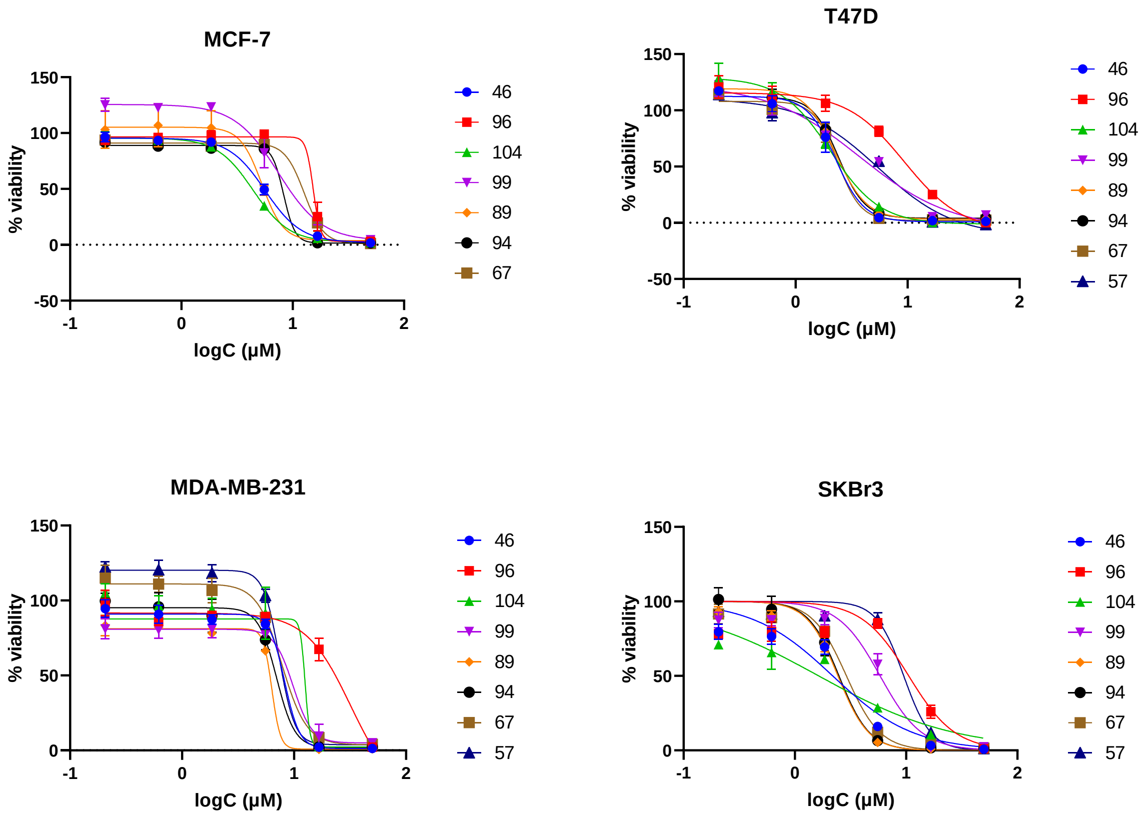

Figure S1. Representative IC_{50} curves on different breast cancer cell lines after 72 h incubation with compounds.

Table S1. IC_{50} values of representative compounds on different breast cancer cell lines. IC_{50} values are presented as mean ± SD of three independent experiments.

Compound	IC_{50} (μM)			
	MCF-7	SKBr3	T47D	MDA-MB-231
**46**	7.2 ± 0.6	10.5 ± 1.4	6.1 ± 0.8	12.7 ± 1.3
**57**	4.1 ± 2.0	3.0 ± 1.2	1.9 ± 0.6	5.6 ± 0.4
**67**	4.4 ± 1.1	3.3 ± 0.5	2.7 ± 0.5	7.4 ± 0.2
**89**	3.1 ± 0.7	2.5 ± 0.0	1.8 ± 0.8	5.3 ± 0.7
**94**	2.9 ± 1.0	2.4 ± 0.8	2.4 ± 0.0	6.4 ± 0.8
**96**	8.3 ± 0.6	12.2 ± 2.1	9.6 ± 0.3	15.3 ± 3.6
**99**	4.5 ± 2.8	4.6 ± 1.7	3.2 ± 0.7	8.2 ± 1.3
**104**	1.8 ± 0.5	2.5±0.8	1.6±0.6	4.4±0.8
**17-DMAG**	0.5 ± 0.1	0.2±0.0	0.2±0.0	0.3±0.0

1.2 1D ^{1}H STD NMR spectra

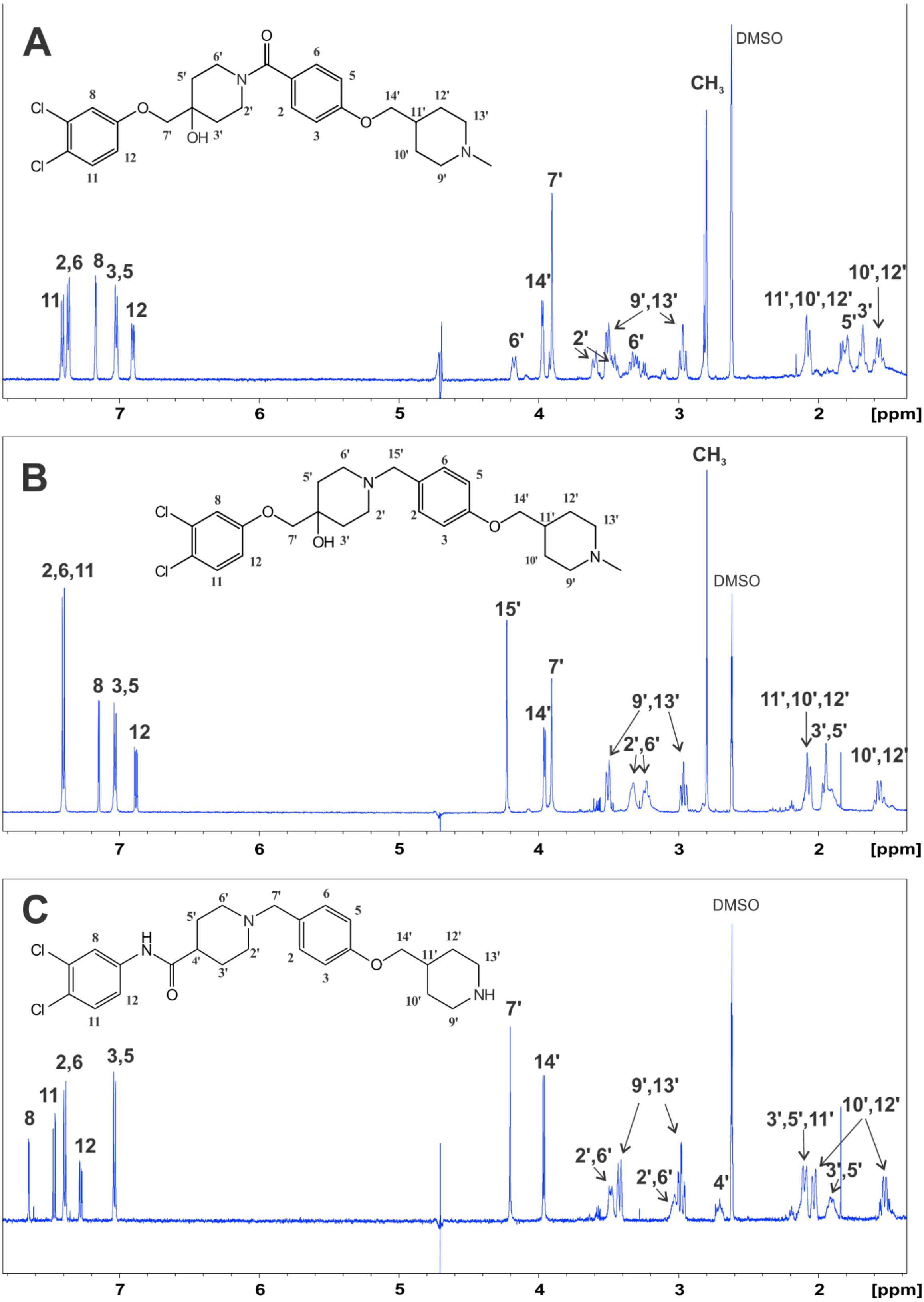

Figure S2. ^{1}H NMR spectra for compounds **96** (A)**, 104** (B) and **89** (C) recorded at 0.3 mM concentration in 50 mM KPO_4, 100 mM KCl, 5 mM $MgSO_4$, D_2O buffer, pD 7.5.

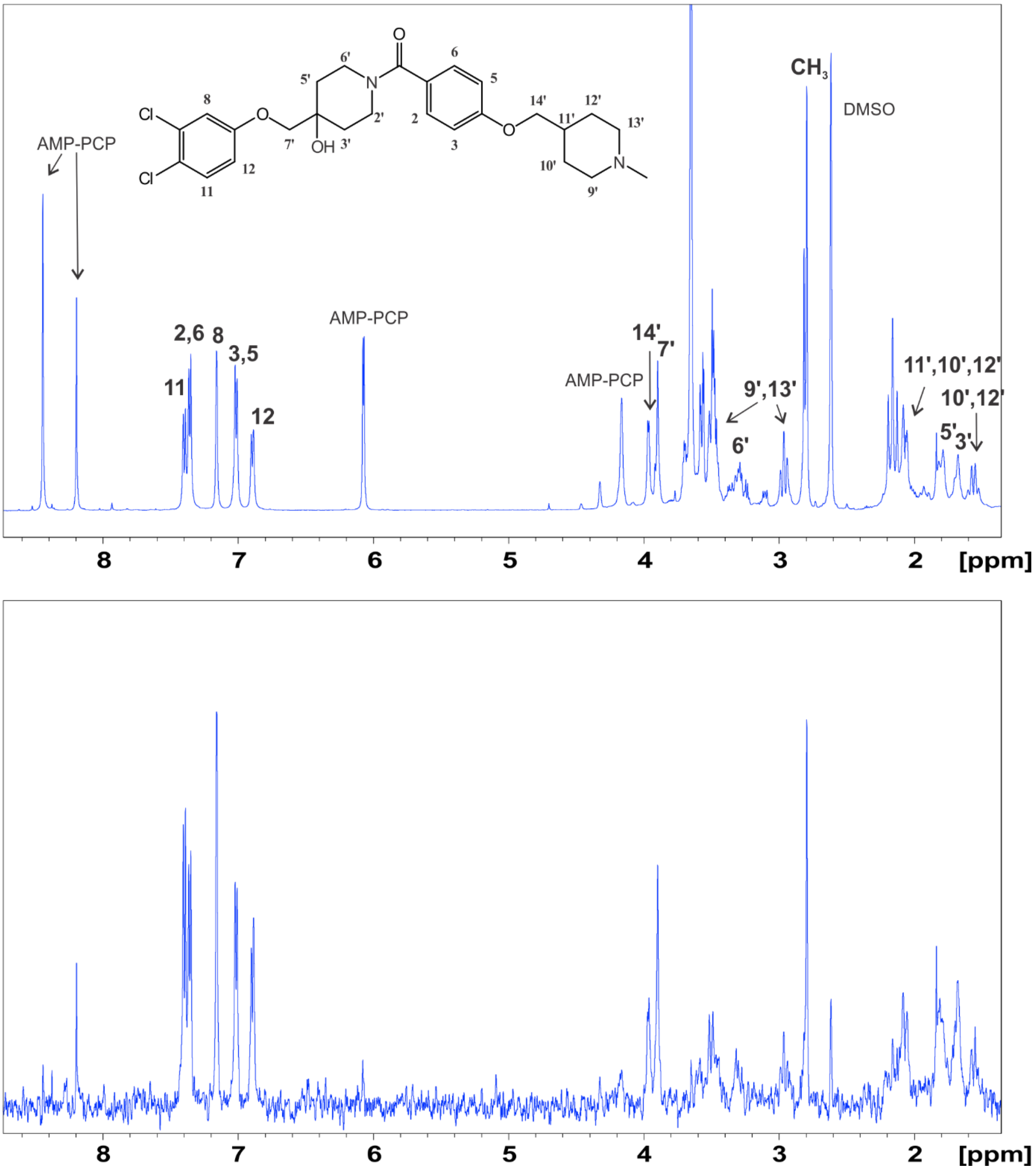

Figure S3. 1D ^{1}H STD NMR spectra for compound **96** recorded at a HSP90α:ligand ratio of 1:200. Molecular structure illustrates proton nomenclature. Reference STD spectra (top) with proton assignment and difference STD spectra (bottom) are shown. The unassigned proton signals between 3.5 and 3.8 ppm belong to glycerol. The signals of H2’ and the low-field protons H3’ and H9’ protons overlap with the signals of glycerol. The low-field signal of H6’ overlaps with the signal of AMP-PCP at 4.17 ppm. The spectra are not to scale.

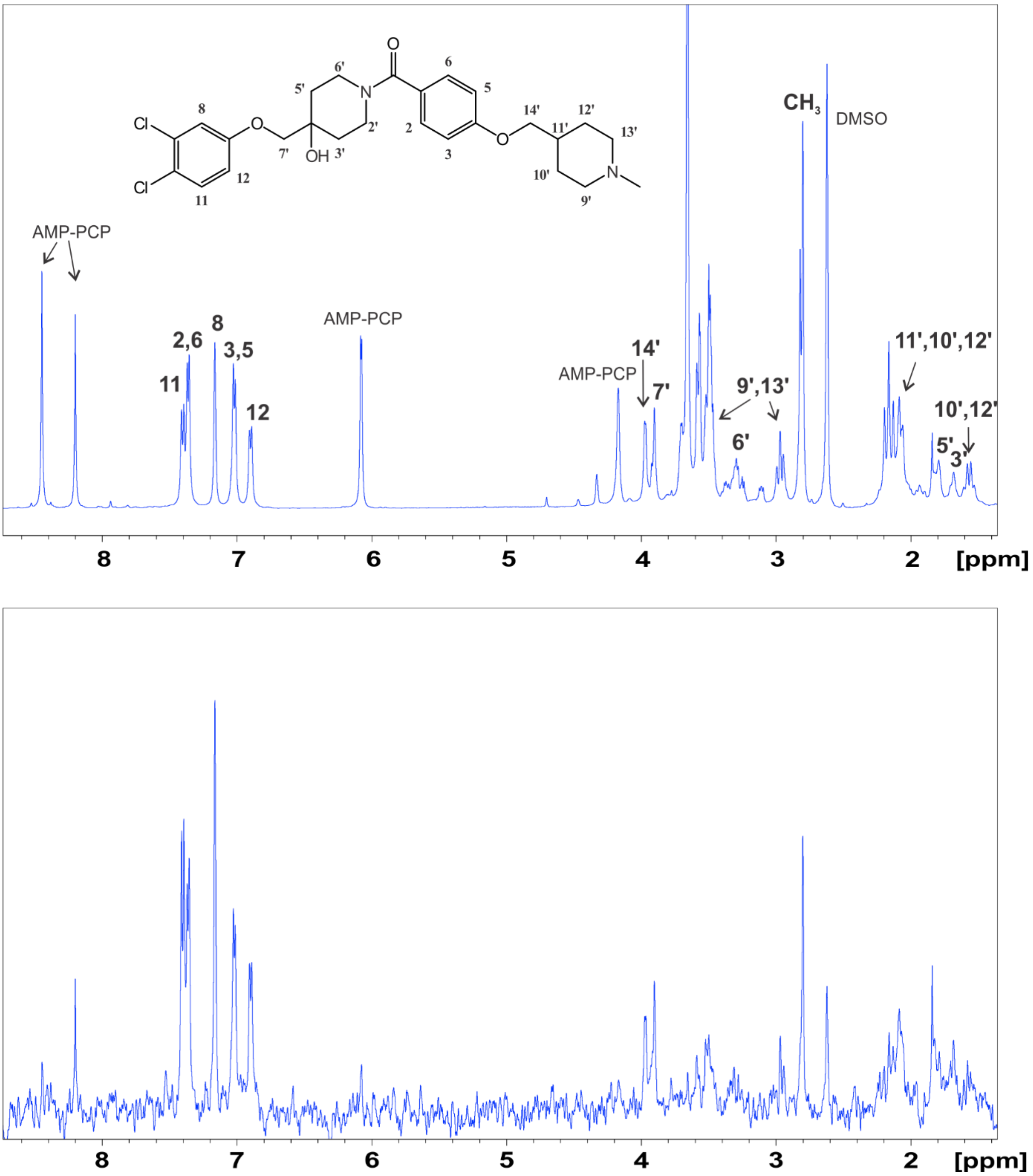

Figure S4. 1D ^{1}H STD NMR spectra for compound **96** recorded at a HSP90β:ligand ratio of 1:200. Molecular structure illustrates proton nomenclature. Reference STD spectra (top) with proton assignment and difference STD spectra (bottom) are shown. The unassigned proton signals between 3.5 and 3.8 ppm belong to glycerol. The signals of H2’ and the low-field protons H3’ and H9’ overlap with the signals of glycerol. The low-field signal of H6’ overlaps with the signal of AMP-PCP at 4.17 ppm. The spectra are not to scale.

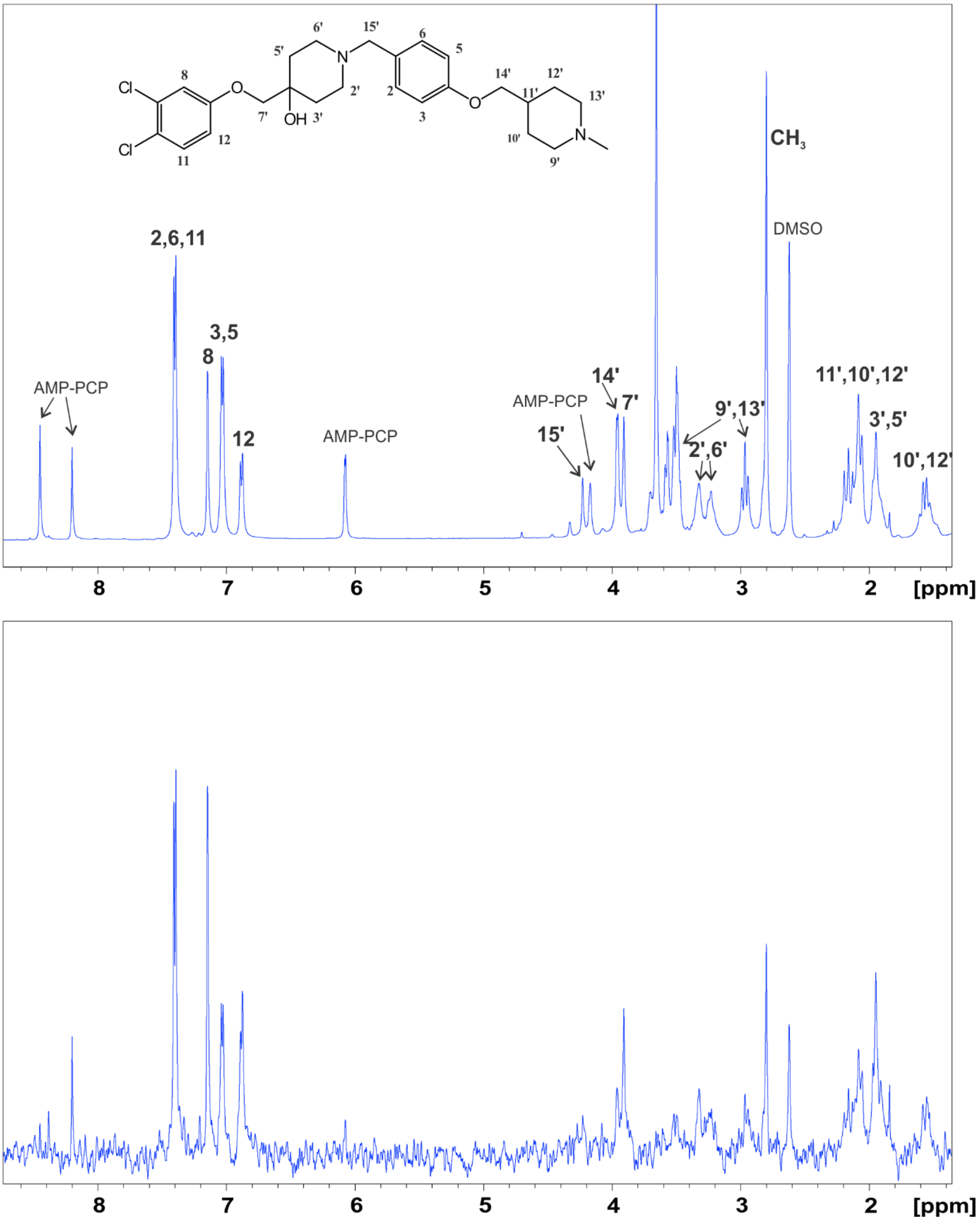

Figure S5. 1D ^{1}H STD NMR spectra for compound **104** recorded at a HSP90β:ligand ratio of 1:200. Molecular structure illustrates proton nomenclature. Reference STD spectra (top) with proton assignment and difference STD spectra (bottom) are shown. The unassigned proton signals between 3.5 and 3.8 ppm belong to glycerol. The signals of the low-field protons H3' and H9' overlap with the signals of glycerol. The spectra are not to scale.

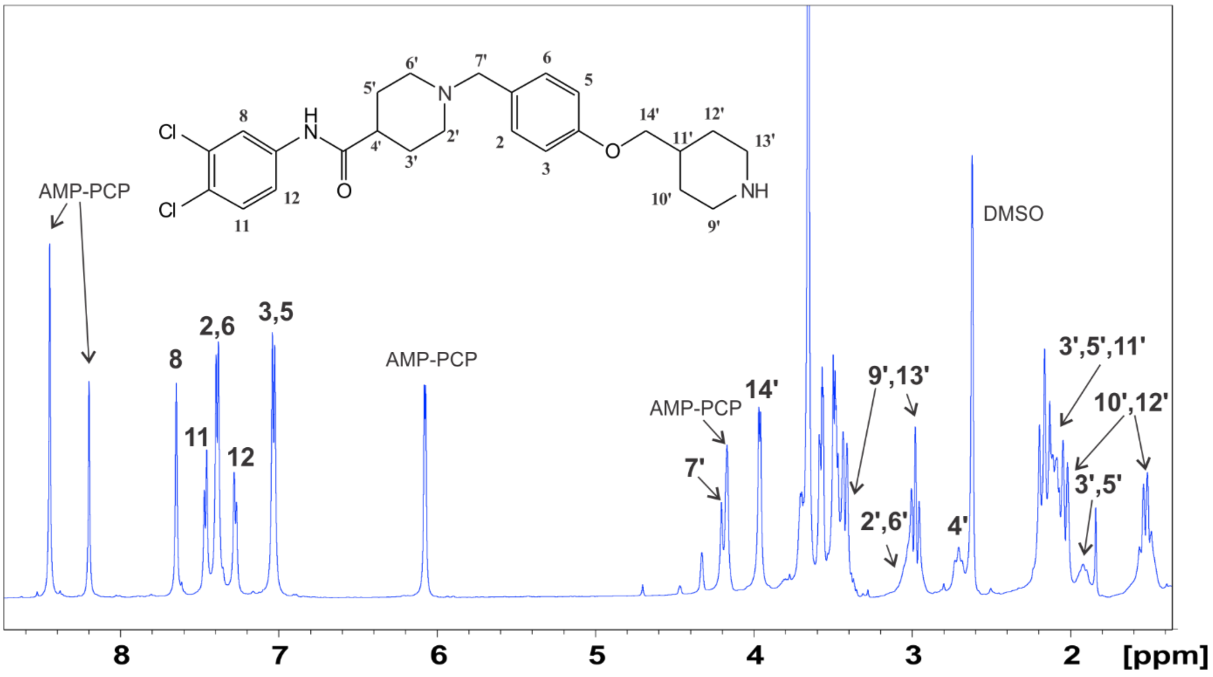

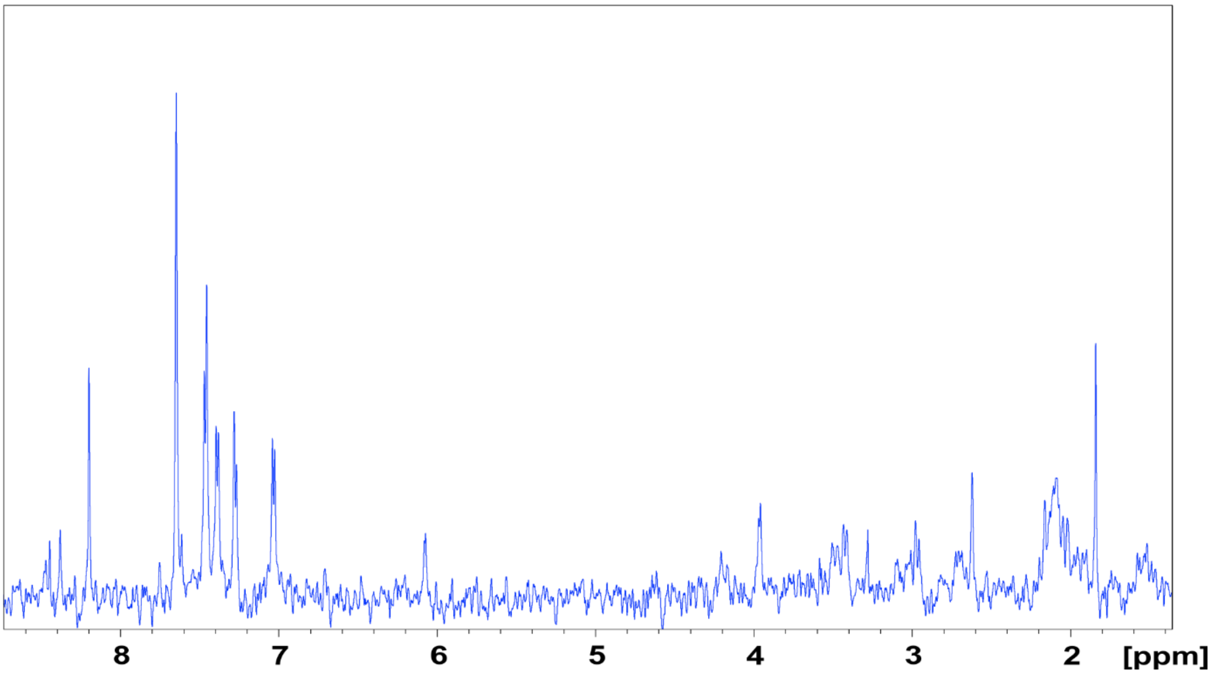

Figure S6. 1D ^{1}H STD NMR spectra for compound **89** recorded at a HSP90β:ligand ratio of 1:200. Molecular structure illustrates proton nomenclature. Reference STD spectra (top) with proton assignment and difference STD spectra (bottom) are shown. The unassigned proton signals between 3.5 and 3.8 ppm belong to glycerol. The signals of the low-field protons H3' and H9' overlap with the signals of glycerol. The spectra are not to scale.

1.3 trNOESY spectra

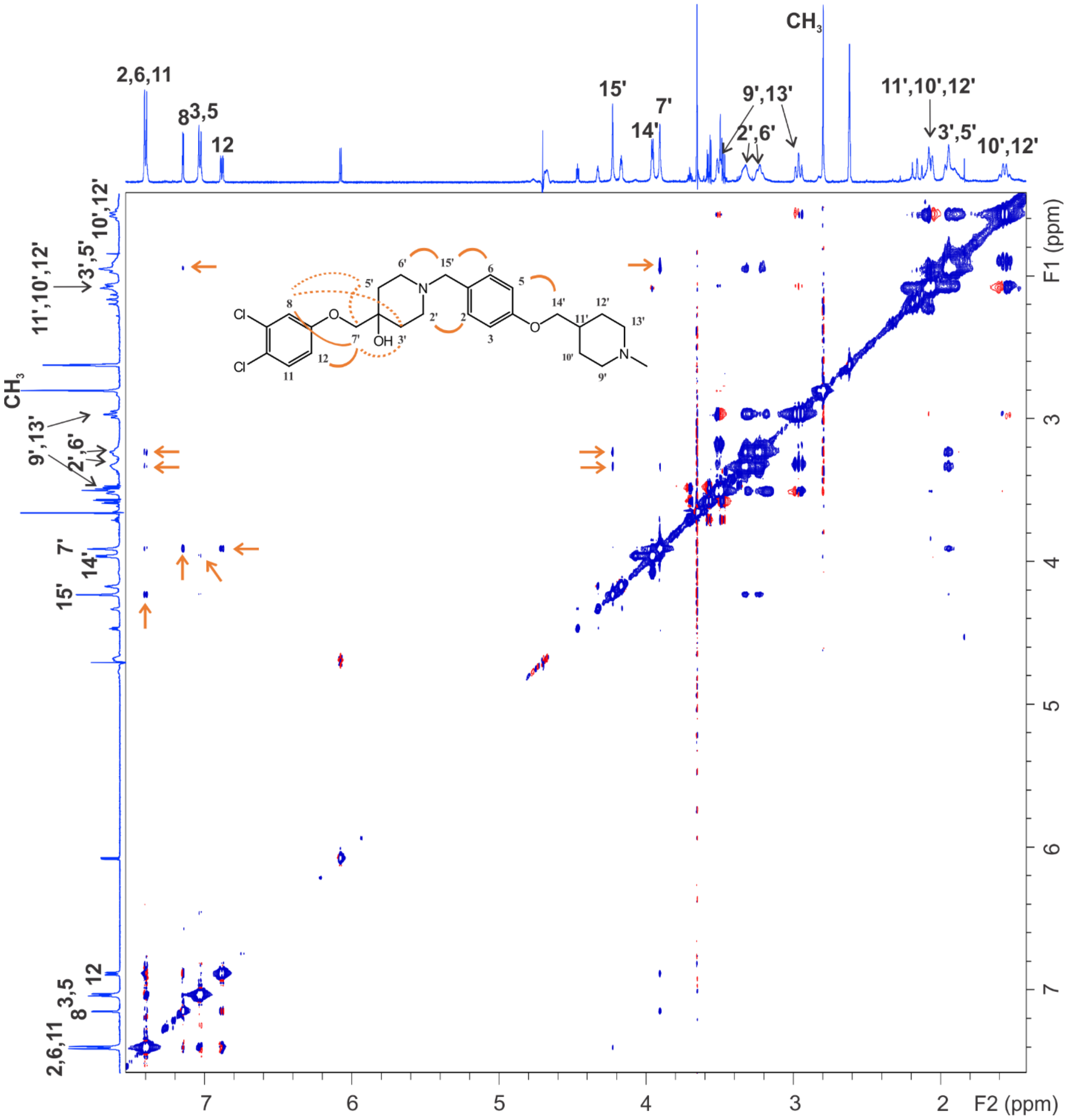

Figure S7. The trNOESY spectrum of **104** in the presence of Hsp90β with the molecular structure illustrating the atom nomenclature and the NOE connectivities between the protons of the different molecular segments. Corresponding NOEs are marked with arrows. The NOE connectivities of the magnetically equivalent protons 2,6 and 3,5 and protons 2' and 6' with overlapping signals are shown schematically for one orientation of the corresponding rings only. The connectivities of H8 and H7' with H3' and H5' are marked with dashed lines, indicating that due to the overlapping signals of the H3' and H5', it is not possible to distinguish whether these two protons have NOEs with both H3' and H5' or only with one of them, which was possible with compound **96**. The weak NOEs of H2,H6 with H14' and of H3,H5 with H15' are most likely a consequence of spin diffusion.

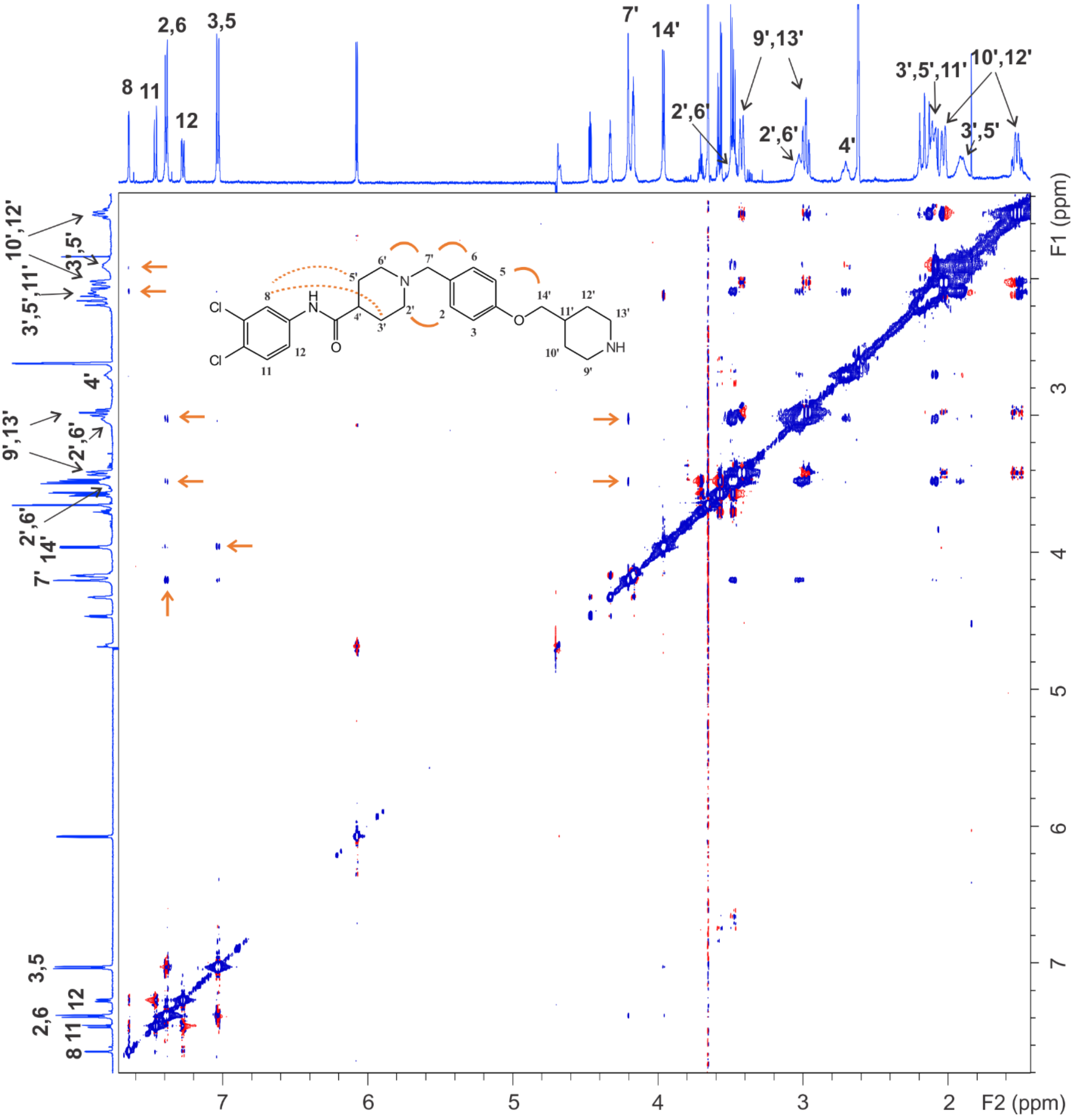

Figure S8. The trNOESY spectrum of **89** in the presence of Hsp90β with the molecular structure illustrating the atom nomenclature and the NOE connectivities between the protons of the different molecular segments. Corresponding NOEs are marked with arrows. The NOE connectivities of the magnetically equivalent protons 2,6 and 3,5 and protons 2' and 6' with overlapping signals are shown schematically for one orientation of the corresponding rings only. The connectivities of H8 are marked with dashed lines, indicating that due to the overlapping signals of the H3' and H5', it is not possible to distinguish whether this proton has NOEs with both H3' and H5' or only with one of them, as observed for compound **96**. The weak NOEs of H2,H6 with H14' and of H3,H5 with H7' are most likely a consequence of spin diffusion.

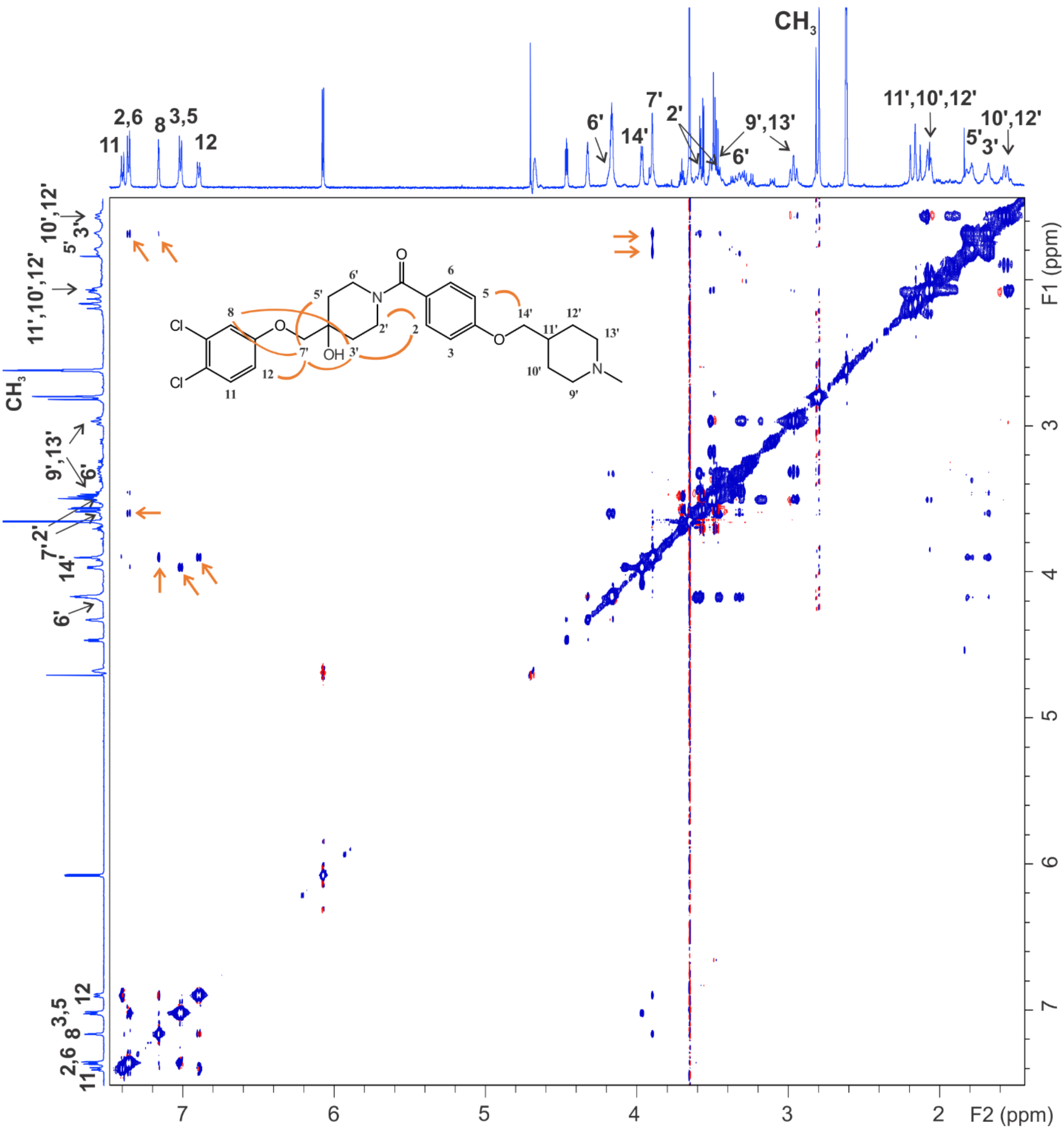

Figure S9. The trNOESY spectrum of **96** in the presence of Hsp90α with the molecular structure illustrating the atom nomenclature and the NOE connections between the protons of the different molecular segments. Corresponding NOEs are marked with arrows. Note that the NOE connectivities of the magnetic equivalent protons 2,6 and 3,5 are schematically shown only for one orientation of the corresponding aromatic ring. The weak non-marked NOESs between aromatic and aliphatic protons are most likely a consequence of spin diffusion.

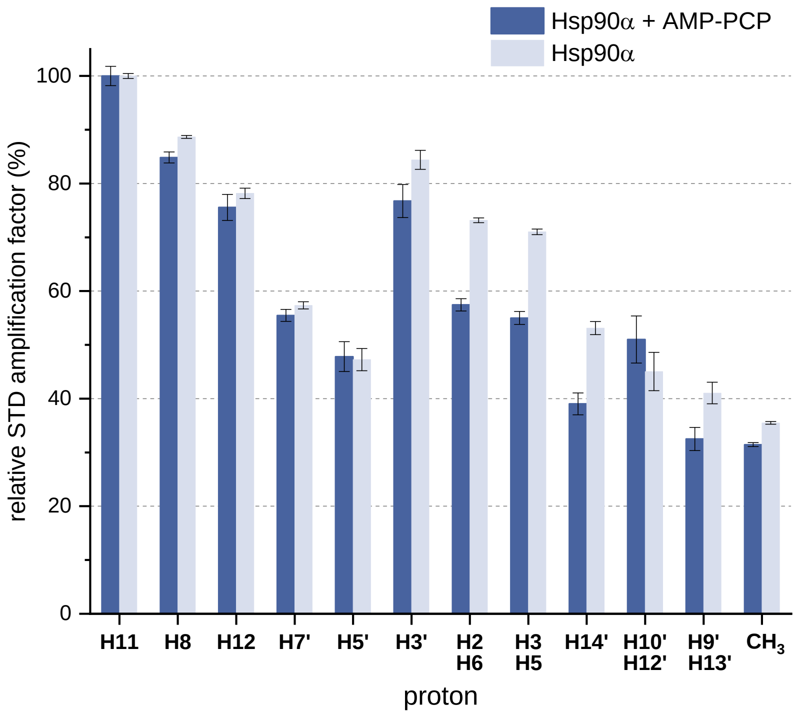

Figure S10. Binding epitope mapping by 1D ^{1}H STD NMR spectroscopy for compounds **96**, at a ratio Hsp90α:**96** of 1:200, with and without the presence of AMP-PCP.

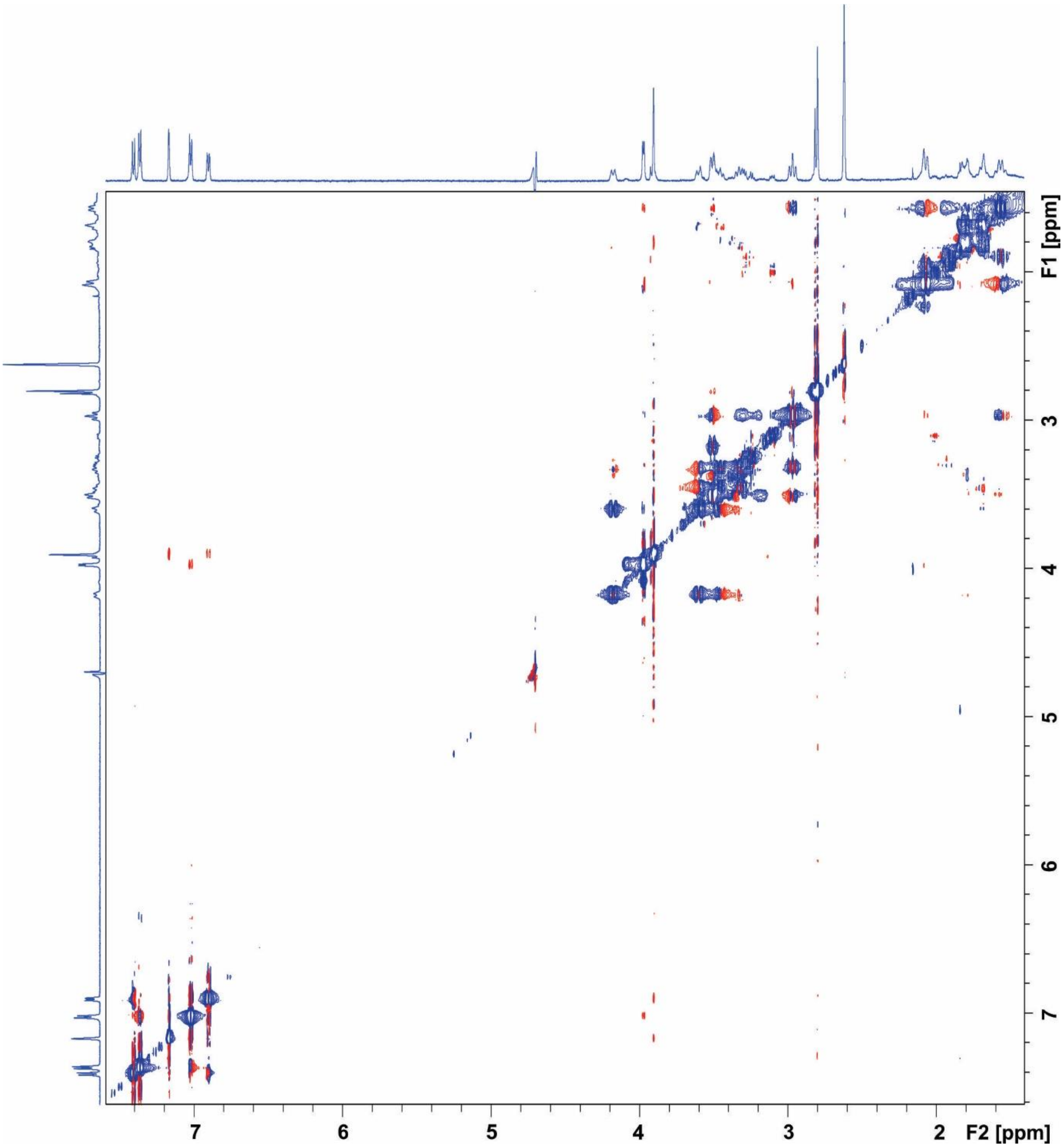

Figure S11. NOESY spectrum of compound **96** in the absence of Hsp90 in a D_2O buffer: 50 mM KPO_4 (pD 7.5), 100 mM KCl, and 2% DMSO-d_6. In contrast to the tr-NOESY spectrum of **96** in the presence of Hsp90β (Figure 7), the NOE signals between molecular segments are in antiphase with respect to the diagonal peaks, indicating fast motion of the free ligand.

Table S2: Relative STD amplification factors (AMP) and T_1 relaxation times for protons of compounds **96**, **104**, and **89**.

**96**			**104**			**89**		
**Proton**	**AMP (%)**	**T_1 (s)**	**Proton**	**AMP (%)**	**T_1 (s)**	**Proton**	**AMP (%)**	**T_1 (s)**
11	100	3.1	11		2.1	11	89	3.2
8	83	2.4	8	100	2.5	8	100	5.5
12	62	1.8	12	80	1.9	12	61	2.8
7'	45	0.3	7'	55	0.4			
						4'	39	0.6
5'	47	0.4	3',5'	78	0.4			
3'	76	0.4	3',5'	78	0.4			
			2',6'	55	0.4			
			15'	29	0.4	7'	21	0.4
2,6	53	2.0				2,6	29	1.4
3,5	48	1.6	3,5	38	1.5	3,5	25	1.5
14'	38	0.3	14'	25	0.5	14'	22	0.4
10'12'	36	0.7	10'12'	43	0.6	10'12'	22	0.6
9'13'	35	0.5	9'13'	29	0.5	9'13'	19	0.6
CH_3	25	0.8	CH_3	21	0.8			

1.4 Studying the binding mode of representative Hsp9 CTD inhibitors by molecular modeling

Compounds **89**, **96** and **104** were docked to the allosteric Hsp90 CTD binding site using the Hsp90β dimer conformation from our previous study.[1] The 3,4-dichlorophenyl moiety of these Hsp90 inhibitors was predicted to form several hydrophobic interactions with Ala608A, Ile605B, and Ala608B side chains. A strong ionic interaction was formed between the piperidine ring D and Glu489A side chain (Figure S13), while additional hydrophobic interactions were observed between Ala600A and the phenyl ring C of these inhibitors. For comparison, inhibitor **96** was also docked to the CTD of Hsp90α dimer and was predicted to form similar interactions (Figure S13D).

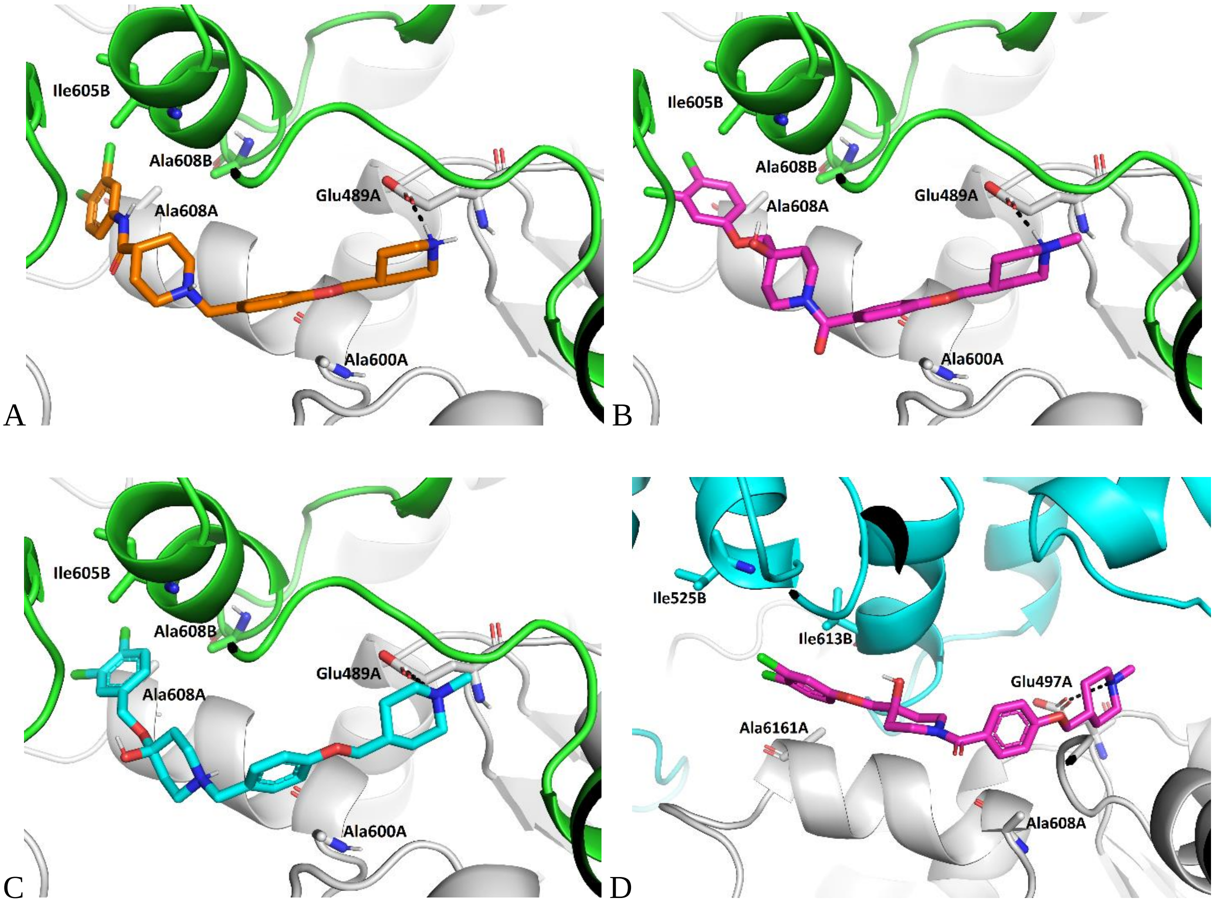

Figure S12. Docking binding mode of **A)** compound **89** (in orange stick), **B) 96** (in magenta sticks), **C) 104** (in cyan sticks) in the Hsp90β (PDB entry: 5FWK; protomers A and B are colored grey and green, respectively) and **D) 96** (in magenta sticks) in the Hsp90α (protomers A and B are colored grey and cyan, respectively) C-terminal-domain binding site. For clarity, only the amino acids that interact with inhibitors are shown. Hydrogen bonds are shown as black dashed lines.

Hsp90β-**96** docking complex was subjected to a 500 ns MD simulation to check the stability of the complex and evaluate interactions with the binding site in the MD trajectory. The interaction features between **96** and the allosteric Hsp90β CTD binding site during the MD simulation were analyzed using the MD analysis tool in LigandScout 4.4 Expert. Figure S14A shows the plot of the most frequently appearing unique structure-based pharmacophore models (SBPM), in terms of the total number of interaction features for each SBPM versus the frequency (#Appearances). The most frequent model (seen 603 times) showed six interaction features, including a positive ionizable feature associated with the tertiary amine and Glu489A, hydrophobic interactions with Ala600A, Ala608A, Ile517B, Ile605B, and Ala608B, and a hydrogen bond with Lys607A (Figure S14B). Figure S14C shows the most frequently occurring interaction partners of **96** in the Hsp90 CTD binding site. 3,4-Dichlorophenyl moiety constantly formed several hydrophobic interactions, tertiary amine formed ionic interaction with Glu489A more than 95% of the simulation time, while a hydrogen bond with Lys607A side chain is present for 63% of the simulation time.

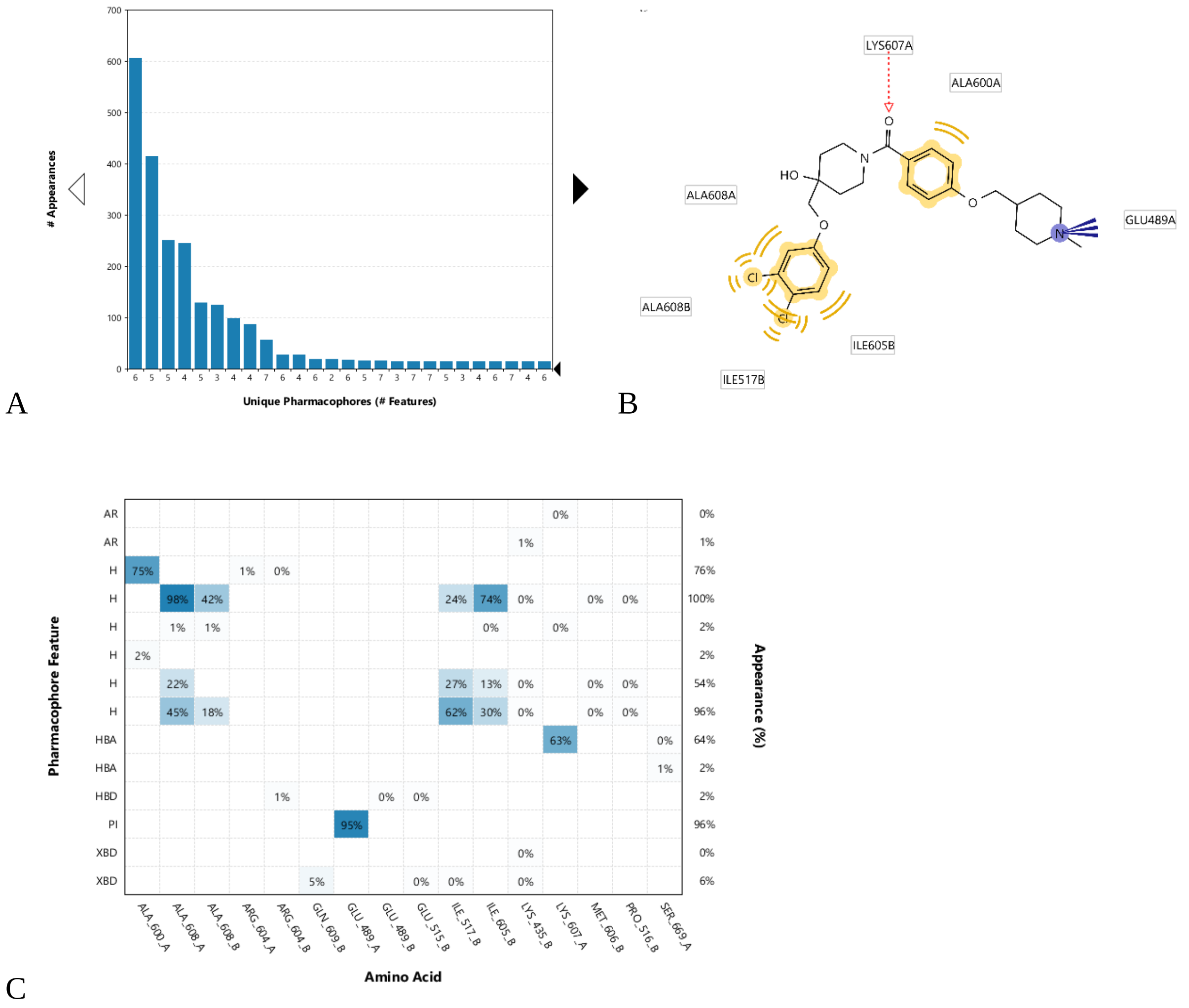

Figure S13. A) Plot of the most frequent unique structure-based pharmacophore models derived from the molecular dynamics simulations of the Hsp90β C-terminal domain in complex with **96**. The numbers below the bars indicate the numbers of interaction features observed during molecular dynamics simulation for the pharmacophore models; **B)** The most frequently occurring interactions of compound **96** in the Hsp90 CTD binding site during the 500 ns molecular dynamics simulation. The pharmacophore features are as follows: hydrophobics (in yellow), hydrogen bond acceptor (red arrow), and positive ionizable (in blue); **C)** Interaction map showing the most frequently occurring interactions of compound **96** in the Hsp90 C-terminal-domain binding site during the 500 ns molecular dynamics simulation.

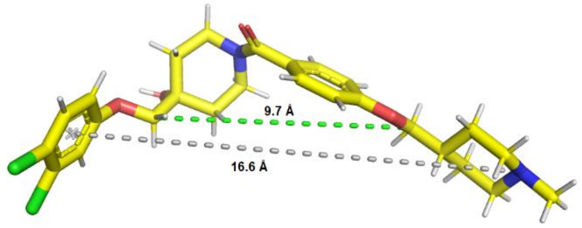

Figure S14. Distances between the described structural elements critical for potent antiproliferative activity.

1.5 Molecular Docking

For the docking with the HYBRID software (OEDOCKING 3.3.0.2: OpenEye Scientific Software, Santa Fe, NM, USA. http://www.eyesopen.com), the predicted Hsp90α and Hsp90β CTD binding site[1] (PDB entry: 5FWK[2]) was prepared using MAKE RECEPTOR (Release 3.2.0.2, OpenEye Scientific Software, Inc., Santa Fe, NM, USA; www.eyesopen.com). The homology model of Hsp90α in the same conformation was built based on the structure of Hsp90β. The grid box around the Hsp90 CTD inhibitor[1] was generated automatically and was not adjusted. This resulted in a box with the following dimensions: 21.7 Å × 24.7 Å × 16.0 Å and the volume of 8550 Å^3. For "Cavity detection", the slow and effective "Molecular" method was used for detection of binding sites. Inner and outer contours of the grid box were also calculated automatically using "Balanced" settings for "Site Shape Potential" calculation. The inner contours were disabled. The ligands were prepared by OMEGA (Release 3.3.1.2, OpenEye Scientific Software, Inc., Santa Fe, NM, USA; www.eyesopen.com).[3] The ligands were then docked to the prepared Hsp90α or Hsp90β CTD binding site using HYBRID (default settings).[4] The results were visualized and analyzed with VIDA (version 4.3.0.4, OpenEye Scientific Software, Inc., Santa Fe, NM, USA, www.eyesopen.com).

1.6 Molecular dynamics simulations

The MD package NAMD (version 3.0)[5] and CHARMM36m[6] force field were used for MD simulations using the cryo-electron microscopy structure of full-length human Hsp90β (PDB code: 5FWK). Molecular mechanics parameters for compound **96** were estimated using the ParamChem tool.[7–9] Steepest descent (10000 steps) and adopted basis Newton−Raphson (10000 steps) energy minimizations were first performed to remove atomic clashes and optimize the atomic coordinates of the Hsp90β−**96** docking complex. The structure of the energy-minimized complex for MD simulation was prepared using psfgen in VMD (version 1.9.4.).[10] The complex was embedded in a box of TIP3P water molecules. The system was neutralized by addition of NaCl. The MD simulation was carried out in the NPT ensemble using the periodic boundary conditions. Langevin dynamics and Langevin piston methods were used for temperature (300 K) and pressure (1 atm) control, respectively. Short-range and long-range forces were calculated every 1 and 2 time steps, respectively, with a time step of 2.0 ps. The smooth particle mesh Ewald method was used to calculate the electrostatic interactions.[11] The short-range interactions were cut off at 12 Å. All of the chemical bonds between hydrogen and the heavy atoms were held fixed using the SHAKE algorithm.[12] The simulation consisted of three consecutive steps: (i) solvent equilibration for 1 ns with ligand and protein constrained harmonically around the initial structure; (ii) equilibration of the complete system for 1 ns with ligand and protein released; and (iii) an unconstrained 500 ns production run. For structure-based pharmacophore modeling, 2500 frames from the production run were saved separately and used for interaction analysis.

1.7 Structure-based pharmacophore modeling

The MD trajectory of Hsp90β dimer (PDB Entry: 5FWK) in complex with compound **96** was used for chemical feature interaction analysis using LigandScout 4.4 Expert.[13] The first frame of the trajectory (in PDB format) and the MD trajectory files (DCD format) are needed as input for the creation of an ensemble of structure-based pharmacophore models. LigandScout 4.4 Expert was used to generate 2500 structure-based pharmacophore models from the 500 ns MD simulation.

1.8 Representative ^{1}H and ^{13}C NMR spectra

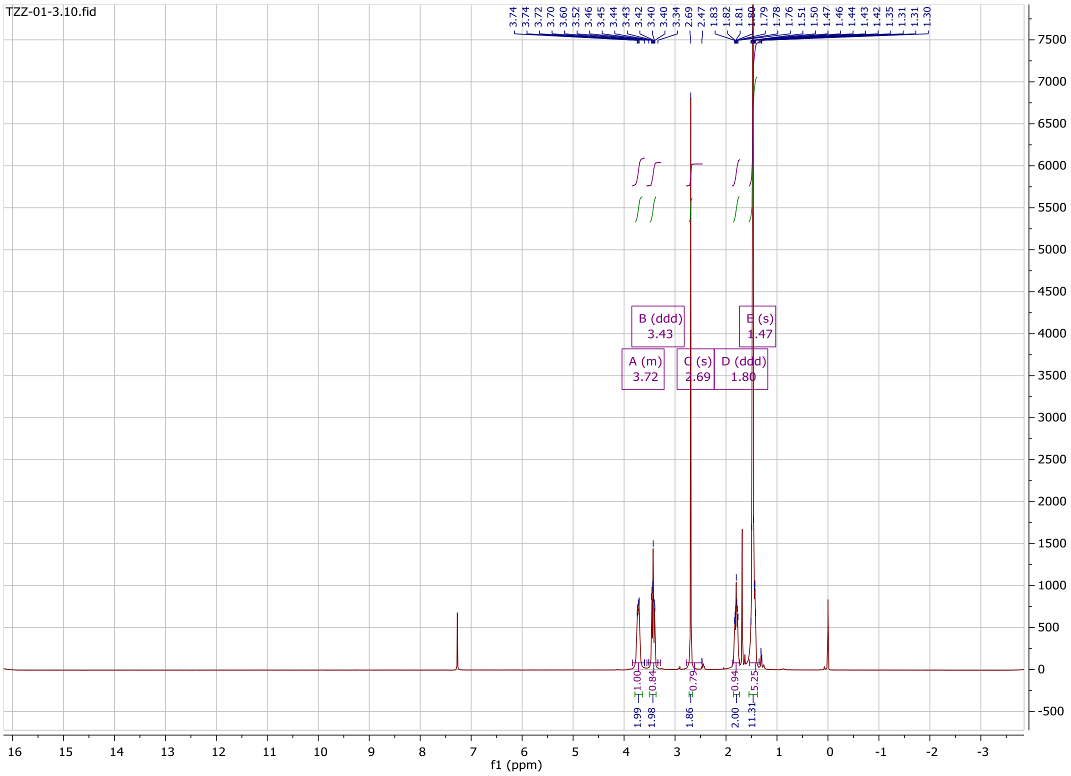

Figure S15. ^{1}H spectrum of compound **1**

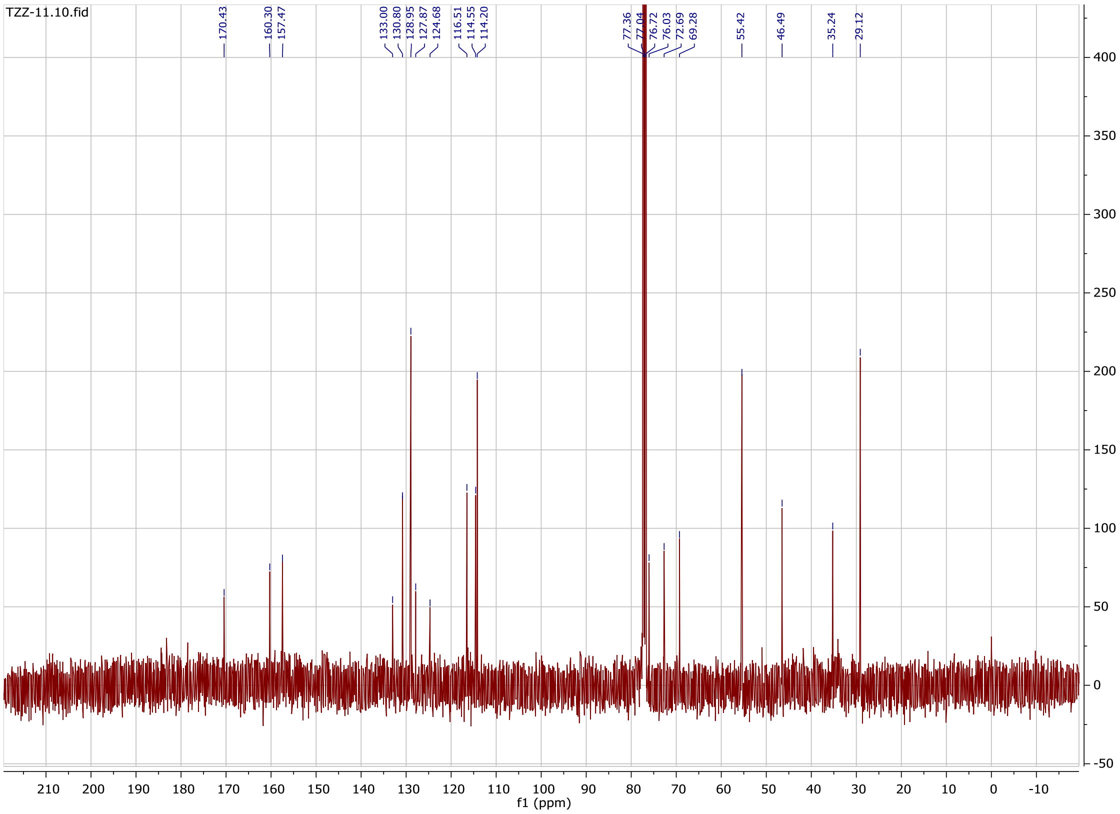

Figure S16. ^{13}C spectrum of compound **1**

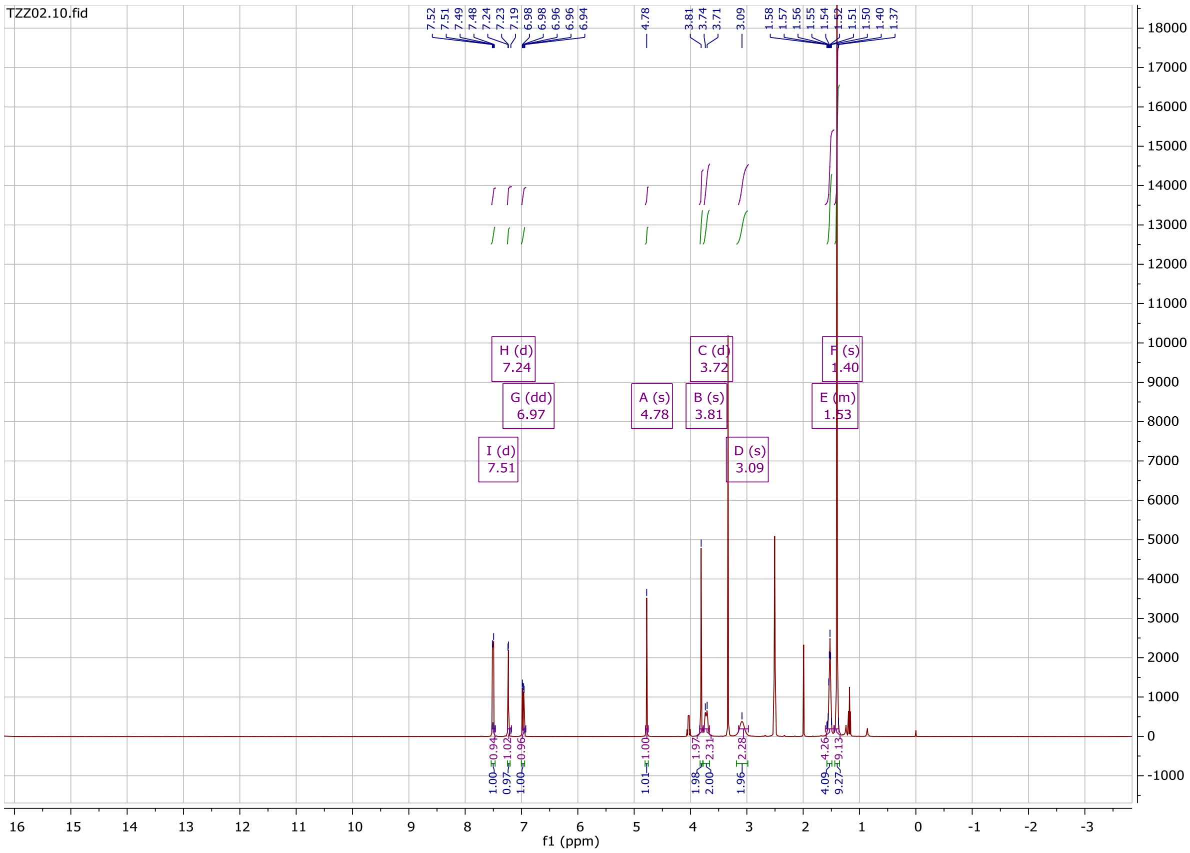

Figure S17. ^{1}H spectrum of compound **3**

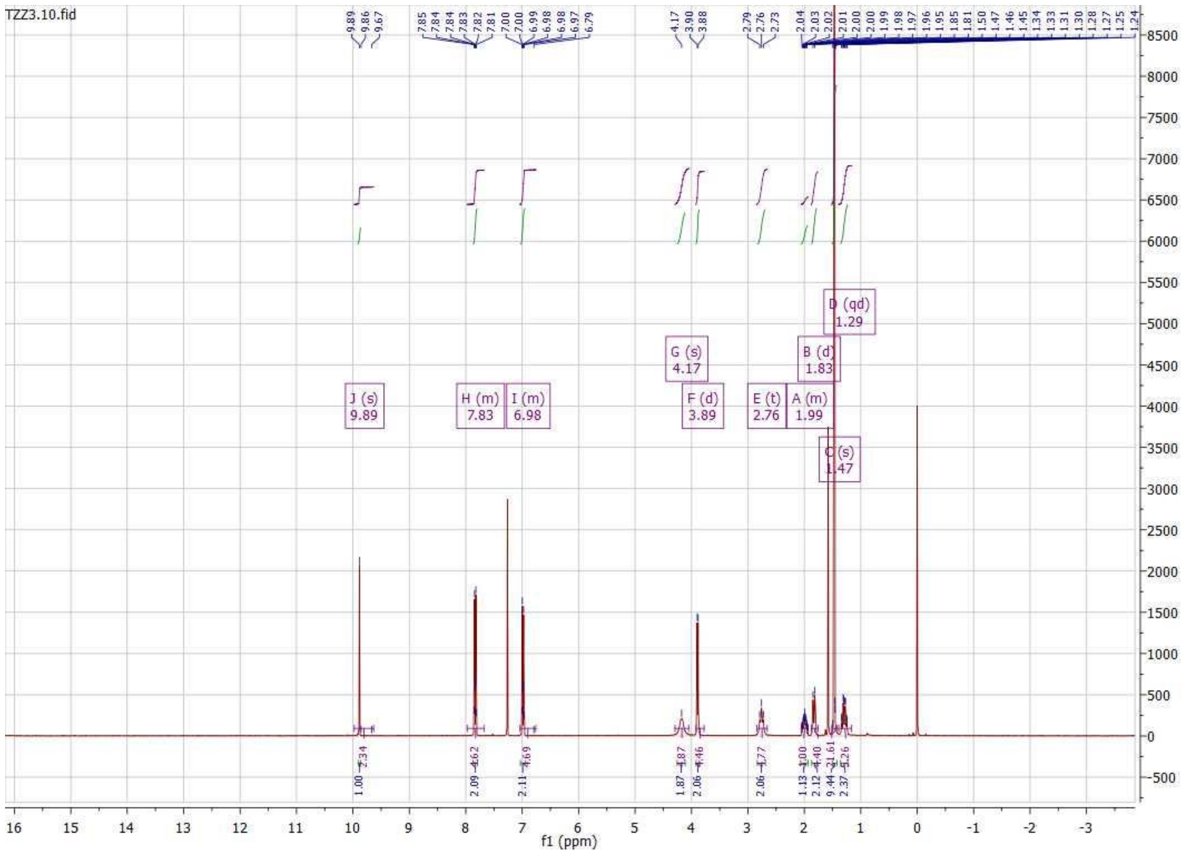

Figure S18. ^{1}H spectrum of compound **24**

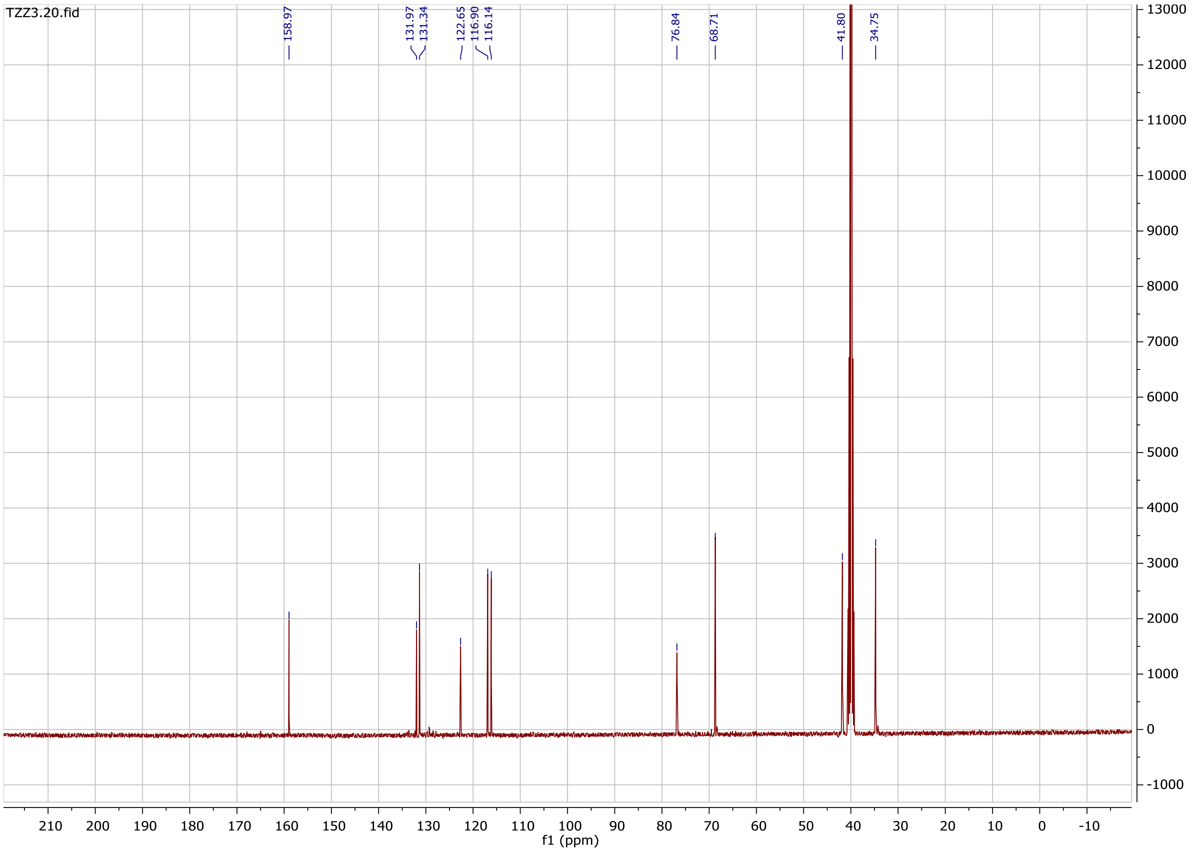

Figure S19. ^{13}C spectrum of compound **24**

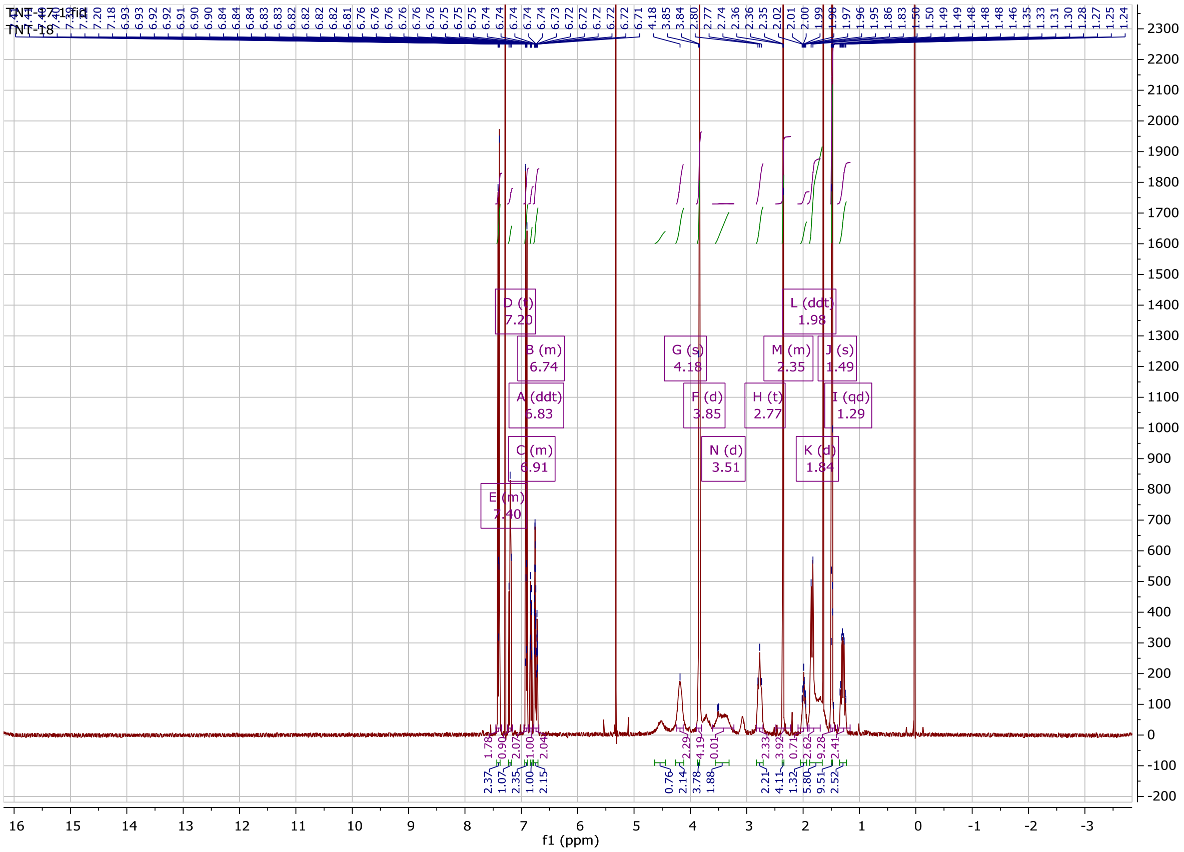

Figure S20. ^{1}H spectrum of compound **45**

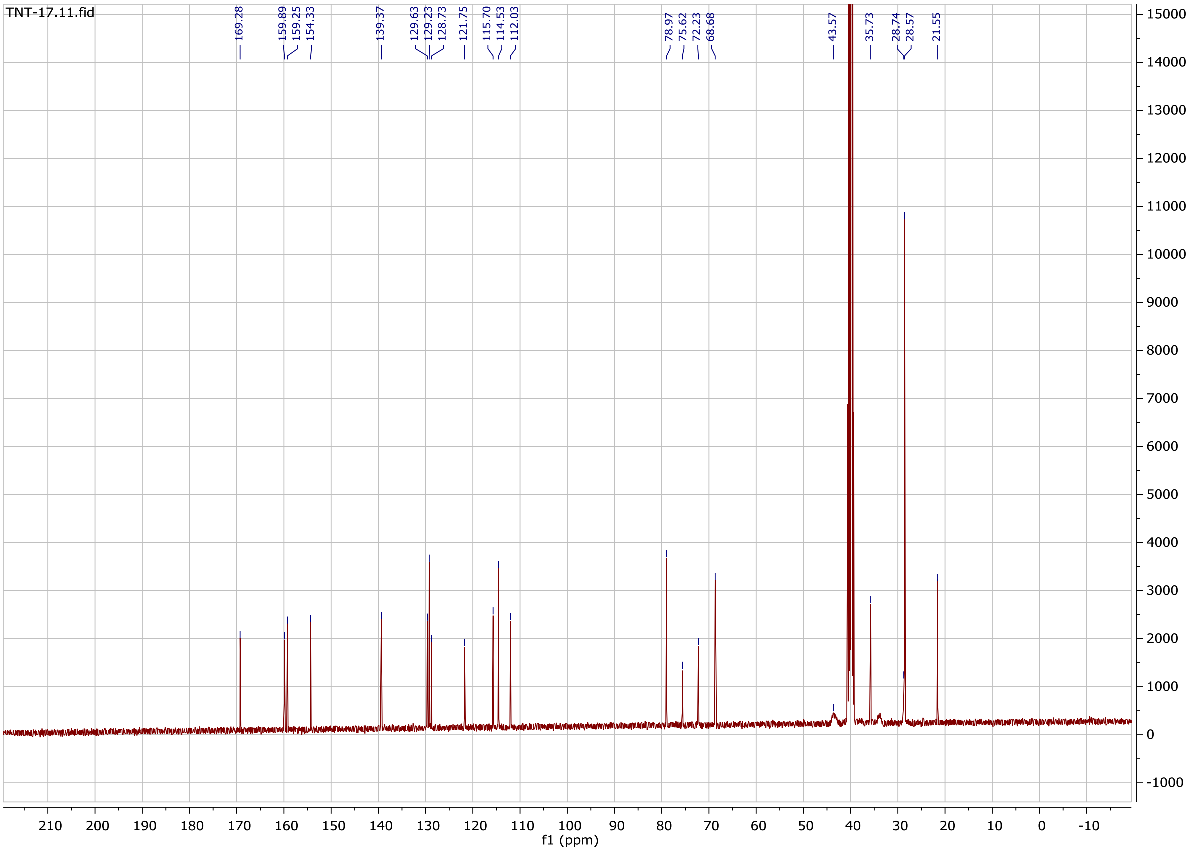

Figure S21. ^{13}C spectrum of compound **45**

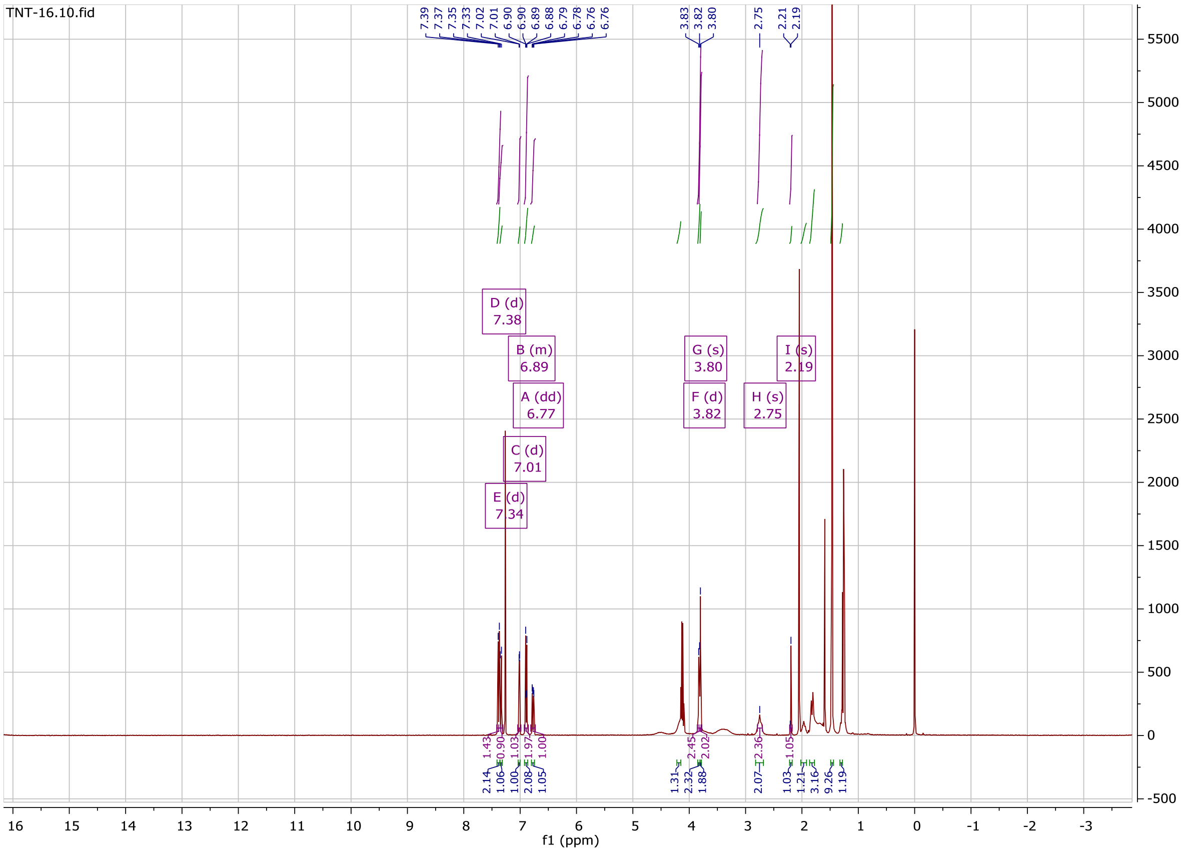

Figure S22. ¹H spectrum of compound **46**

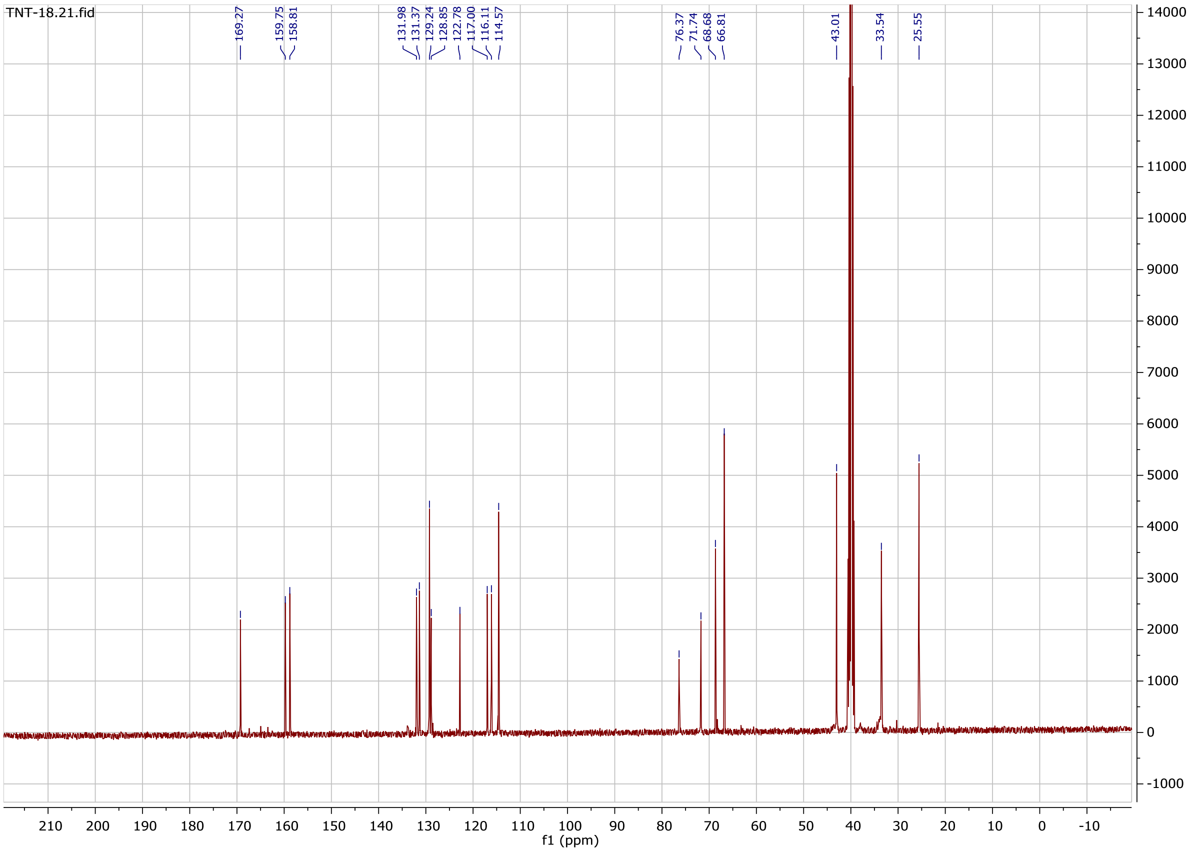

Figure S23. ^{13}C spectrum of compound **46**

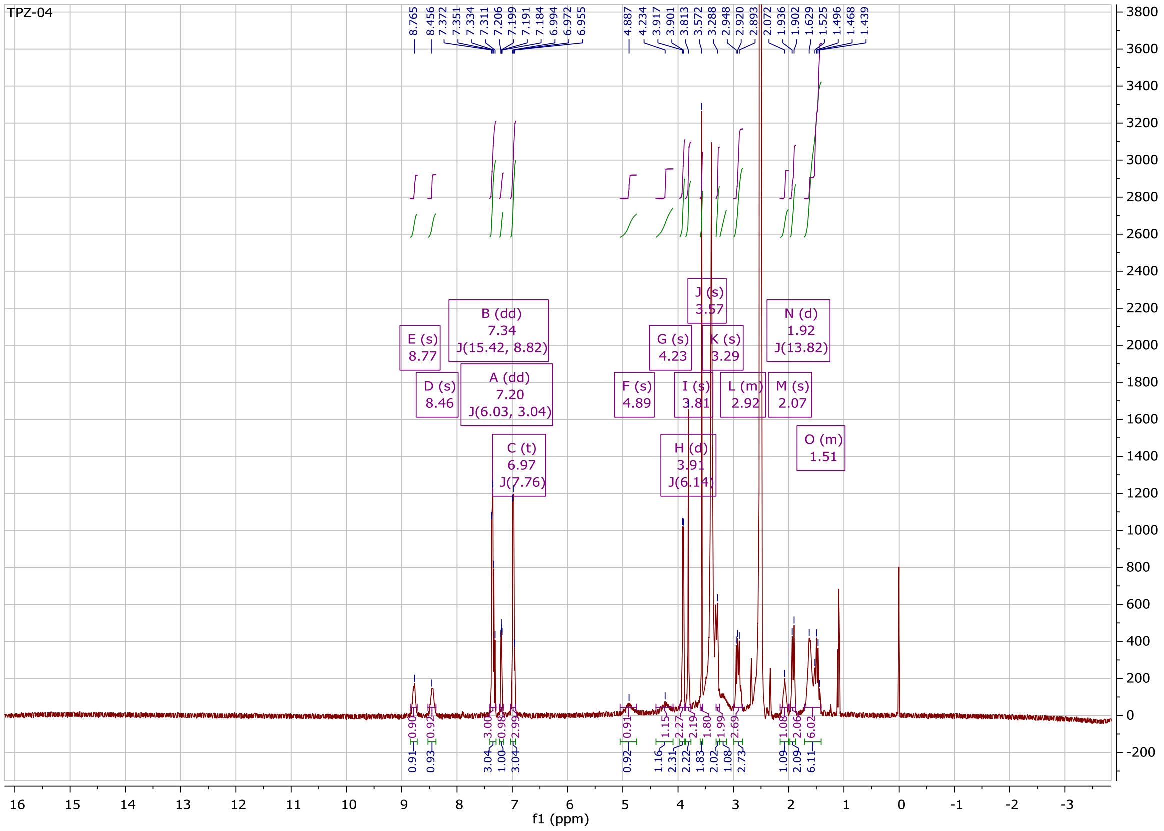

Figure S24. ^{1}H spectrum of compound **47**

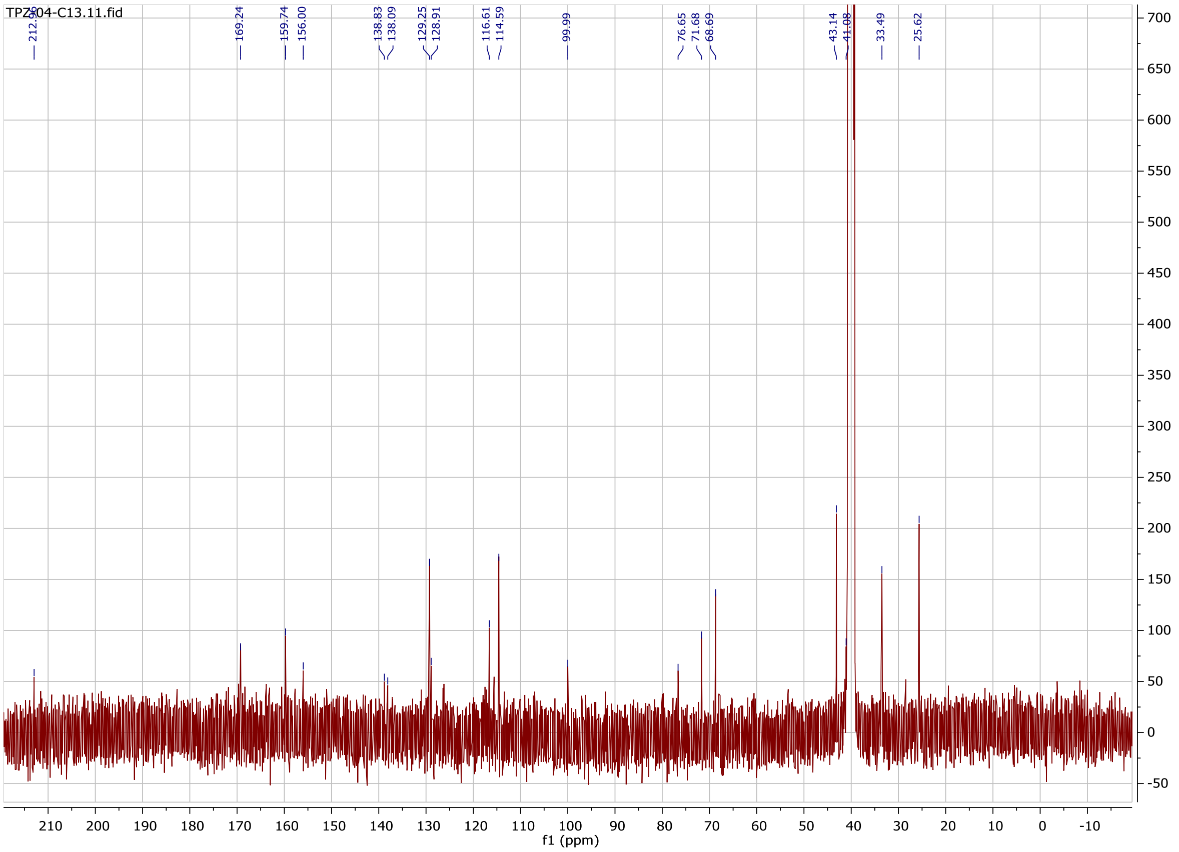

Figure S25. ^{13}C spectrum of compound **47**

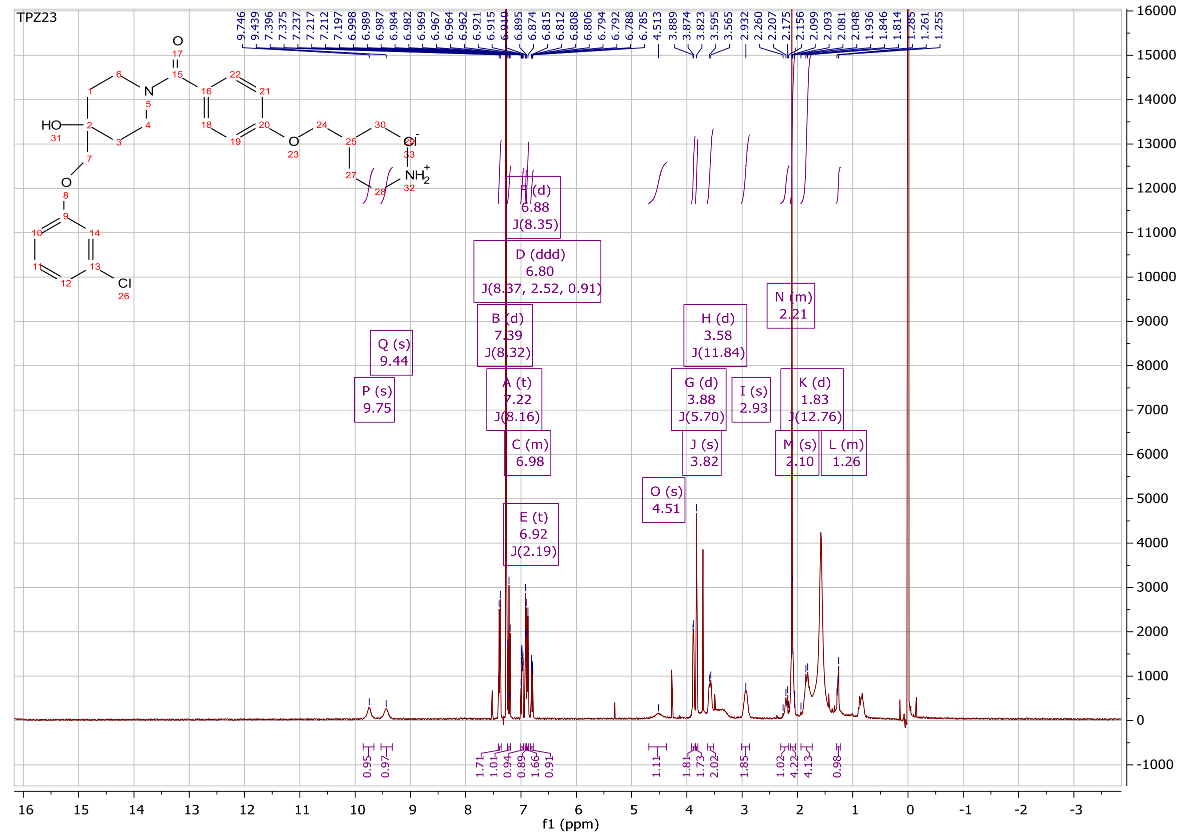

Figure S26. ^{1}H spectrum of compound **48**

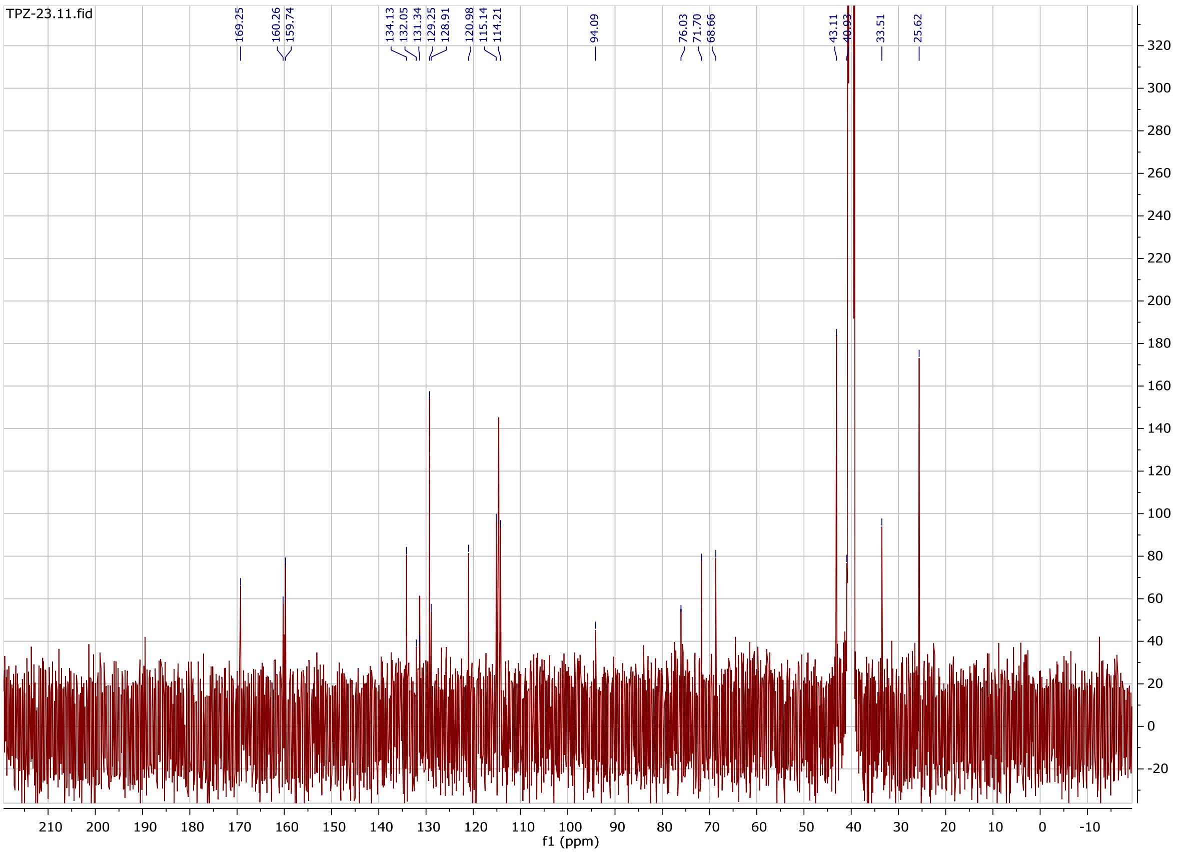

Figure S27. ^{13}C spectrum of compound **48**

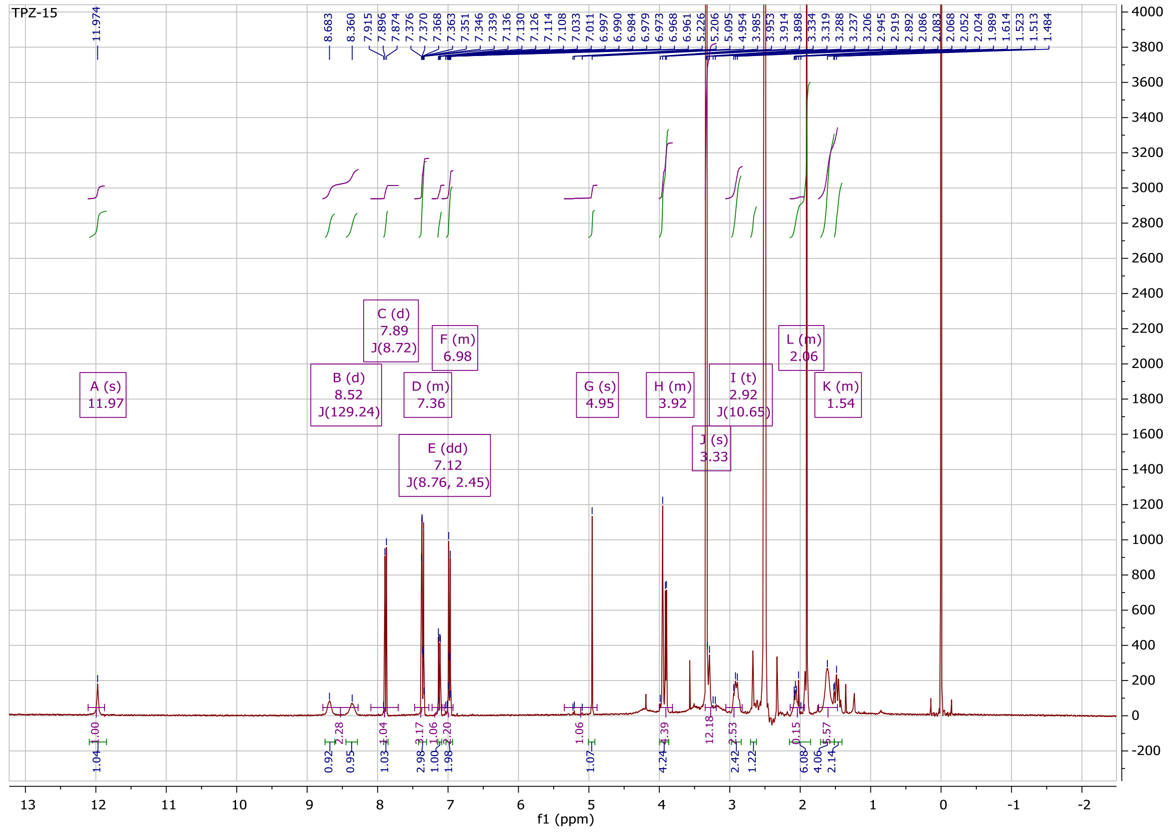

Figure S28. ^{1}H spectrum of compound **49**

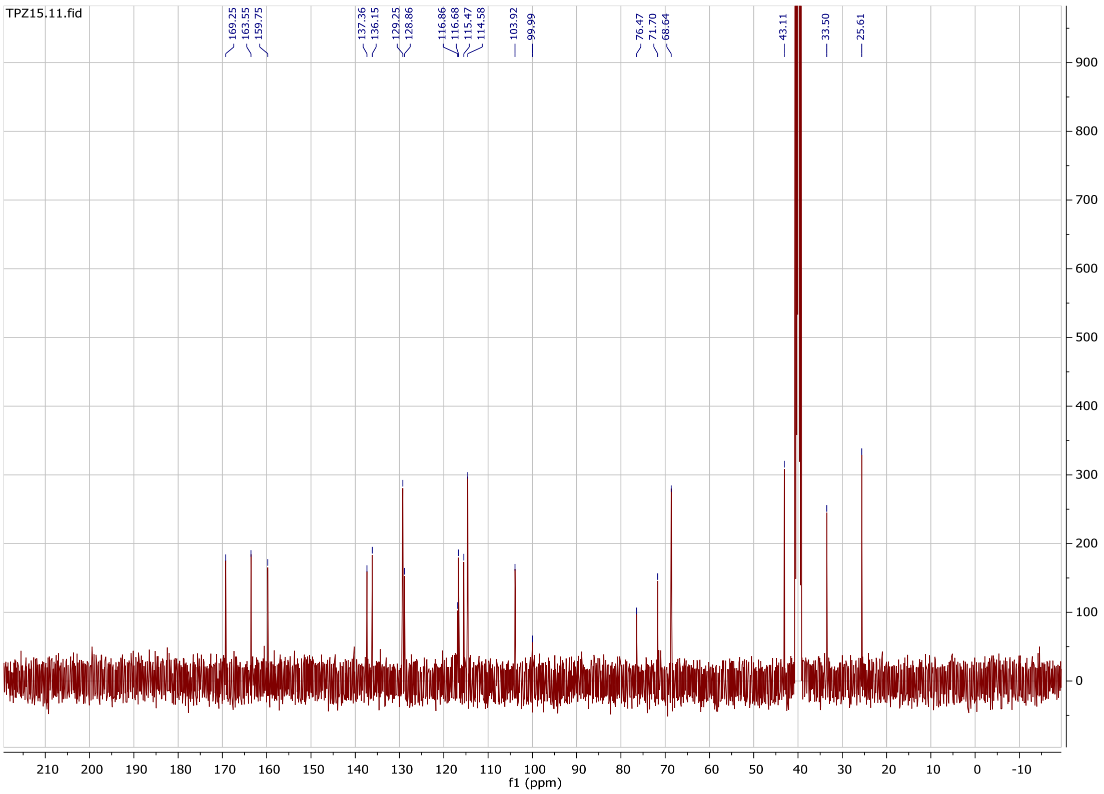

Figure S29. ^{13}C spectrum of compound **49**

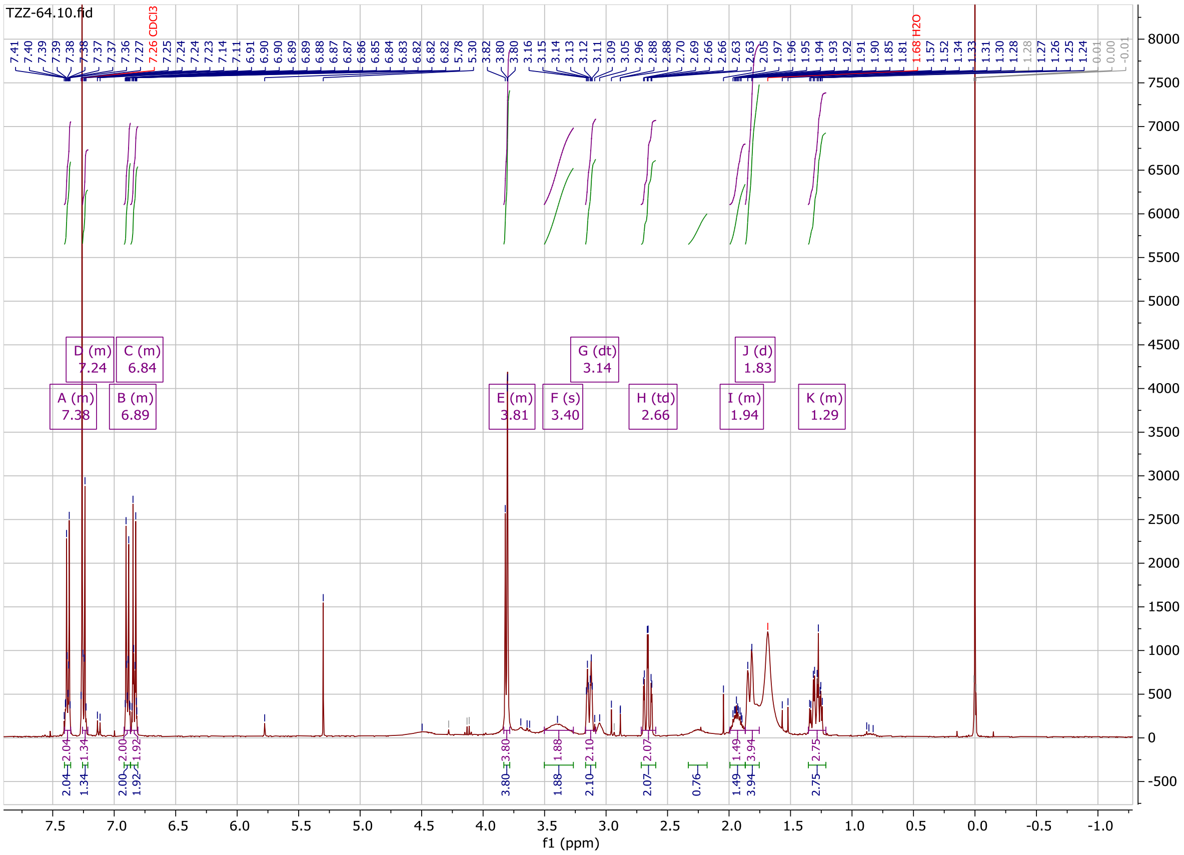

Figure S30. ^{1}H spectrum of compound **50**

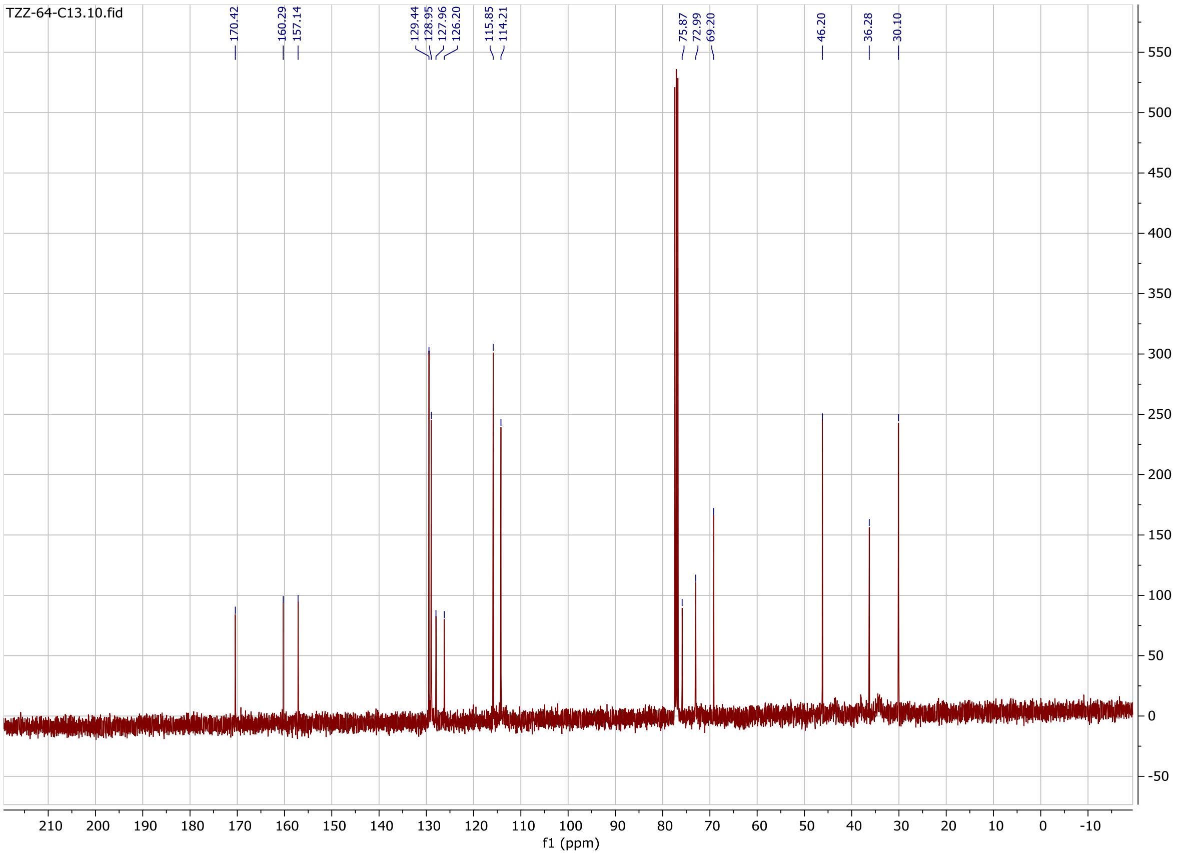

Figure S31. ^{13}C spectrum of compound **50**

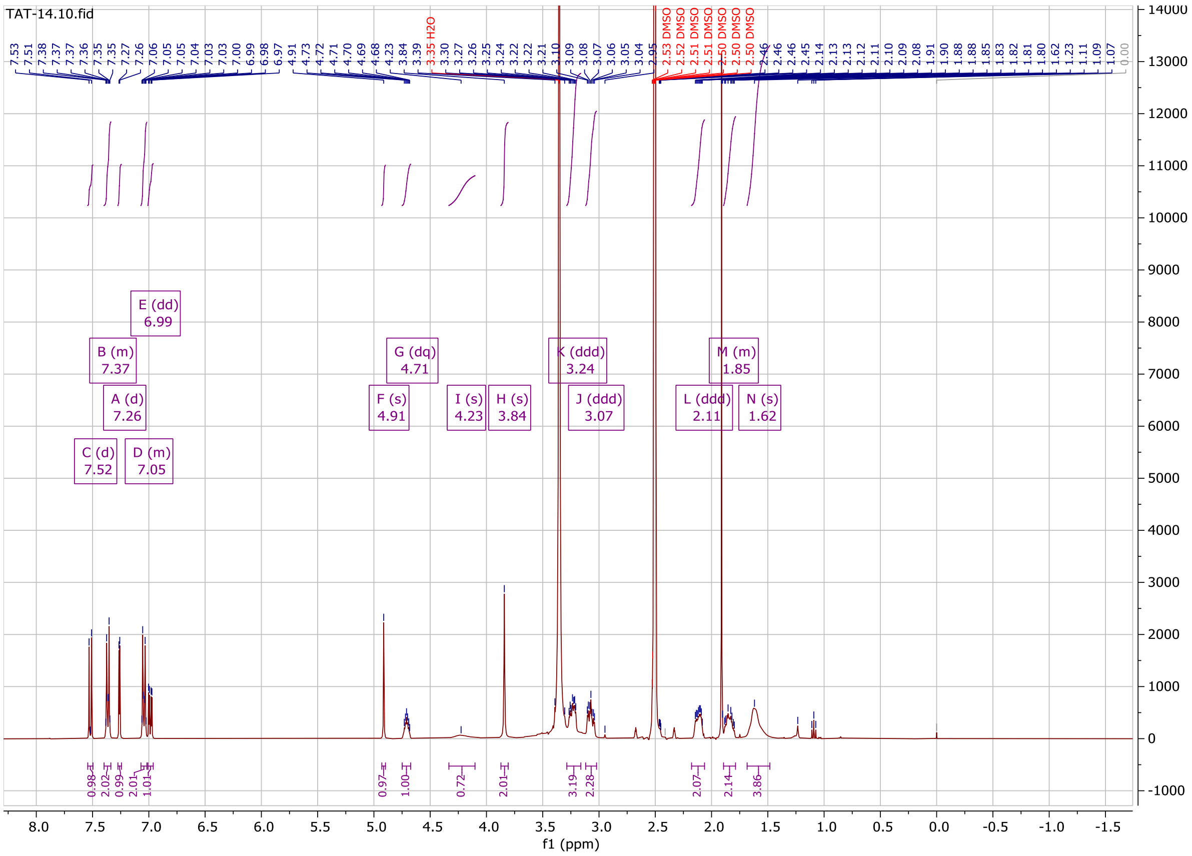

Figure S32. ^{1}H spectrum of compound **51**

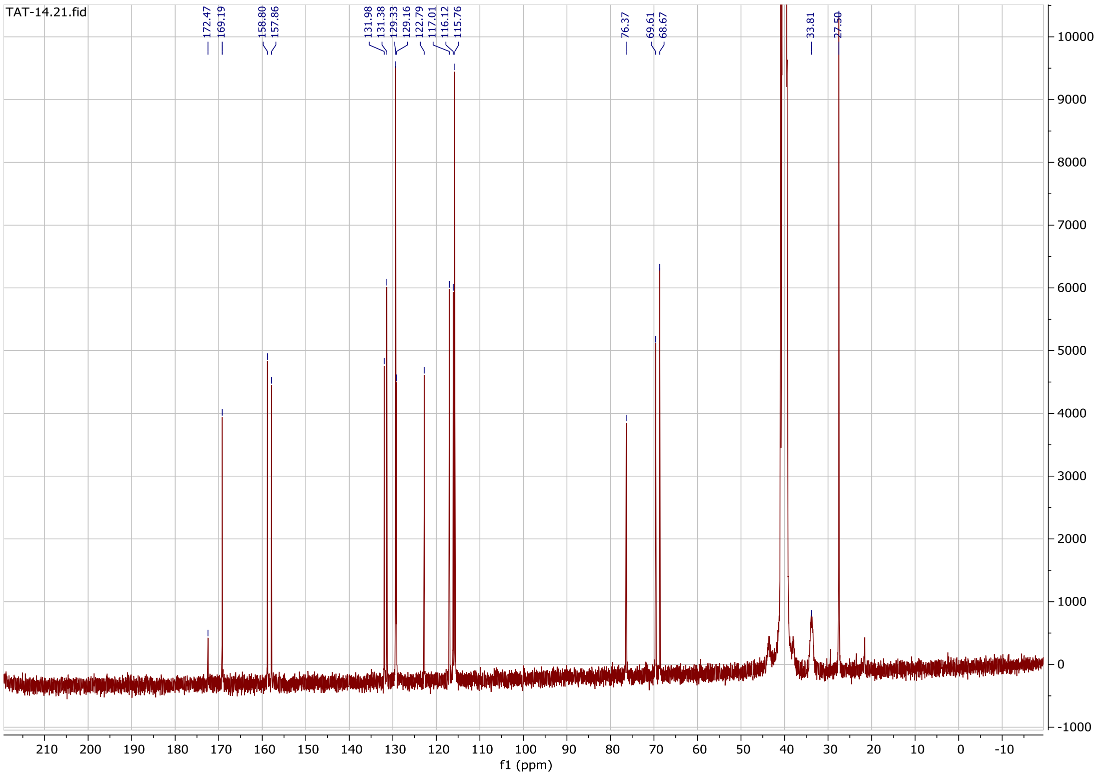

Figure S33. ^{13}C spectrum of compound **51**

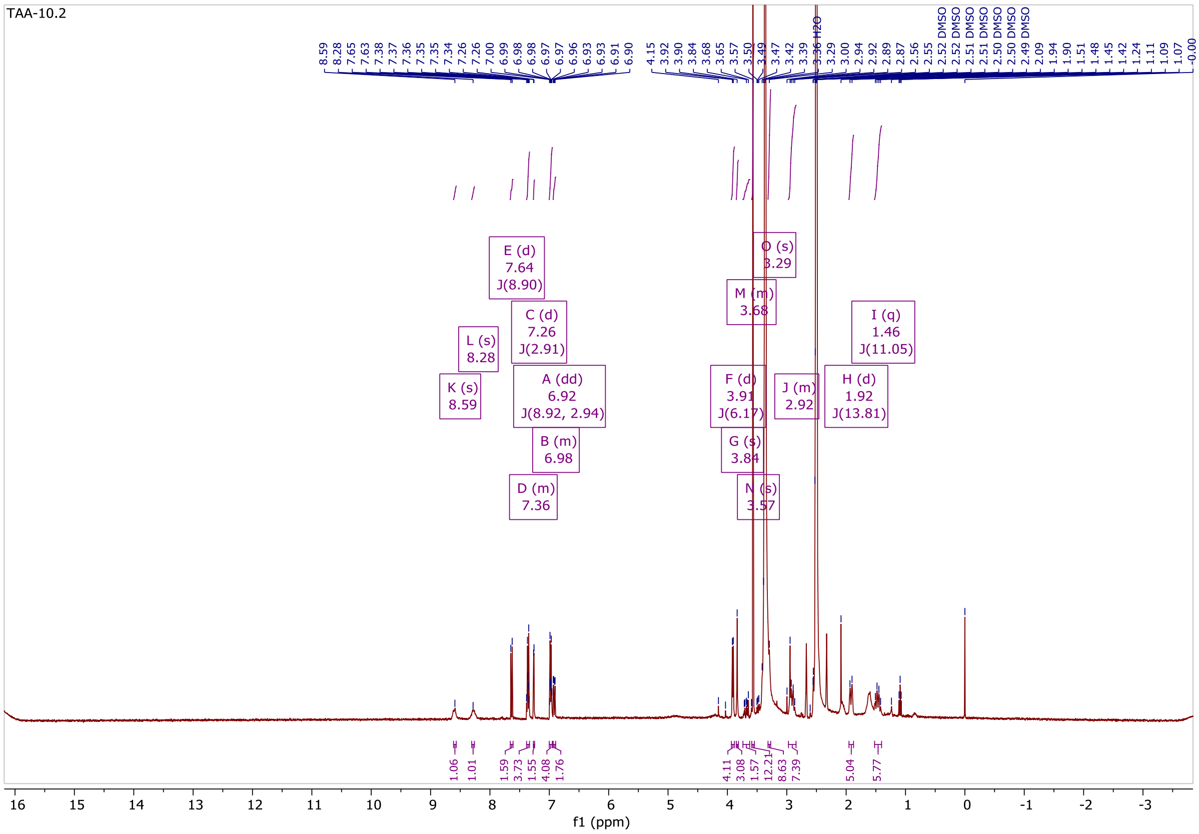

Figure S34. ^{1}H spectrum of compound **53**

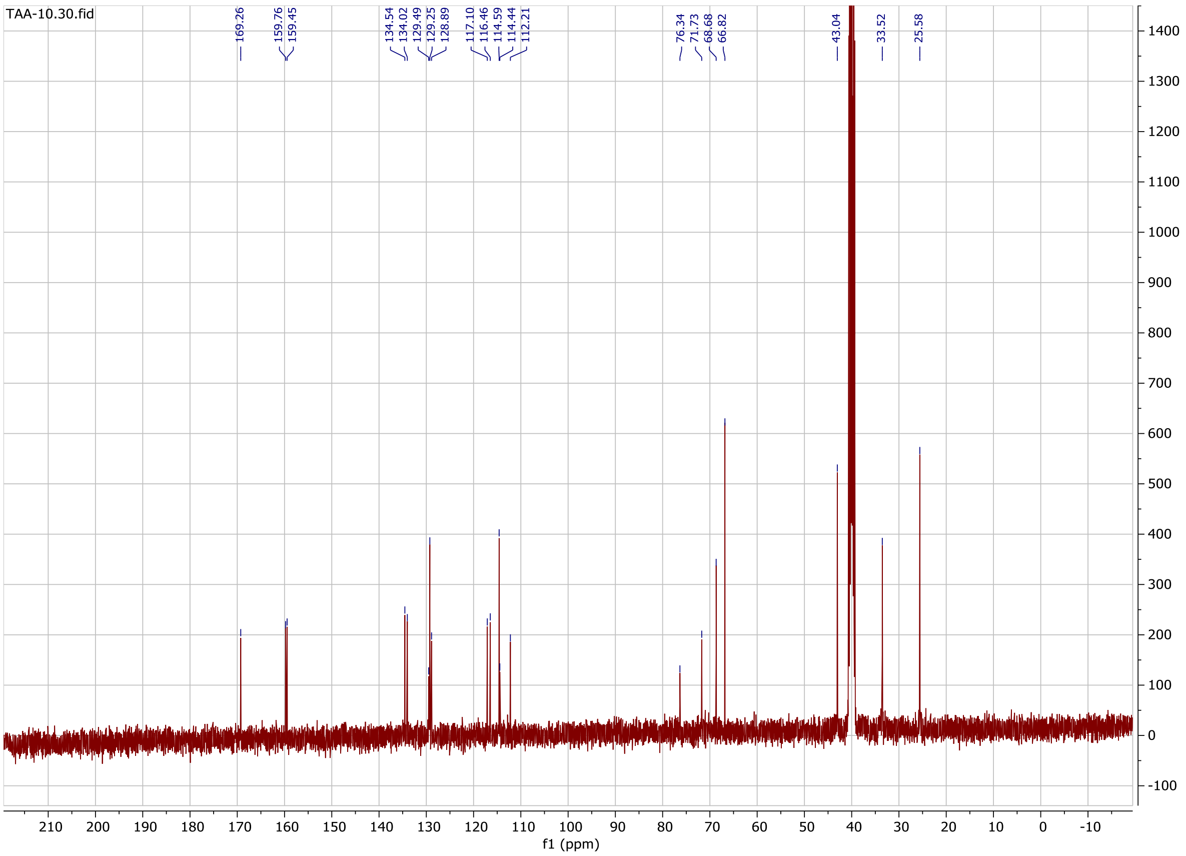

Figure S35. ^{13}C spectrum of compound **53**

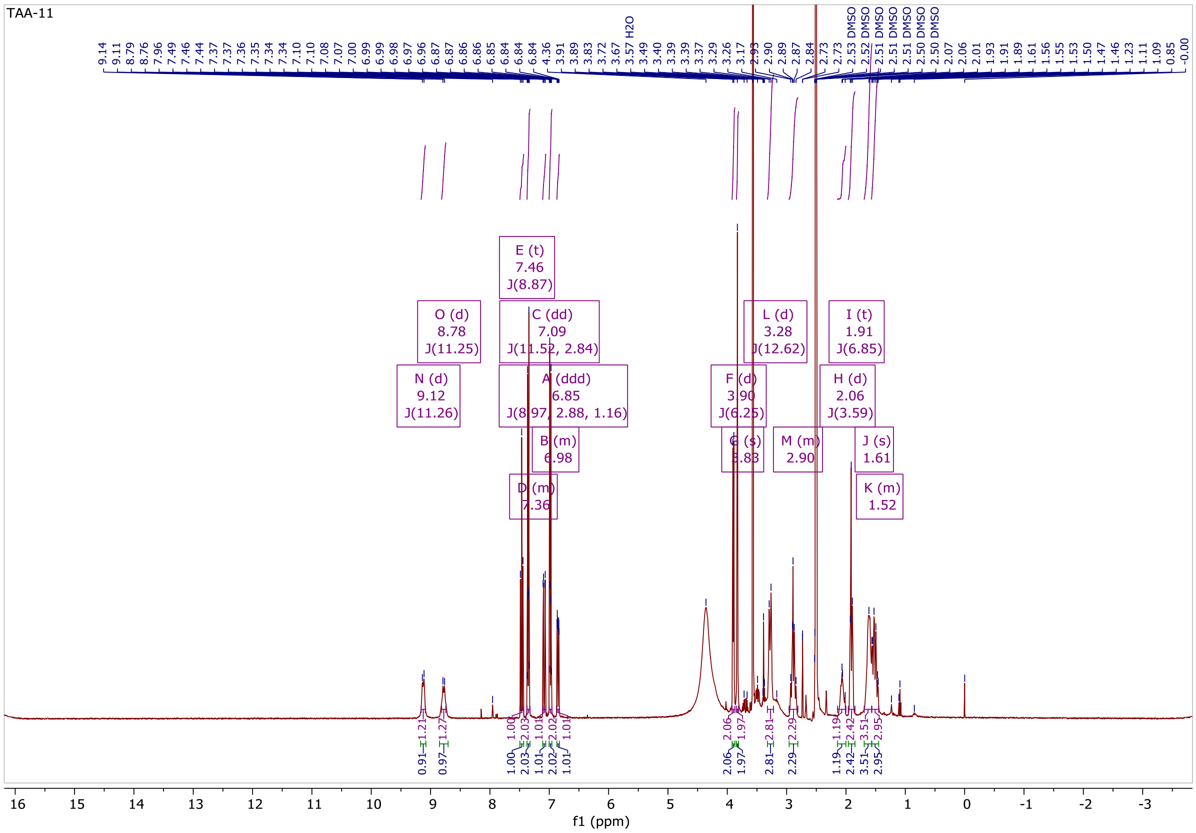

Figure S36. ^{1}H spectrum of compound **54**

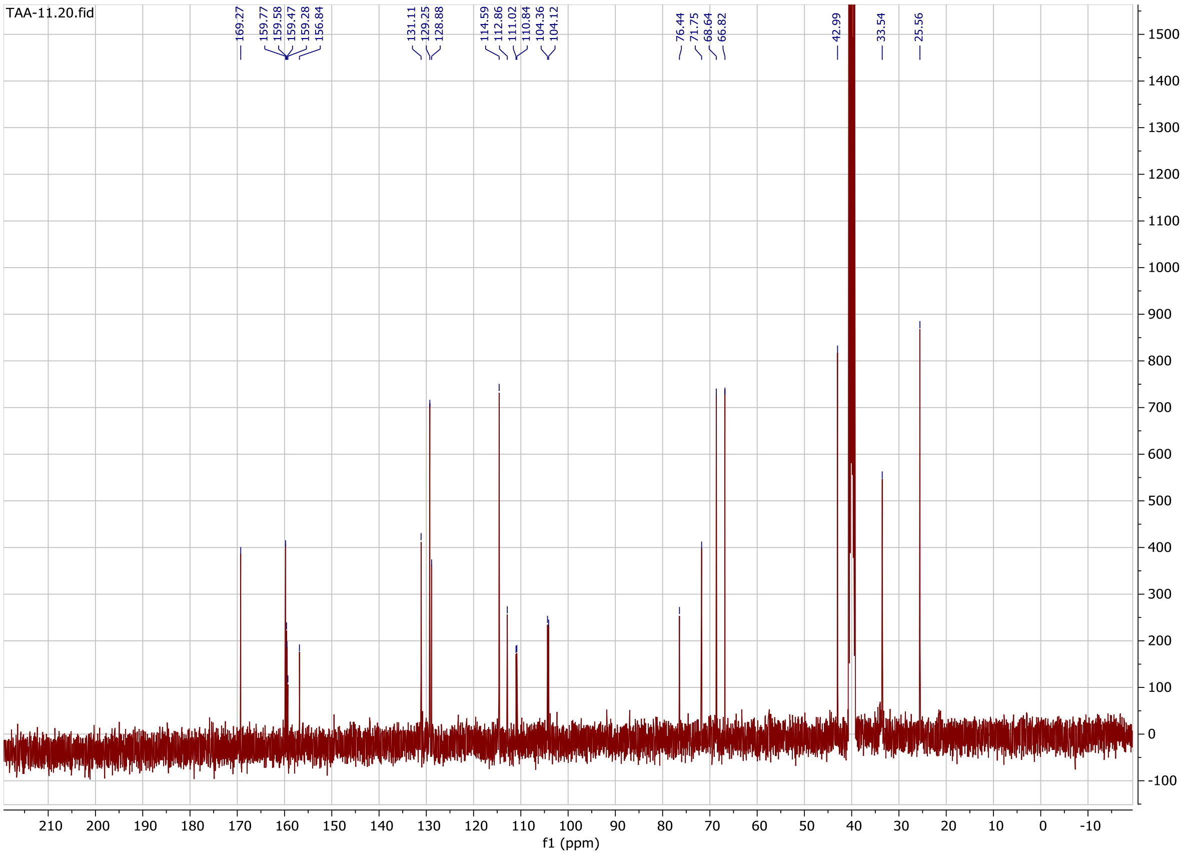

Figure S37. ^{13}C spectrum of compound **54**

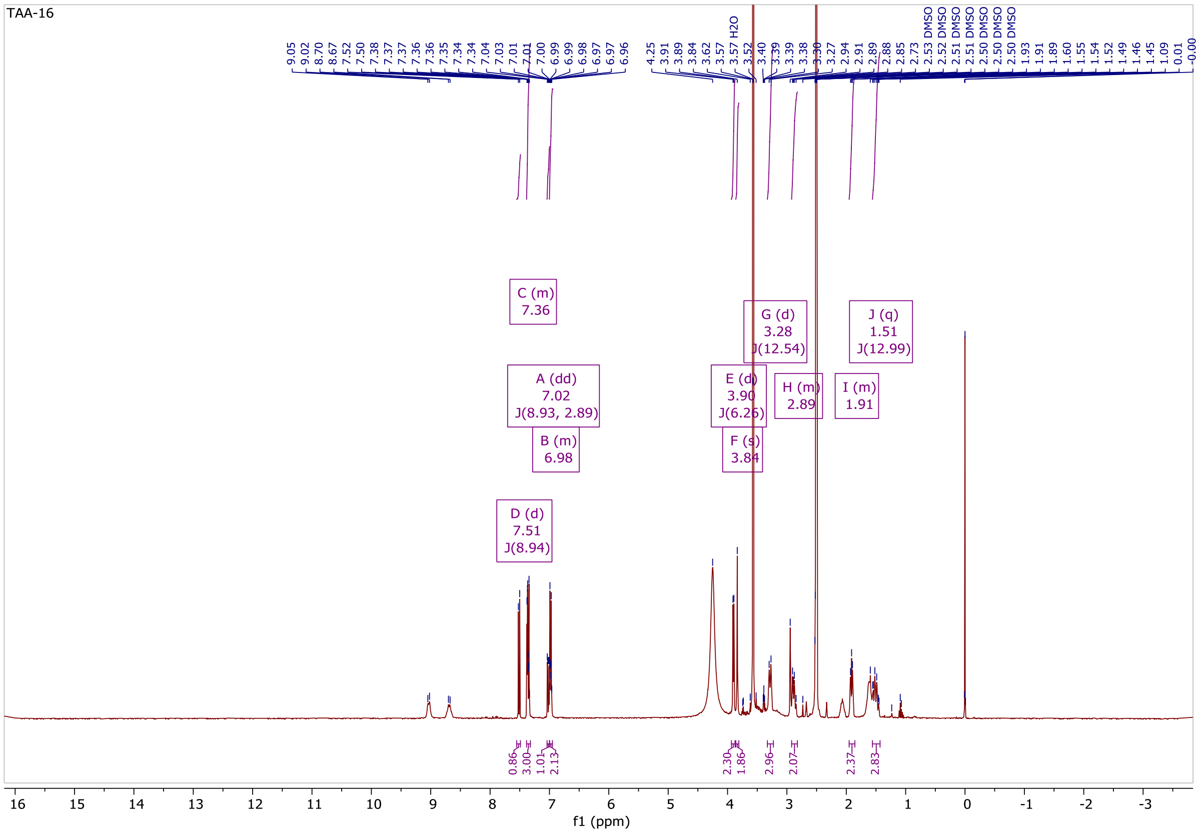

Figure S38. ^{1}H spectrum of compound **55**

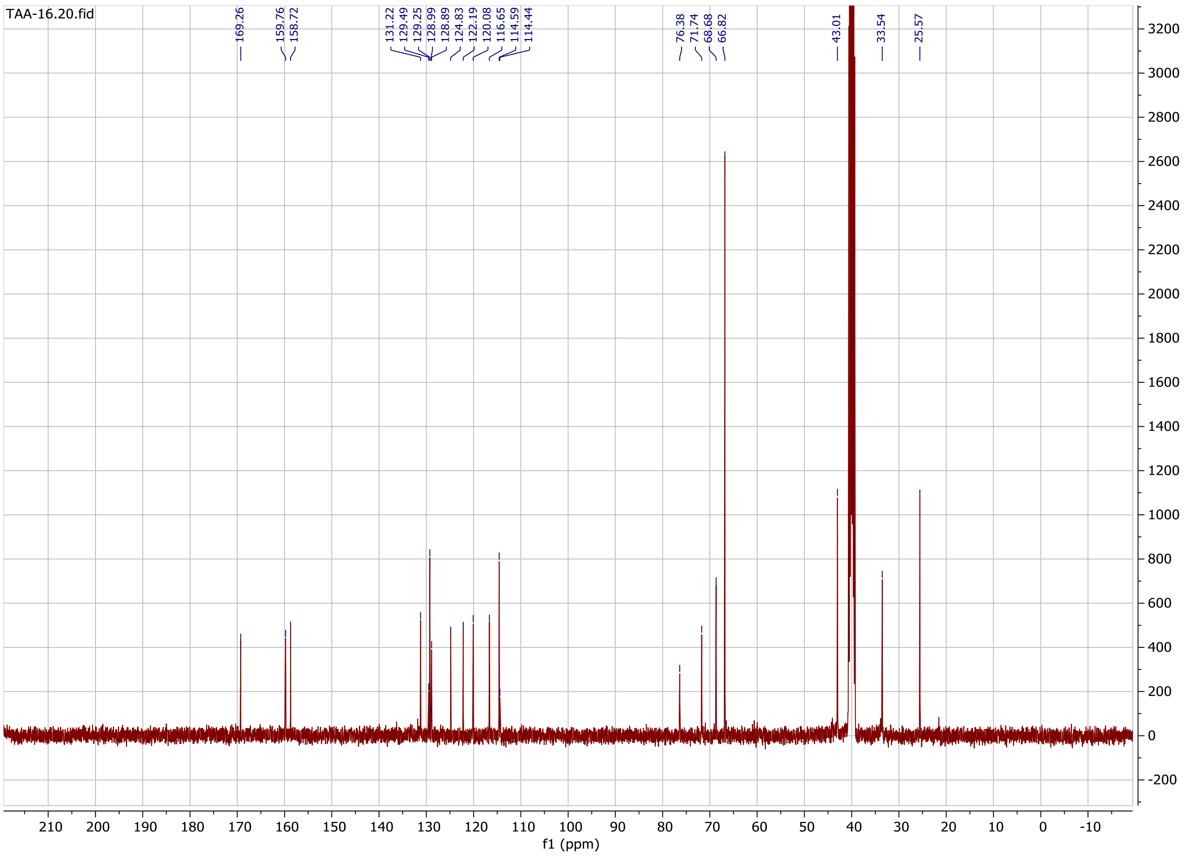

Figure S39. ^{13}C spectrum of compound **55**

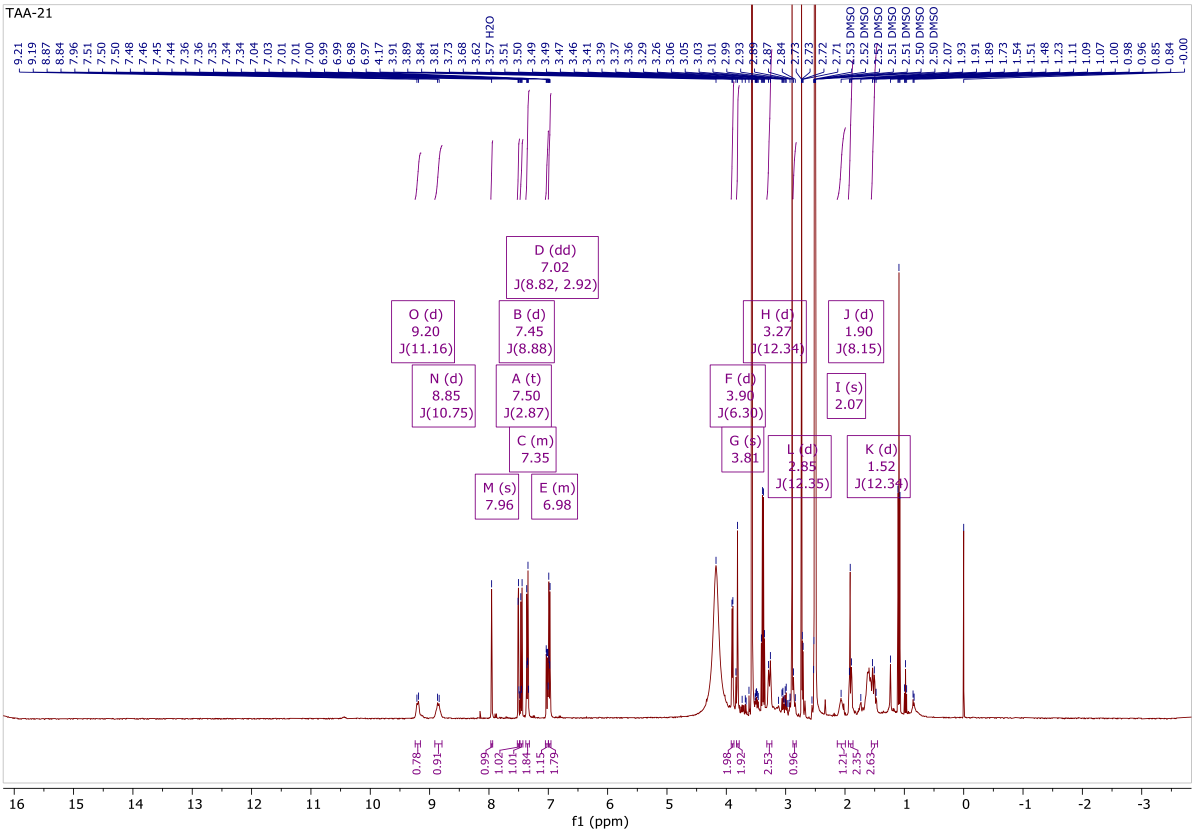

Figure S40. ^{1}H spectrum of compound **56**

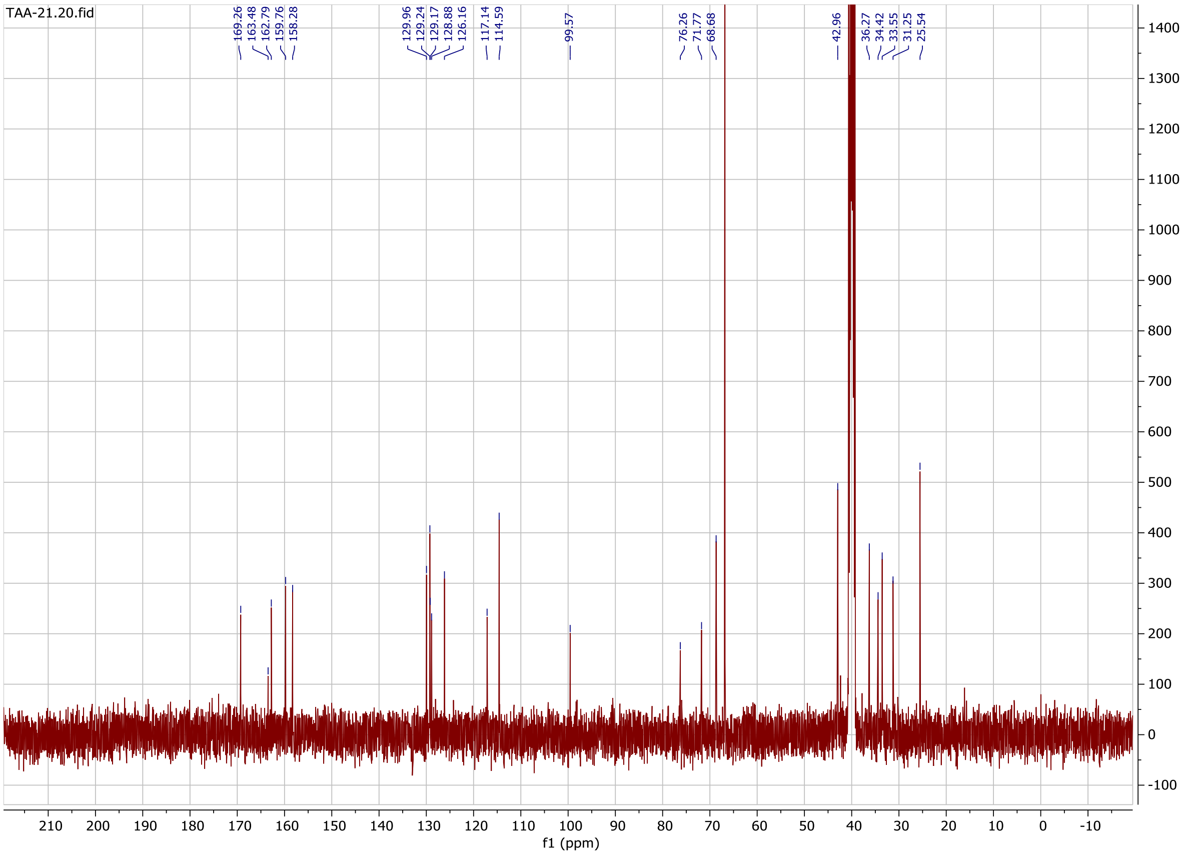

Figure S41. ^{13}C spectrum of compound **56**

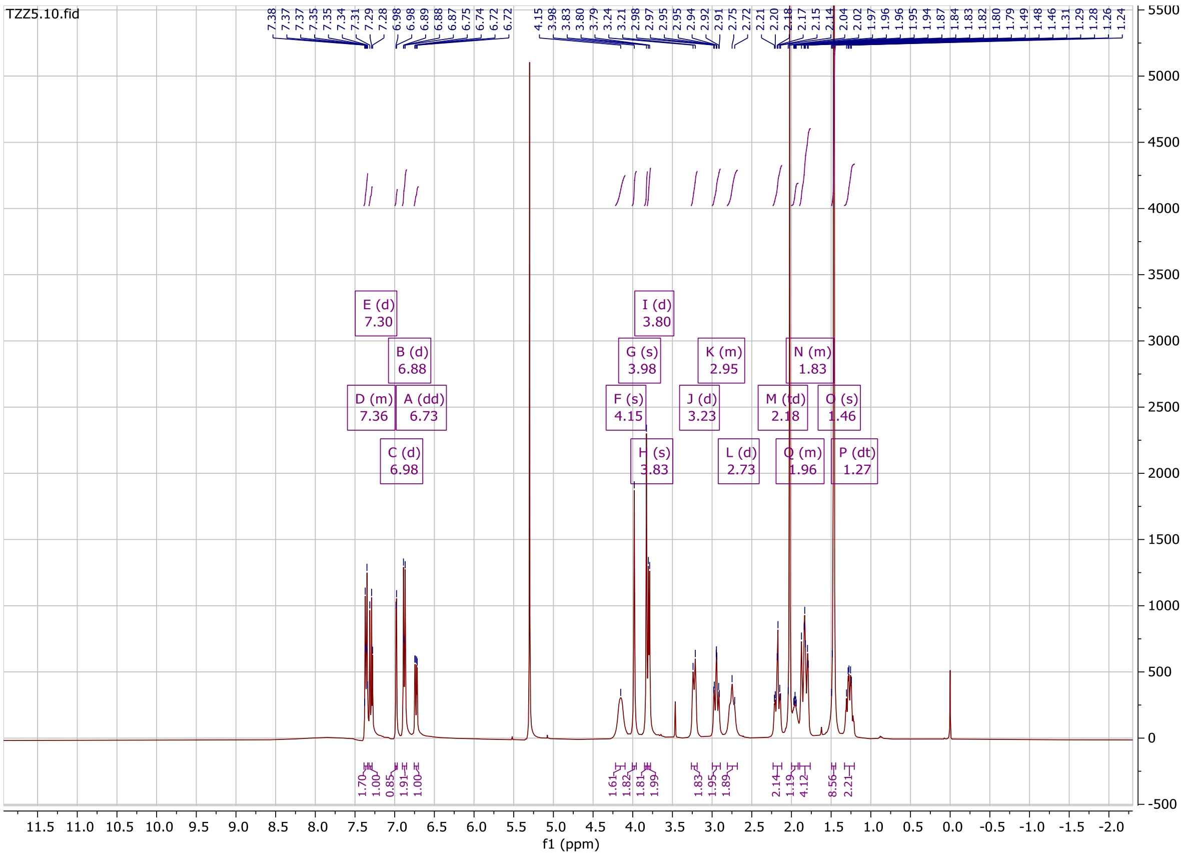

Figure S42. ^{1}H spectrum of compound **44**

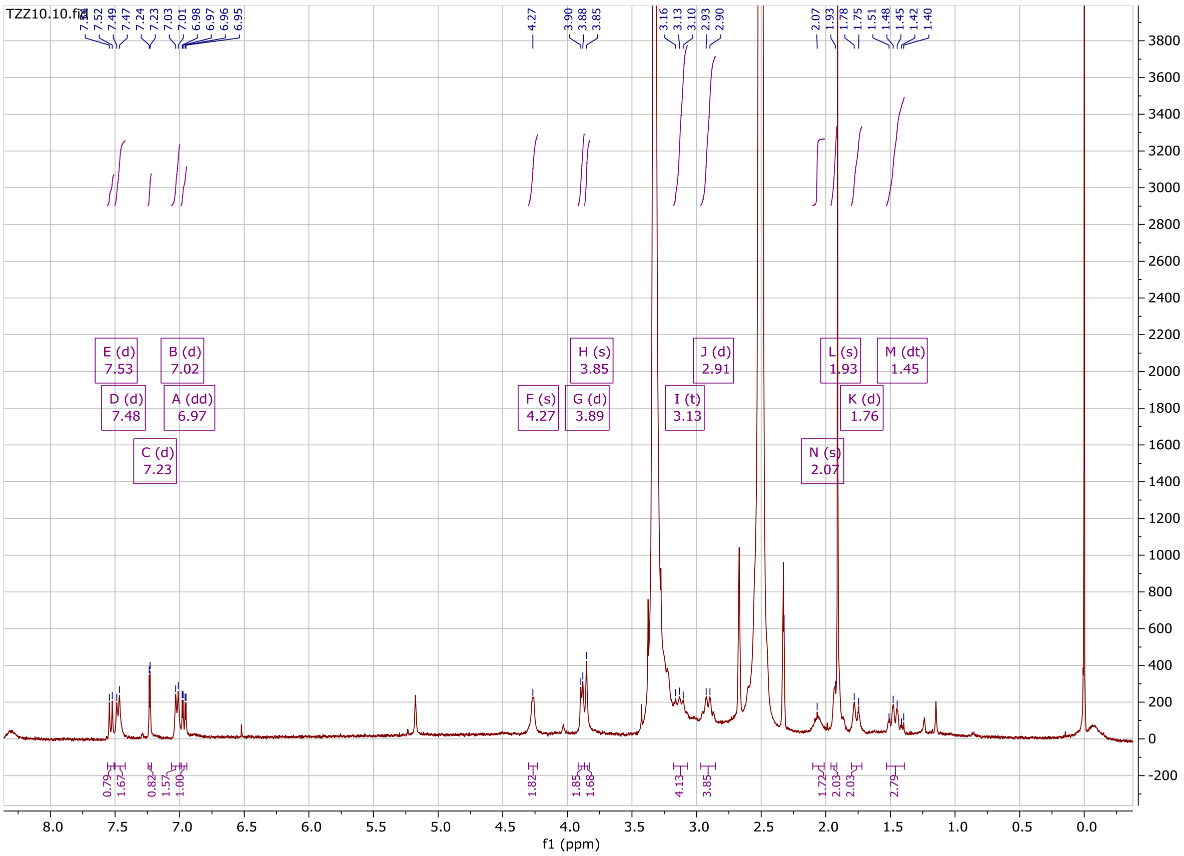

Figure S43. ^{1}H spectrum of compound **57**

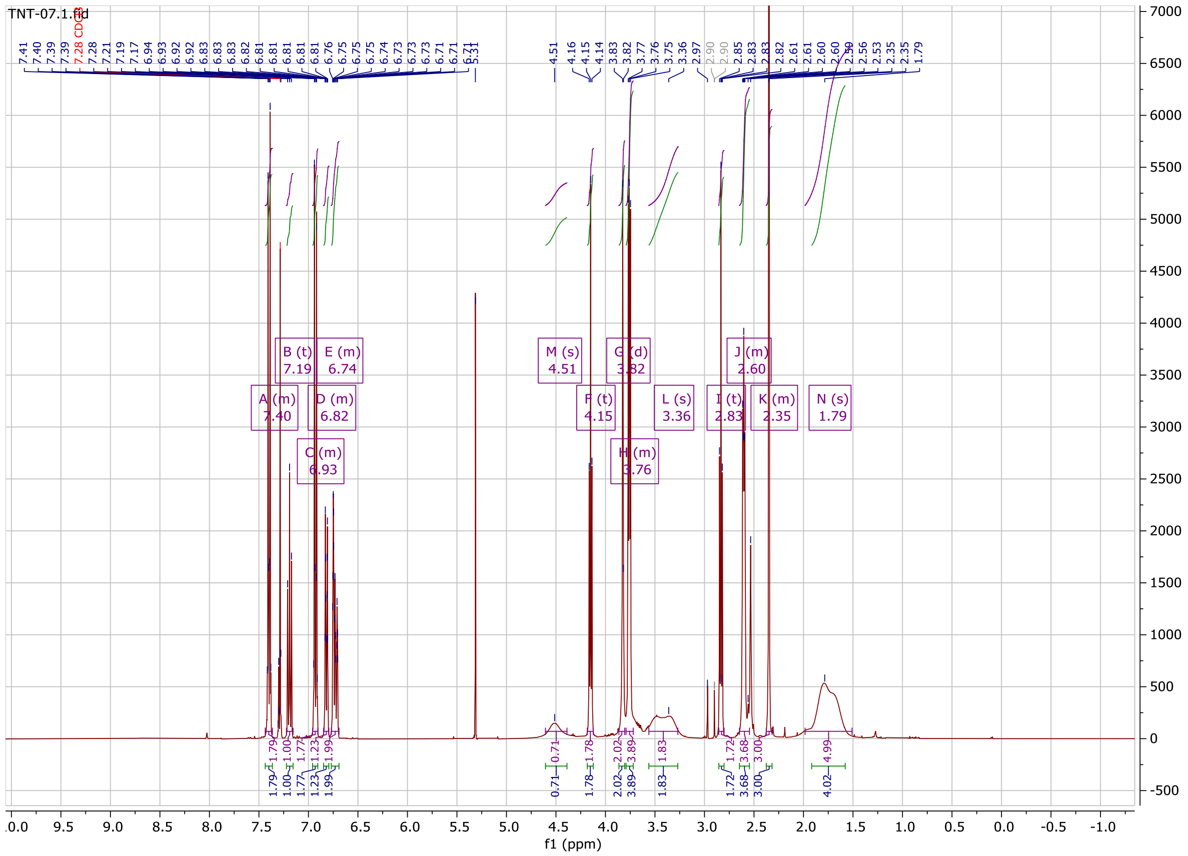

Figure S44. ^{1}H spectrum of compound **58**

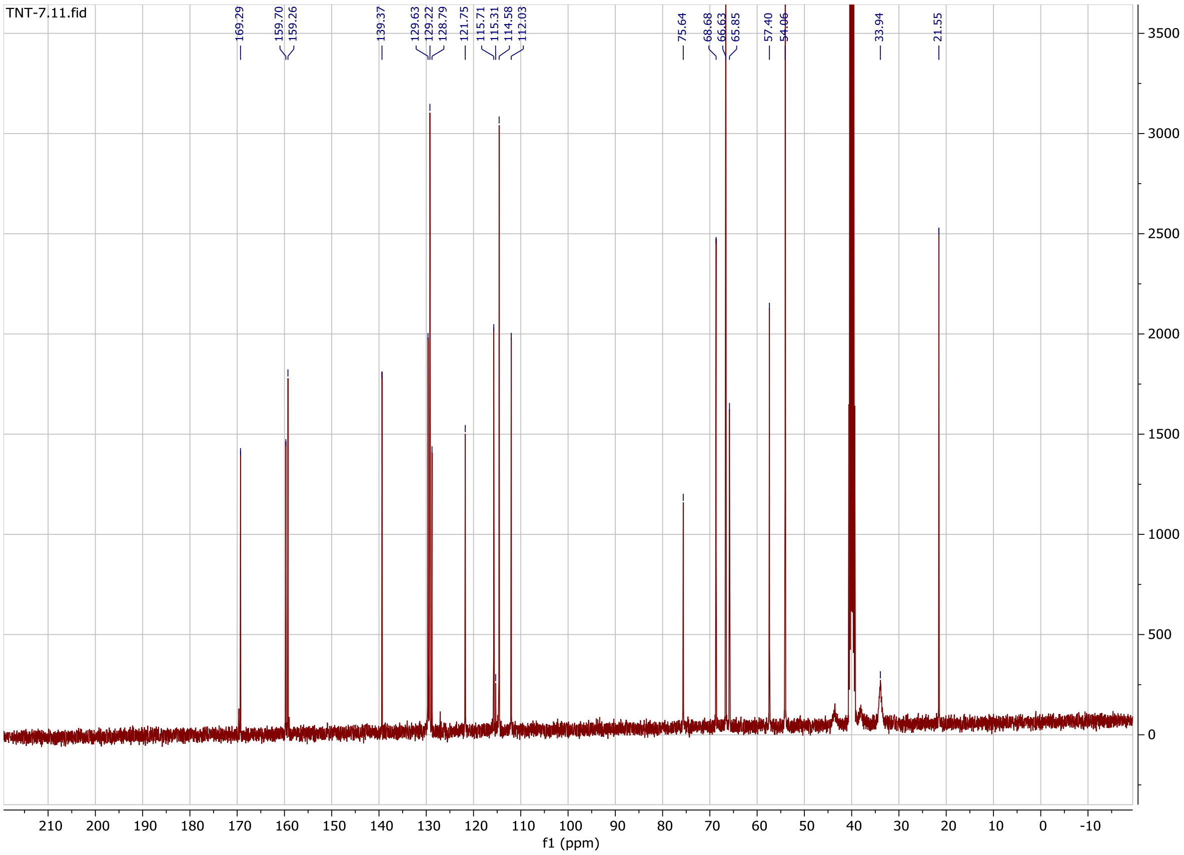

Figure S45. ^{13}C spectrum of compound **58**

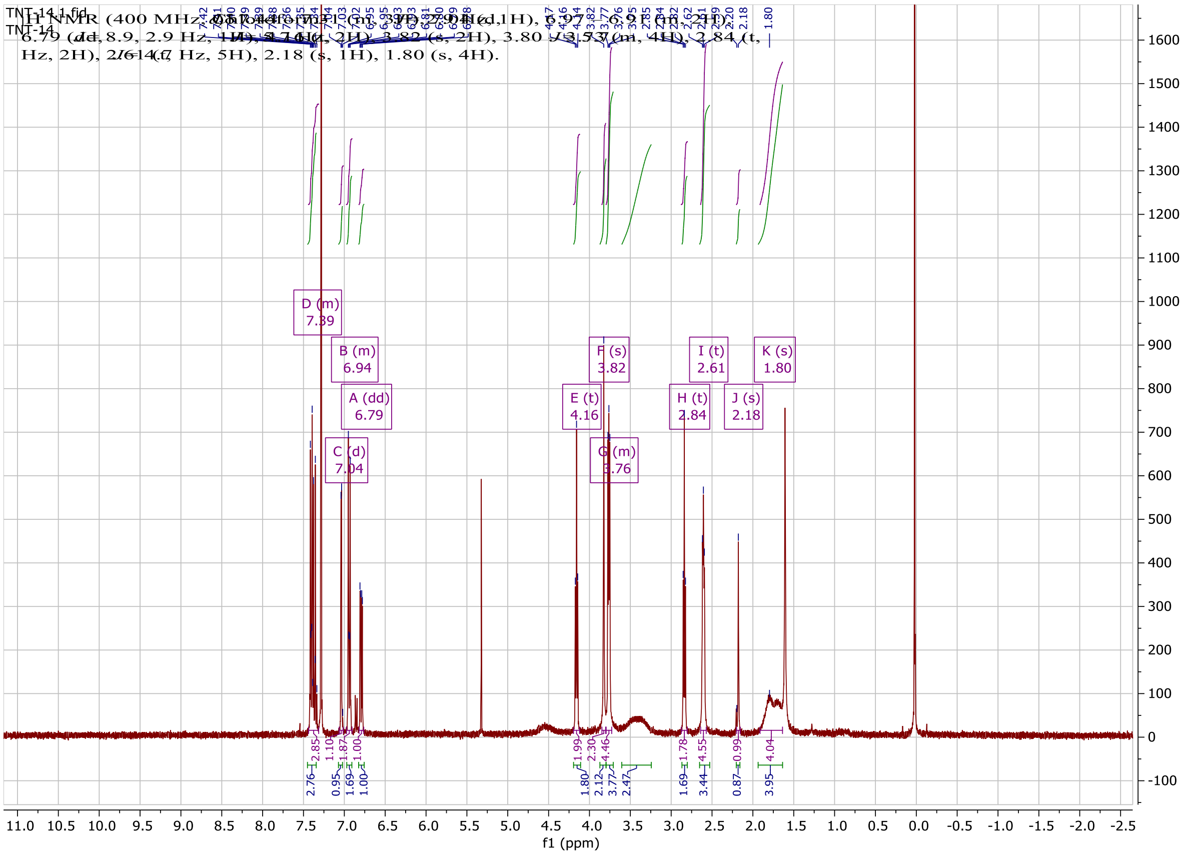

Figure S46. ^{1}H spectrum of compound **59**

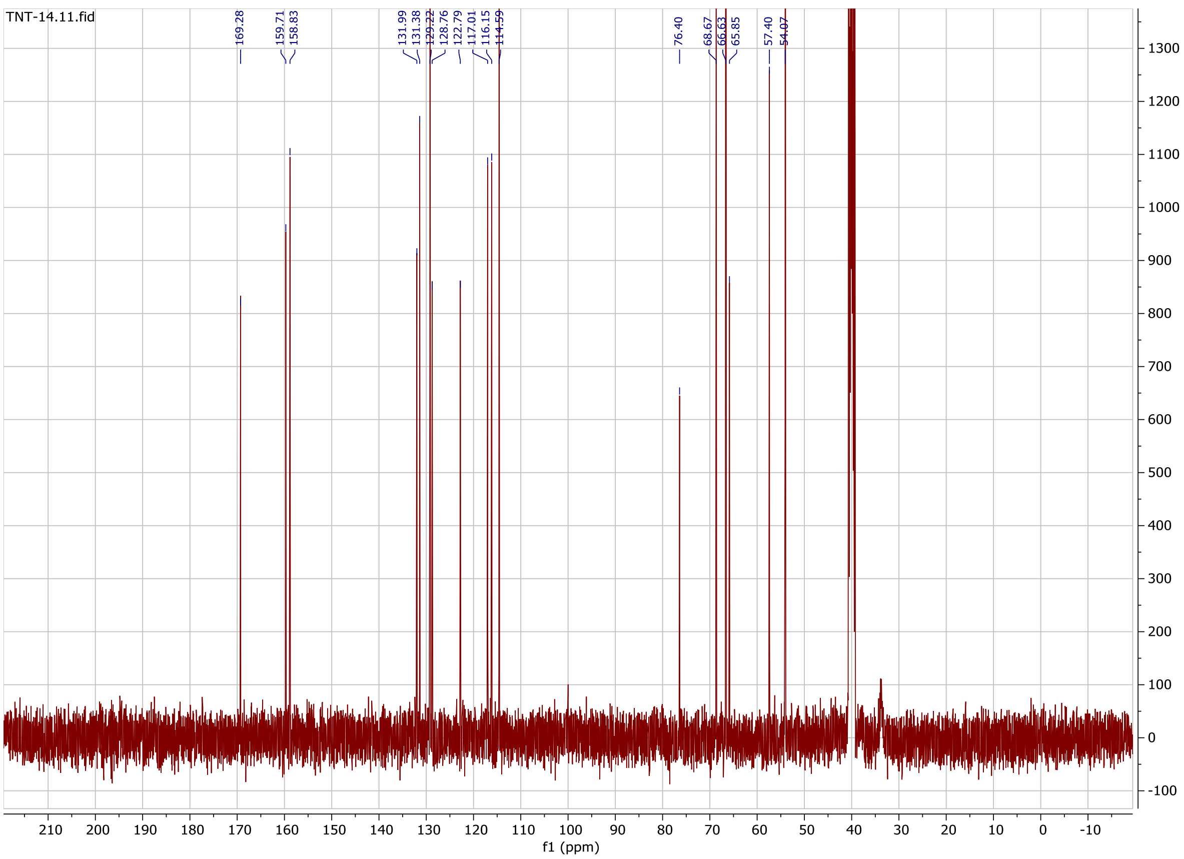

Figure S47. ^{13}C spectrum of compound **59**

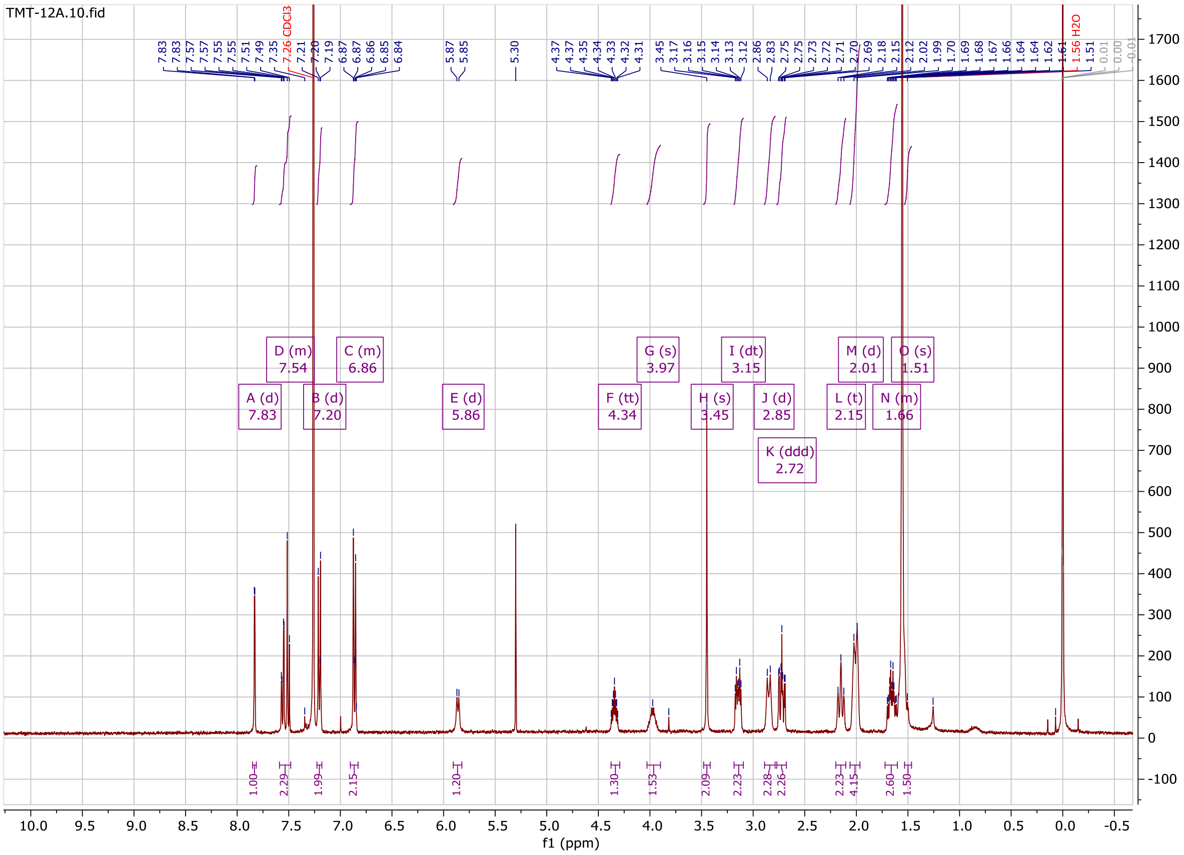

Figure S48. ^{1}H spectrum of compound **66**

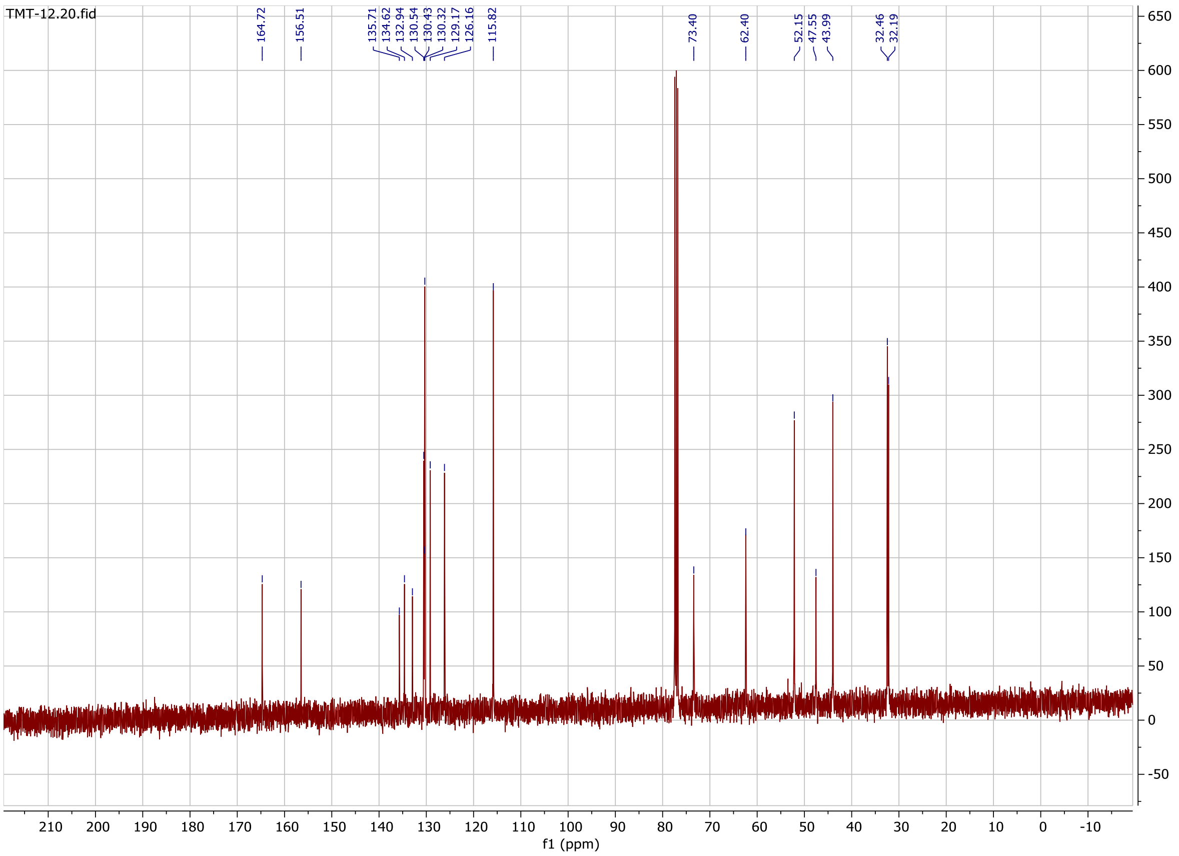

Figure S49. ^{13}C spectrum of compound **66**

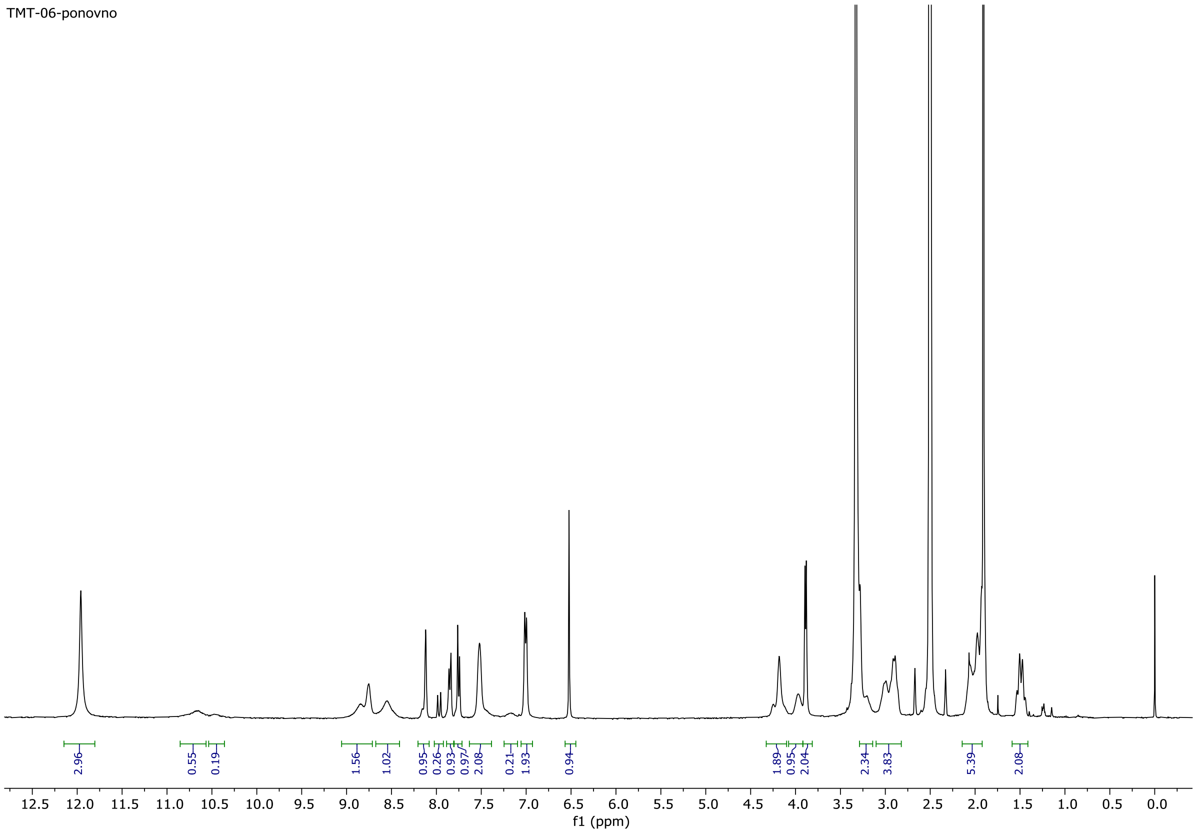

Figure S50. ^{1}H spectrum of compound **67**

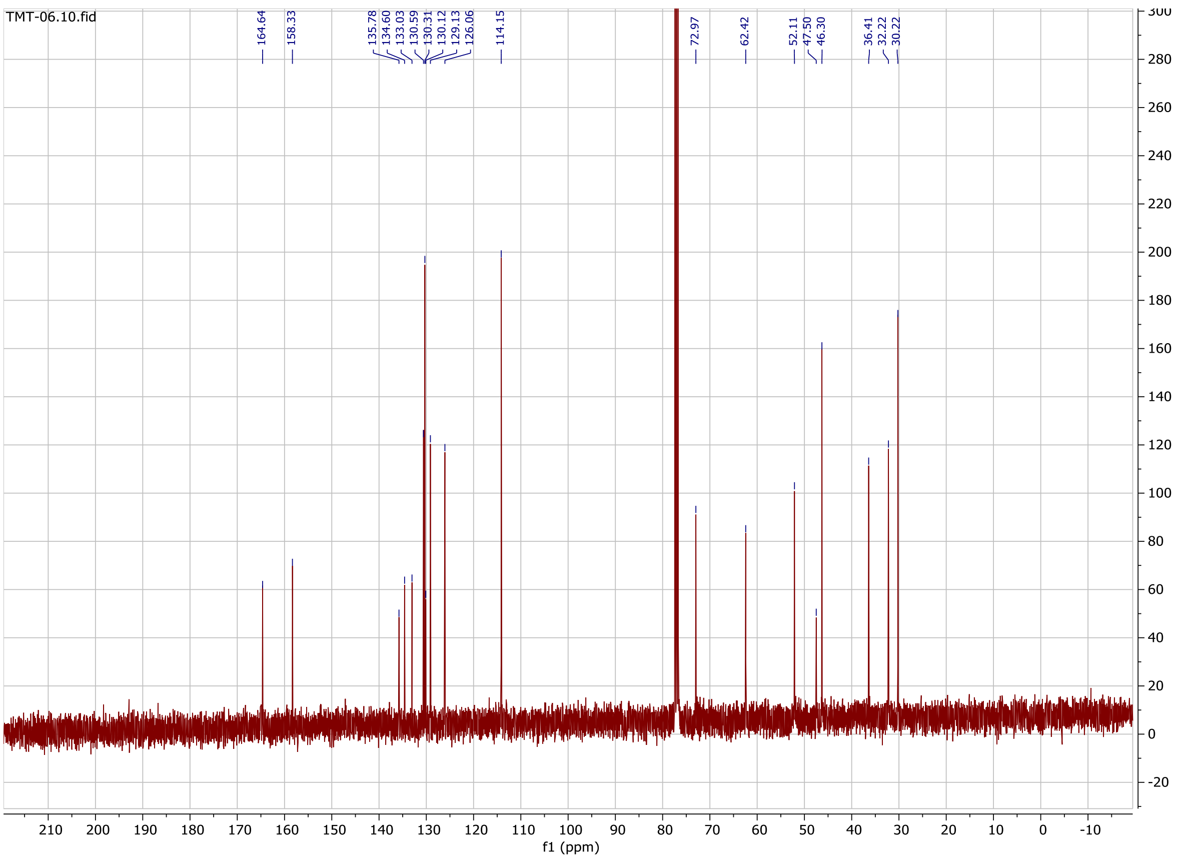

Figure S51. ^{13}C spectrum of compound **67**

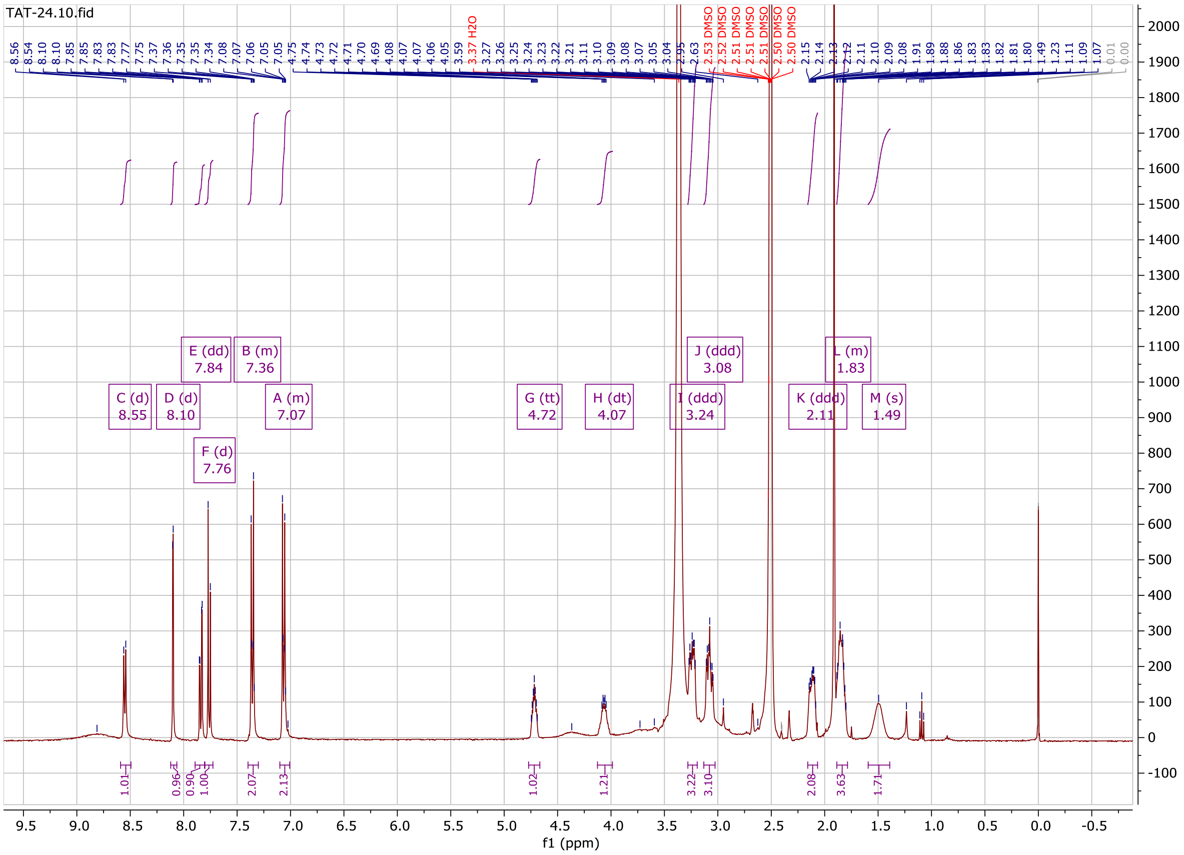

Figure S52. ^{1}H spectrum of compound **68**

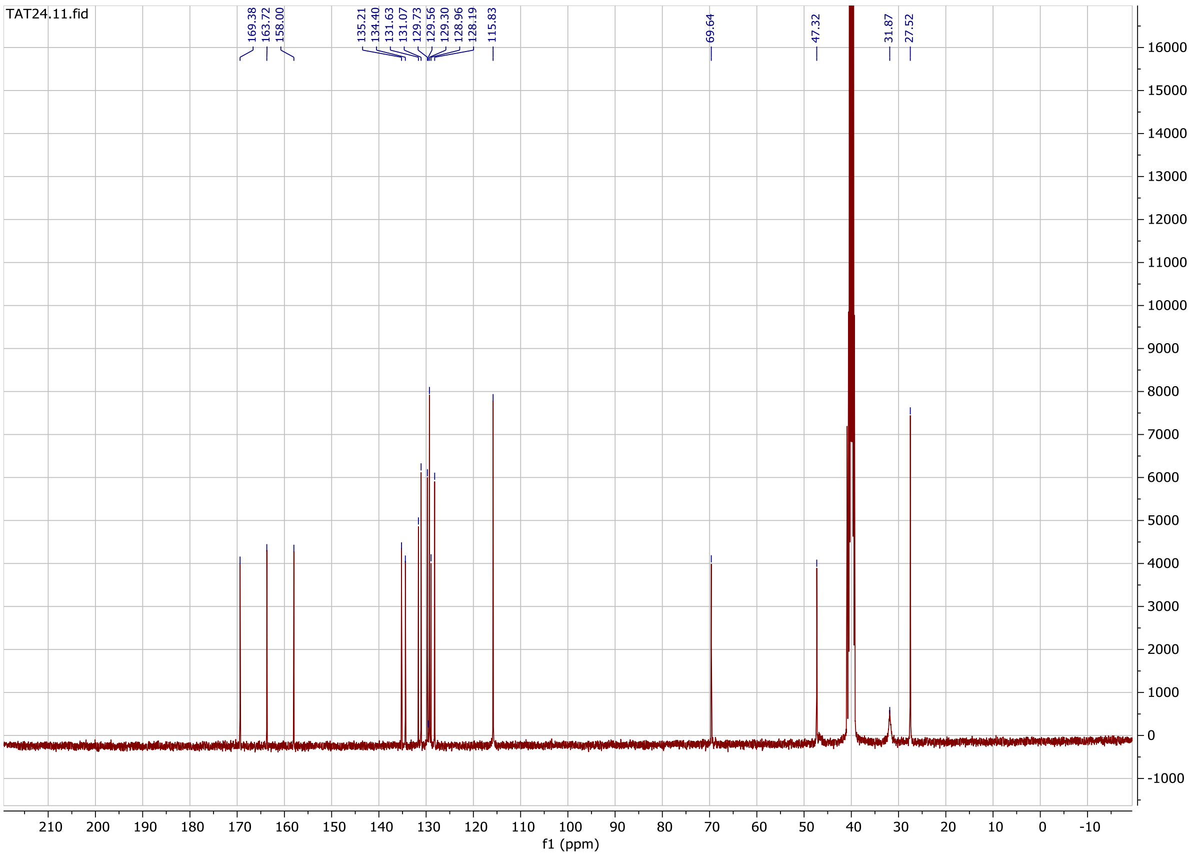

Figure S53. ^{13}C spectrum of compound **68**

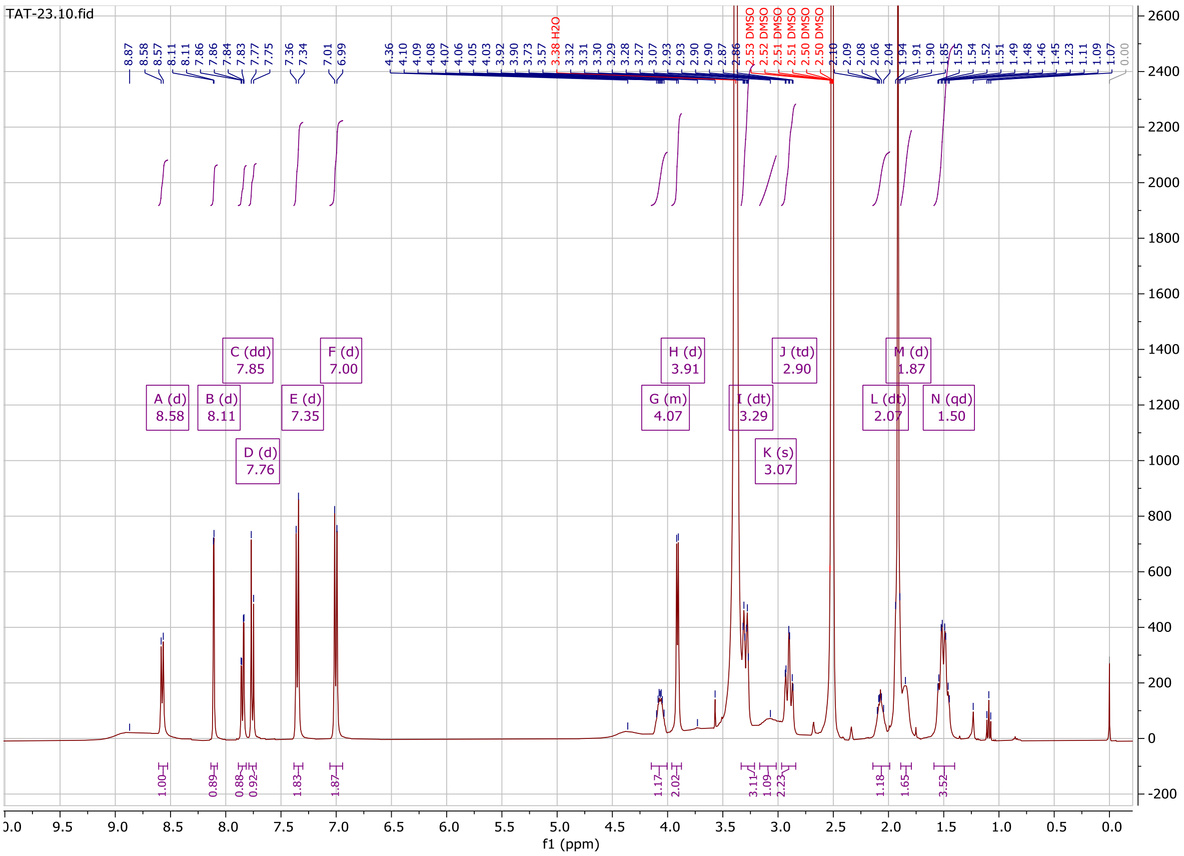

Figure S54. ^{1}H spectrum of compound **69**

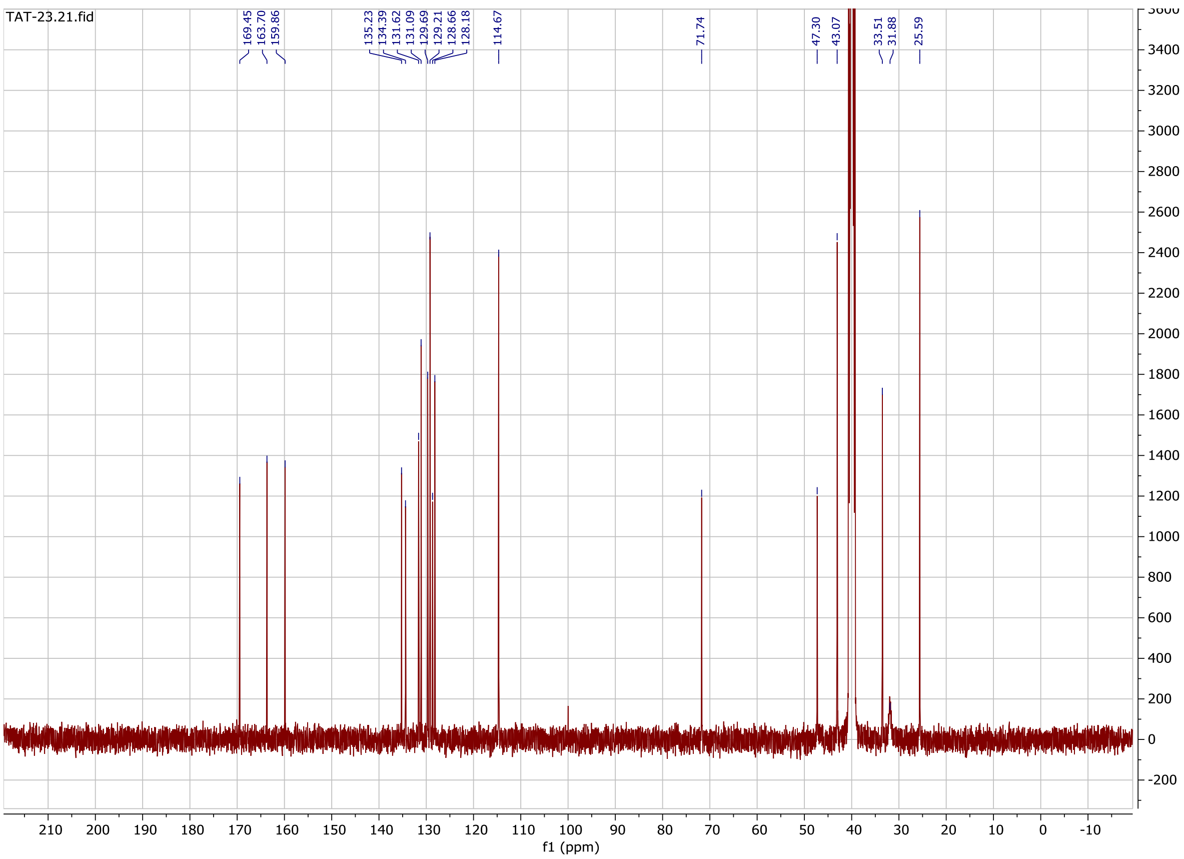

Figure S55. ^{13}C spectrum of compound **69**

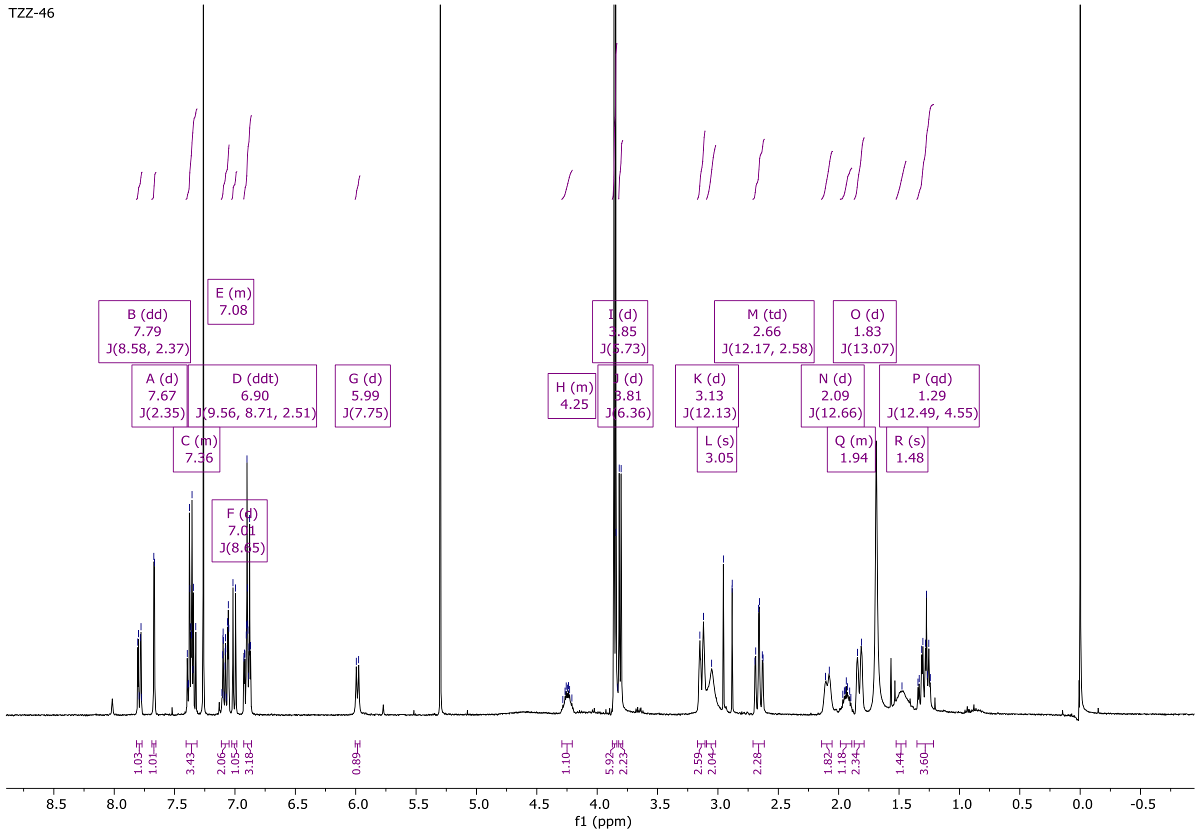

Figure S56. ^{1}H spectrum of compound **112**

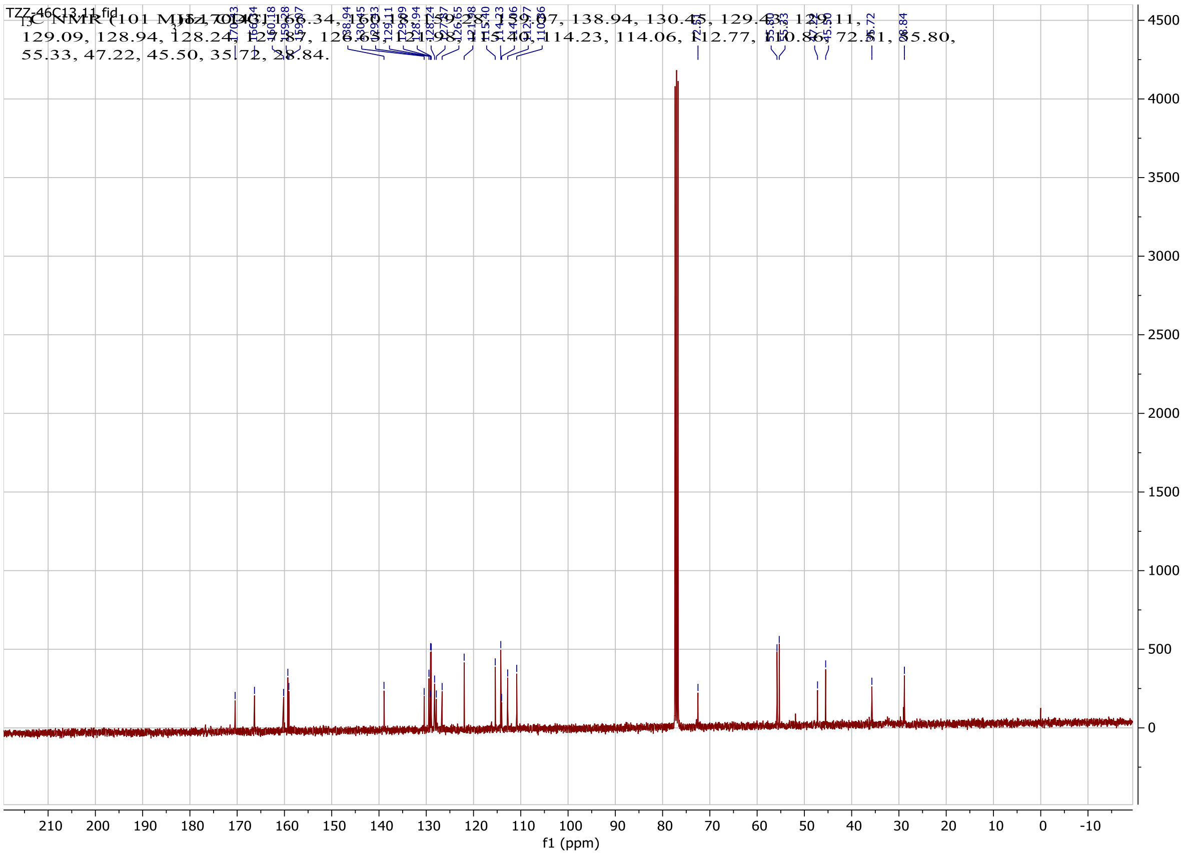

Figure S57. ^{13}C spectrum of compound **112**

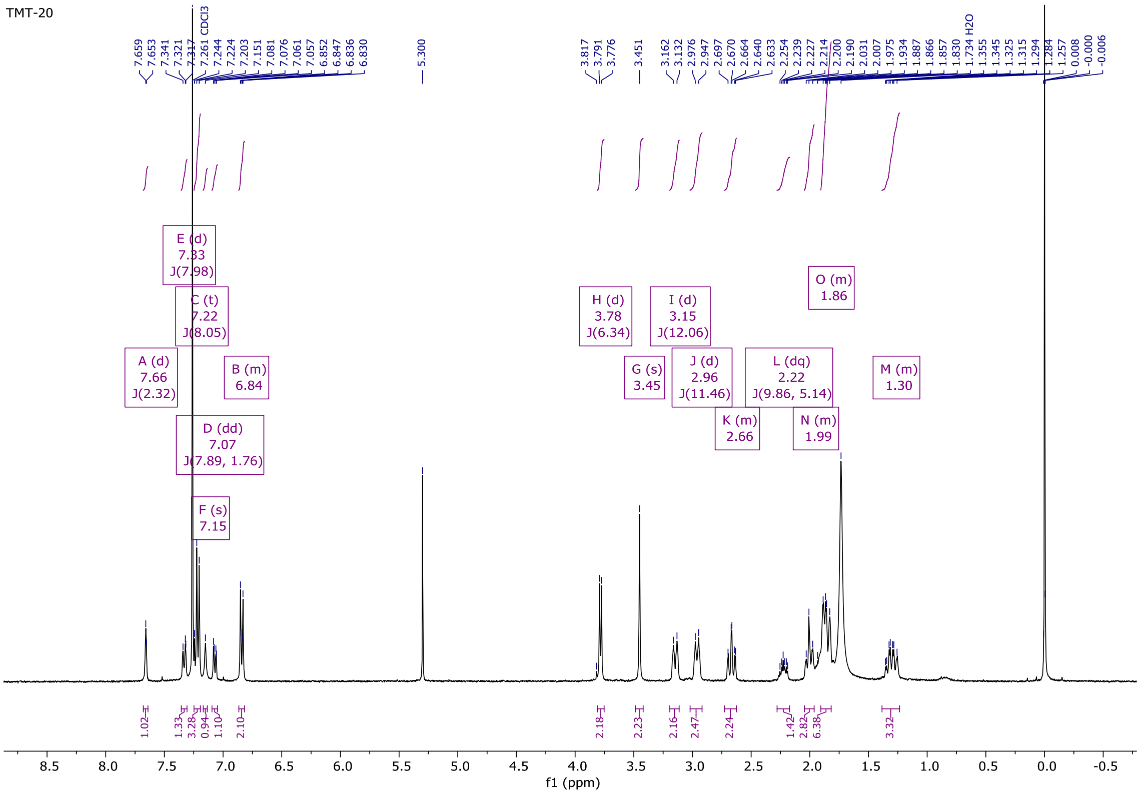

Figure S58. ^{1}H spectrum of compound **88**

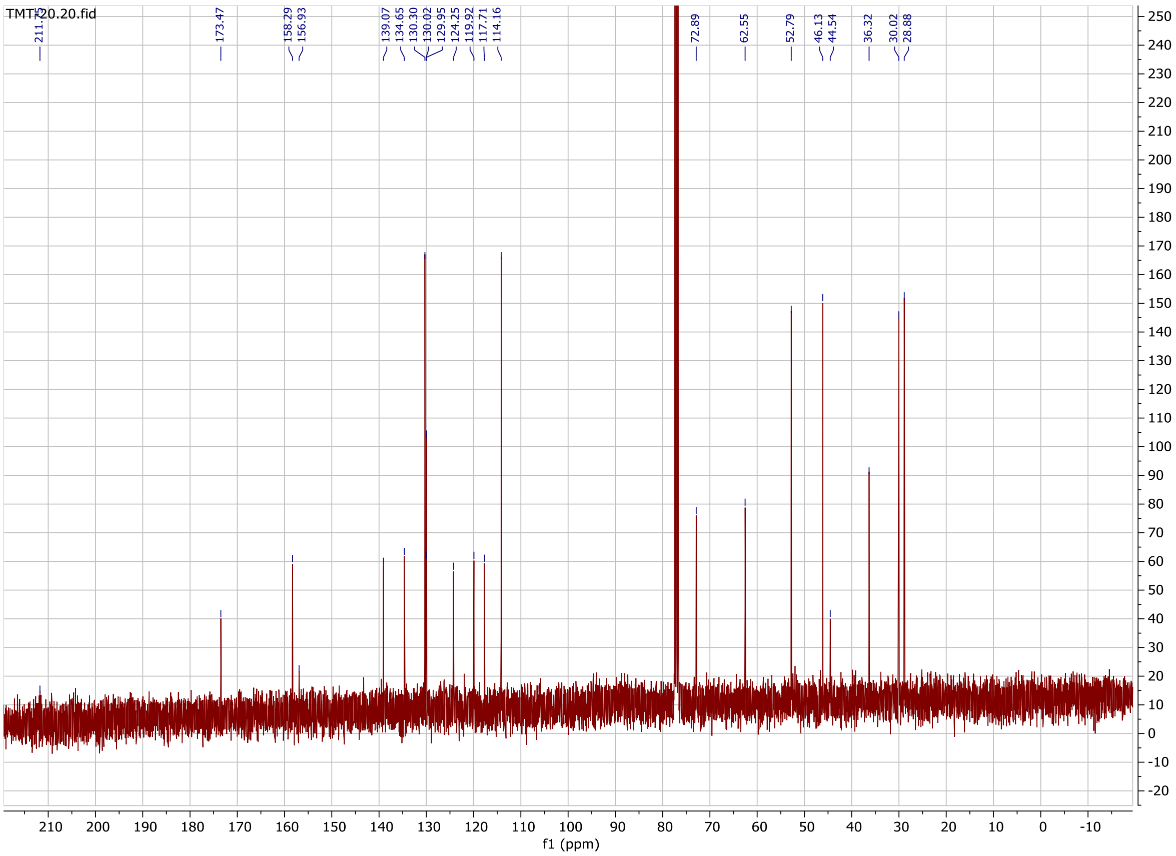

Figure S59. ^{13}C spectrum of compound **88**

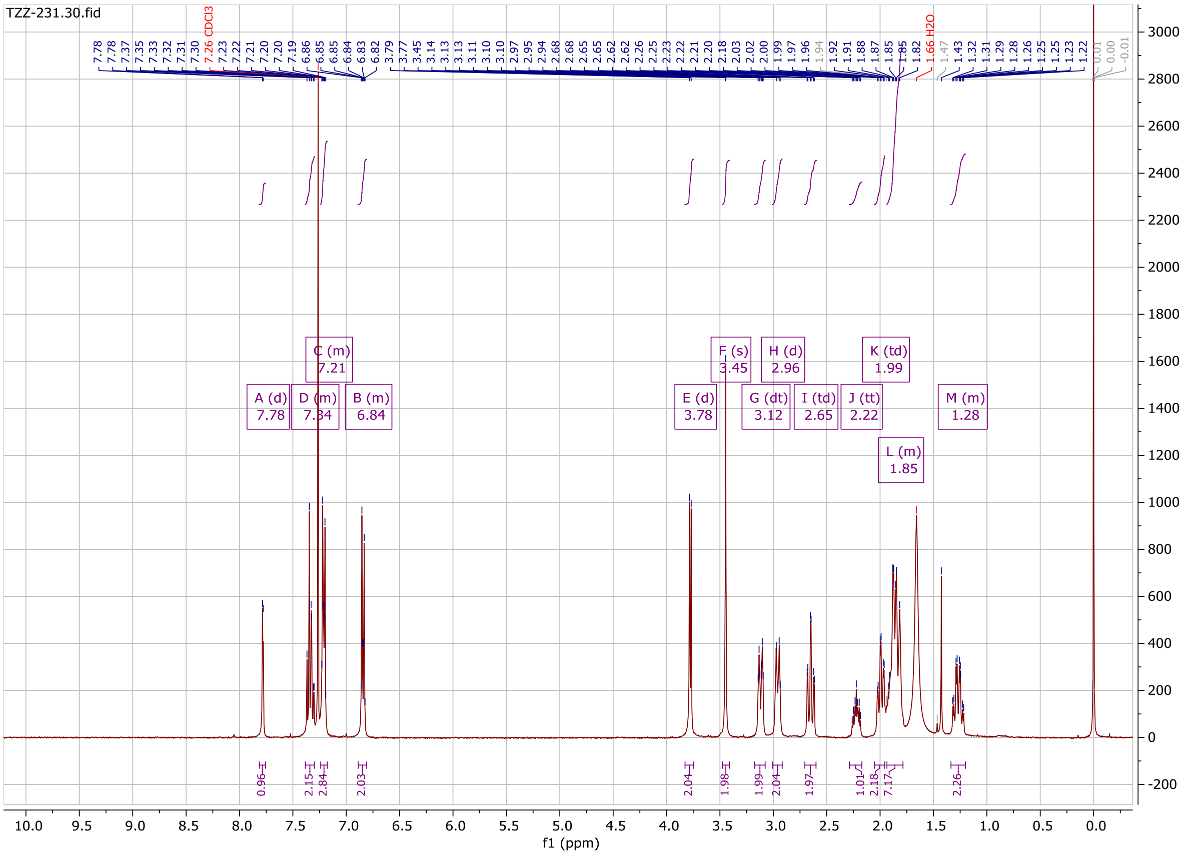

Figure S60. ^{1}H spectrum of compound **89**

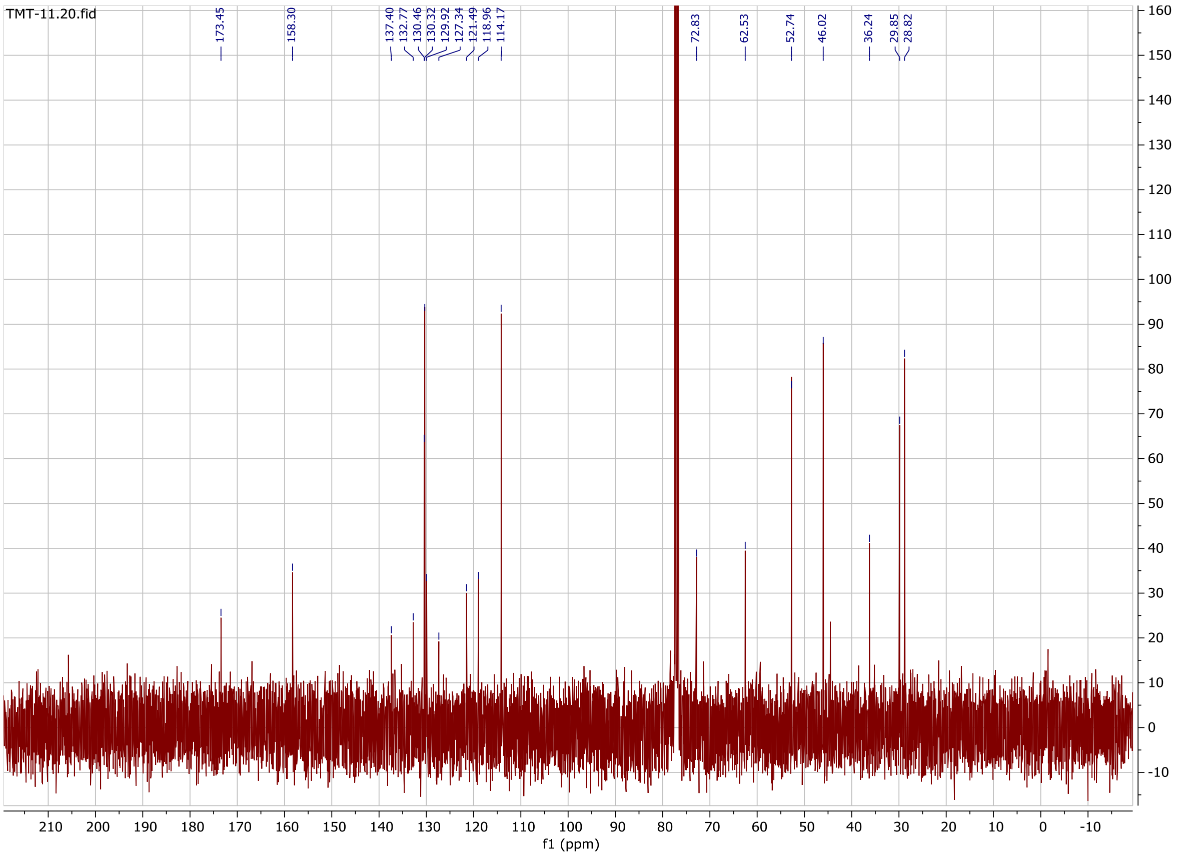

Figure S61. ^{13}C spectrum of compound **89**

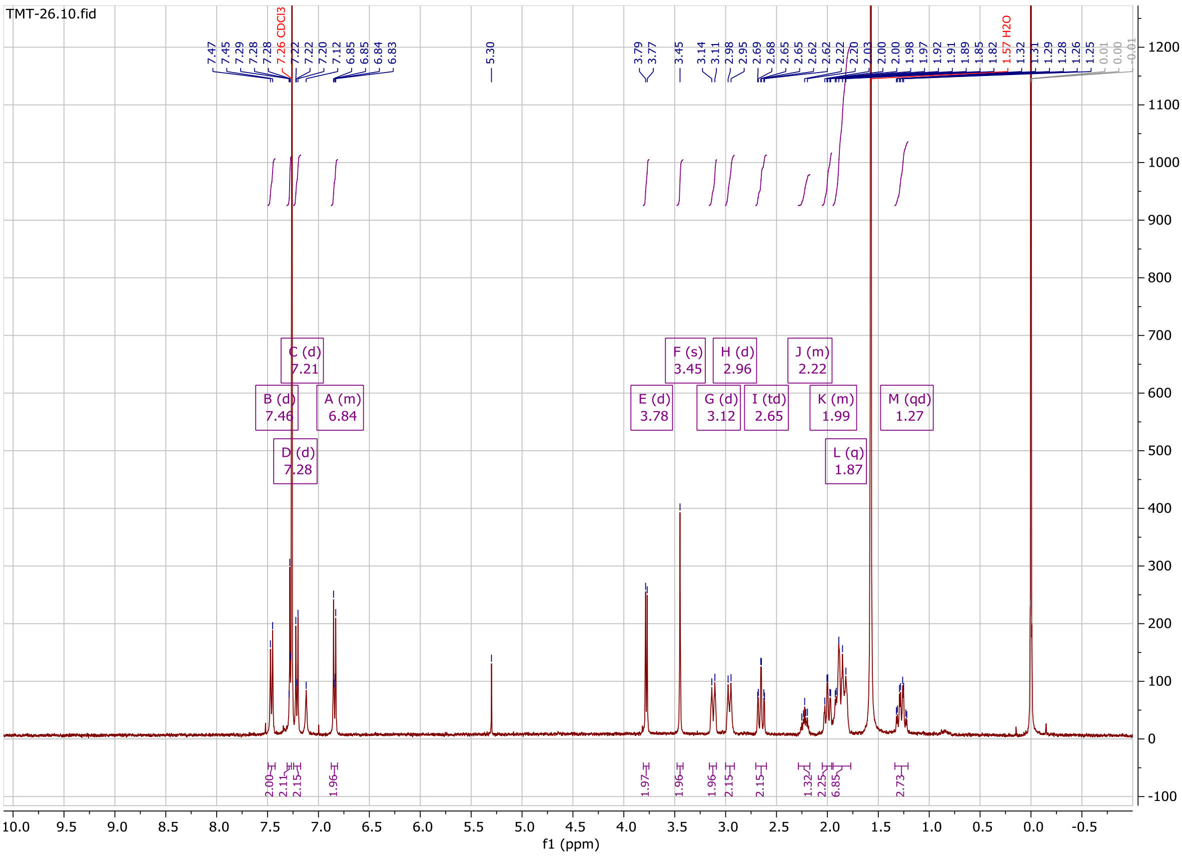

Figure S62. ^{1}H spectrum of compound **90**

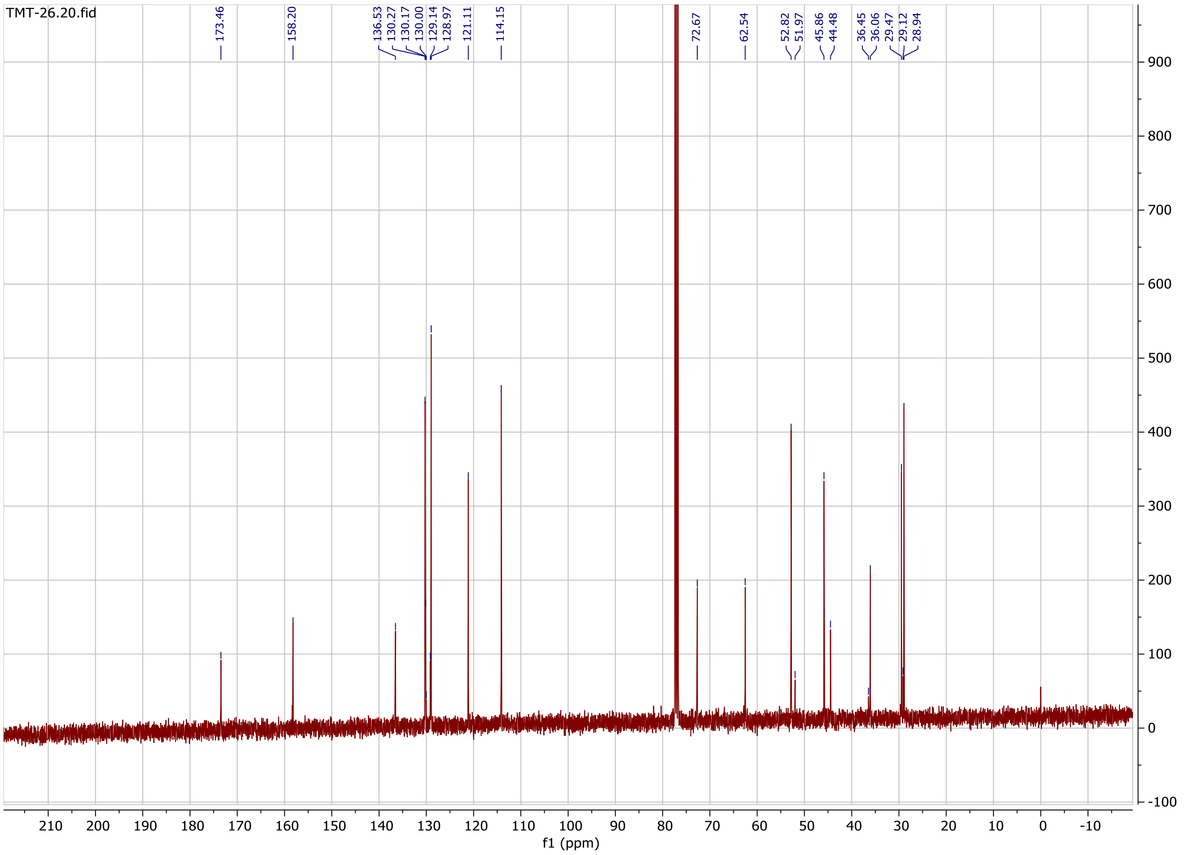

Figure S63. ^{13}C spectrum of compound **90**

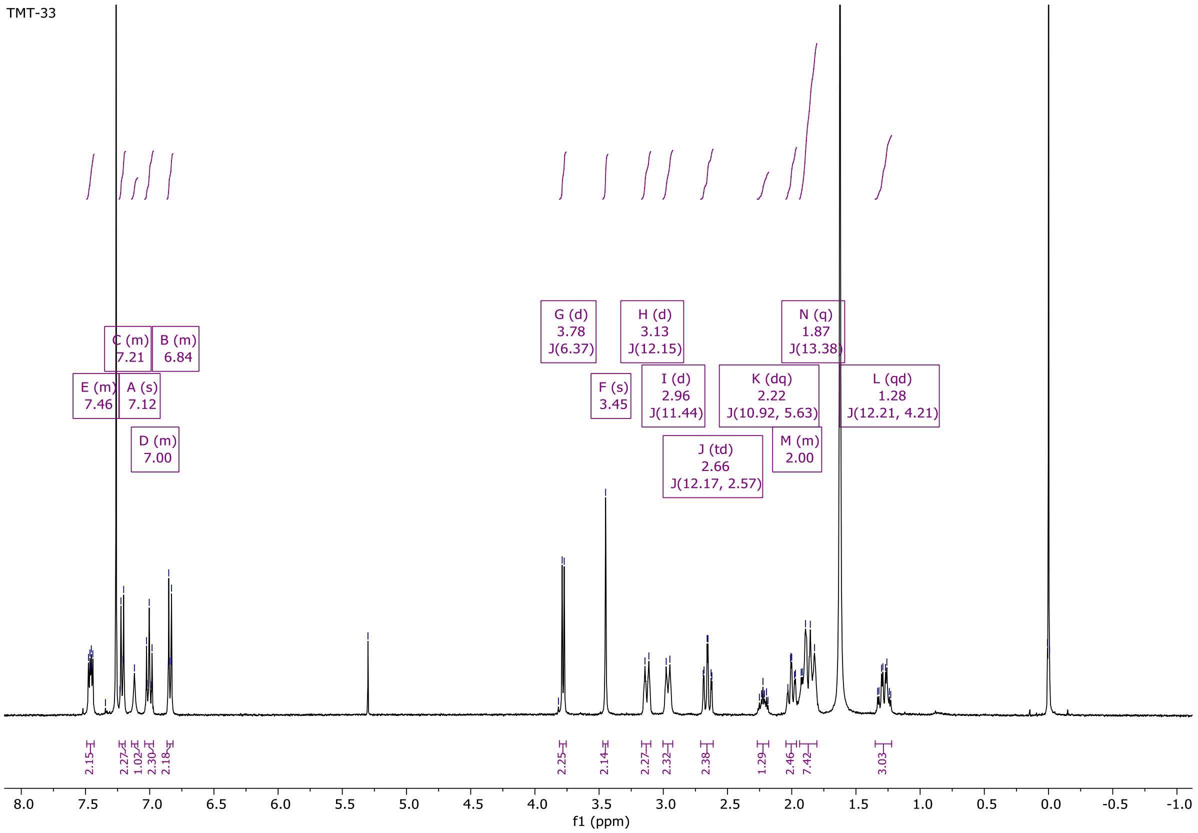

Figure S64. ^{1}H spectrum of compound **91**

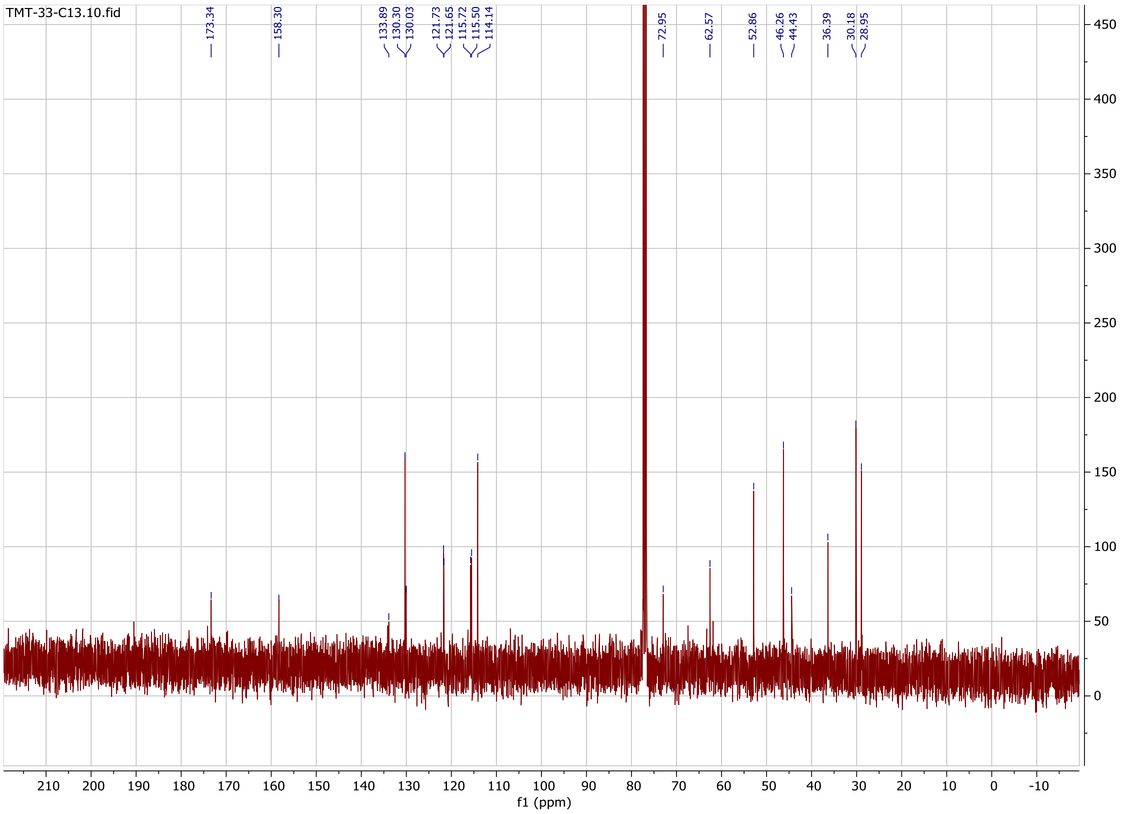

Figure S65. ^{13}C spectrum of compound **91**

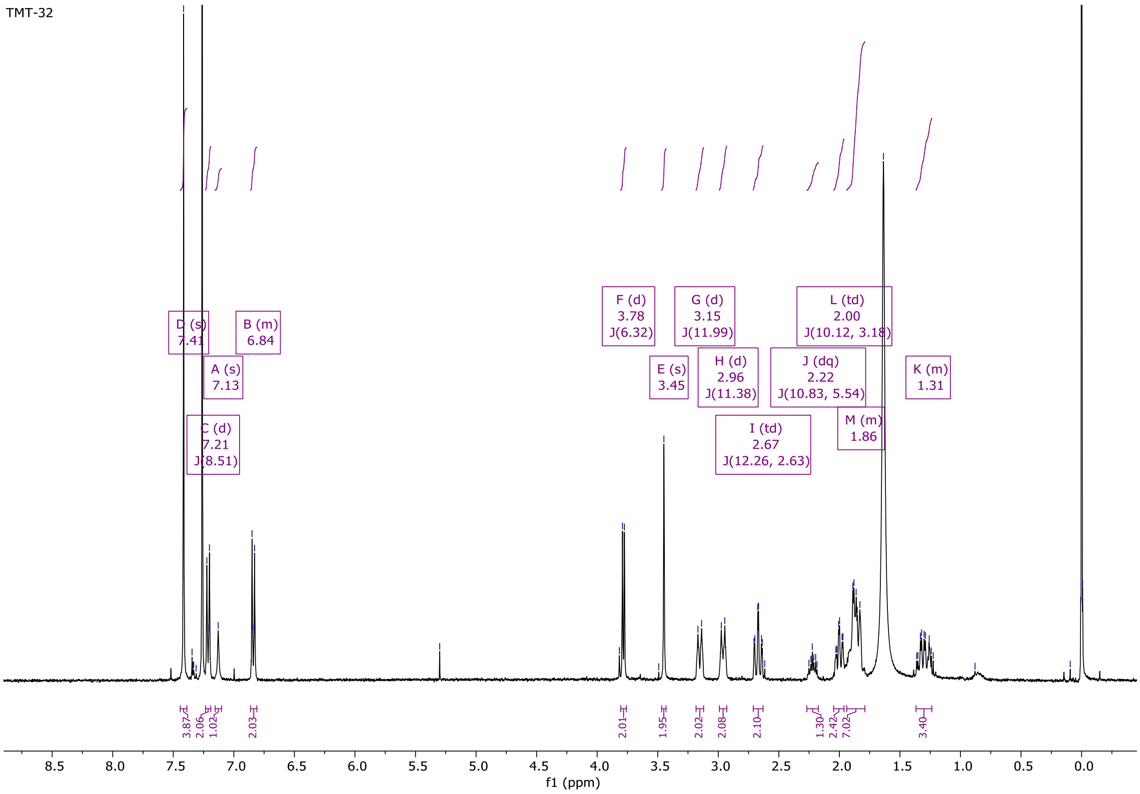

Figure S66. ^{1}H spectrum of compound **92**

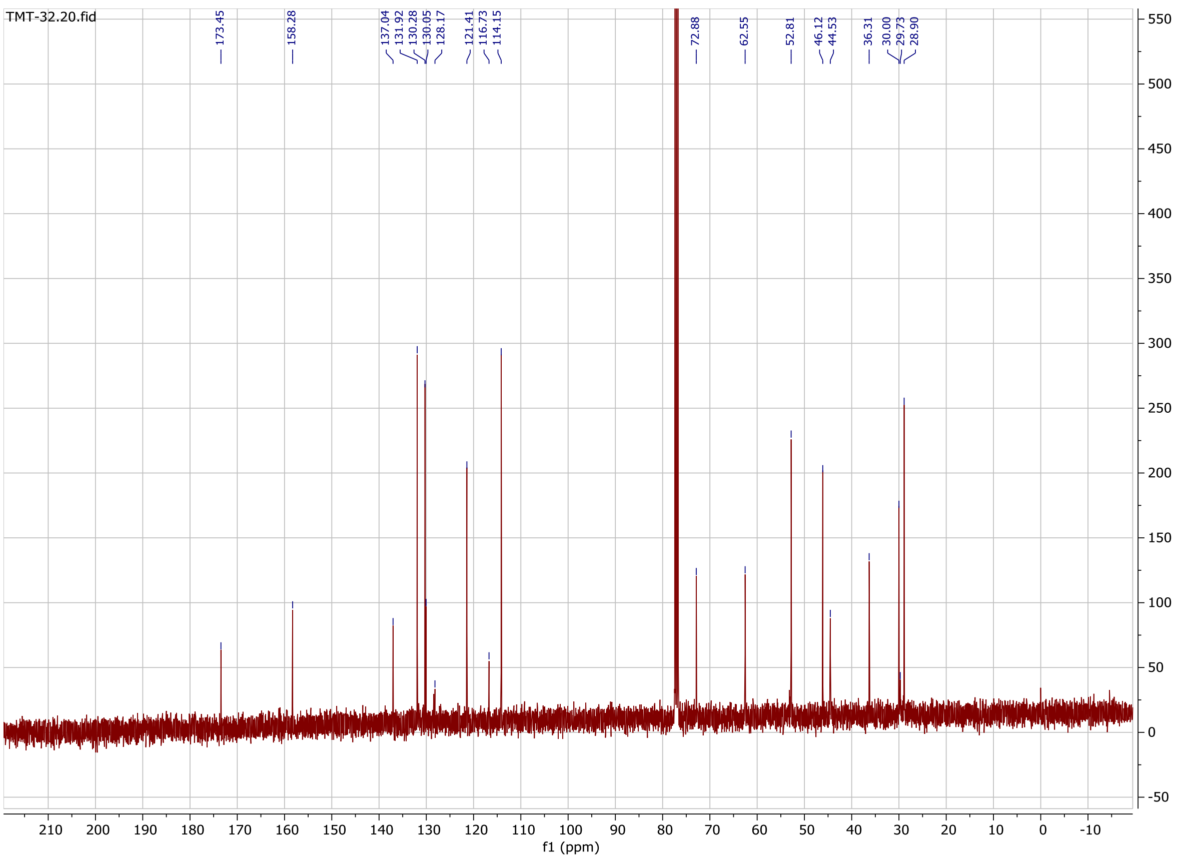

Figure S67. ^{13}C spectrum of compound **92**

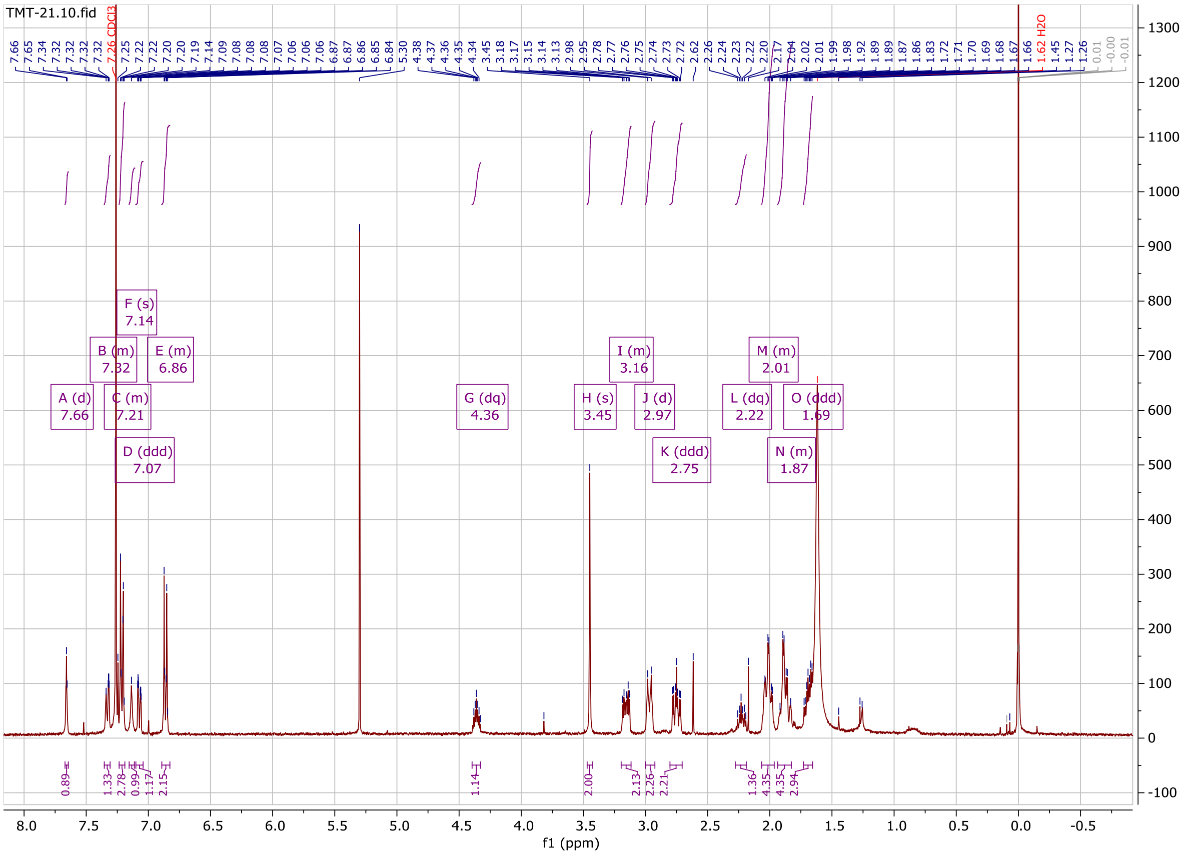

Figure S68. ^{1}H spectrum of compound **93**

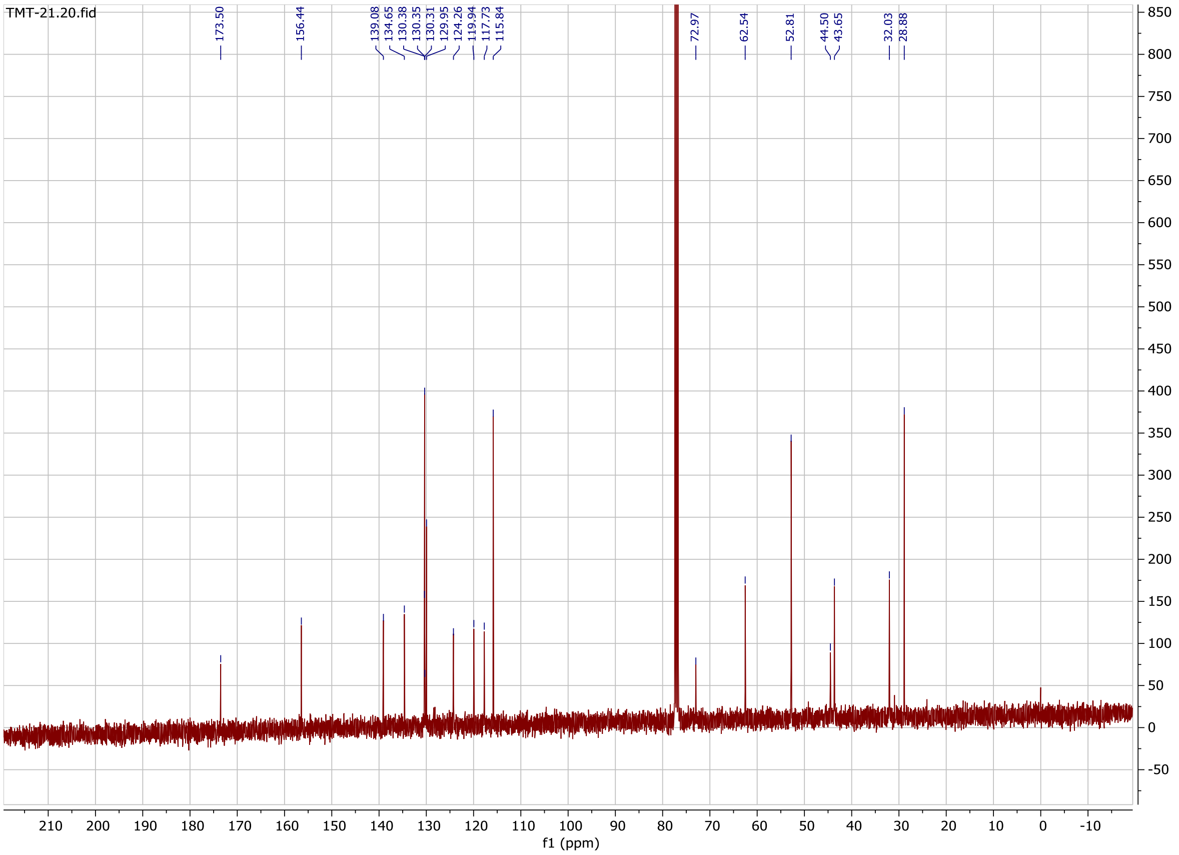

Figure S69. ^{13}C spectrum of compound **93**

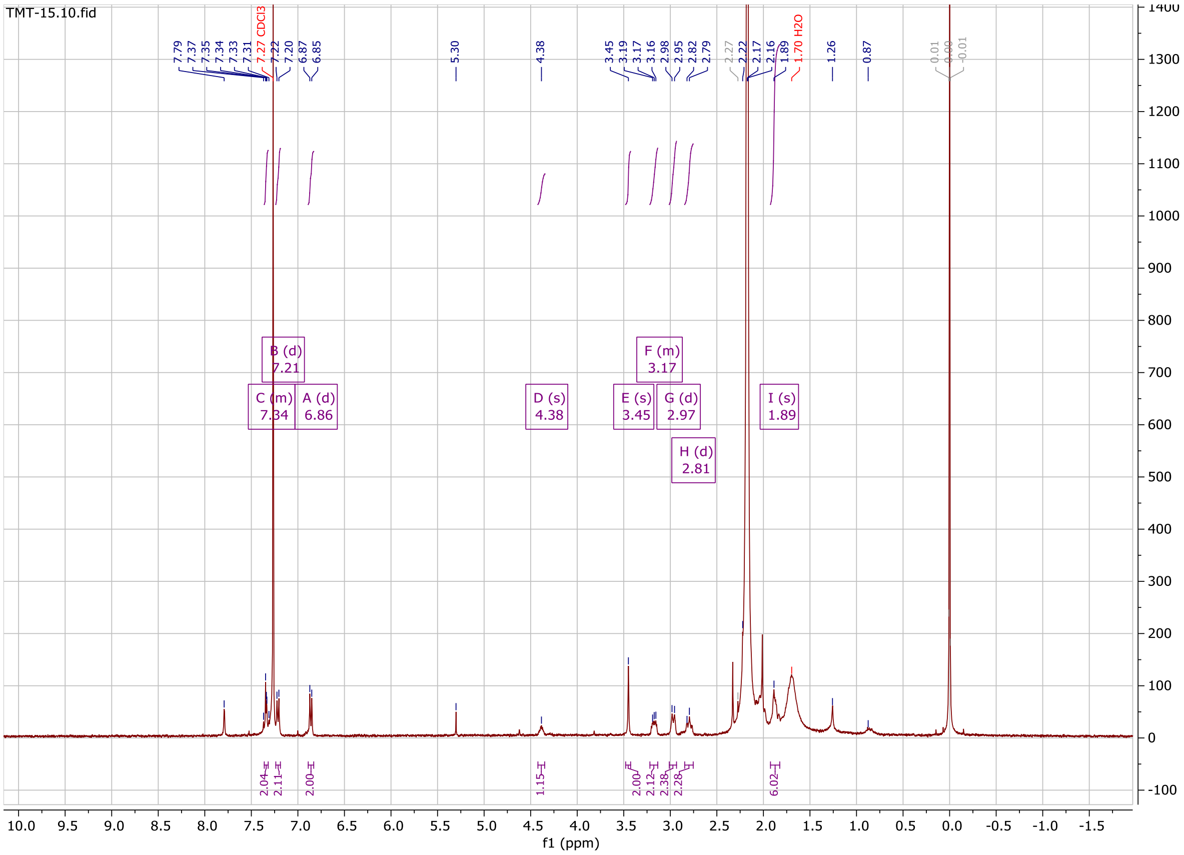

Figure S70. ^{1}H spectrum of compound **94**

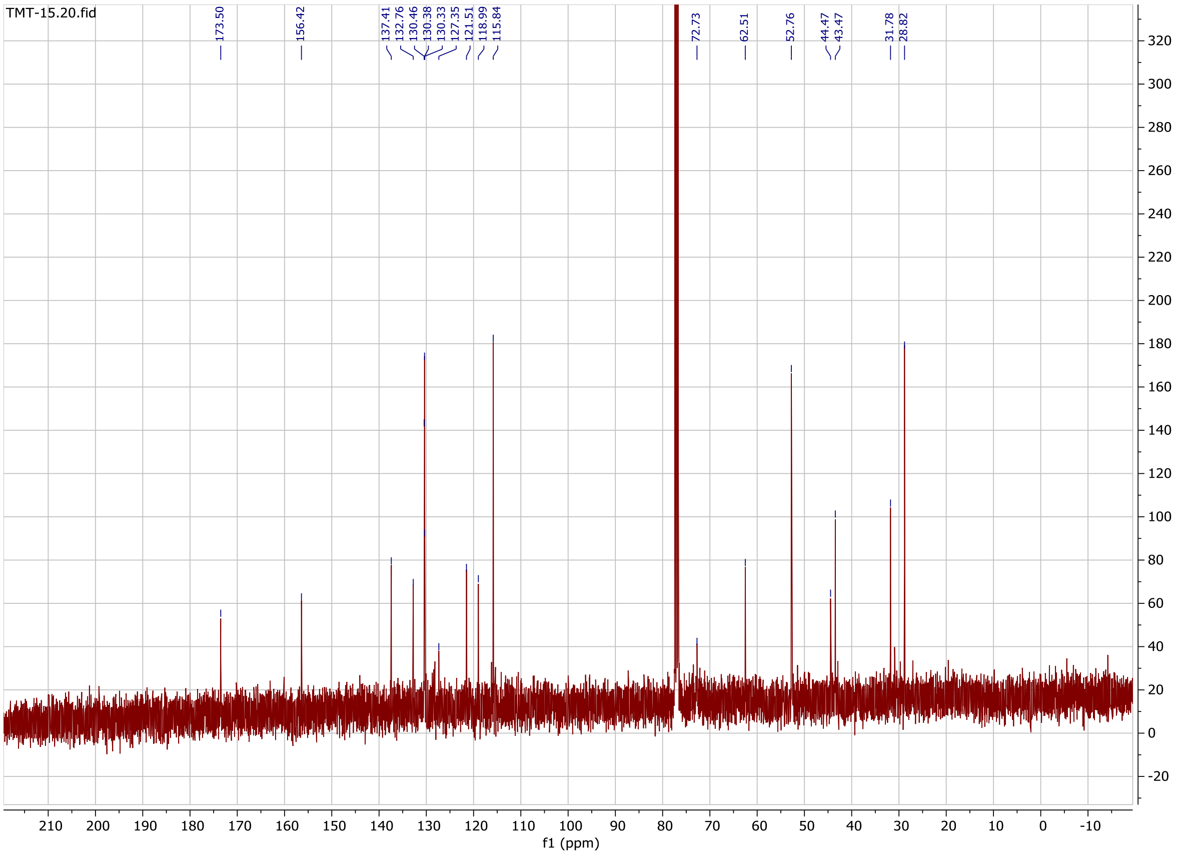

Figure S71. ^{13}C spectrum of compound **94**

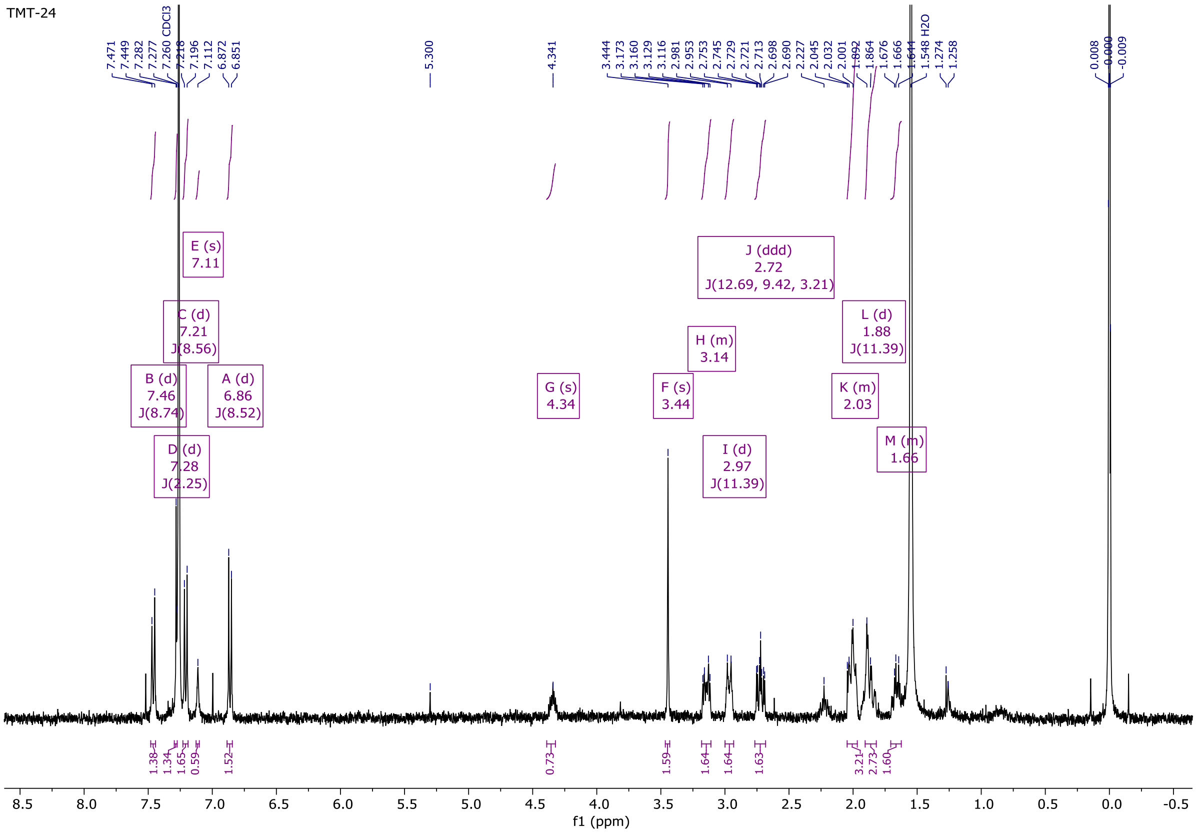

Figure S72. ^{1}H spectrum of compound **95**

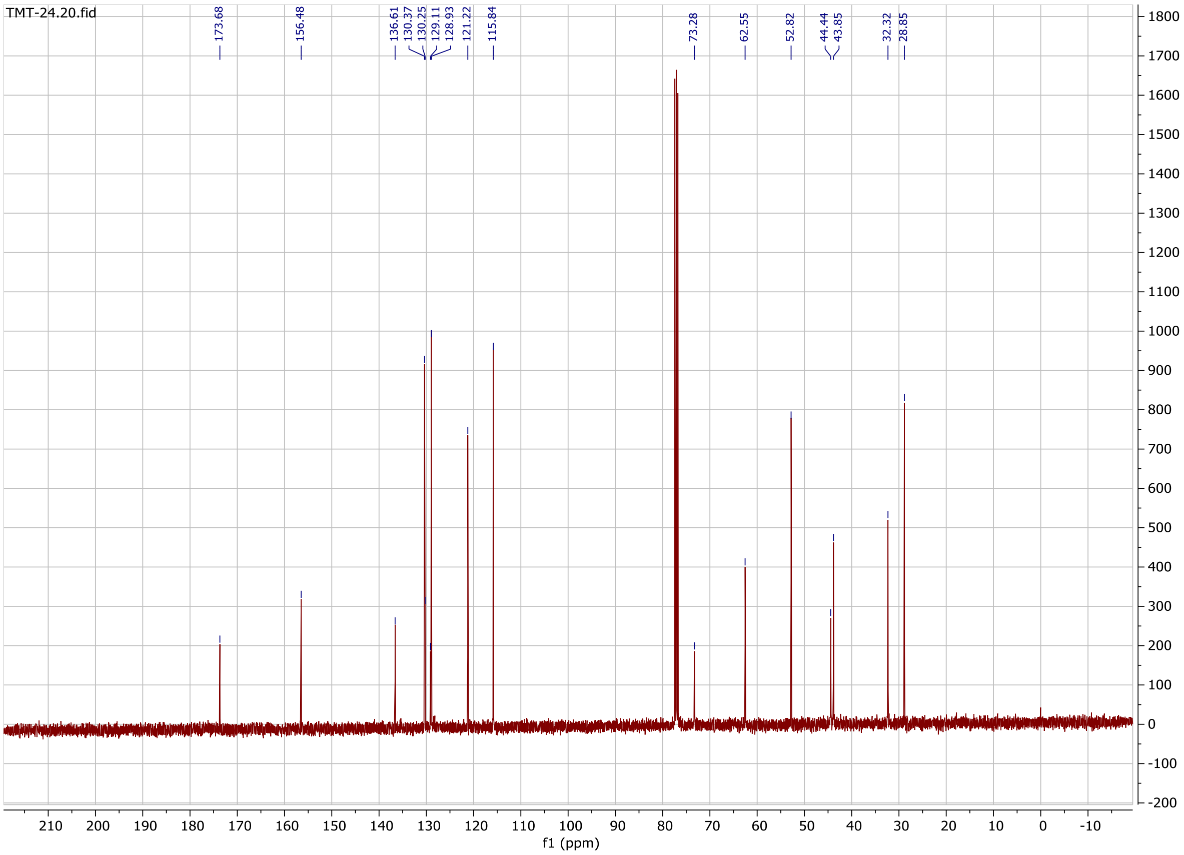

Figure S73. ^{13}C spectrum of compound **95**

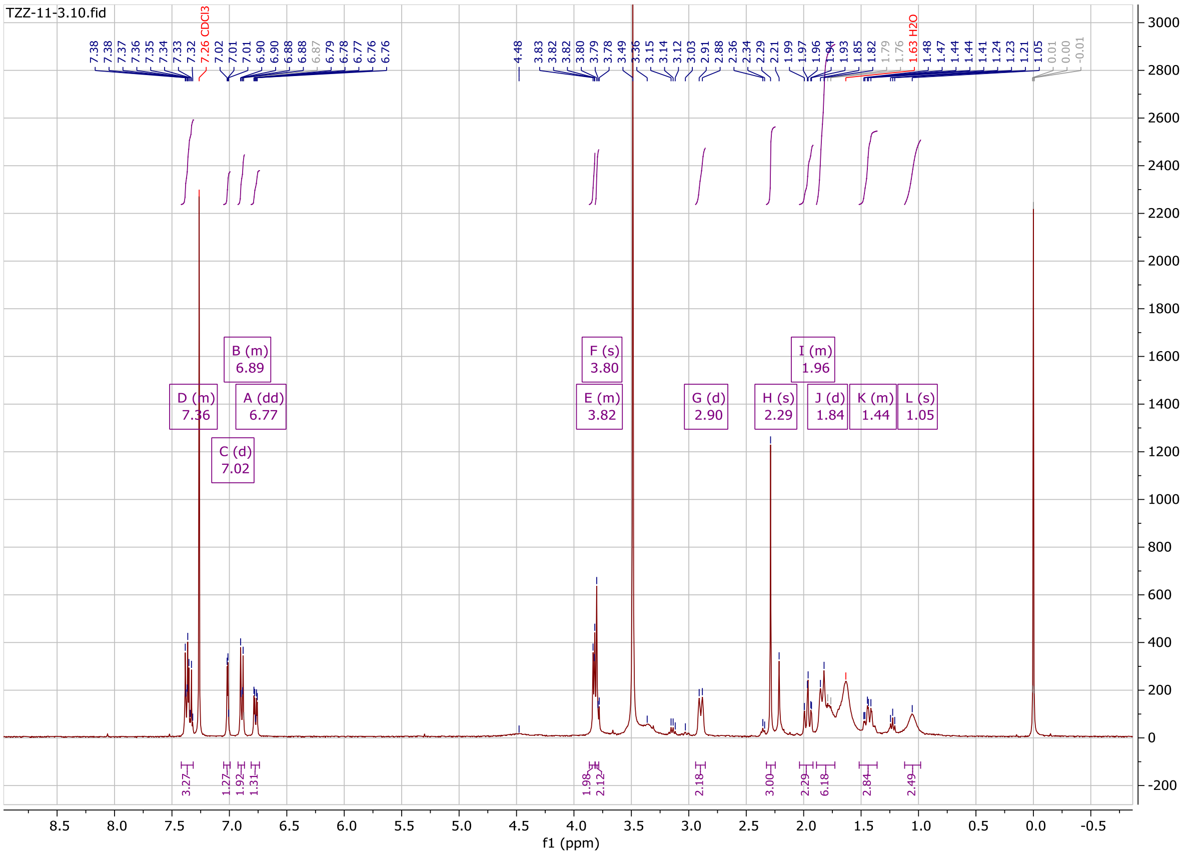

Figure S74. ^{1}H spectrum of compound **96**

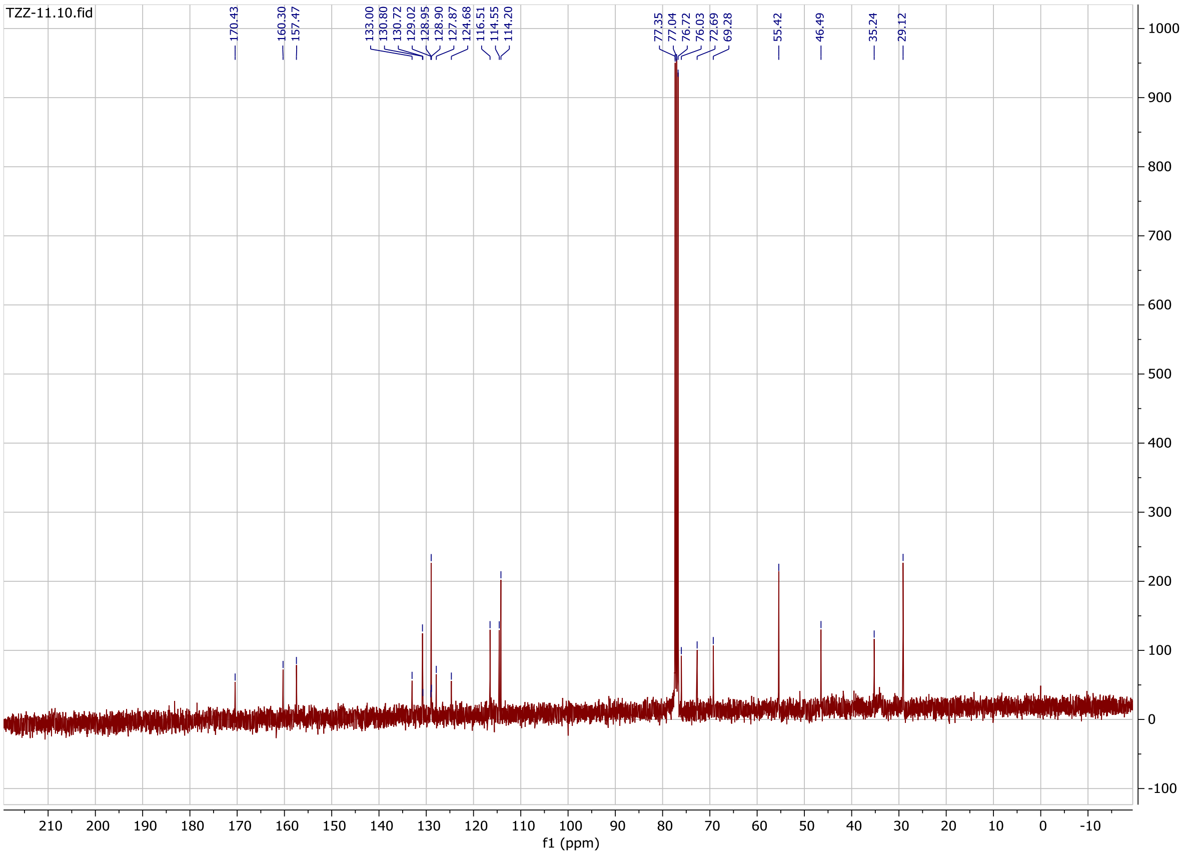

Figure S75. ^{13}C spectrum of compound **96**

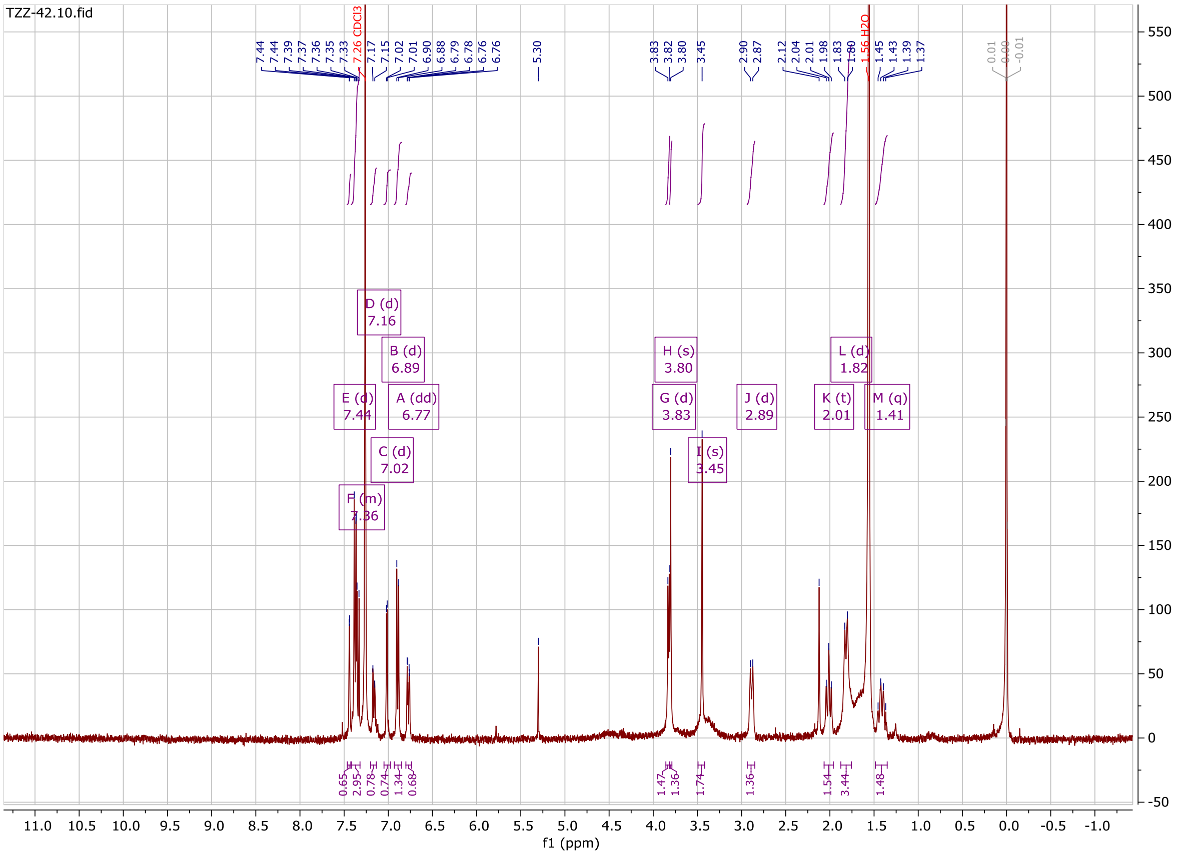

Figure S76. ^{1}H spectrum of compound **97**

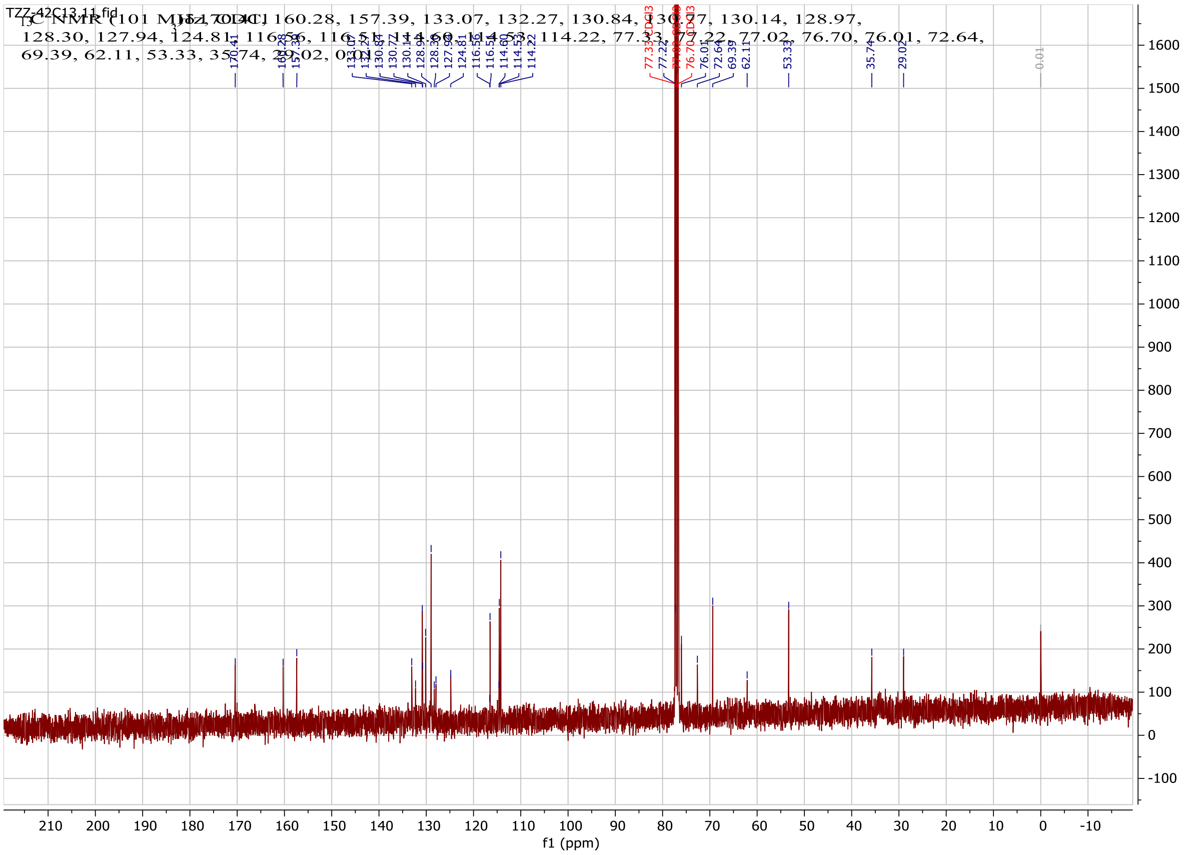

Figure S77. ^{13}C spectrum of compound **97**

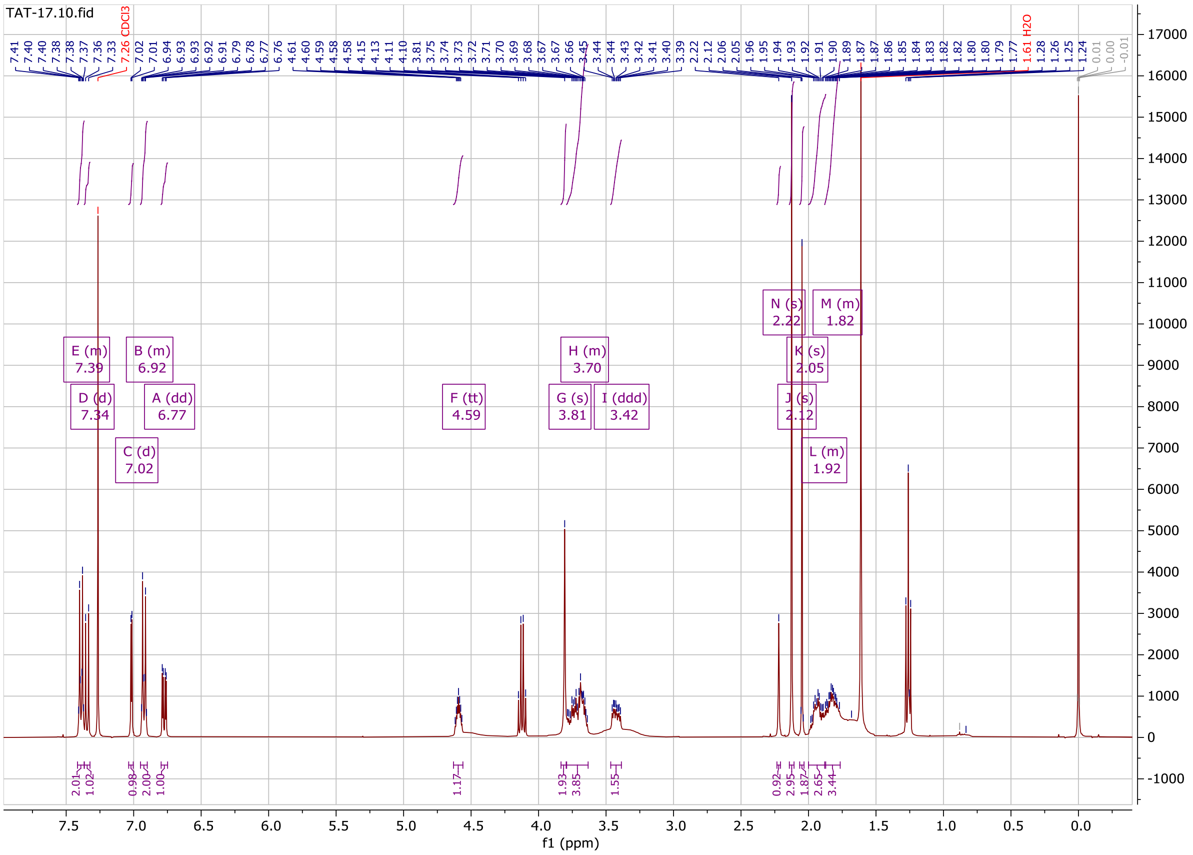

Figure S78. ^{1}H spectrum of compound **103**

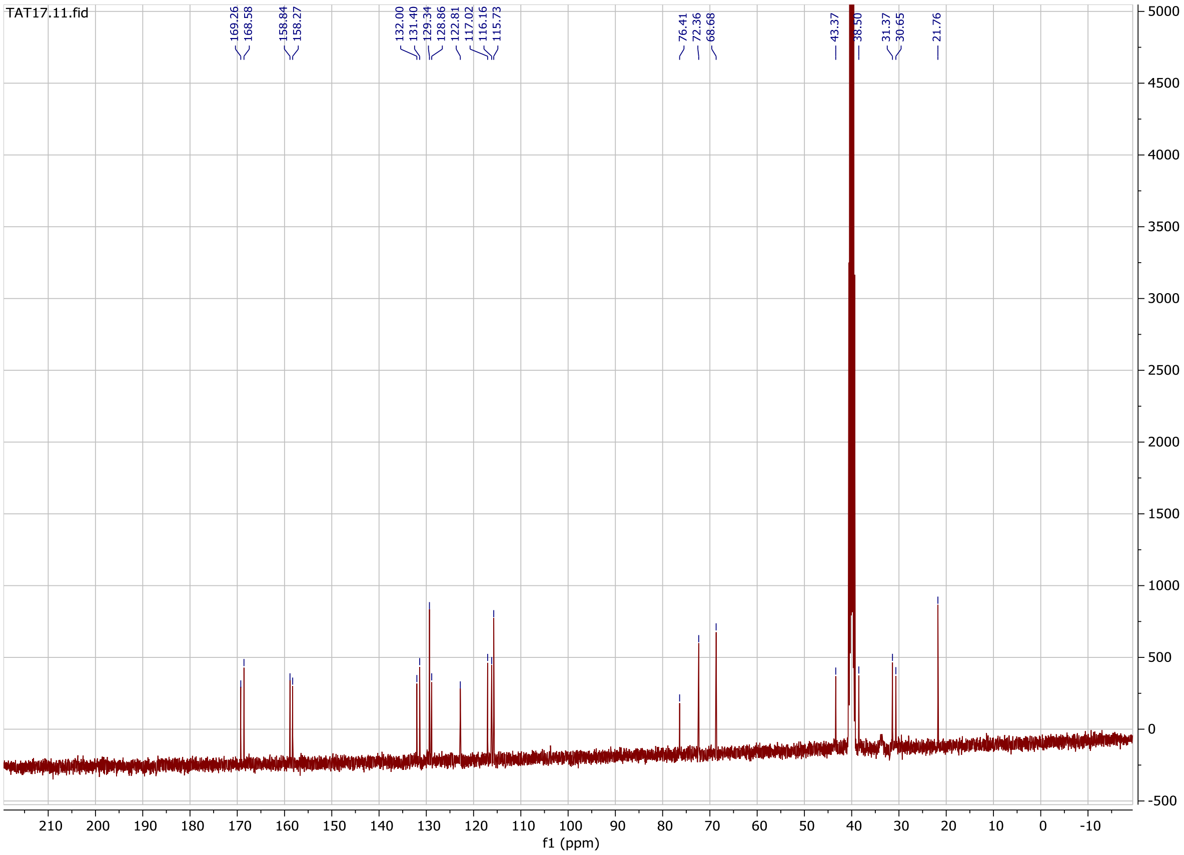

Figure S79. ^{13}C spectrum of compound **103**

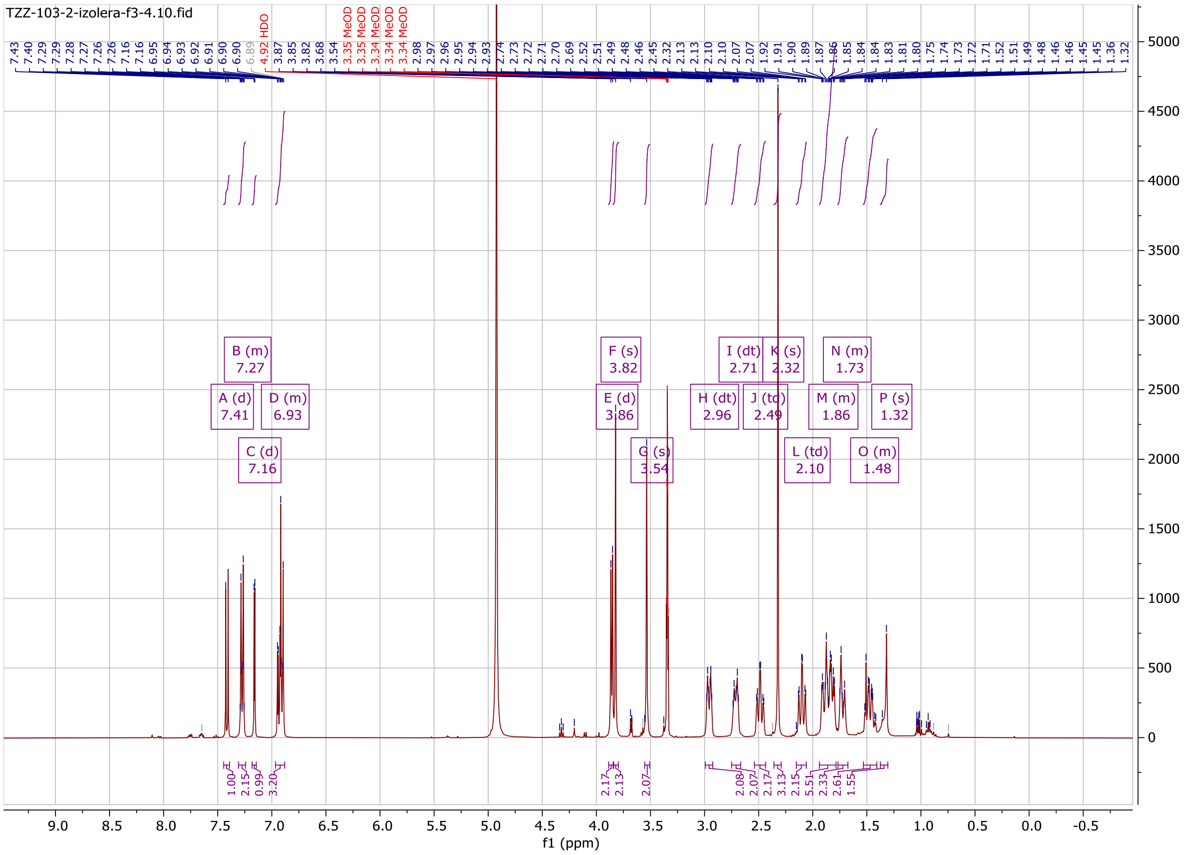

Figure S80. ^{1}H spectrum of compound **104**

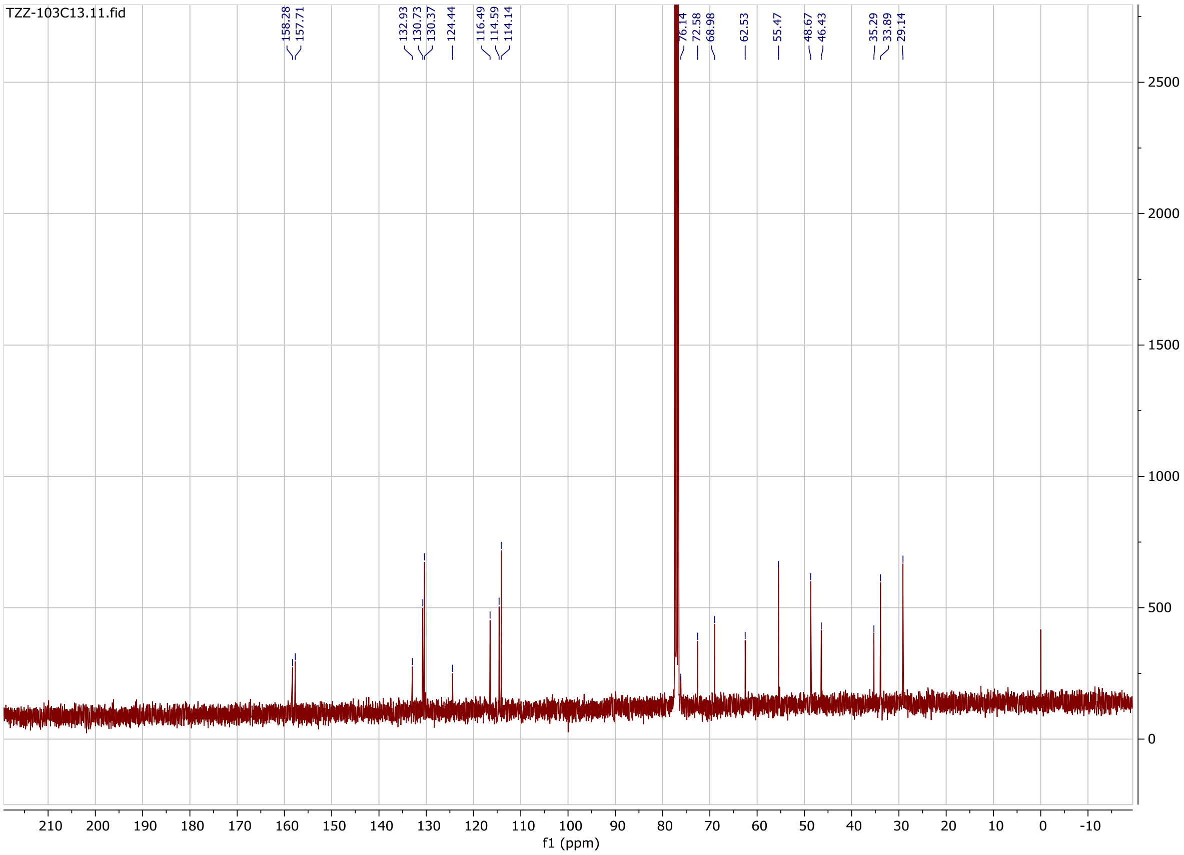

Figure S81. ^{13}Cspectrum of compound **104**

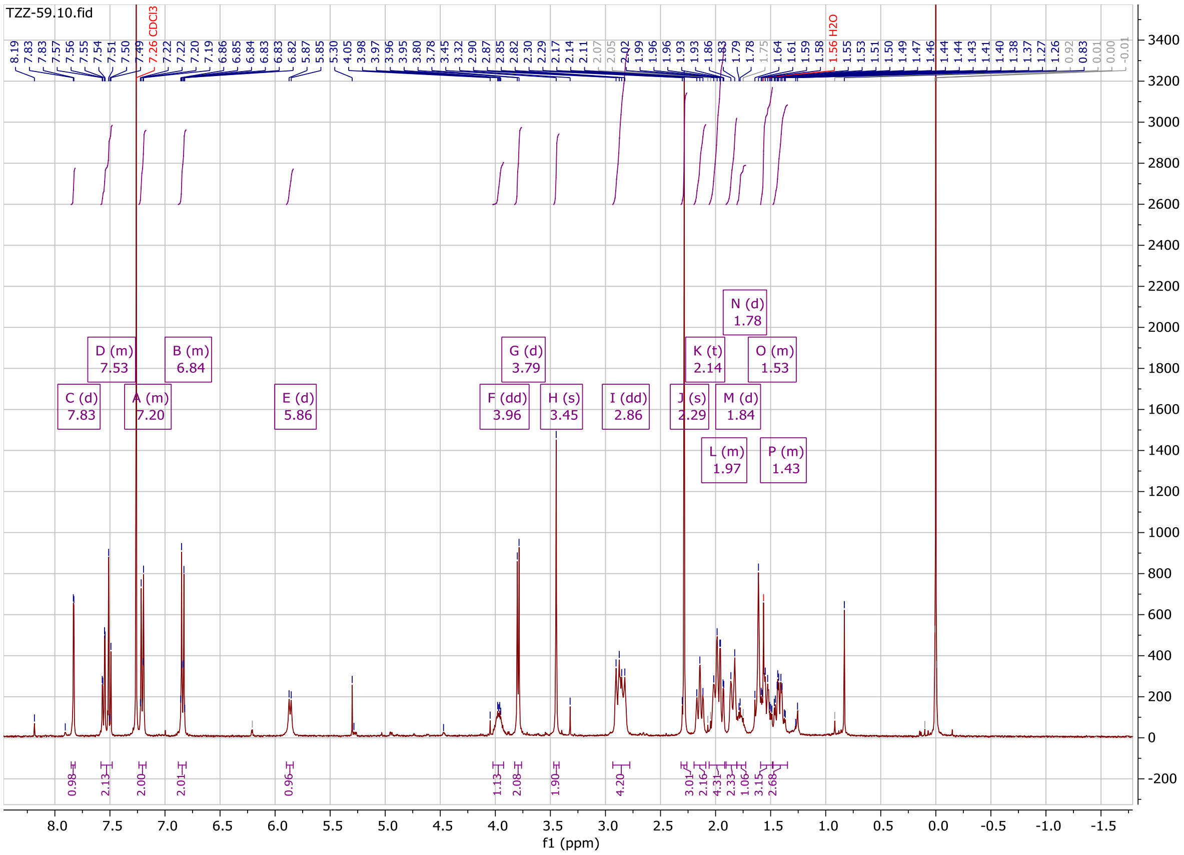

Figure S82. ^{1}H spectrum of compound **105**

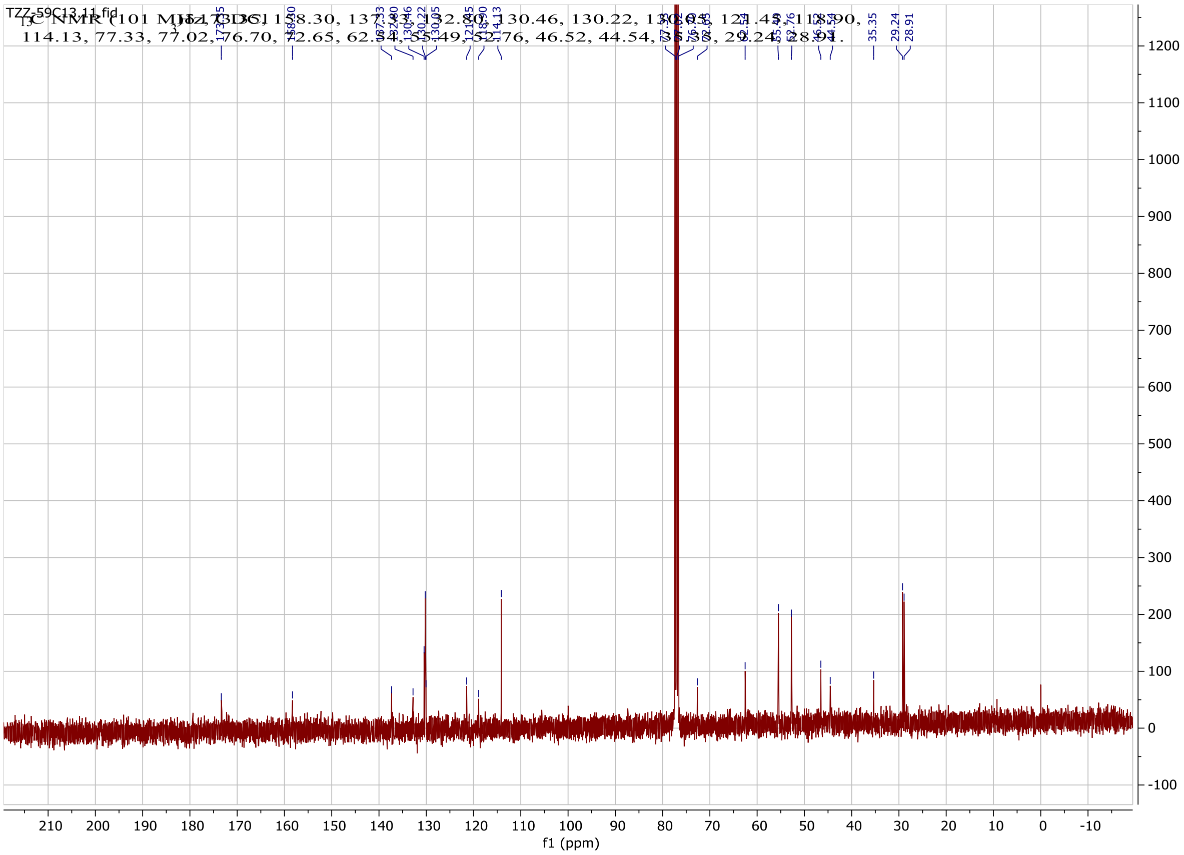

Figure S83. ^{13}Cspectrum of compound **105**

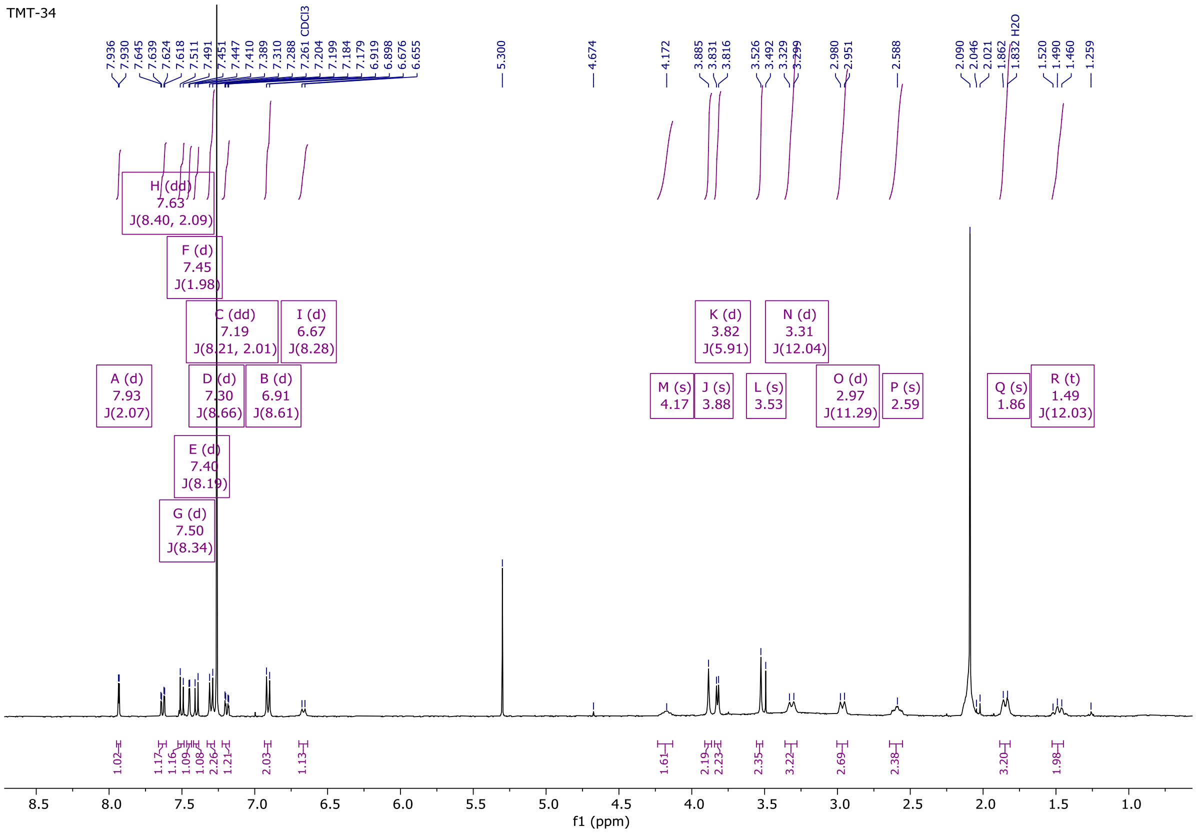

Figure S84. ^{1}H spectrum of compound **106**

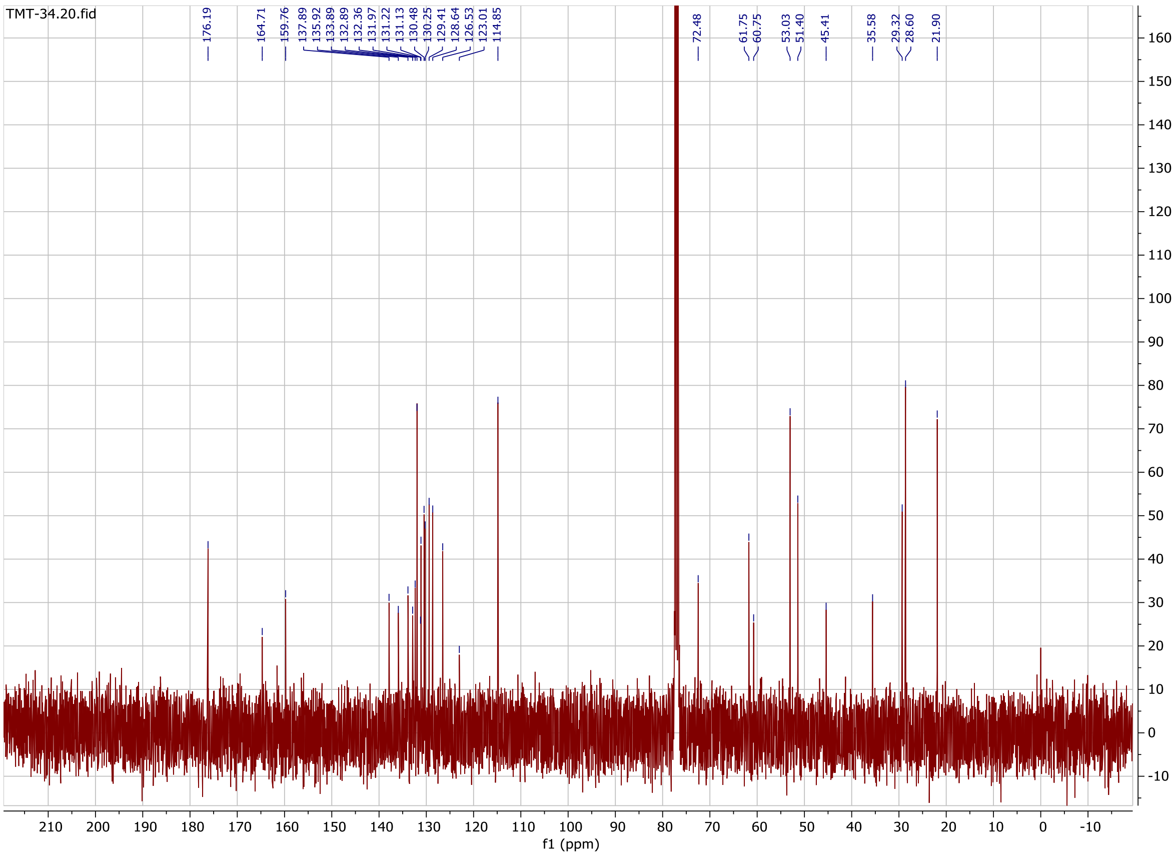

Figure S85. ^{13}C spectrum of compound **106**

1.9 Representative HPLC and UPLC chromatograms

Instrument:ULTIMATE3000 Sequence:TVS21_19.12.22 Page 5 of 54

Chromatogram and Results

Injection Details

Injection Name:	**TNT17**	*Run Time (min):*	**8,00**
Vial Number:	**BA2**	*Injection Volume:*	**2,00**
Injection Type:	**Unknown**	*Channel:*	**UV_VIS_3**
Calibration Level:		*Wavelength:*	**254**
Instrument Method:	**Purity_PROTAC_short**	*Bandwidth:*	**16**
Processing Method:	**Quantitative**	*Dilution Factor:*	**1,0000**
Injection Date/Time:	**19.dec..22 10:58**	*Sample Weight:*	**1,0000**

Chromatogram

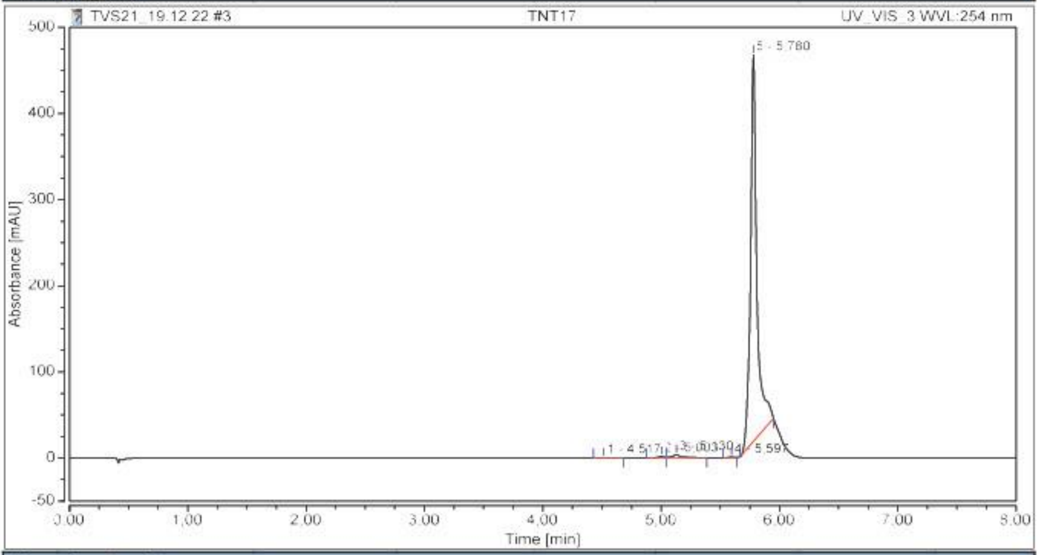

Integration Results

No.	Peak Name	Retention Time min	Area mAU*min	Height mAU	Relative Area %	Relative Height %	Amount n.a.
1		4,517	0,030	0,357	0,10	0,08	n.a.
2		5,003	0,106	1,747	0,37	0,38	n.a.
3		5,130	0,360	3,887	1,25	0,85	n.a.
4		5,597	0,032	0,678	0,11	0,15	n.a.
5		5,780	28,381	448,747	98,17	98,54	n.a.
Total:			28,910	455,416	100,00	100,00	

TVS21_19.12.22/Integration

Chromeleon (c) Dionex
Version 7.2.9.11323

Figure S86. UPLC chromatogram of compound **45**

Chromatogram and Results

Injection Details

Injection Name:	**TNT18**	*Run Time (min):*	**8,00**
Vial Number:	**BA3**	*Injection Volume:*	**2,00**
Injection Type:	**Unknown**	*Channel:*	**UV_VIS_3**
Calibration Level:		*Wavelength:*	**254**
Instrument Method:	**Purity_PROTAC_short**	*Bandwidth:*	**16**
Processing Method:	**Quantitative**	*Dilution Factor:*	**1,0000**
Injection Date/Time:	**19.dec..22 11:10**	*Sample Weight:*	**1,0000**

Chromatogram

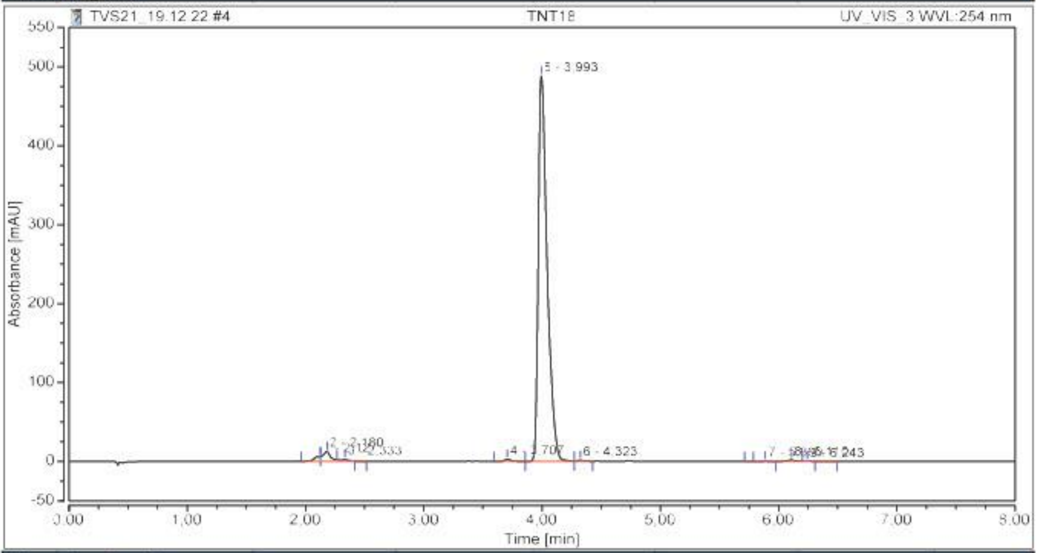

Integration Results

No.	Peak Name	Retention Time min	Area mAU*min	Height mAU	Relative Area %	Relative Height %	Amount n.a.
1		2,127	0,351	6,038	0,79	1,17	n.a.
2		2,180	1,139	12,743	2,56	2,47	n.a.
3		2,333	0,056	1,011	0,12	0,20	n.a.
4		3,707	0,174	2,926	0,39	0,57	n.a.
5		3,993	42,444	488,801	95,49	94,83	n.a.
6		4,323	0,094	1,578	0,21	0,31	n.a.
7		5,890	0,016	0,332	0,04	0,06	n.a.
8		6,110	0,172	1,957	0,39	0,38	n.a.
9		6,243	0,002	0,041	0,00	0,01	n.a.
Total:			44,447	515,428	100,00	100,00	

Chromeleon (c) Dionex Version 7.2.9.11323

Figure S87. UPLC chromatogram of compound **46**

Chromatogram and Results

Injection Details

Injection Name:	**TPZ8**	*Run Time (min):*	**8,00**
Vial Number:	**BA4**	*Injection Volume:*	**2,00**
Injection Type:	**Unknown**	*Channel:*	**UV_VIS_3**
Calibration Level:		*Wavelength:*	**254**
Instrument Method:	**Purity_PROTAC_short**	*Bandwidth:*	**16**
Processing Method:	**Quantitative**	*Dilution Factor:*	**1,0000**
Injection Date/Time:	**19.dec..22 11:22**	*Sample Weight:*	**1,0000**

Chromatogram

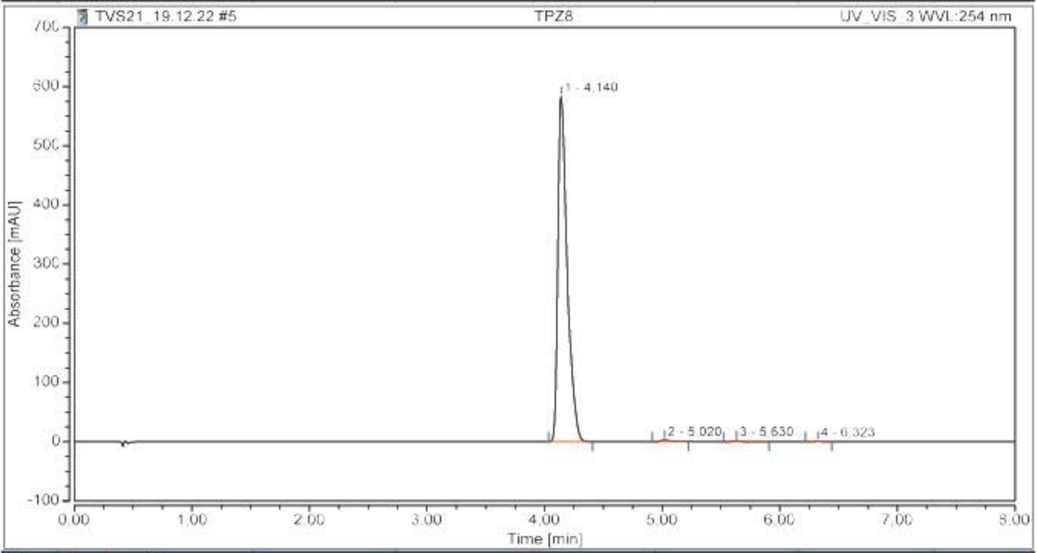

Integration Results

No.	Peak Name	Retention Time min	Area mAU*min	Height mAU	Relative Area %	Relative Height %	Amount n.a.
1		4,140	53,473	583,658	99,23	99,14	n.a.
2		5,020	0,245	2,428	0,45	0,41	n.a.
3		5,630	0,128	1,823	0,24	0,31	n.a.
4		6,323	0,040	0,821	0,07	0,14	n.a.
**Total:**			**53,886**	**588,730**	**100,00**	**100,00**	

Chromeleon (c) Dionex
Version 7.2.9.11323

Figure S88. UPLC chromatogram of compound **47**

Chromatogram and Results

Injection Details

Injection Name:	**TPZ23**	*Run Time (min):*	**8,00**
Vial Number:	**BA5**	*Injection Volume:*	**2,00**
Injection Type:	**Unknown**	*Channel:*	**UV_VIS_3**
Calibration Level:		*Wavelength:*	**254**
Instrument Method:	**Purity_PROTAC_short**	*Bandwidth:*	**16**
Processing Method:	**Quantitative**	*Dilution Factor:*	**1,0000**
Injection Date/Time:	**19.dec..22 11:34**	*Sample Weight:*	**1,0000**

Chromatogram

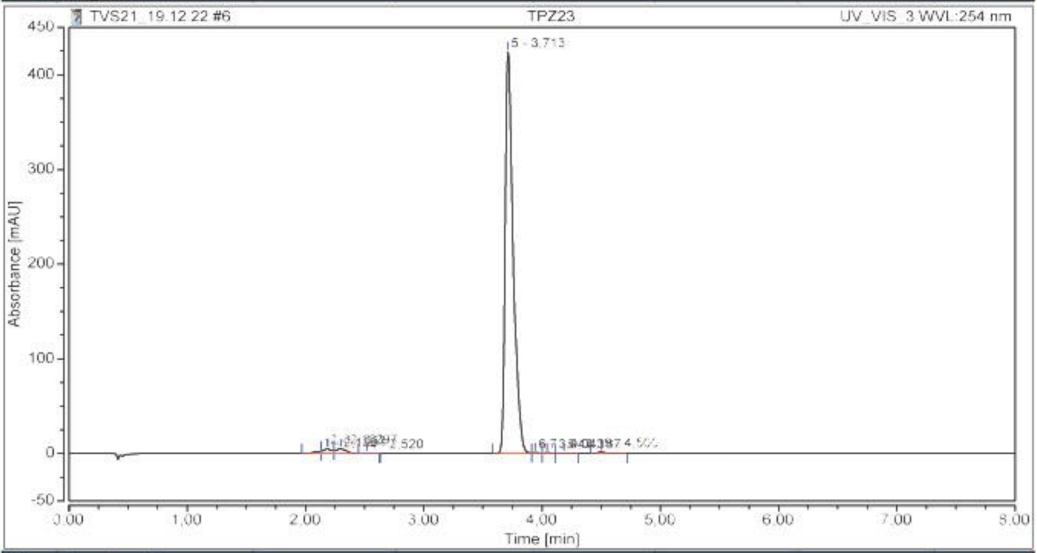

Integration Results

No.	Peak Name	Retention Time min	Area mAU*min	Height mAU	Relative Area %	Relative Height %	Amount n.a.
1		2,130	0,147	2,216	0,43	0,50	n.a.
2		2,183	0,387	5,024	1,12	1,14	n.a.
3		2,297	0,495	4,995	1,44	1,13	n.a.
4		2,520	0,046	0,663	0,13	0,15	n.a.
5		3,713	33,137	424,352	96,11	96,14	n.a.
6		3,943	0,057	0,945	0,17	0,21	n.a.
7		4,043	0,053	0,881	0,15	0,20	n.a.
8		4,187	0,046	0,628	0,13	0,14	n.a.
9		4,500	0,109	1,706	0,32	0,39	n.a.
Total:			34,478	441,409	100,00	100,00	

TVS21_19.12.22/Integration

Chromeleon (c) Dionex
Version 7.2.9.11323

Figure S89. UPLC chromatogram of compound **48**

Chromatogram and Results

Injection Details

Injection Name:	TPZ15	Run Time (min):	8,00
Vial Number:	BA6	Injection Volume:	2,00
Injection Type:	Unknown	Channel:	UV_VIS_3
Calibration Level:		Wavelength:	254
Instrument Method:	Purity_PROTAC_short	Bandwidth:	16
Processing Method:	Quantitative	Dilution Factor:	1,0000
Injection Date/Time:	19.dec..22 11:46	Sample Weight:	1,0000

Chromatogram

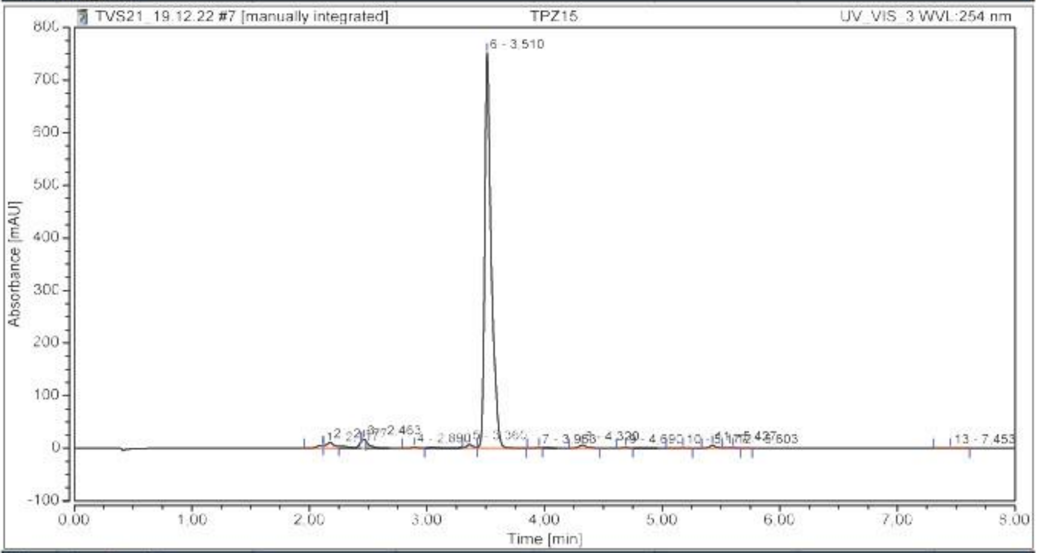

Integration Results

No.	Peak Name	Retention Time min	Area mAU*min	Height mAU	Relative Area %	Relative Height %	Amount n.a.
1		2,117	0,292	5,052	0,55	0,62	n.a.
2		2,177	0,886	11,165	1,68	1,38	n.a.
3		2,463	0,627	17,013	1,19	2,10	n.a.
4		2,890	0,123	1,928	0,23	0,24	n.a.
5		3,360	0,447	7,265	0,85	0,90	n.a.
6		3,510	49,196	751,976	93,45	92,86	n.a.
7		3,953	0,042	0,683	0,08	0,08	n.a.
8		4,320	0,425	5,896	0,81	0,73	n.a.
9		4,690	0,070	1,138	0,13	0,14	n.a.
10		5,177	0,039	0,656	0,07	0,08	n.a.
11		5,427	0,425	5,928	0,81	0,73	n.a.
12		5,603	0,037	0,633	0,07	0,08	n.a.
13		7,453	0,035	0,499	0,07	0,06	n.a.
Total:			52,644	809,830	100,00	100,00	

Figure S90. UPLC chromatogram of compound **49**

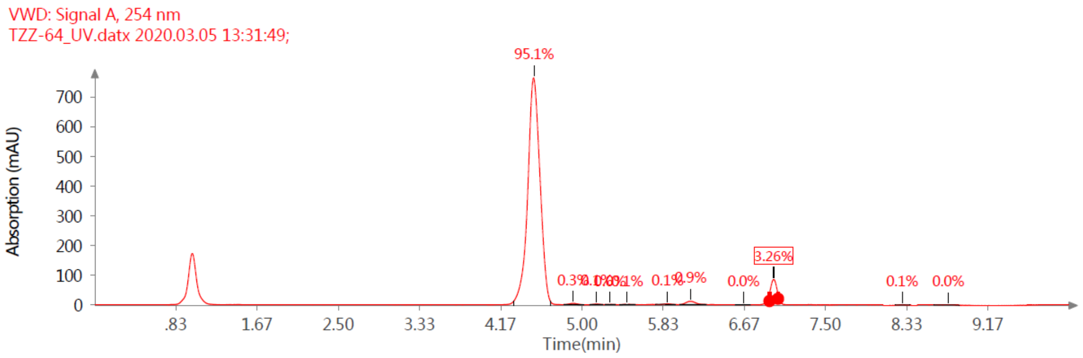

Figure S91. HPLC chromatogram of compound **50**

Chromatogram and Results

Injection Details

Injection Name:	**TAT14**	*Run Time (min):*	**8,00**
Vial Number:	**BA7**	*Injection Volume:*	**2,00**
Injection Type:	**Unknown**	*Channel:*	**UV_VIS_3**
Calibration Level:		*Wavelength:*	**254**
Instrument Method:	**Purity_PROTAC_short**	*Bandwidth:*	**16**
Processing Method:	**Quantitative**	*Dilution Factor:*	**1,0000**
Injection Date/Time:	**19.dec..22 11:58**	*Sample Weight:*	**1,0000**

Chromatogram

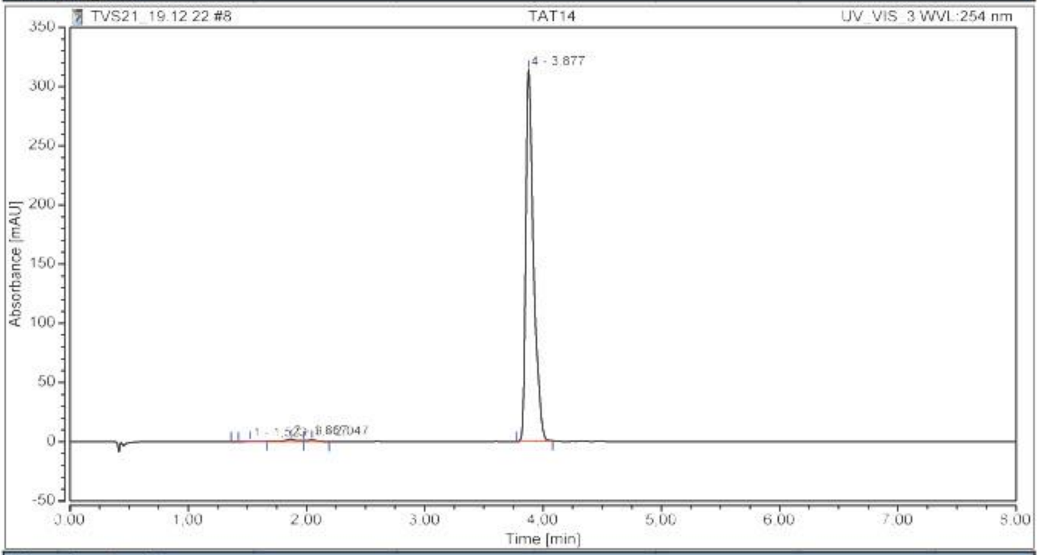

Integration Results

No.	Peak Name	Retention Time min	Area mAU*min	Height mAU	Relative Area %	Relative Height %	Amount n.a.
1		1,523	0,021	0,184	0,09	0,06	n.a.
2		1,867	0,328	2,152	1,35	0,68	n.a.
3		2,047	0,141	1,431	0,58	0,45	n.a.
4		3,877	23,924	314,114	97,99	98,81	n.a.
**Total:**			**24,414**	**317,881**	**100,00**	**100,00**	

TVS21_19.12.22/Integration

Chromeleon (c) Dionex
Version 7.2.9.11323

Figure S92. UPLC chromatogram of compound **51**

Chromatogram and Results

Injection Details

Injection Name:	**TPZ28**	*Run Time (min):*	**8,00**
Vial Number:	**BA8**	*Injection Volume:*	**2,00**
Injection Type:	**Unknown**	*Channel:*	**UV_VIS_3**
Calibration Level:		*Wavelength:*	**254**
Instrument Method:	**Purity_PROTAC_short**	*Bandwidth:*	**16**
Processing Method:	**Quantitative**	*Dilution Factor:*	**1,0000**
Injection Date/Time:	**19.dec..22 12:09**	*Sample Weight:*	**1,0000**

Chromatogram

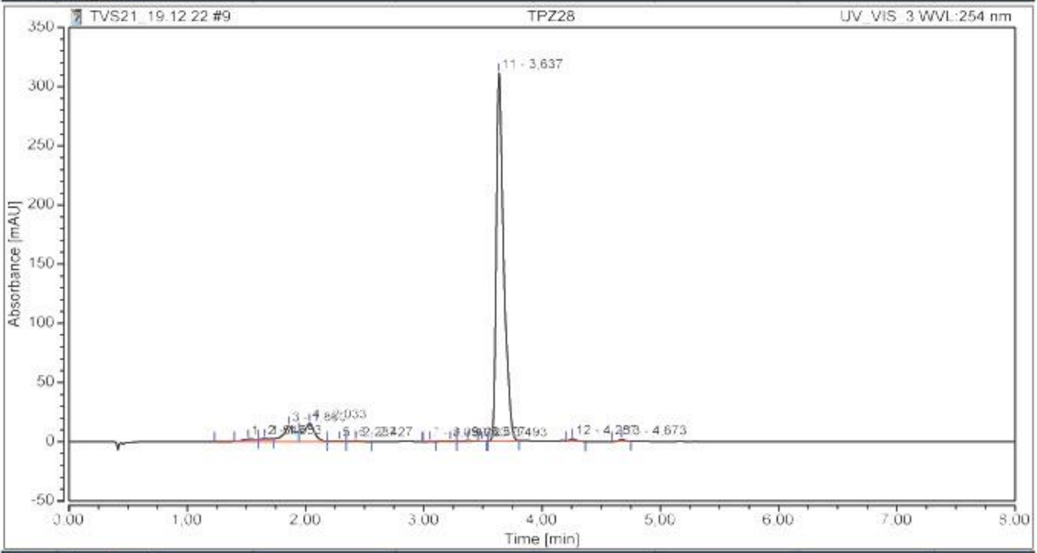

Integration Results

No.	Peak Name	Retention Time min	Area mAU*min	Height mAU	Relative Area %	Relative Height %	Amount n.a.
1		1,517	0,128	1,143	0,48	0,33	n.a.
2		1,653	0,041	0,596	0,15	0,17	n.a.
3		1,860	1,967	13,131	7,35	3,77	n.a.
4		2,033	1,666	15,590	6,22	4,48	n.a.
5		2,287	0,058	0,623	0,22	0,18	n.a.
6		2,427	0,063	0,740	0,24	0,21	n.a.
7		3,050	0,006	0,125	0,02	0,04	n.a.
8		3,223	0,042	0,404	0,16	0,12	n.a.
9		3,377	0,077	0,972	0,29	0,28	n.a.
10		3,493	0,003	0,079	0,01	0,02	n.a.
11		3,637	22,520	311,126	84,12	89,32	n.a.
12		4,257	0,113	2,142	0,42	0,62	n.a.
13		4,673	0,086	1,651	0,32	0,47	n.a.
**Total:**			**26,770**	**348,323**	**100,00**	**100,00**	

Chromeleon (c) Dionex
Version 7.2.9.11323

Figure S93. UPLC chromatogram of compound **52**

Chromatogram and Results

Injection Details

Injection Name:	**TAA-10**	*Run Time (min):*	**8,00**
Vial Number:	**BA2**	*Injection Volume:*	**5,00**
Injection Type:	**Unknown**	*Channel:*	**UV_VIS_3**
Calibration Level:		*Wavelength:*	**254**
Instrument Method:	**Purity_PROTAC_short**	*Bandwidth:*	**16**
Processing Method:	**Quantitative**	*Dilution Factor:*	**1,0000**
Injection Date/Time:	**14.apr..22 08:14**	*Sample Weight:*	**1,0000**

Chromatogram

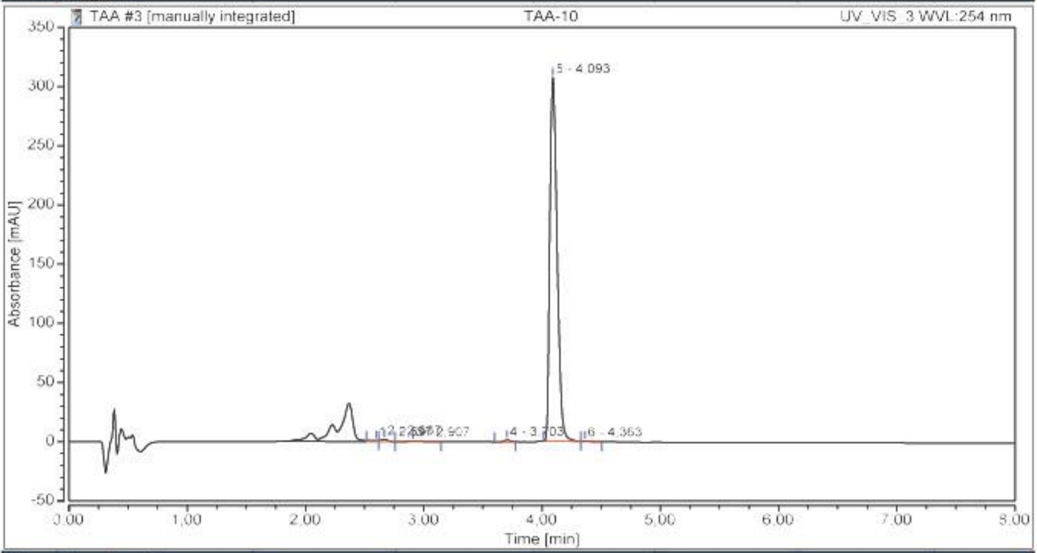

Integration Results

No.	Peak Name	Retention Time min	Area mAU*min	Height mAU	Relative Area %	Relative Height %	Amount n.a.
1		2,597	0,106	1,139	0,47	0,36	n.a.
2		2,667	0,134	1,964	0,60	0,63	n.a.
3		2,907	0,102	0,752	0,45	0,24	n.a.
4		3,703	0,087	1,573	0,39	0,50	n.a.
5		4,093	21,980	307,482	97,93	98,11	n.a.
6		4,363	0,036	0,508	0,16	0,16	n.a.
**Total:**			**22,444**	**313,418**	**100,00**	**100,00**	

Figure S94. UPLC chromatogram of compound **53**

Chromatogram and Results

Injection Details

Injection Name:	TAA-11	*Run Time (min):*	8,00
Vial Number:	BA3	*Injection Volume:*	5,00
Injection Type:	Unknown	*Channel:*	UV_VIS_3
Calibration Level:		*Wavelength:*	254
Instrument Method:	Purity_PROTAC_short	*Bandwidth:*	16
Processing Method:	Quantitative	*Dilution Factor:*	1,0000
Injection Date/Time:	14.apr..22 08:26	*Sample Weight:*	1,0000

Chromatogram

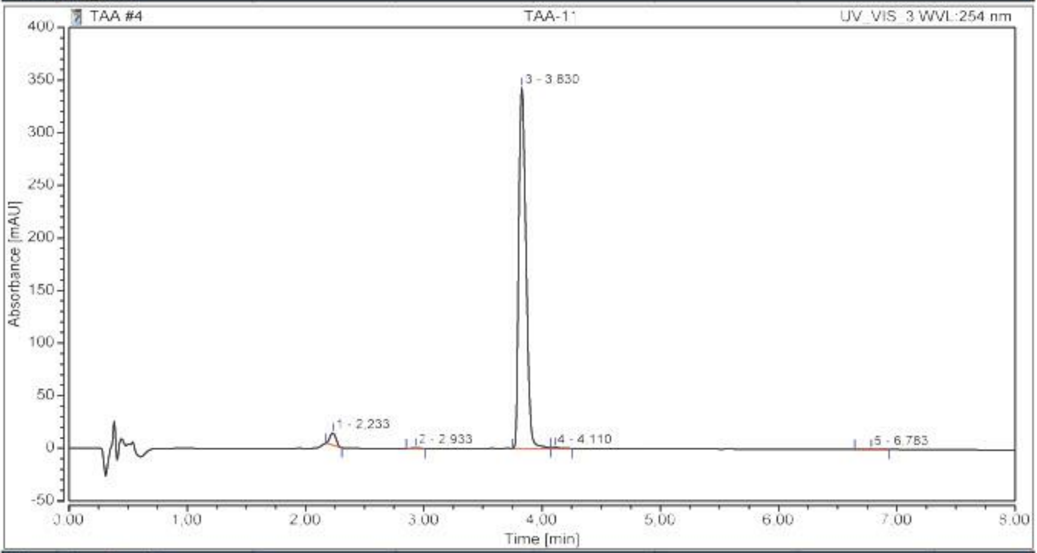

Integration Results

No.	Peak Name	Retention Time min	Area mAU*min	Height mAU	Relative Area %	Relative Height %	Amount n.a.
1		2,233	0,703	11,758	2,81	3,30	n.a.
2		2,933	0,032	0,456	0,13	0,13	n.a.
3		3,830	24,205	342,951	96,65	96,28	n.a.
4		4,110	0,074	0,794	0,30	0,22	n.a.
5		6,783	0,029	0,244	0,12	0,07	n.a.
Total:			25,043	356,201	100,00	100,00	

TAA/Integration

Chromeleon (c) Dionex
Version 7.2.9.11323

Figure S95. UPLC chromatogram of compound **54**

Chromatogram and Results

Injection Details

Injection Name:	**TAA-16**	*Run Time (min):*	**8,00**
Vial Number:	**BA4**	*Injection Volume:*	**5,00**
Injection Type:	**Unknown**	*Channel:*	**UV_VIS_3**
Calibration Level:		*Wavelength:*	**254**
Instrument Method:	**Purity_PROTAC_short**	*Bandwidth:*	**16**
Processing Method:	**Quantitative**	*Dilution Factor:*	**1,0000**
Injection Date/Time:	**14.apr..22 08:38**	*Sample Weight:*	**1,0000**

Chromatogram

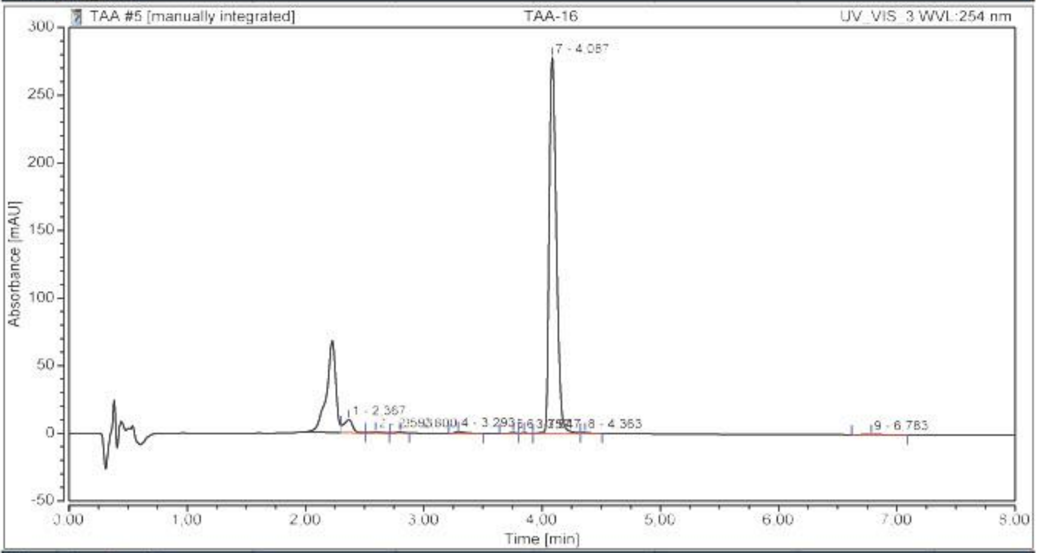

Integration Results

No.	Peak Name	Retention Time min	Area mAU*min	Height mAU	Relative Area %	Relative Height %	Amount n.a.
1		2,367	0,810	9,544	3,86	3,26	n.a.
2		2,593	0,062	0,588	0,30	0,20	n.a.
3		2,800	0,064	0,788	0,30	0,27	n.a.
4		3,293	0,137	1,508	0,65	0,51	n.a.
5		3,757	0,053	0,764	0,25	0,26	n.a.
6		3,847	0,042	0,692	0,20	0,24	n.a.
7		4,087	19,722	278,169	93,93	94,92	n.a.
8		4,363	0,059	0,736	0,28	0,25	n.a.
9		6,783	0,048	0,267	0,23	0,09	n.a.
Total:			20,996	293,056	100,00	100,00	

TAA/Integration

Chromeleon (c) Dionex
Version 7.2.9.11323

Figure S96. UPLC chromatogram of compound **55**

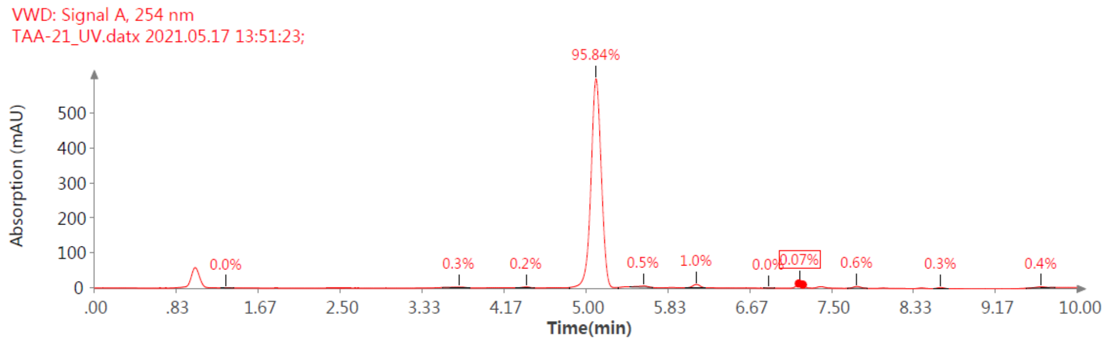

Figure S97. HPLC chromatogram of compound **56**

Chromatogram and Results

Injection Details

Injection Name:	**TZZ10**	*Run Time (min):*	**8,00**
Vial Number:	**BB1**	*Injection Volume:*	**2,00**
Injection Type:	**Unknown**	*Channel:*	**UV_VIS_3**
Calibration Level:		*Wavelength:*	**254**
Instrument Method:	**Purity_PROTAC_short**	*Bandwidth:*	**16**
Processing Method:	**Quantitative**	*Dilution Factor:*	**1,0000**
Injection Date/Time:	**19.dec..22 12:21**	*Sample Weight:*	**1,0000**

Chromatogram

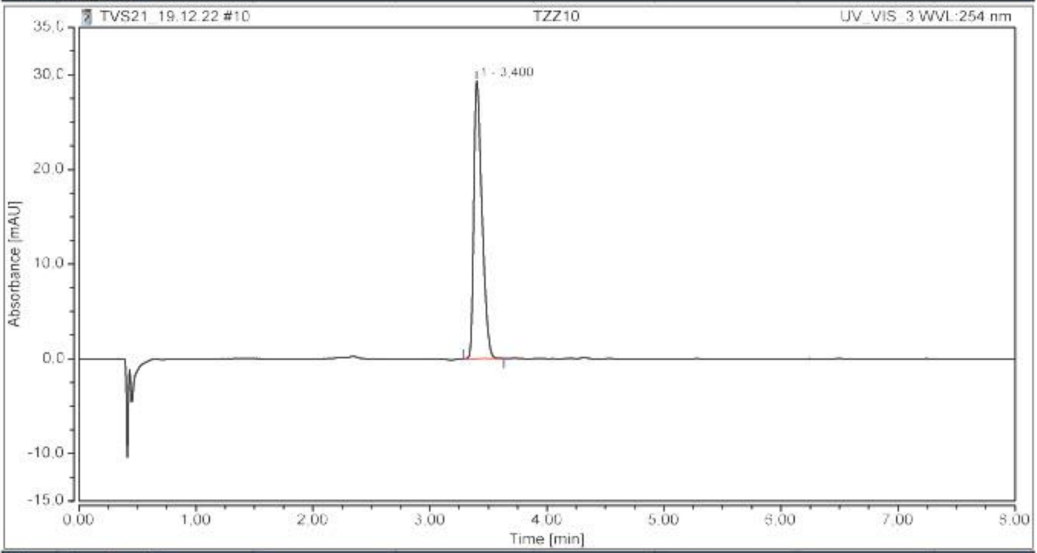

Integration Results

No.	Peak Name	Retention Time min	Area mAU*min	Height mAU	Relative Area %	Relative Height %	Amount n.a.
1		3,400	2,380	29,329	100,00	100,00	n.a.
**Total:**			**2,380**	**29,329**	**100,00**	**100,00**	

Figure S98. UPLC chromatogram of compound **57**

Chromatogram and Results

Injection Details

Injection Name:	**TNT14**	*Run Time (min):*	**8,00**
Vial Number:	**BD2**	*Injection Volume:*	**2,00**
Injection Type:	**Unknown**	*Channel:*	**UV_VIS_3**
Calibration Level:		*Wavelength:*	**254**
Instrument Method:	**Purity_PROTAC_short**	*Bandwidth:*	**16**
Processing Method:	**Quantitative**	*Dilution Factor:*	**1,0000**
Injection Date/Time:	**19.dec..22 15:44**	*Sample Weight:*	**1,0000**

Chromatogram

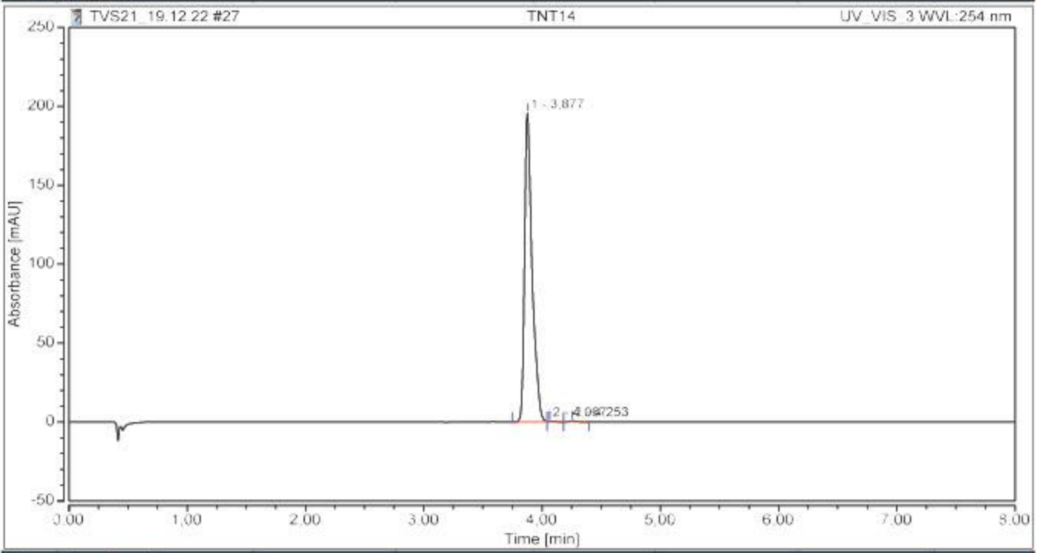

Integration Results

No.	Peak Name	Retention Time min	Area mAU*min	Height mAU	Relative Area %	Relative Height %	Amount n.a.
1		3,877	14,485	195,755	99,42	99,32	n.a.
2		4,067	0,036	0,615	0,25	0,31	n.a.
3		4,253	0,049	0,717	0,33	0,36	n.a.
**Total:**			**14,569**	**197,087**	**100,00**	**100,00**	

Figure S99. UPLC chromatogram of compound **58**

Chromatogram and Results

Injection Details

Injection Name:	**TNT7**	*Run Time (min):*	**8,00**
Vial Number:	**BB2**	*Injection Volume:*	**2,00**
Injection Type:	**Unknown**	*Channel:*	**UV_VIS_3**
Calibration Level:		*Wavelength:*	**254**
Instrument Method:	**Purity_PROTAC_short**	*Bandwidth:*	**16**
Processing Method:	**Quantitative**	*Dilution Factor:*	**1,0000**
Injection Date/Time:	**19.dec..22 12:33**	*Sample Weight:*	**1,0000**

Chromatogram

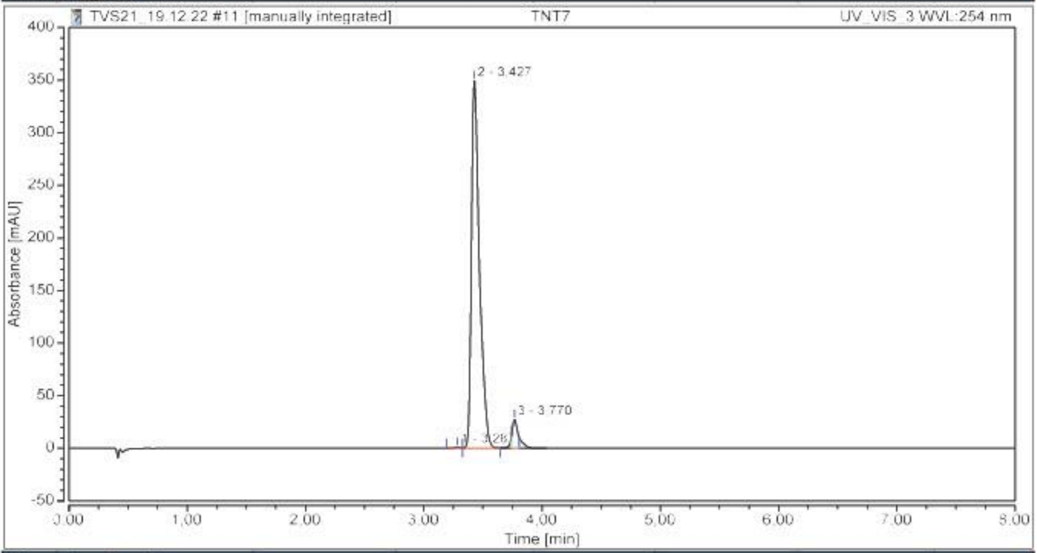

Integration Results

No.	Peak Name	Retention Time min	Area mAU*min	Height mAU	Relative Area %	Relative Height %	Amount n.a.
1		3,287	0,046	0,738	0,16	0,20	n.a.
2		3,427	27,465	349,591	95,42	92,49	n.a.
3		3,770	1,274	27,628	4,43	7,31	n.a.
**Total:**			**28,784**	**377,957**	**100,00**	**100,00**	

Chromeleon (c) Dionex
Version 7.2.9.11323

Figure S100. UPLC chromatogram of compound **59**

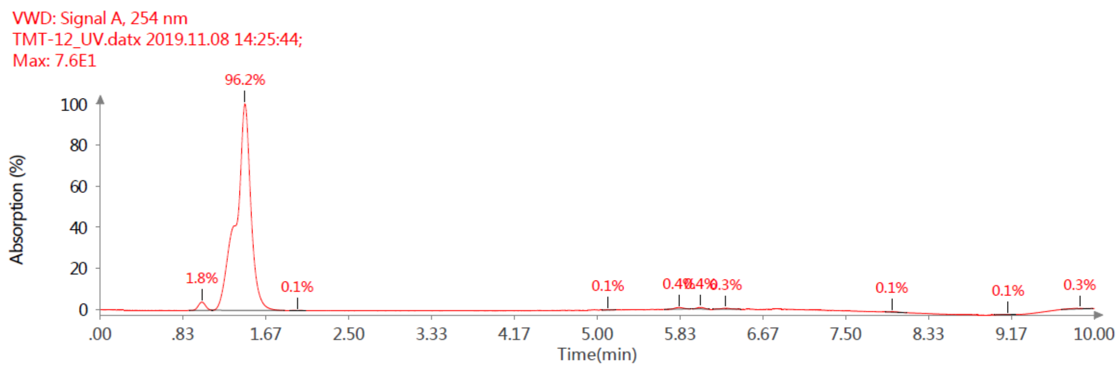

Figure S101. HPLC chromatogram of compound **66**

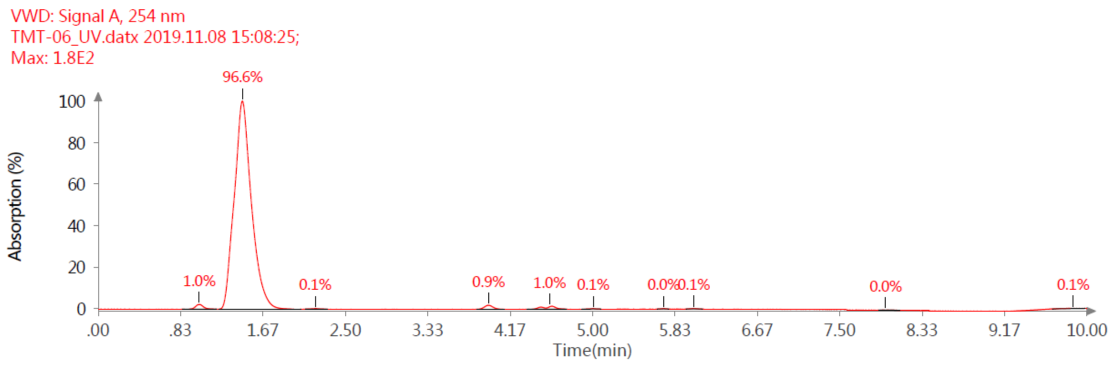

Figure S102. HPLC chromatogram of compound **67**

Chromatogram and Results

Injection Details

Injection Name:	TAT24	Run Time (min):	8,00
Vial Number:	BB3	Injection Volume:	2,00
Injection Type:	Unknown	Channel:	UV_VIS_3
Calibration Level:		Wavelength:	254
Instrument Method:	Purity_PROTAC_short	Bandwidth:	16
Processing Method:	Quantitative	Dilution Factor:	1,0000
Injection Date/Time:	19.dec..22 12:45	Sample Weight:	1,0000

Chromatogram

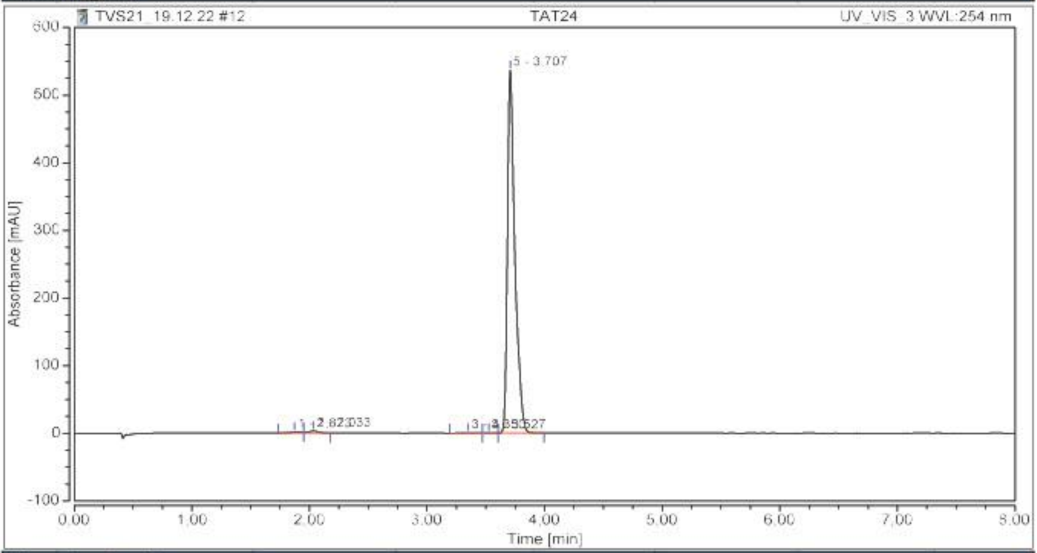

Integration Results

No.	Peak Name	Retention Time min	Area mAU*min	Height mAU	Relative Area %	Relative Height %	Amount n.a.
1		1,873	0,195	1,824	0,49	0,34	n.a.
2		2,033	0,332	3,249	0,83	0,60	n.a.
3		3,350	0,066	0,546	0,16	0,10	n.a.
4		3,527	0,030	0,368	0,08	0,07	n.a.
5		3,707	39,529	536,642	98,45	98,90	n.a.
Total:			40,152	542,628	100,00	100,00	

Chromeleon (c) Dionex
Version 7.2.9.11323

Figure S103. UPLC chromatogram of compound **68**

Chromatogram and Results

Injection Details

Injection Name:	TAT23	*Run Time (min):*	8,00
Vial Number:	BB4	*Injection Volume:*	2,00
Injection Type:	Unknown	*Channel:*	UV_VIS_3
Calibration Level:		*Wavelength:*	254
Instrument Method:	Purity_PROTAC_short	*Bandwidth:*	16
Processing Method:	Quantitative	*Dilution Factor:*	1,0000
Injection Date/Time:	19.dec..22 12:57	*Sample Weight:*	1,0000

Chromatogram

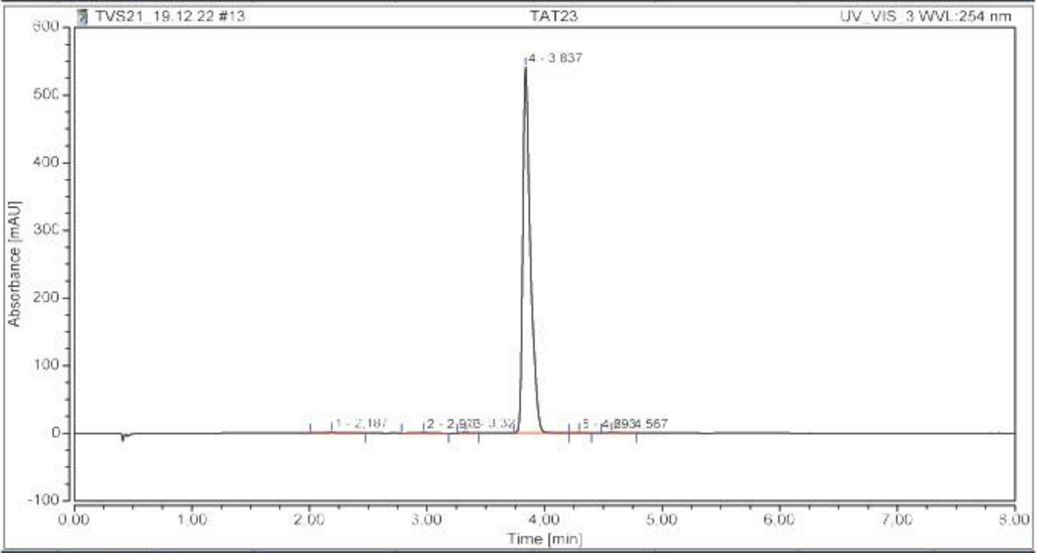

Integration Results

No.	Peak Name	Retention Time min	Area mAU*min	Height mAU	Relative Area %	Relative Height %	Amount n.a.
1		2,187	0,156	1,340	0,38	0,25	n.a.
2		2,973	0,090	0,988	0,22	0,18	n.a.
3		3,327	0,073	1,235	0,18	0,23	n.a.
4		3,837	40,640	541,431	98,86	99,03	n.a.
5		4,293	0,040	0,646	0,10	0,12	n.a.
6		4,567	0,110	1,076	0,27	0,20	n.a.
**Total:**			**41,109**	**546,716**	**100,00**	**100,00**	

Chromeleon (c) Dionex
Version 7.2.9.11323

Figure S104. UPLC chromatogram of compound **69**

Chromatogram and Results

Injection Details

Injection Name:	TZZ46	*Run Time (min):*	8,00
Vial Number:	BC8	*Injection Volume:*	2,00
Injection Type:	Unknown	*Channel:*	UV_VIS_3
Calibration Level:		*Wavelength:*	254
Instrument Method:	Purity_PROTAC_short	*Bandwidth:*	16
Processing Method:	Quantitative	*Dilution Factor:*	1,0000
Injection Date/Time:	19.dec..22 15:20	*Sample Weight:*	1,0000

Chromatogram

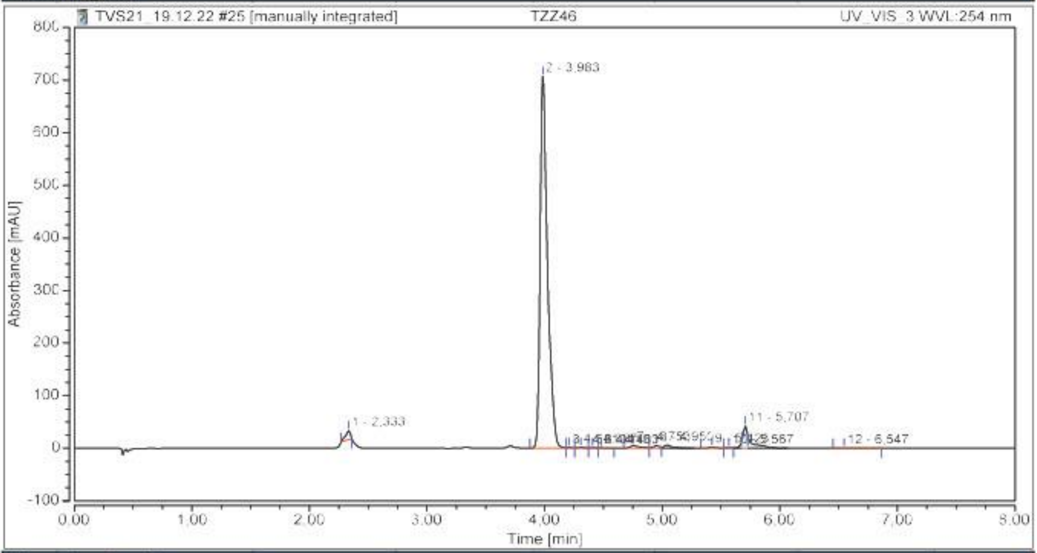

Integration Results

No.	Peak Name	Retention Time min	Area mAU*min	Height mAU	Relative Area %	Relative Height %	Amount n.a.
1		2,333	0,807	17,045	1,48	2,18	n.a.
2		3,983	51,057	706,892	93,49	90,27	n.a.
3		4,210	0,069	1,093	0,13	0,14	n.a.
4		4,307	0,106	1,450	0,19	0,19	n.a.
5		4,410	0,046	0,740	0,08	0,09	n.a.
6		4,483	0,030	0,542	0,06	0,07	n.a.
7		4,753	0,427	5,447	0,78	0,70	n.a.
8		4,950	0,275	4,677	0,50	0,60	n.a.
9		5,423	0,126	1,759	0,23	0,22	n.a.
10		5,567	0,053	0,844	0,10	0,11	n.a.
11		5,707	1,573	42,057	2,88	5,37	n.a.
12		6,547	0,045	0,545	0,08	0,07	n.a.
Total:			54,614	783,090	100,00	100,00	

Chromeleon (c) Dionex
Version 7.2.9.11323

Figure S105. UPLC chromatogram of compound **112**

Chromatogram and Results

Injection Details

Injection Name:	**TMT20**	*Run Time (min):*	**8,00**
Vial Number:	**BB5**	*Injection Volume:*	**2,00**
Injection Type:	**Unknown**	*Channel:*	**UV_VIS_3**
Calibration Level:		*Wavelength:*	**254**
Instrument Method:	**Purity_PROTAC_short**	*Bandwidth:*	**16**
Processing Method:	**Quantitative**	*Dilution Factor:*	**1,0000**
Injection Date/Time:	**19.dec..22 13:09**	*Sample Weight:*	**1,0000**

Chromatogram

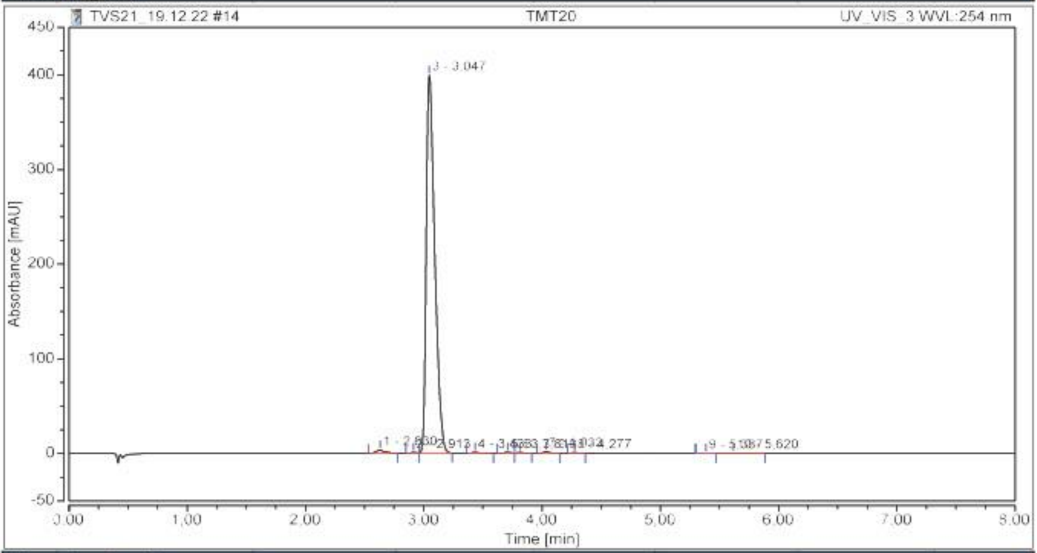

Integration Results

No.	Peak Name	Retention Time min	Area mAU*min	Height mAU	Relative Area %	Relative Height %	Amount n.a.
1		2,630	0,295	3,534	0,87	0,86	n.a.
2		2,913	0,051	1,070	0,15	0,26	n.a.
3		3,047	33,236	399,160	97,87	97,41	n.a.
4		3,433	0,063	1,124	0,19	0,27	n.a.
5		3,710	0,071	1,121	0,21	0,27	n.a.
6		3,813	0,046	0,830	0,13	0,20	n.a.
7		4,033	0,103	1,594	0,30	0,39	n.a.
8		4,277	0,037	0,725	0,11	0,18	n.a.
9		5,387	0,006	0,091	0,02	0,02	n.a.
10		5,620	0,051	0,538	0,15	0,13	n.a.
**Total:**			**33,958**	**409,788**	**100,00**	**100,00**	

TVS21_19.12.22/Integration

Chromeleon (c) Dionex
Version 7.2.9.11323

Figure S106. UPLC chromatogram of compound **88**

Chromatogram and Results

Injection Details

Injection Name:	**TMT11**	*Run Time (min):*	**8,00**
Vial Number:	**BC3**	*Injection Volume:*	**2,00**
Injection Type:	**Unknown**	*Channel:*	**UV_VIS_3**
Calibration Level:		*Wavelength:*	**254**
Instrument Method:	**Purity_PROTAC_short**	*Bandwidth:*	**16**
Processing Method:	**Quantitative**	*Dilution Factor:*	**1,0000**
Injection Date/Time:	**20.dec..22 14:11**	*Sample Weight:*	**1,0000**

Chromatogram

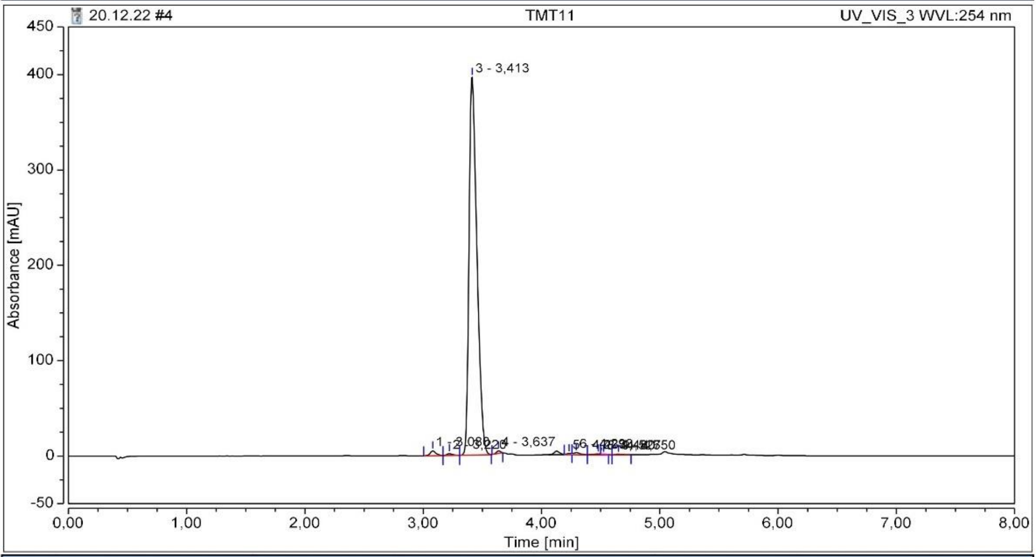

Integration Results

No.	Peak Name	Retention Time min	Area mAU*min	Height mAU	Relative Area %	Relative Height %	Amount n.a.
1		3,080	0,291	5,189	0,92	1,26	n.a.
2		3,220	0,110	2,021	0,35	0,49	n.a.
3		3,413	30,747	396,303	97,23	96,23	n.a.
4		3,637	0,106	2,809	0,34	0,68	n.a.
5		4,233	0,068	1,424	0,21	0,35	n.a.
6		4,293	0,155	2,357	0,49	0,57	n.a.
7		4,480	0,096	0,915	0,30	0,22	n.a.
8		4,527	0,005	0,173	0,02	0,04	n.a.
9		4,650	0,045	0,659	0,14	0,16	n.a.
**Total:**			**31,624**	**411,849**	**100,00**	**100,00**	

Chromeleon (c) Dionex
Version 7.2.9.11323

Figure S107. UPLC chromatogram of compound **89**

Chromatogram and Results

Injection Details

Injection Name:	**TMT33**	*Run Time (min):*	**8,00**
Vial Number:	**BB6**	*Injection Volume:*	**2,00**
Injection Type:	**Unknown**	*Channel:*	**UV_VIS_3**
Calibration Level:		*Wavelength:*	**254**
Instrument Method:	**Purity_PROTAC_short**	*Bandwidth:*	**16**
Processing Method:	**Quantitative**	*Dilution Factor:*	**1,0000**
Injection Date/Time:	**19.dec..22 13:21**	*Sample Weight:*	**1,0000**

Chromatogram

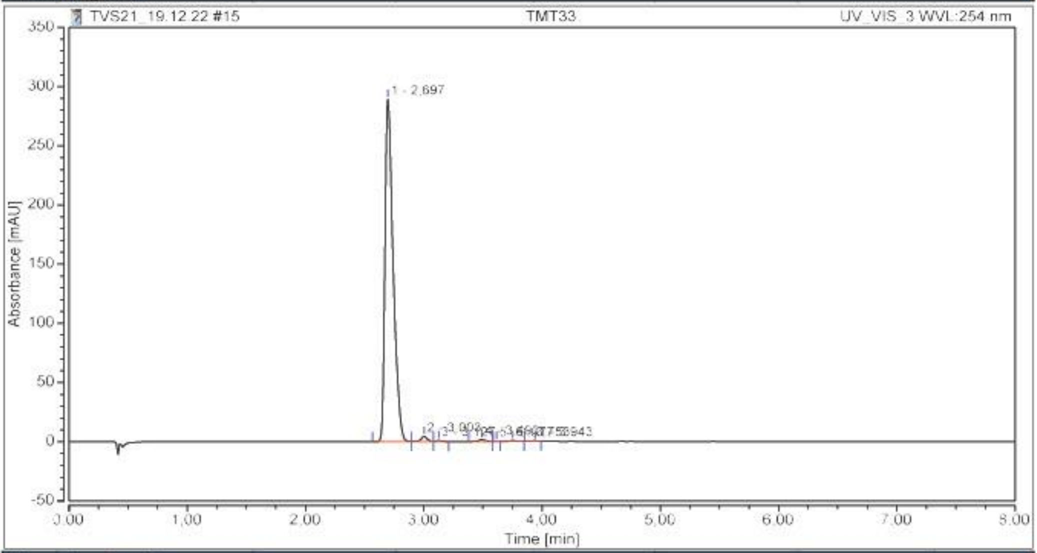

Integration Results

No.	Peak Name	Retention Time min	Area mAU*min	Height mAU	Relative Area %	Relative Height %	Amount n.a.
1		2,697	23,007	289,377	97,66	97,06	n.a.
2		3,003	0,276	4,564	1,17	1,53	n.a.
3		3,127	0,057	0,944	0,24	0,32	n.a.
4		3,493	0,112	1,779	0,47	0,60	n.a.
5		3,617	0,004	0,119	0,02	0,04	n.a.
6		3,753	0,065	0,816	0,27	0,27	n.a.
7		3,943	0,039	0,545	0,17	0,18	n.a.
**Total:**			**23,558**	**298,144**	**100,00**	**100,00**	

Chromeleon (c) Dionex
Version 7.2.9.11323

Figure S108. UPLC chromatogram of compound **91**

Chromatogram and Results

Injection Details

Injection Name:	**TMT21**	*Run Time (min):*	**8,00**
Vial Number:	**BB7**	*Injection Volume:*	**2,00**
Injection Type:	**Unknown**	*Channel:*	**UV_VIS_3**
Calibration Level:		*Wavelength:*	**254**
Instrument Method:	**Purity_PROTAC_short**	*Bandwidth:*	**16**
Processing Method:	**Quantitative**	*Dilution Factor:*	**1,0000**
Injection Date/Time:	**19.dec..22 13:33**	*Sample Weight:*	**1,0000**

Chromatogram

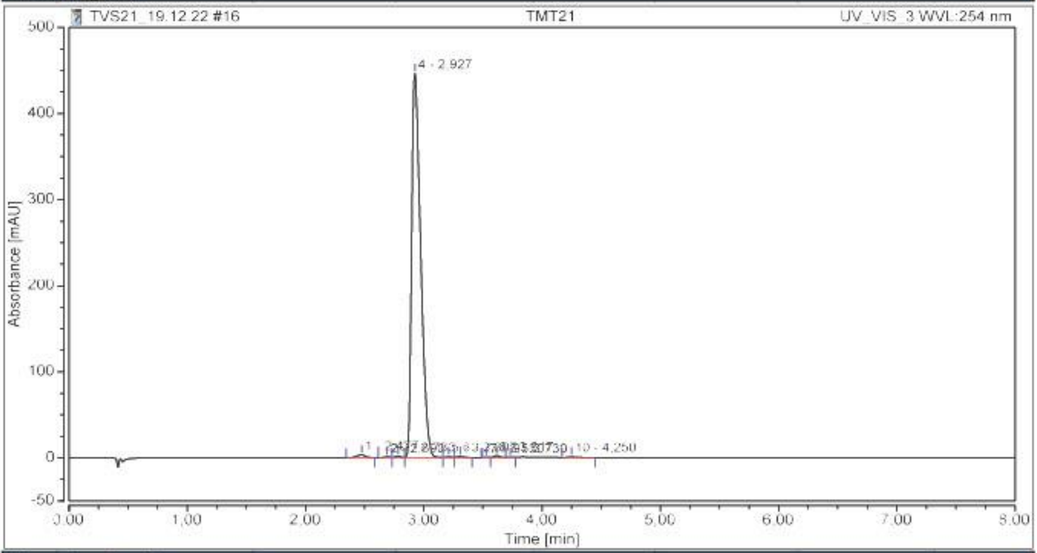

Integration Results

No.	Peak Name	Retention Time min	Area mAU*min	Height mAU	Relative Area %	Relative Height %	Amount n.a.
1		2,477	0,285	3,500	0,71	0,76	n.a.
2		2,690	0,054	0,936	0,13	0,20	n.a.
3		2,783	0,089	1,473	0,22	0,32	n.a.
4		2,927	39,228	446,683	97,98	97,33	n.a.
5		3,210	0,061	1,124	0,15	0,24	n.a.
6		3,307	0,090	1,348	0,23	0,29	n.a.
7		3,530	0,006	0,195	0,02	0,04	n.a.
8		3,617	0,127	2,227	0,32	0,49	n.a.
9		3,730	0,009	0,211	0,02	0,05	n.a.
10		4,250	0,090	1,237	0,22	0,27	n.a.
**Total:**			**40,039**	**458,934**	**100,00**	**100,00**	

Chromeleon (c) Dionex
Version 7.2.9.11323

Figure S109. UPLC chromatogram of compound **93**

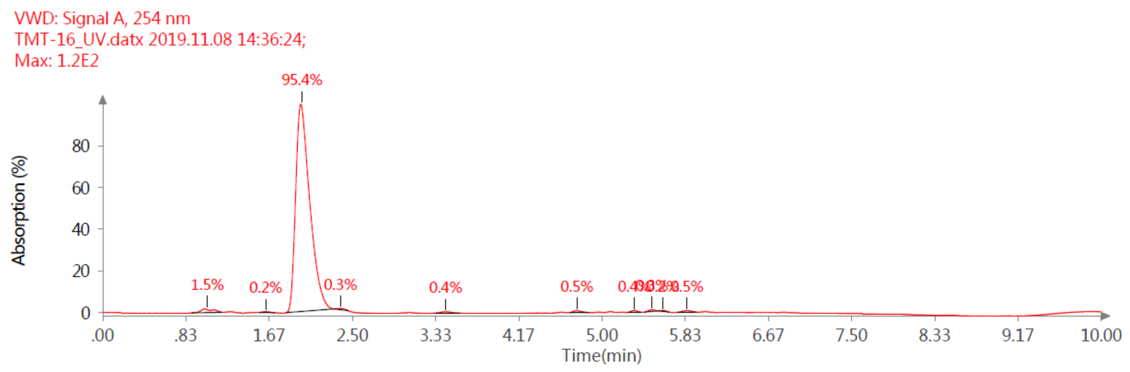

Figure S110. HPLC chromatogram of compound **94**

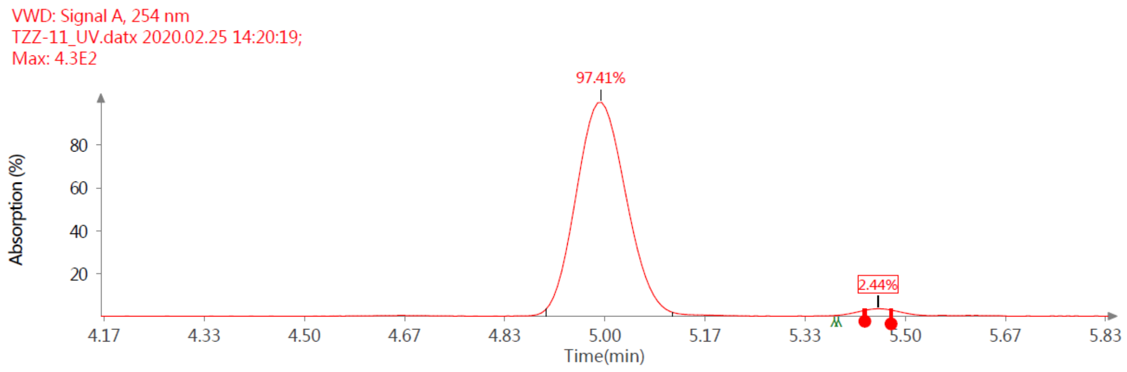

Figure S111. HPLC chromatogram of compound **96**

Chromatogram and Results

Injection Details

Injection Name:	**TZZ59**	*Run Time (min):*	**8,00**
Vial Number:	**BC6**	*Injection Volume:*	**2,00**
Injection Type:	**Unknown**	*Channel:*	**UV_VIS_3**
Calibration Level:		*Wavelength:*	**254**
Instrument Method:	**Purity_PROTAC_short**	*Bandwidth:*	**16**
Processing Method:	**Quantitative**	*Dilution Factor:*	**1,0000**
Injection Date/Time:	**19.dec..22 14:56**	*Sample Weight:*	**1,0000**

Chromatogram

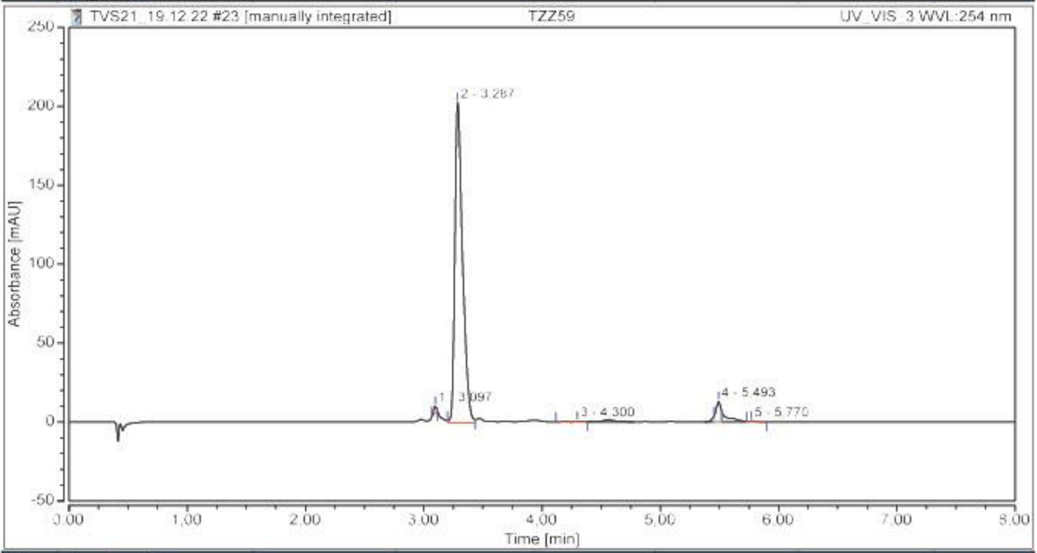

Integration Results

No.	Peak Name	Retention Time min	Area mAU*min	Height mAU	Relative Area %	Relative Height %	Amount n.a.
1		3,097	0,126	4,550	0,79	2,05	n.a.
2		3,287	15,241	202,963	95,03	91,59	n.a.
3		4,300	0,041	0,425	0,26	0,19	n.a.
4		5,493	0,594	13,087	3,71	5,91	n.a.
5		5,770	0,036	0,584	0,22	0,26	n.a.
**Total:**			**16,038**	**221,610**	**100,00**	**100,00**	

Chromeleon (c) Dionex
Version 7.2.9.11323

Figure S112. UPLC chromatogram of compound **97**

Chromatogram and Results

Injection Details

Injection Name:	**TBV7**	*Run Time (min):*	**8,00**
Vial Number:	**BC1**	*Injection Volume:*	**2,00**
Injection Type:	**Unknown**	*Channel:*	**UV_VIS_3**
Calibration Level:		*Wavelength:*	**254**
Instrument Method:	**Purity_PROTAC_short**	*Bandwidth:*	**16**
Processing Method:	**Quantitative**	*Dilution Factor:*	**1,0000**
Injection Date/Time:	**19.dec..22 13:57**	*Sample Weight:*	**1,0000**

Chromatogram

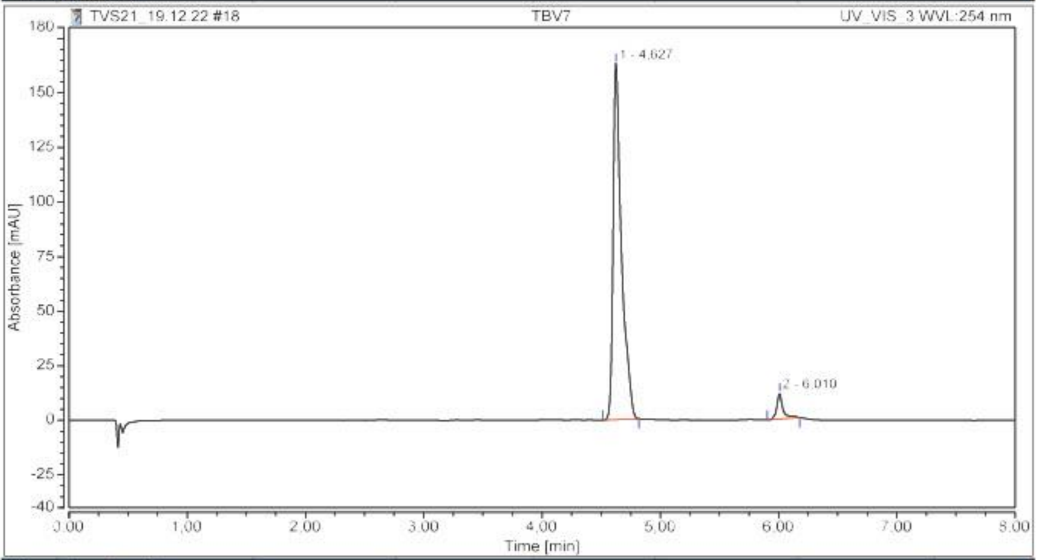

Integration Results

No.	Peak Name	Retention Time min	Area mAU*min	Height mAU	Relative Area %	Relative Height %	Amount n.a.
1		4,627	13,379	163,242	94,89	93,20	n.a.
2		6,010	0,720	11,911	5,11	6,80	n.a.
**Total:**			**14,099**	**175,153**	**100,00**	**100,00**	

Figure S113. UPLC chromatogram of compound **98**

Chromatogram and Results

Injection Details

Injection Name:	**TBV8**	*Run Time (min):*	**8,00**
Vial Number:	**BC2**	*Injection Volume:*	**2,00**
Injection Type:	**Unknown**	*Channel:*	**UV_VIS_3**
Calibration Level:		*Wavelength:*	**254**
Instrument Method:	**Purity_PROTAC_short**	*Bandwidth:*	**16**
Processing Method:	**Quantitative**	*Dilution Factor:*	**1,0000**
Injection Date/Time:	**19.dec..22 14:08**	*Sample Weight:*	**1,0000**

Chromatogram

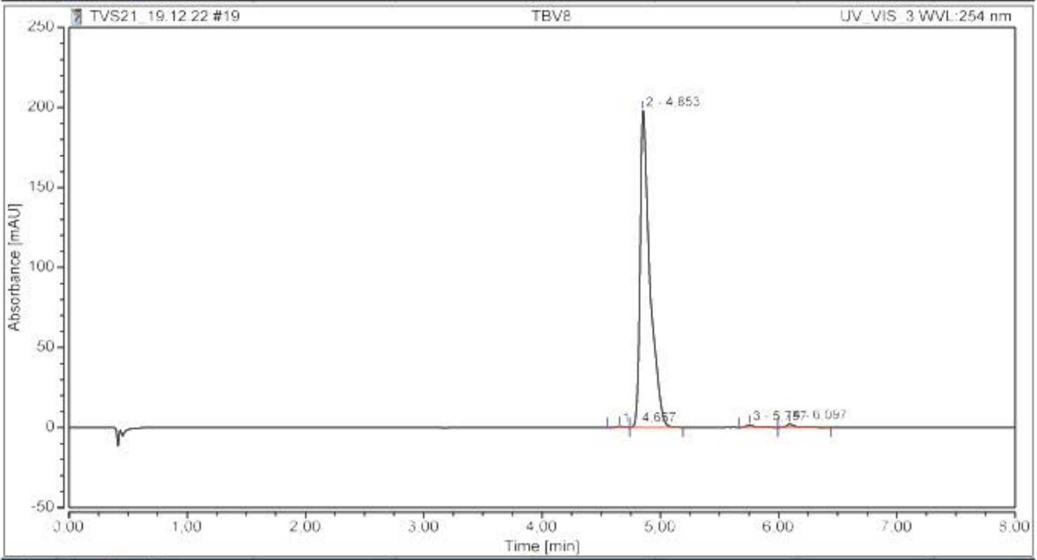

Integration Results

No.	Peak Name	Retention Time min	Area mAU*min	Height mAU	Relative Area %	Relative Height %	Amount n.a.
1		4,657	0,038	0,609	0,20	0,30	n.a.
2		4,853	19,079	197,844	98,11	97,84	n.a.
3		5,757	0,139	1,539	0,71	0,76	n.a.
4		6,097	0,191	2,212	0,98	1,09	n.a.
**Total:**			**19,446**	**202,205**	**100,00**	**100,00**	

Chromeleon (c) Dionex
Version 7.2.9.11323

Figure S114. UPLC chromatogram of compound **99**

Chromatogram and Results

Injection Details

Injection Name:	**TBV9**	*Run Time (min):*	**8,00**
Vial Number:	**BC3**	*Injection Volume:*	**2,00**
Injection Type:	**Unknown**	*Channel:*	**UV_VIS_3**
Calibration Level:		*Wavelength:*	**254**
Instrument Method:	**Purity_PROTAC_short**	*Bandwidth:*	**16**
Processing Method:	**Quantitative**	*Dilution Factor:*	**1,0000**
Injection Date/Time:	**19.dec..22 14:20**	*Sample Weight:*	**1,0000**

Chromatogram

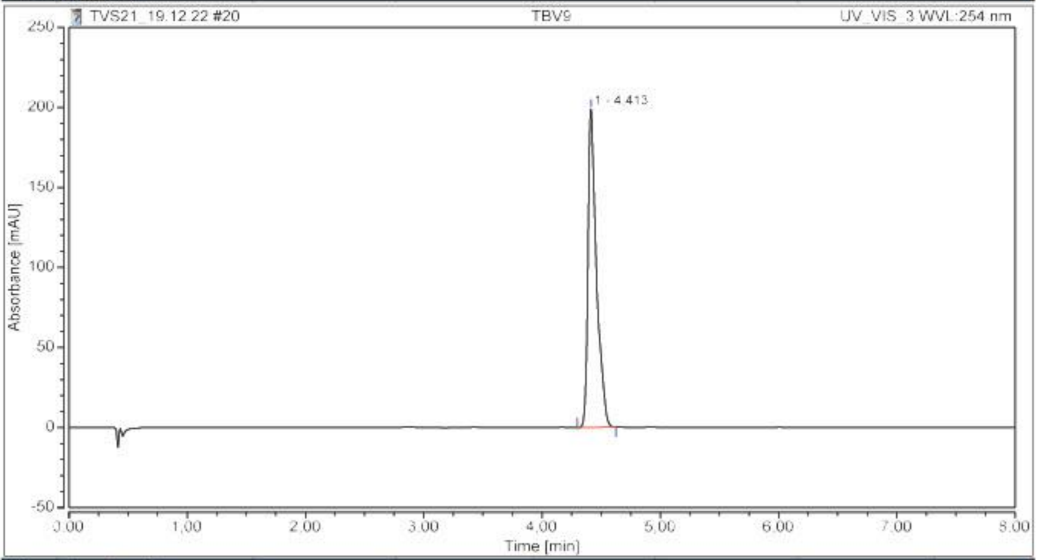

Integration Results

No.	Peak Name	Retention Time min	Area mAU*min	Height mAU	Relative Area %	Relative Height %	Amount n.a.
1		4,413	16,626	198,930	100,00	100,00	n.a.
**Total:**			**16,626**	**198,930**	**100,00**	**100,00**	

Figure S115. UPLC chromatogram of compound **100**

Chromatogram and Results

Injection Details

Injection Name:	TBV13	*Run Time (min):*	8,00
Vial Number:	BB8	*Injection Volume:*	2,00
Injection Type:	Unknown	*Channel:*	UV_VIS_3
Calibration Level:		*Wavelength:*	254
Instrument Method:	Purity_PROTAC_short	*Bandwidth:*	16
Processing Method:	Quantitative	*Dilution Factor:*	1,0000
Injection Date/Time:	19.dec..22 13:45	*Sample Weight:*	1,0000

Chromatogram

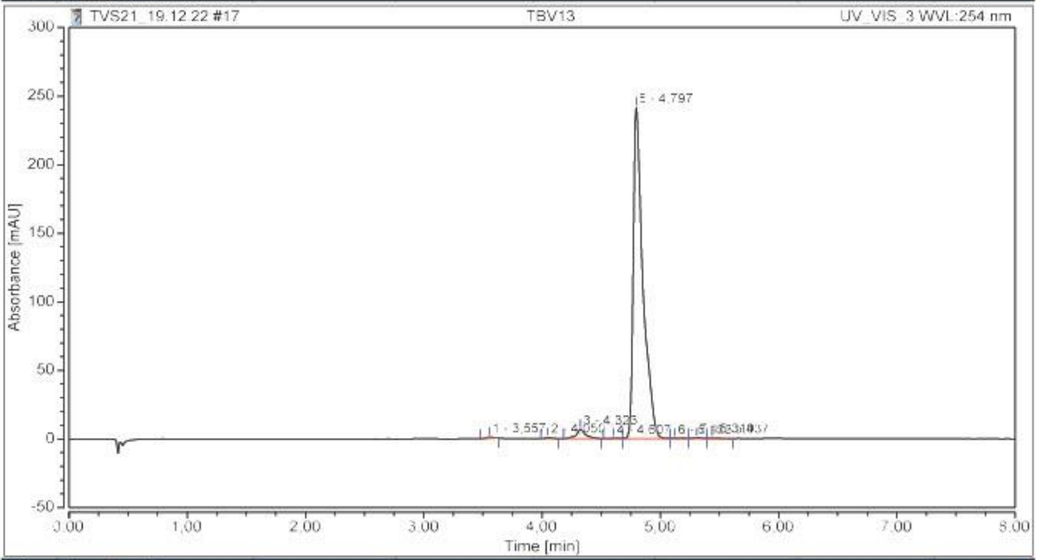

Integration Results

No.	Peak Name	Retention Time min	Area mAU*min	Height mAU	Relative Area %	Relative Height %	Amount n.a.
1		3,557	0,061	0,930	0,26	0,37	n.a.
2		4,050	0,044	0,701	0,19	0,28	n.a.
3		4,323	0,631	7,070	2,67	2,80	n.a.
4		4,607	0,031	0,376	0,13	0,15	n.a.
5		4,797	22,707	241,726	96,16	95,80	n.a.
6		5,123	0,038	0,322	0,16	0,13	n.a.
7		5,310	0,067	0,759	0,28	0,30	n.a.
8		5,437	0,035	0,439	0,15	0,17	n.a.
Total:			23,613	252,324	100,00	100,00	

Chromeleon (c) Dionex
Version 7.2.9.11323

Figure S116. UPLC chromatogram of compound **101**

Chromatogram and Results

Injection Details			
Injection Name:	**TBV16**	*Run Time (min):*	**8,00**
Vial Number:	**BC4**	*Injection Volume:*	**2,00**
Injection Type:	**Unknown**	*Channel:*	**UV_VIS_3**
Calibration Level:		*Wavelength:*	**254**
Instrument Method:	**Purity_PROTAC_short**	*Bandwidth:*	**16**
Processing Method:	**Quantitative**	*Dilution Factor:*	**1,0000**
Injection Date/Time:	**19.dec..22 14:32**	*Sample Weight:*	**1,0000**

Chromatogram

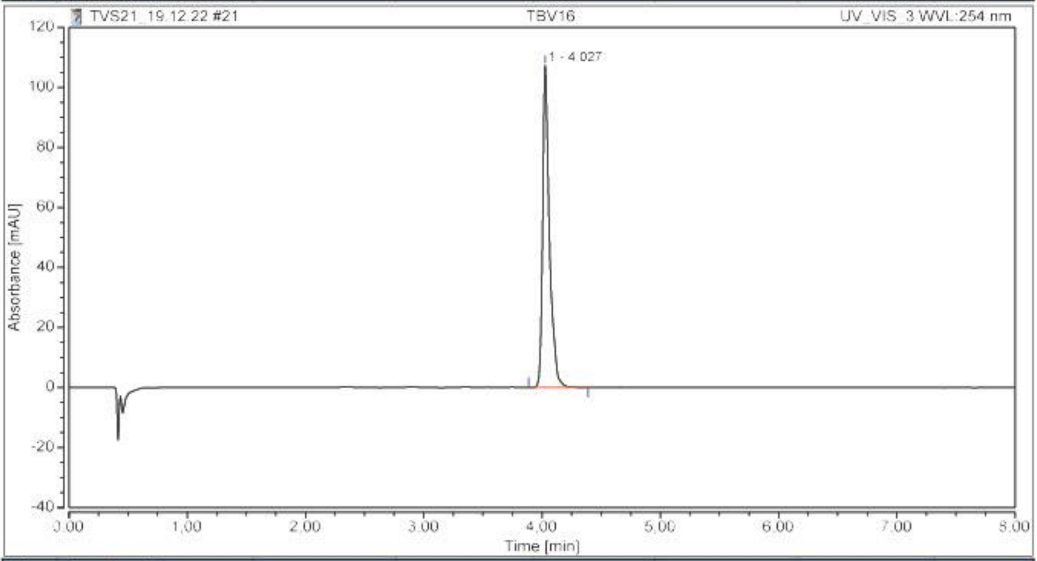

Integration Results

No.	Peak Name	Retention Time min	Area mAU*min	Height mAU	Relative Area %	Relative Height %	Amount n.a.
1		4,027	7,148	107,387	100,00	100,00	n.a.
**Total:**			**7,148**	**107,387**	**100,00**	**100,00**	

Figure S117. UPLC chromatogram of compound **102**

Instrument:ULTIMATE3000 Sequence:TVS21_19.12.22 Page 43 of 54

Chromatogram and Results

Injection Details

Injection Name:	**TAT17**	*Run Time (min):*	**8,00**
Vial Number:	**BC5**	*Injection Volume:*	**2,00**
Injection Type:	**Unknown**	*Channel:*	**UV_VIS_3**
Calibration Level:		*Wavelength:*	**254**
Instrument Method:	**Purity_PROTAC_short**	*Bandwidth:*	**16**
Processing Method:	**Quantitative**	*Dilution Factor:*	**1,0000**
Injection Date/Time:	**19.dec..22 14:44**	*Sample Weight:*	**1,0000**

Chromatogram

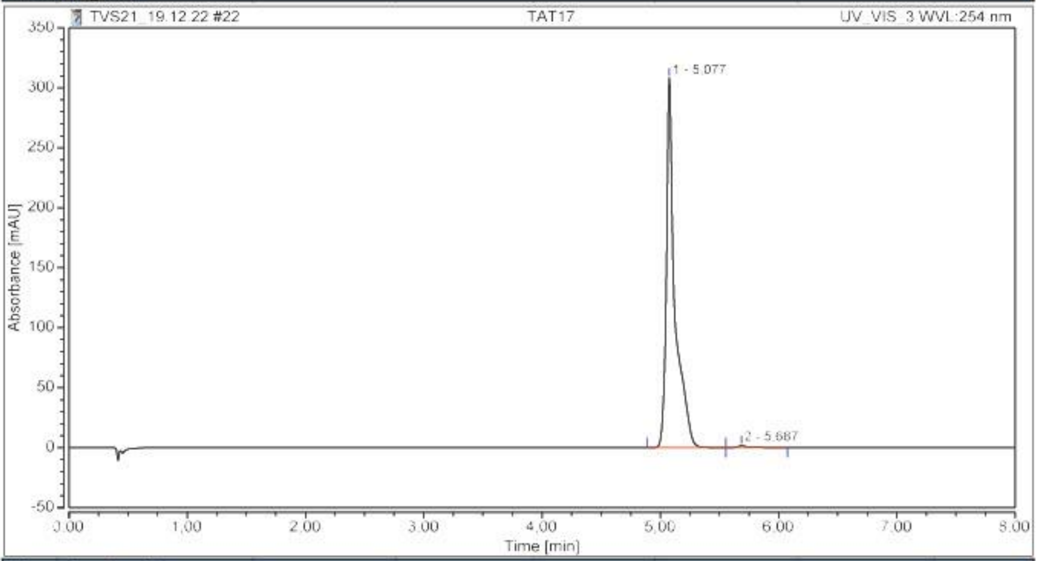

Integration Results

No.	Peak Name	Retention Time min	Area mAU*min	Height mAU	Relative Area %	Relative Height %	Amount n.a.
1		5,077	25,943	308,255	99,37	99,46	n.a.
2		5,687	0,164	1,677	0,63	0,54	n.a.
**Total:**			**26,108**	**309,932**	**100,00**	**100,00**	

TVS21_19.12.22/Integration

Chromeleon (c) Dionex
Version 7.2.9.11323

Figure S118. UPLC chromatogram of compound **103**

Chromatogram and Results

Injection Details

Injection Name:	TZZ-103-F4	*Run Time (min):*	8,00
Vial Number:	RA4	*Injection Volume:*	2,00
Injection Type:	Unknown	*Channel:*	UV_VIS_3
Calibration Level:		*Wavelength:*	254
Instrument Method:	Purity_PROTAC_short	*Bandwidth:*	16
Processing Method:	Quantitative	*Dilution Factor:*	1,0000
Injection Date/Time:	11.jan..22 14:05	*Sample Weight:*	1,0000

Chromatogram

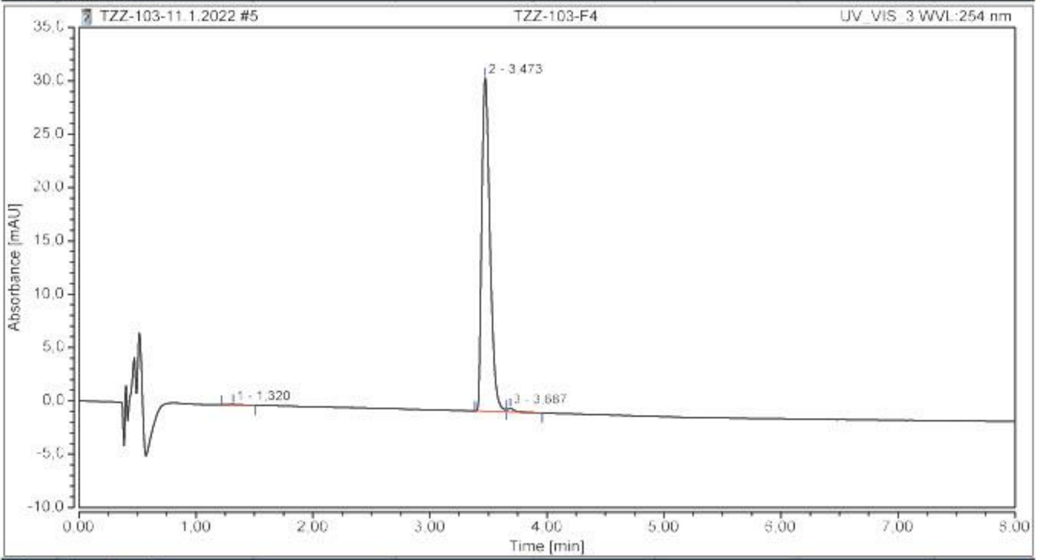

Integration Results

No.	Peak Name	Retention Time min	Area mAU*min	Height mAU	Relative Area %	Relative Height %	Amount n.a.
1		1,320	0,005	0,049	0,21	0,15	n.a.
2		3,473	2,536	31,277	98,82	98,76	n.a.
3		3,687	0,025	0,345	0,98	1,09	n.a.
Total:			2,566	31,671	100,00	100,00	

Figure S119. UPLC chromatogram of compound **104**

Instrument:ULTIMATE3000 Sequence:TVS21_19.12.22 Page 45 of 54

Chromatogram and Results

Injection Details

Injection Name:	**TZZ59**	*Run Time (min):*	**8,00**
Vial Number:	**BC6**	*Injection Volume:*	**2,00**
Injection Type:	**Unknown**	*Channel:*	**UV_VIS_3**
Calibration Level:		*Wavelength:*	**254**
Instrument Method:	**Purity_PROTAC_short**	*Bandwidth:*	**16**
Processing Method:	**Quantitative**	*Dilution Factor:*	**1,0000**
Injection Date/Time:	**19.dec..22 14:56**	*Sample Weight:*	**1,0000**

Chromatogram

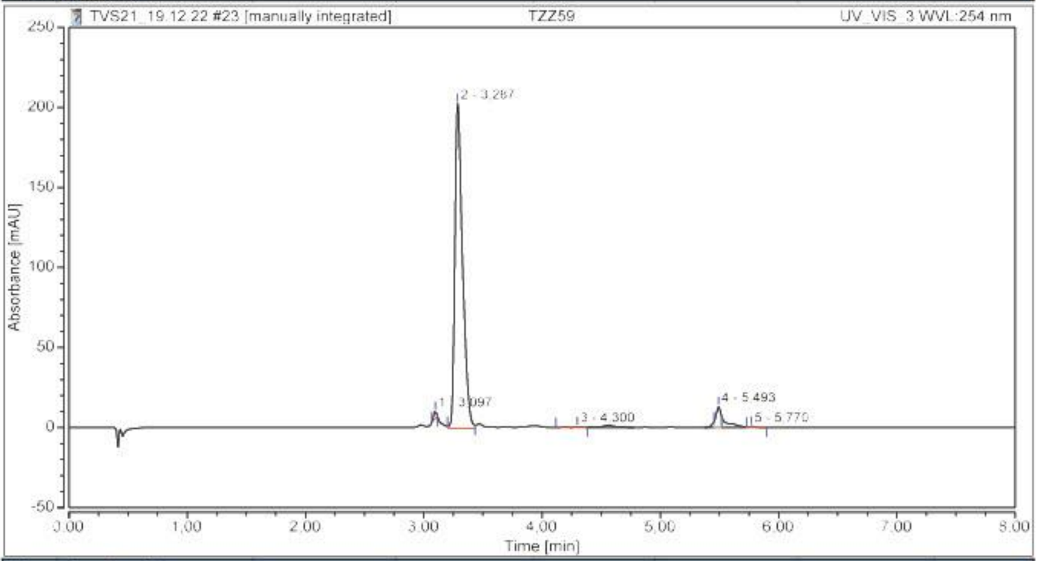

Integration Results

No.	Peak Name	Retention Time min	Area mAU*min	Height mAU	Relative Area %	Relative Height %	Amount n.a.
1		3,097	0,126	4,550	0,79	2,05	n.a.
2		3,287	15,241	202,963	95,03	91,59	n.a.
3		4,300	0,041	0,425	0,26	0,19	n.a.
4		5,493	0,594	13,087	3,71	5,91	n.a.
5		5,770	0,036	0,584	0,22	0,26	n.a.
**Total:**			**16,038**	**221,610**	**100,00**	**100,00**	

TVS21_19.12.22/Integration

Chromeleon (c) Dionex
Version 7.2.9.11323

Figure S120. UPLC chromatogram of compound **105**

Chromatogram and Results

Injection Details

Injection Name:	**TMT34**	*Run Time (min):*	**8,00**
Vial Number:	**BC7**	*Injection Volume:*	**2,00**
Injection Type:	**Unknown**	*Channel:*	**UV_VIS_3**
Calibration Level:		*Wavelength:*	**254**
Instrument Method:	**Purity_PROTAC_short**	*Bandwidth:*	**16**
Processing Method:	**Quantitative**	*Dilution Factor:*	**1,0000**
Injection Date/Time:	**19.dec..22 15:08**	*Sample Weight:*	**1,0000**

Chromatogram

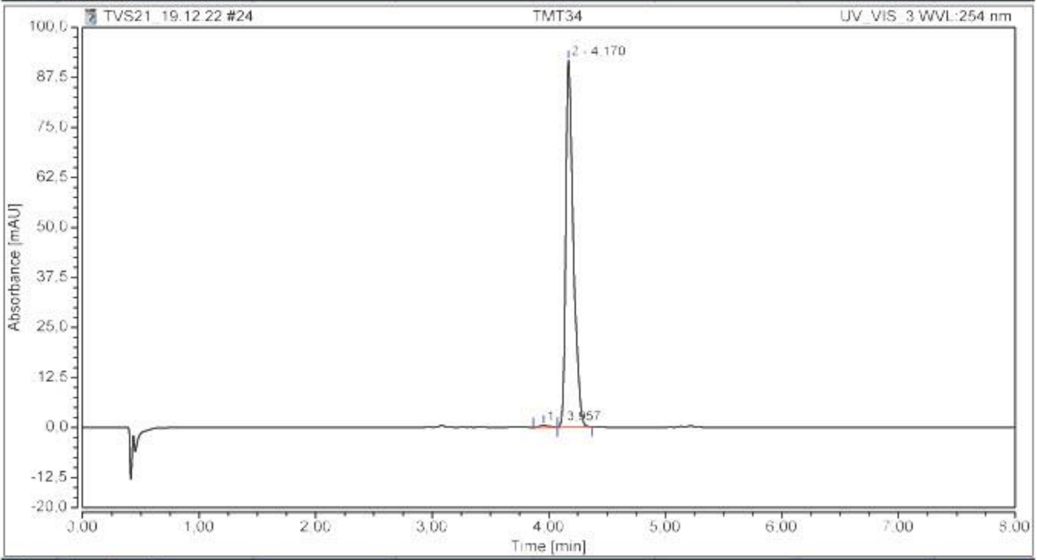

Integration Results

No.	Peak Name	Retention Time min	Area mAU*min	Height mAU	Relative Area %	Relative Height %	Amount n.a.
1		3,957	0,046	0,542	0,66	0,59	n.a.
2		4,170	6,932	91,727	99,34	99,41	n.a.
**Total:**			**6,977**	**92,269**	**100,00**	**100,00**	

Chromeleon (c) Dionex
Version 7.2.9.11323

Figure S121. UPLC chromatogram of compound **106**

Chromatogram and Results

Injection Details

Injection Name:	TZZ28	*Run Time (min):*	8,00
Vial Number:	BD1	*Injection Volume:*	2,00
Injection Type:	Unknown	*Channel:*	UV_VIS_3
Calibration Level:		*Wavelength:*	254
Instrument Method:	Purity_PROTAC_short	*Bandwidth:*	16
Processing Method:	Quantitative	*Dilution Factor:*	1,0000
Injection Date/Time:	19.dec..22 15:32	*Sample Weight:*	1,0000

Chromatogram

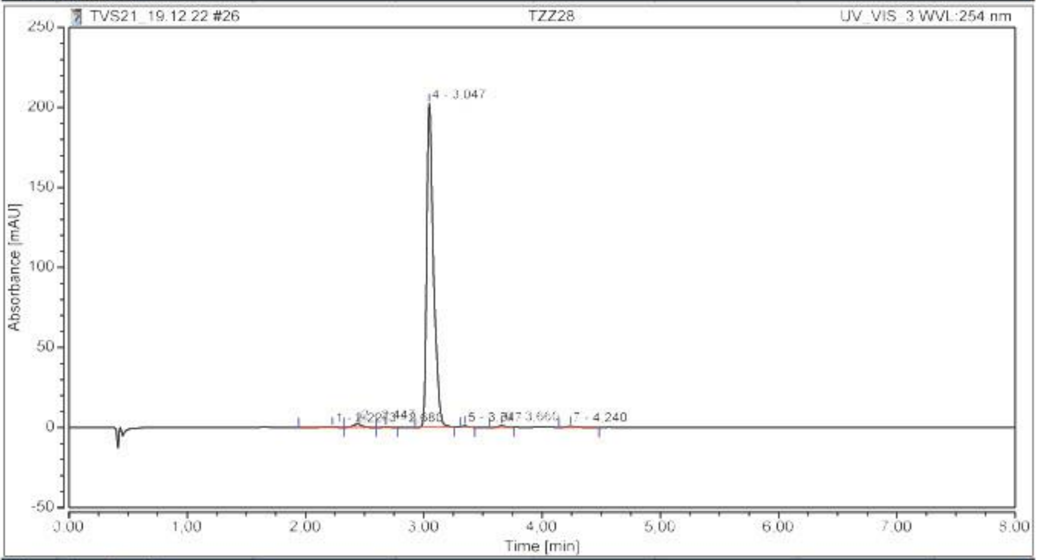

Integration Results

No.	Peak Name	Retention Time min	Area mAU*min	Height mAU	Relative Area %	Relative Height %	Amount n.a.
1		2,227	0,054	0,457	0,39	0,22	n.a.
2		2,447	0,185	2,144	1,32	1,03	n.a.
3		2,680	0,035	0,561	0,25	0,27	n.a.
4		3,047	13,507	202,183	96,79	97,26	n.a.
5		3,347	0,032	0,756	0,23	0,36	n.a.
6		3,660	0,071	1,124	0,51	0,54	n.a.
7		4,240	0,071	0,662	0,51	0,32	n.a.
Total:			13,954	207,887	100,00	100,00	

Chromeleon (c) Dionex
Version 7.2.9.11323

Figure S122. UPLC chromatogram of compound **119**

1.10 WB images used for quantification

Table S3: Annotation of samples in individual lanes for MDA-MB-231 cell line. All membranes have the same order of samples.

Lane	Sample
1	non-treated cells, 1st biological repetition
2	**104** 5 µM, 1st biological repetition
3	**104** 20 µM, 1st biological repetition
4	**89** 5 µM, 1st biological repetition
5	**89** 20 µM, 1st biological repetition
6	17-DMAG 0.5 µM, 1st biological repetition
7	Non treated cells, 2nd biological repetition
8	**104** 5 µM, 2nd biological repetition
9	**104** 20 µM, 2nd biological repetition
10	**89** 5 µM, 2nd biological repetition
11	**89** 20 µM, 2nd biological repetition
12	17-DMAG 0.5 µM, 2nd biological repetition

Table S4: Annotation of samples in individual lanes for MCF-7 cell line. All membranes have the same order of samples.

Lane	Sample
1	non-treated cells
2	17-DMAG, 0.5 µM
3	**104** 10 µM
4	**104** 5 µM
5	**89** 10 µM
6	**89** 5 µM

Table S5: Annotation of samples in individual lanes for SKBr3 cell line. All membranes have the same order of samples.

Lane	Sample
1	non-treated cells
2	17-DMAG, 0.5 µM
3	**104** 10 µM
4	**104** 5 µM
5	**89** 10 µM
6	**89** 5 µM

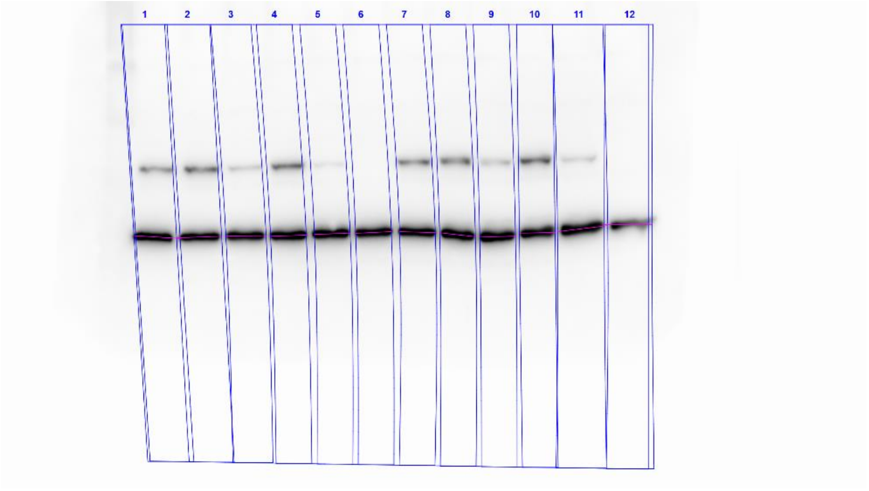

Figure S123. Image used for quantification: GAPDH, 1st and 2nd biological repetitions, MDA-MB-231

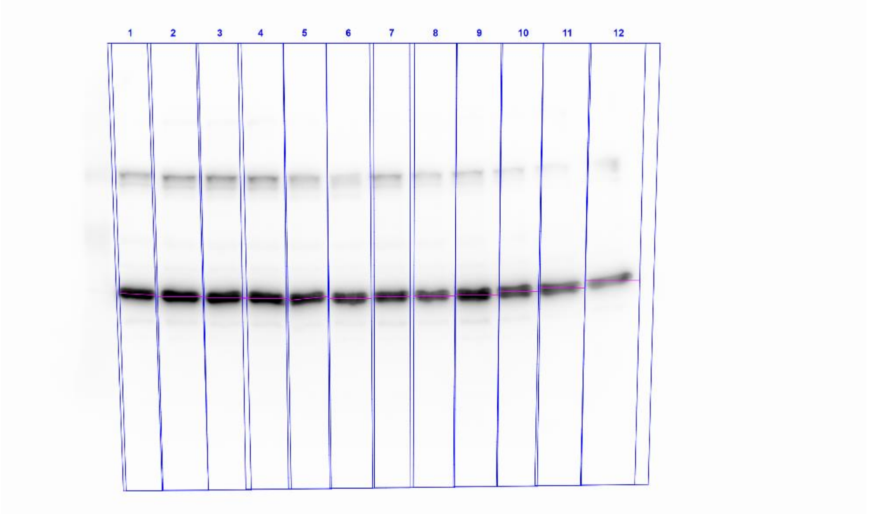

Figure S124. Image used for quantification: GAPDH, 1st and 2nd biological repetitions, MDA-MB-231

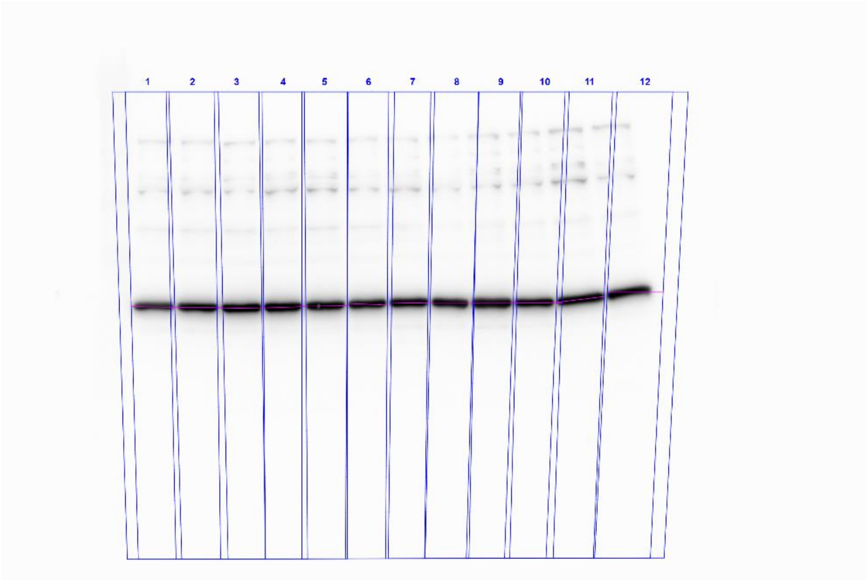

Figure S125. Image used for quantification: GAPDH, 1st and 2nd biological repetitions, MDA-MB-231

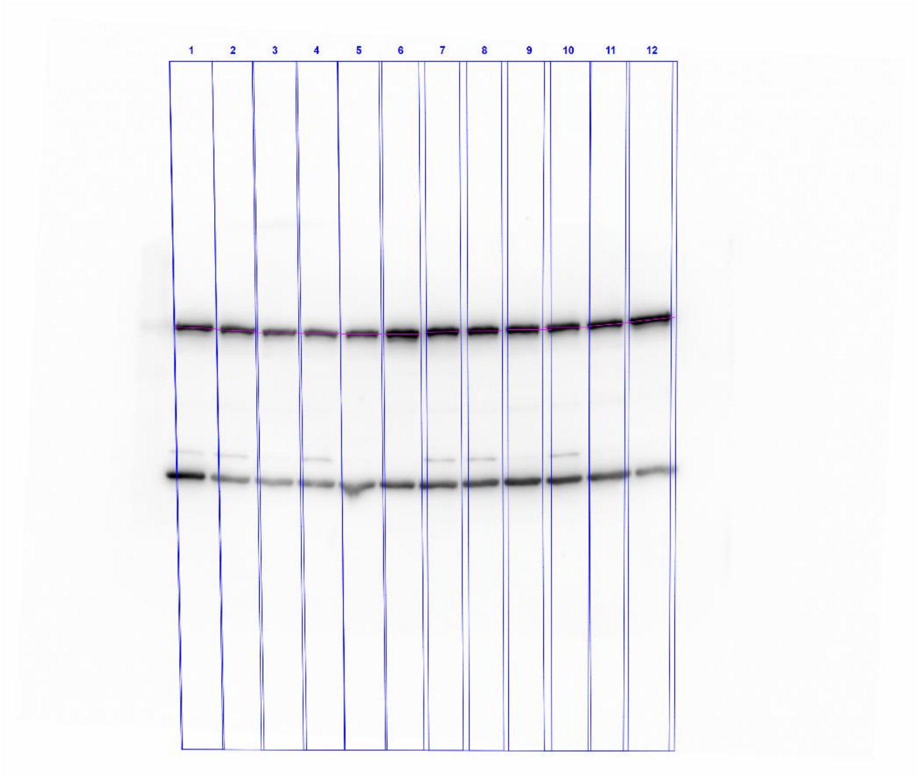

Figure S126. Image used for quantification: Hsp90, 1st and 2nd biological repetitions, MDA-MB-231

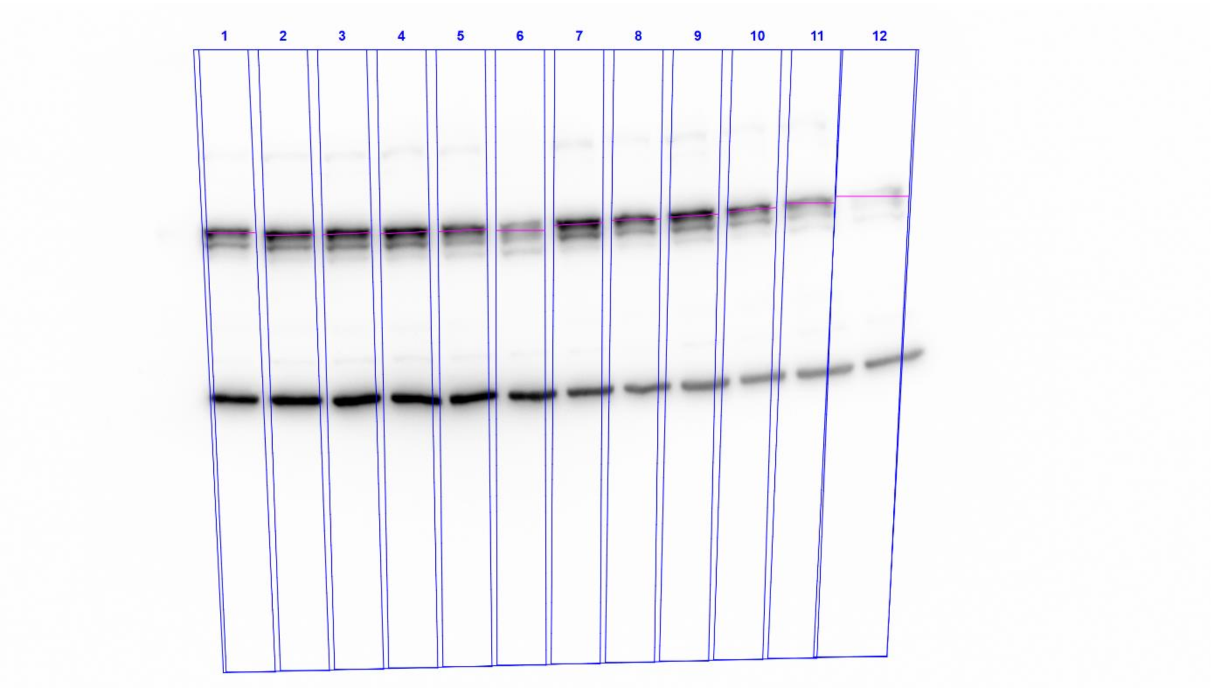

Figure S127. Image used for quantification: Akt, 1st and 2nd biological repetitions, MDA-MB-231

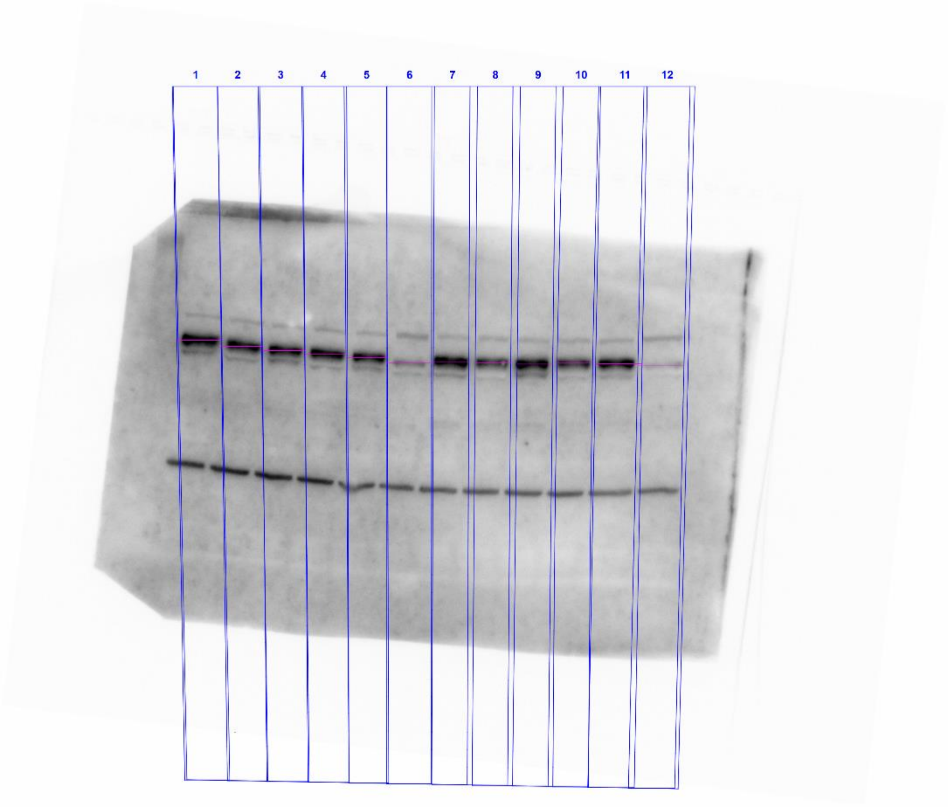

Figure S128. Image used for quantification: c-Raf, 1st and 2nd biological repetitions, MDA-MB-231

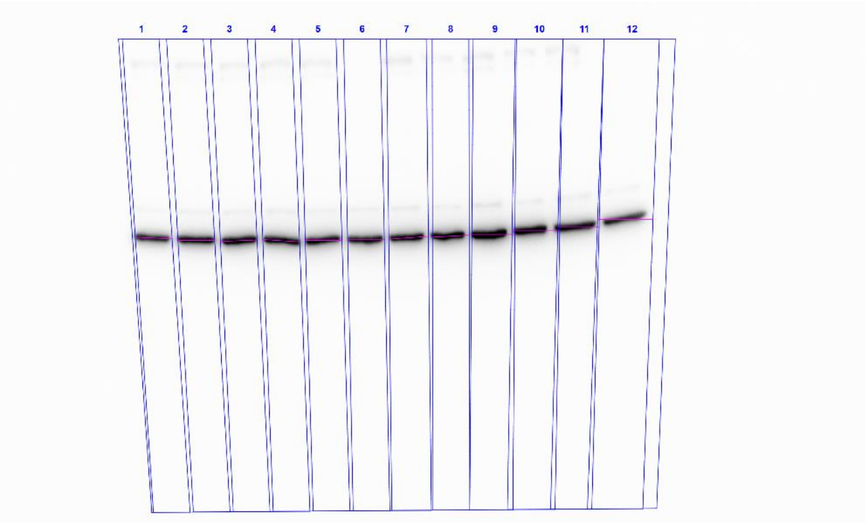

Figure S129. Image used for quantification: ERK, 1st and 2nd biological repetitions, MDA-MB-231

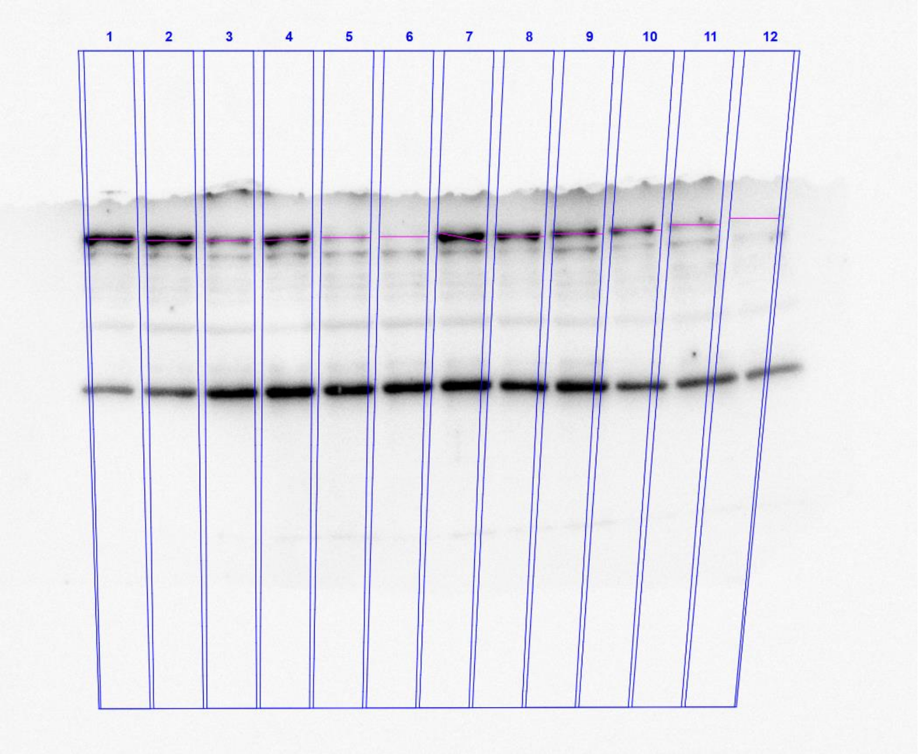

Figure S130. Image used for quantification: p-Akt, 1st and 2nd biological repetitions, MDA-MB-231

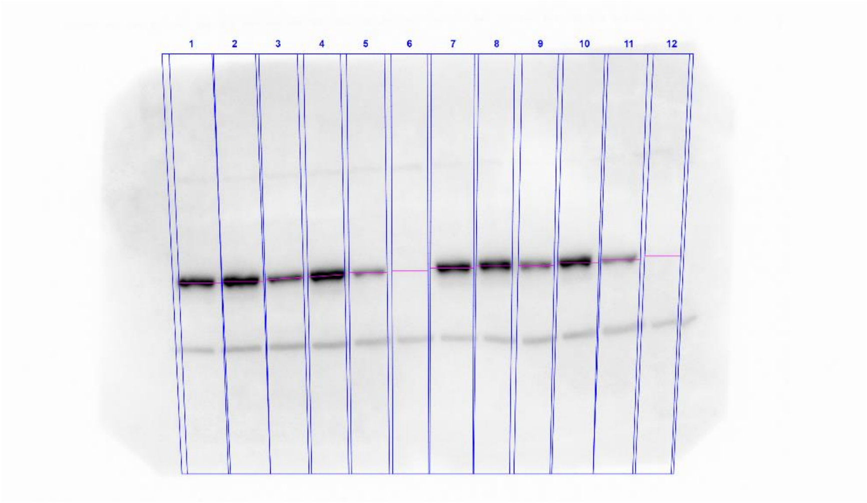

Figure S131. Image used for quantification: p-MEK, 1st and 2nd biological repetitions, MDA-MB-231

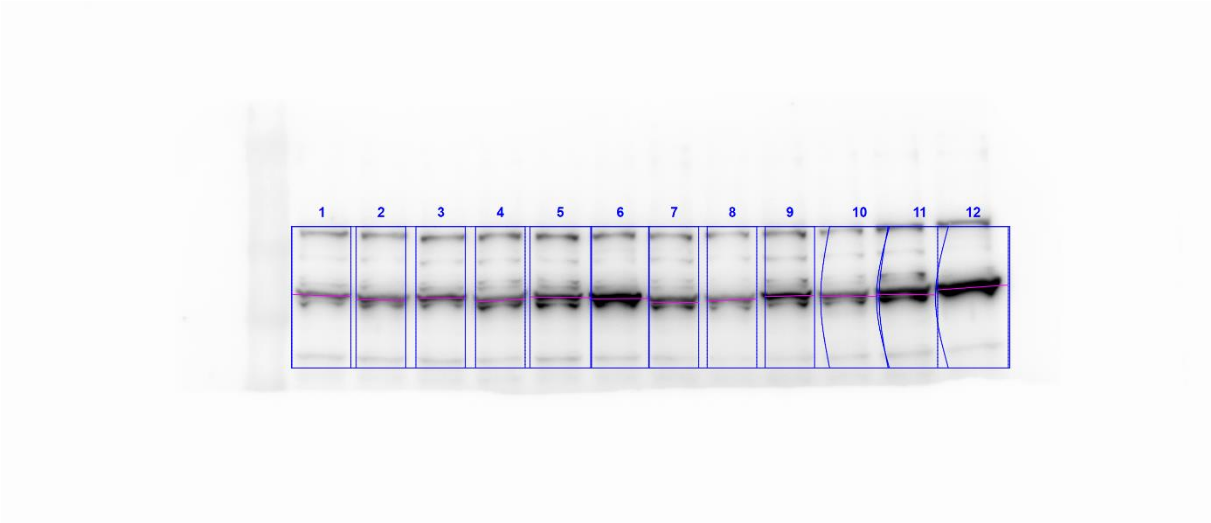

Figure S132. Image used for quantification: Hsp70, 1st and 2nd biological repetitions, MDA-MB-231

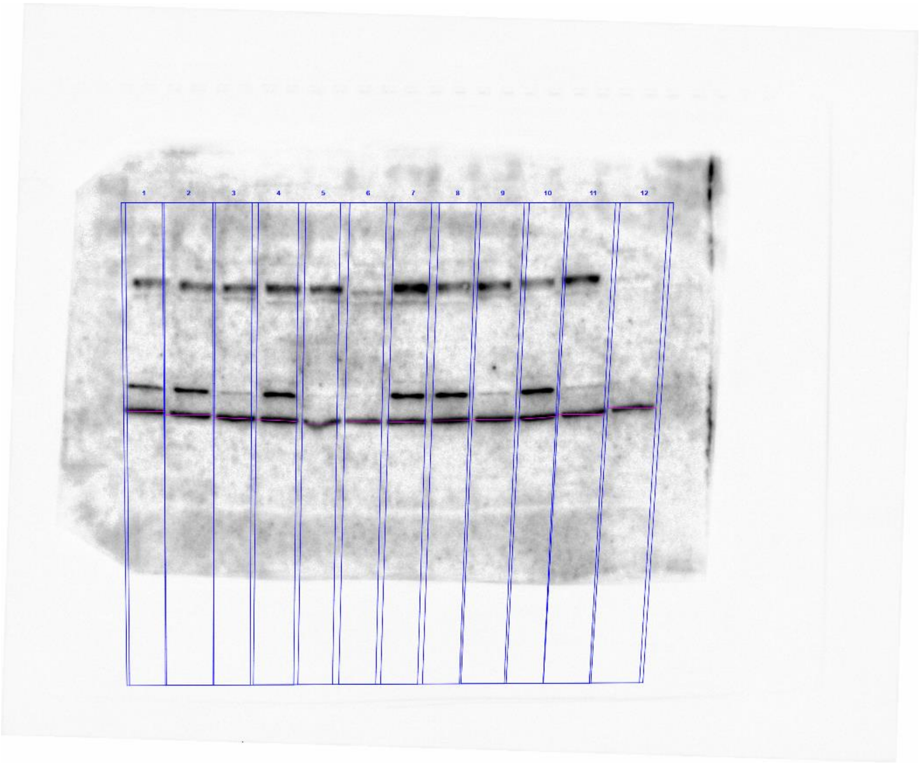

Figure S133. Image used for quantification: p-ERK, 1st and 2nd biological repetitions, MDA-MB-231

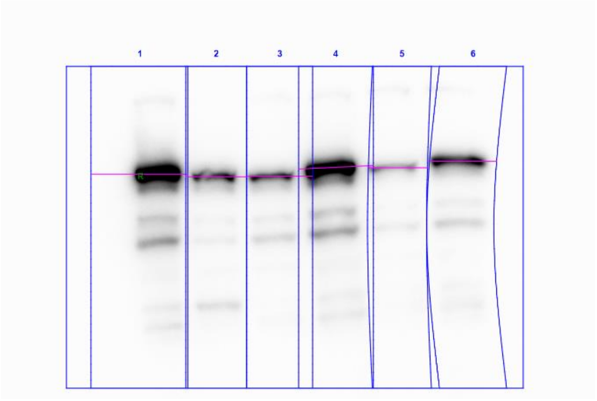

Figure S134. Image used for quantification: ERα, 1st biological repetition, MCF-7

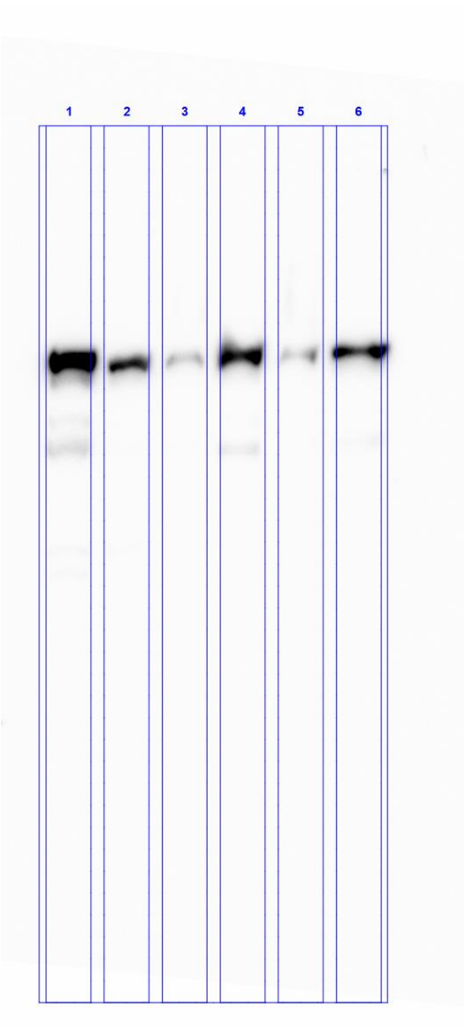

Figure S135. Image used for quantification: ERα, 2nd biological repetition, MCF-7

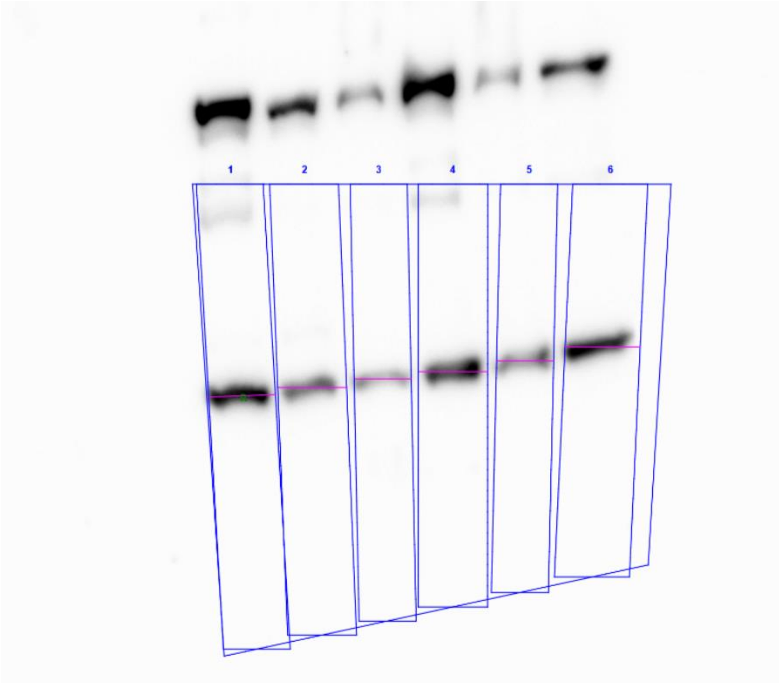

Figure S136. Image used for quantification: CDK4, 1st biological repetition, MCF-7

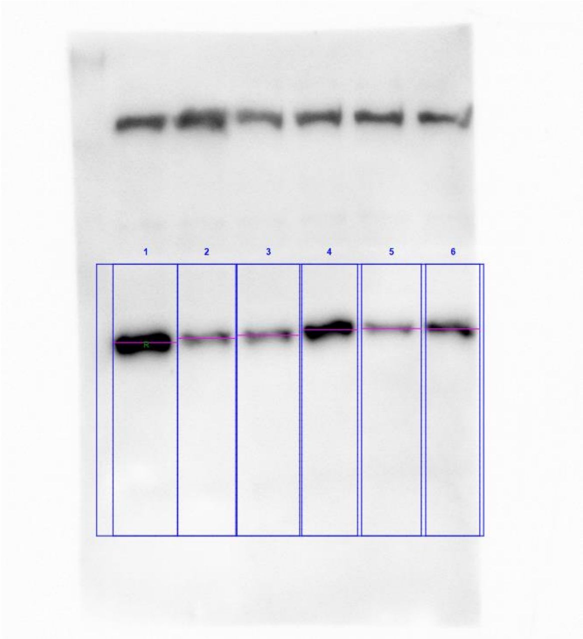

Figure S137. Image used for quantification: CDK4, 2nd biological repetition, MCF-7

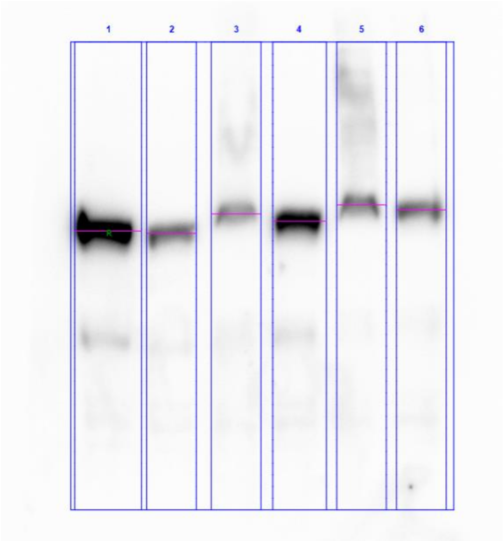

Figure S138. Image used for quantification: c-Raf, 2nd biological repetition, MCF-7

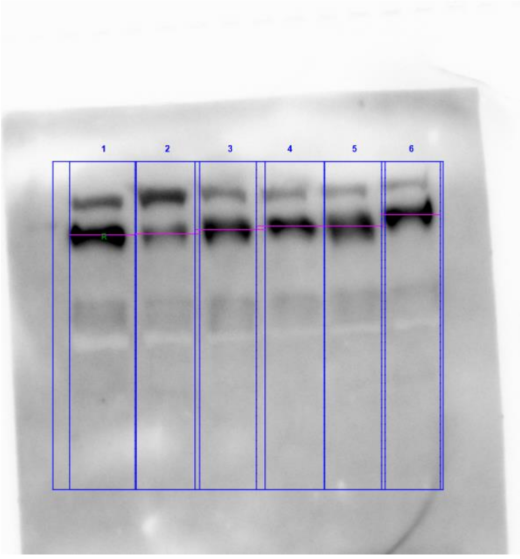

Figure S139. Image used for quantification: cRaf, 1st biological repetition, MCF-7

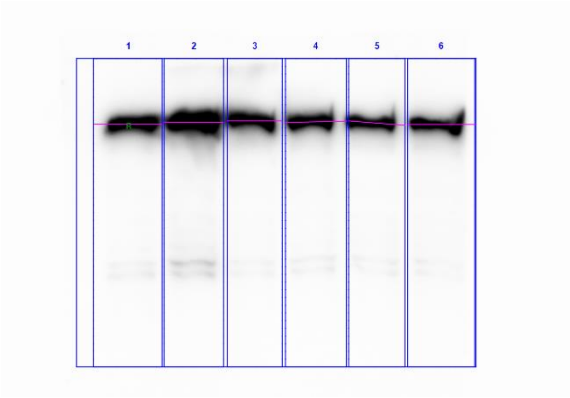

Figure S140. Image used for quantification: Hsp90, 1st biological repetition, MCF-7

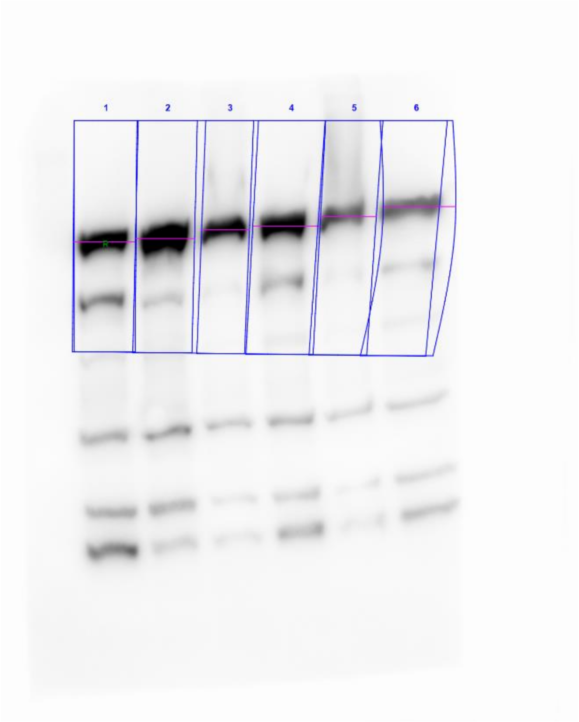

Figure S141. Image used for quantification: Hsp90, 2nd biological repetition, MCF-7

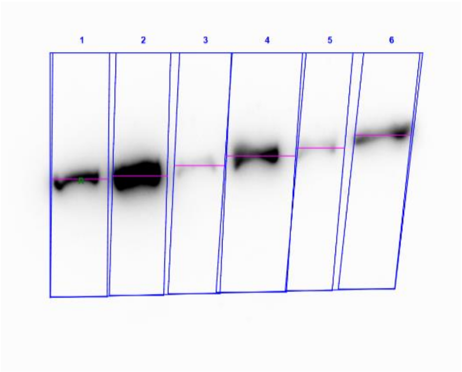

Figure S142. Image used for quantification: Hsp70, 1st biological repetition, MCF-7

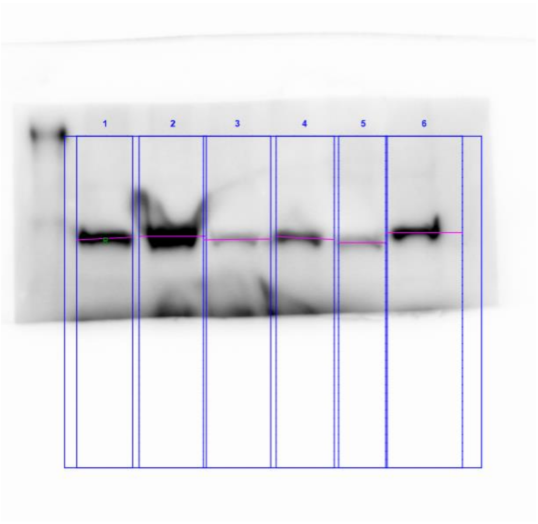

Figure S143. Image used for quantification: Hsp70, 2nd biological repetition, MCF-7

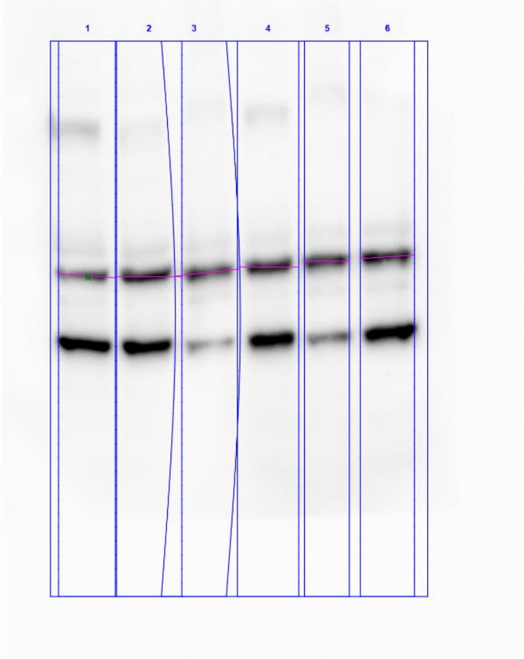

Figure S144. Image used for quantification: β-actin, MCF-7

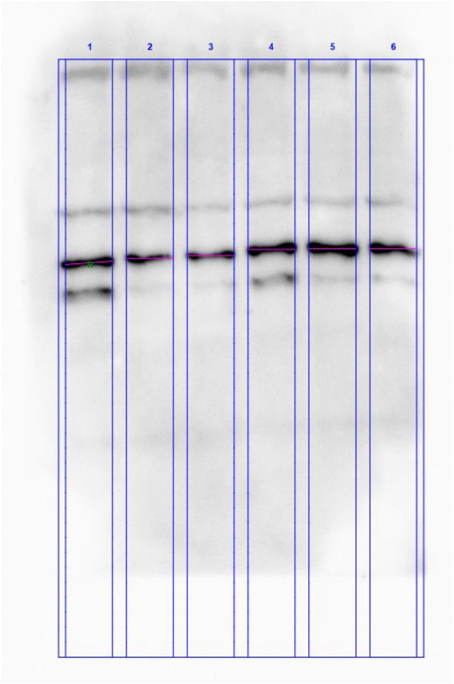

Figure S145. Image used for quantification: β-actin, MCF-7

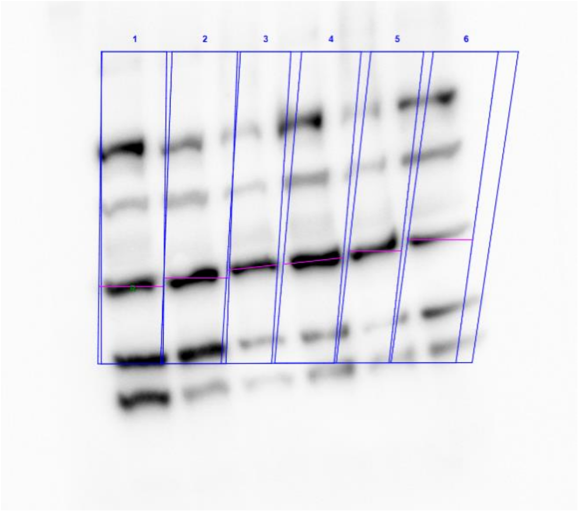

Figure S146. Image used for quantification: β-actin, MCF-7

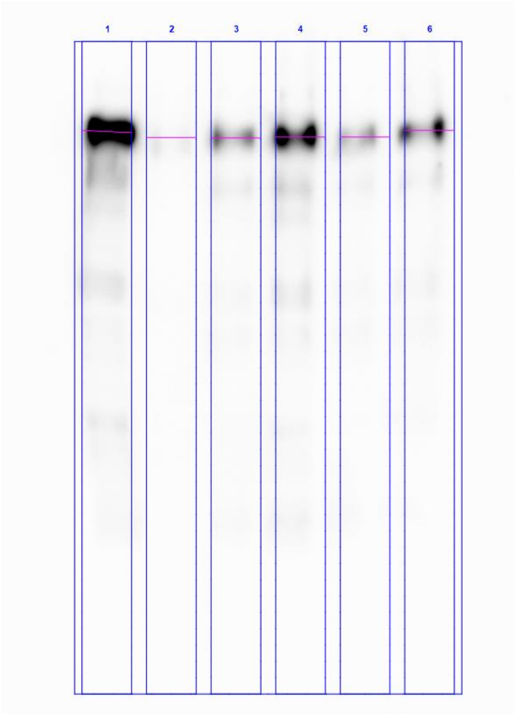

Figure S147. Image used for quantification: Her2, 1st biological repetition, SKBr3

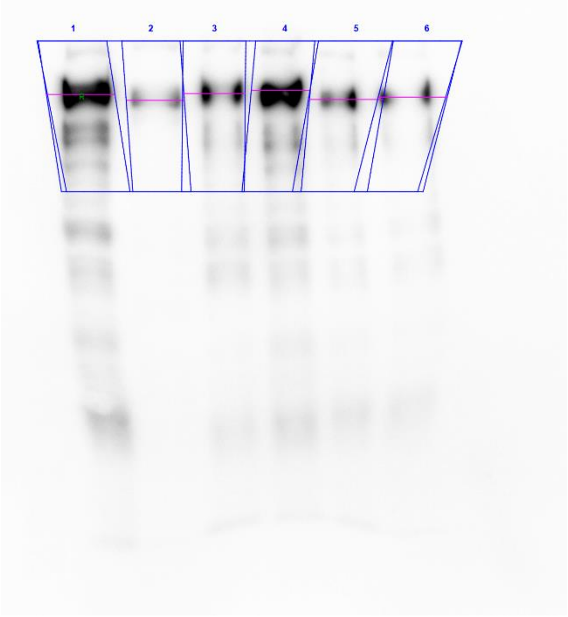

Figure S148. Image used for quantification: Her2, 2nd biological repetition, SKBr3

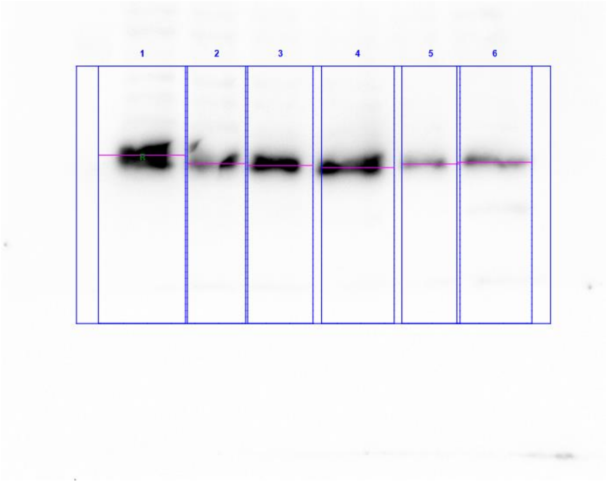

Figure S149. Image used for quantification: CDK4, 1st biological repetition, SKBr3

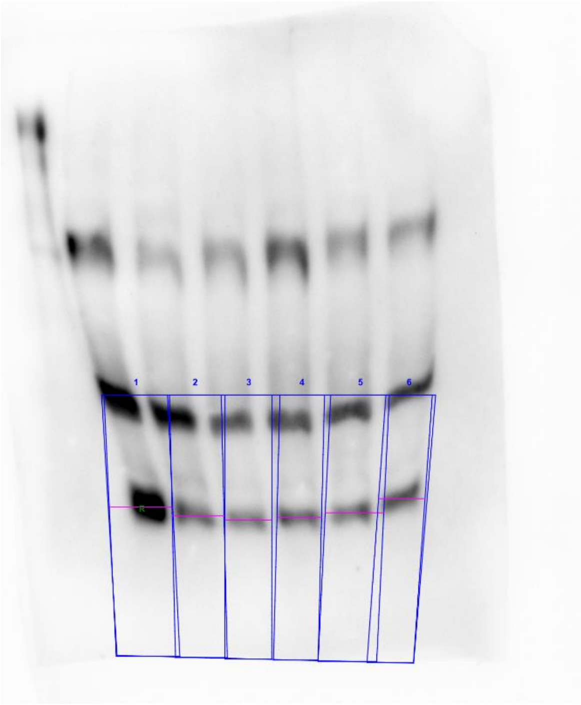

Figure S150. Image used for quantification: CDK4, 2nd biological repetition, SKBr3

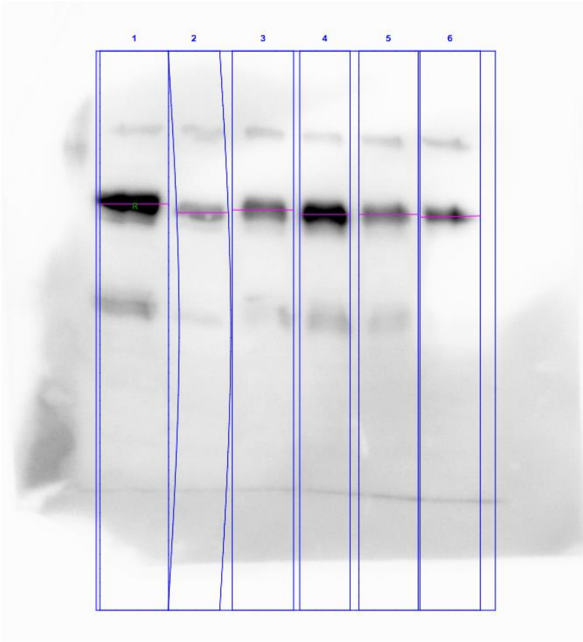

Figure S151. Image used for quantification: cRaf, 2nd biological repetition, SKBr3

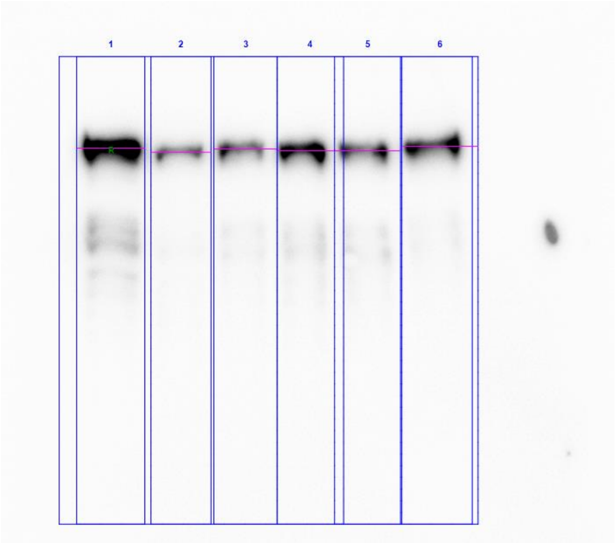

Figure S152. Image used for quantification: cRaf, 1st biological repetition, SKBr3

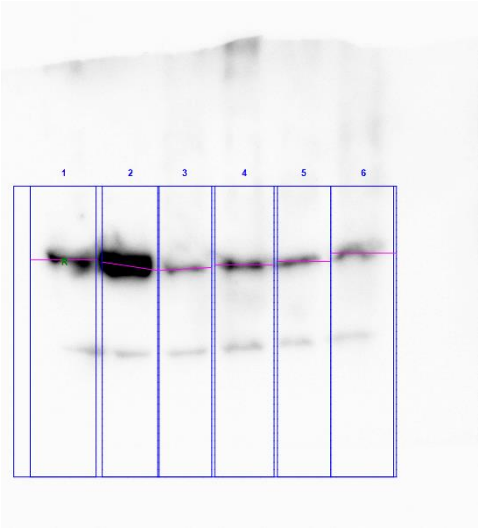

Figure S153. Image used for quantification: Hsp70, 1st biological repetition, SKBr3

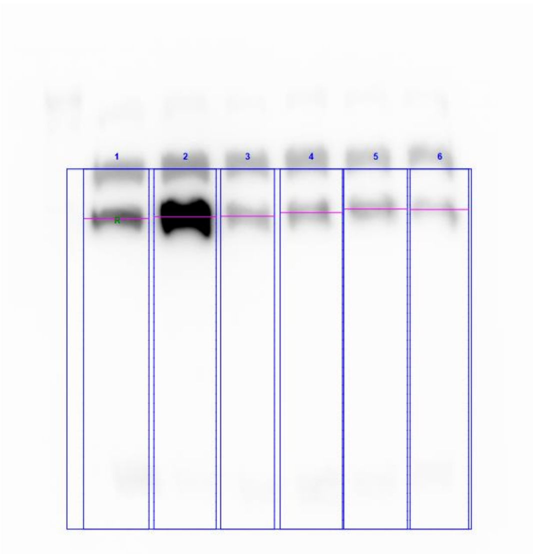

Figure S154. Image used for quantification: Hsp70, 2nd biological repetition, SKBr3

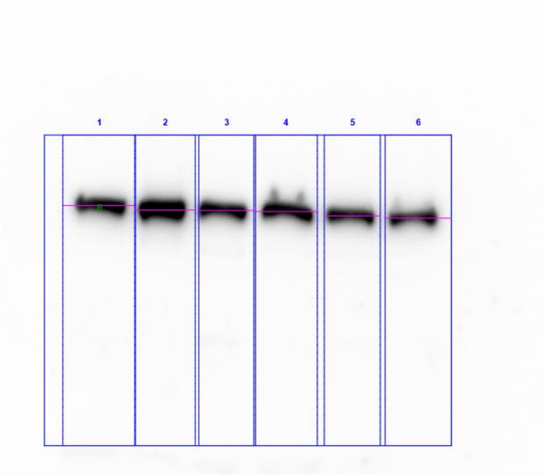

Figure S155. Image used for quantification: Hsp90, 1st biological repetition, SKBr3

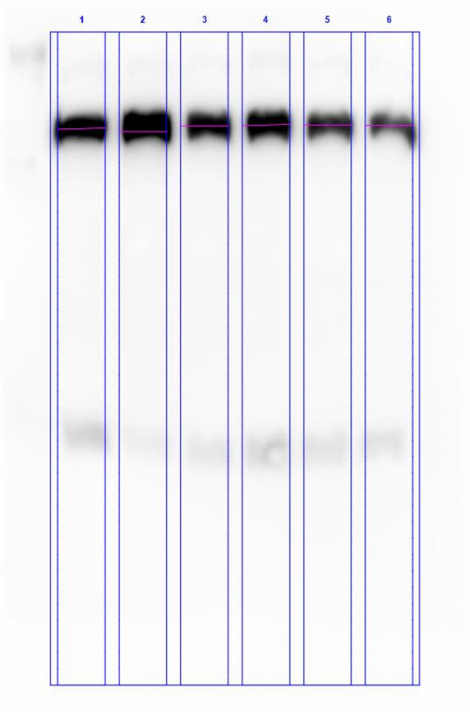

Figure S156. Image used for quantification: Hsp90, 2nd biological repetition, SKBr3

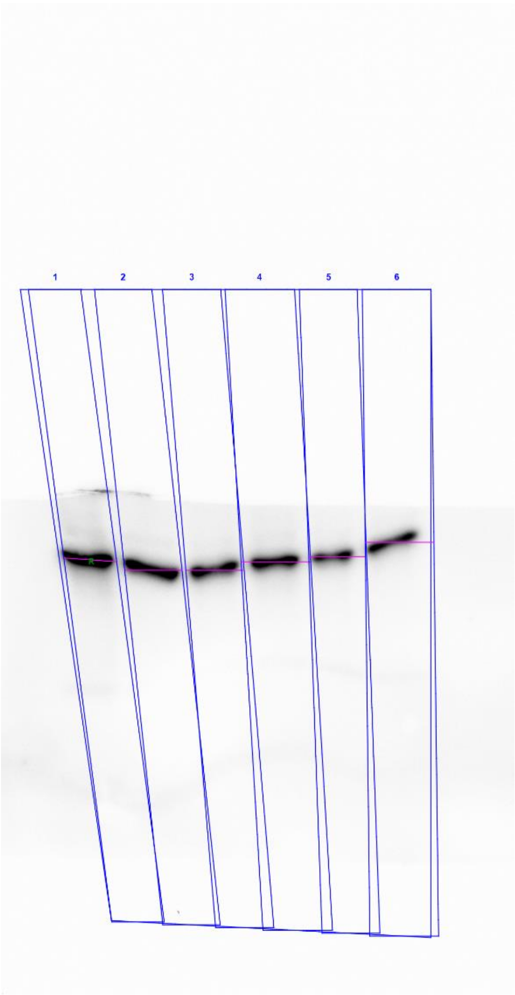

Figure S157. Image used for quantification: β-tubulin, SKBr3

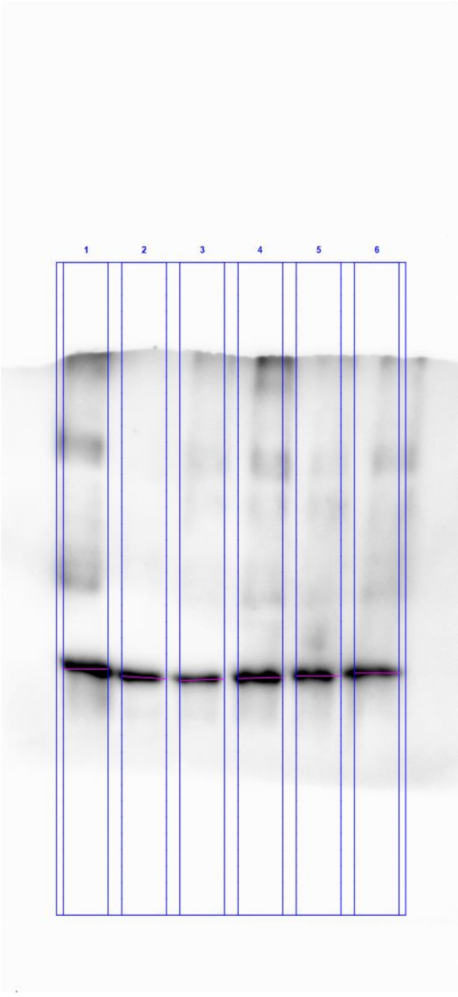

Figure S158. Image used for quantification: β-tubulin, SKBr3

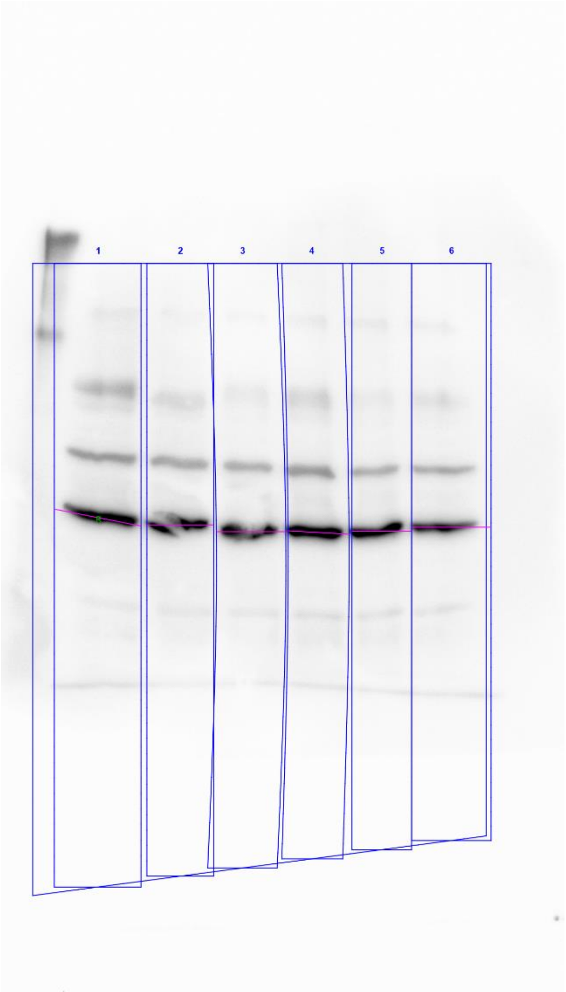

Figure S159. Image used for quantification: β-actin, SKBr3

1.11 Proliferation of untreated MDA-MB-231 cells

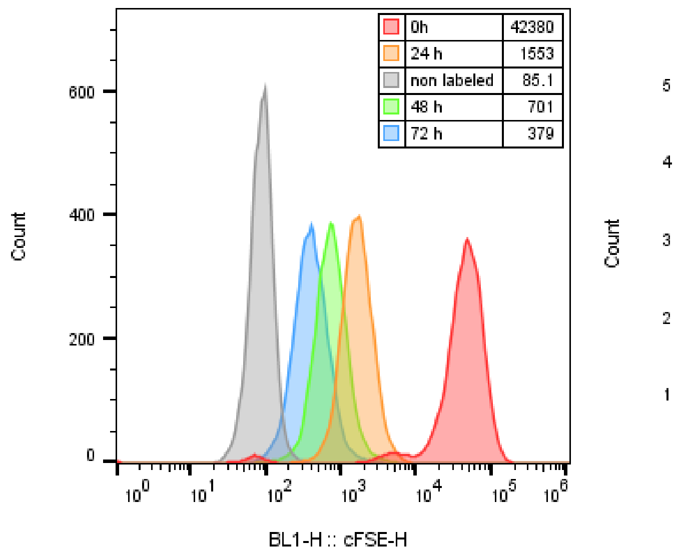

Figure S160. Graph of CFSE fluorescence of untreated MDA-MB-231 cells at different time points.

1.12 Determination of MTD in BALB/c nude mice

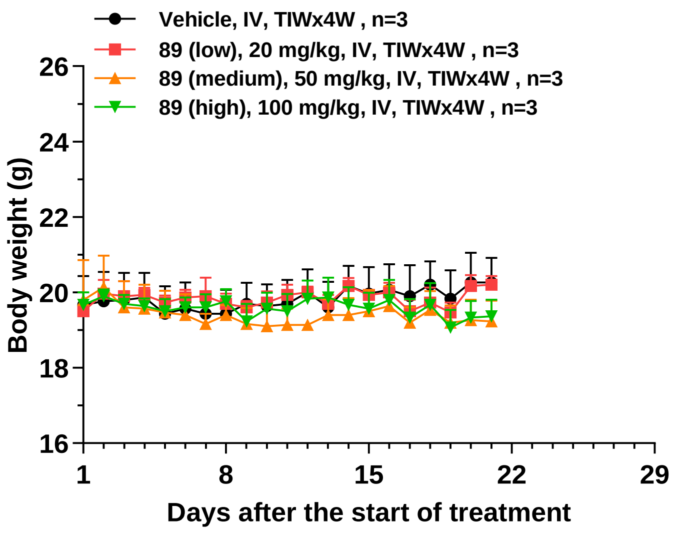

Figure S161. Graph of body weight changes in BALB/c nude mice when treating with vehicle or different dosages of compound **89**.

1.13 Kinase profiling

Kinase profiling system – General Panel was purchased from Promega (Promega, Madison, WI, USA) and was carried out according to manufacturer's protocol. General panel consists of 24 different kinases with substrate pairs. Each kinase had no-enzyme control wells, test wells with compound and no compound control wells. Firstly, 1 µL of compound solution was added to the wells. Then 2 µL of kinase solution and 2 µL of ATP/Substrate solutions were added to each well. Mixture was incubated ar room temperature for 60 minutes, then 5 µL of ADP-Glo™ Reagent (Promega, Madison, WI, USA) was added to all reactions in the plate. After 40 minutes incubation at room temperature 10 µL of Kinase Detection Reagent (Promega, Madison, WI, USA) was added to each well and incubated for another 30 minutes at room temperature. Luminescence was measured using Tecan's Spark Multimode Microplate reader (Tecan Trading AG, Switzerland). The kinase activity was calculated as a percentage of luminescence of test wells divided by luminescence of no compound control wells.

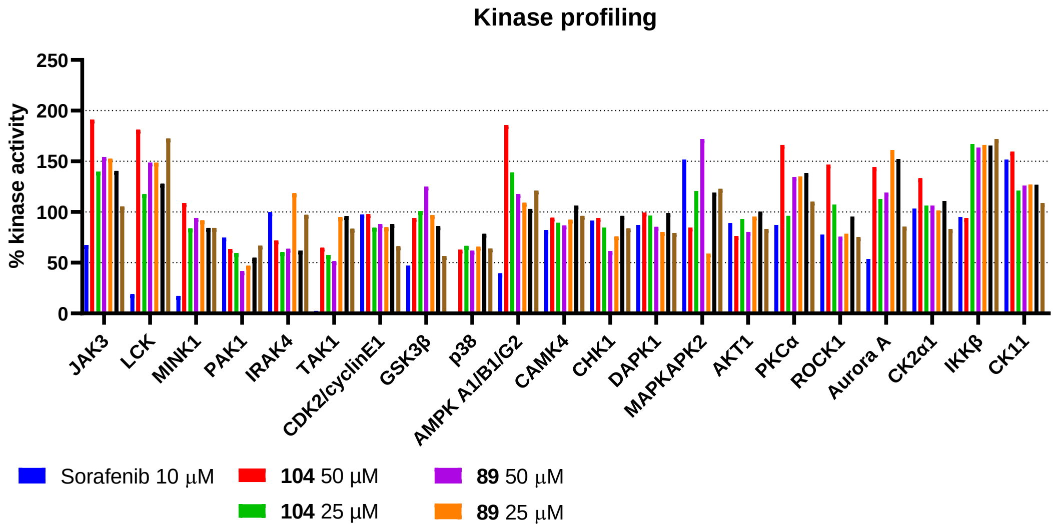

Figure S162. Kinase profiling system, consisting of 22 different kinases was carried out. Compounds **89** and **104** were tested at 50 and 25 µM. Sorafenib was used as a positive control.

1.14 Western blot analysis of MCF-7 cells when co-treated with 89 or 104 and carfilzomib

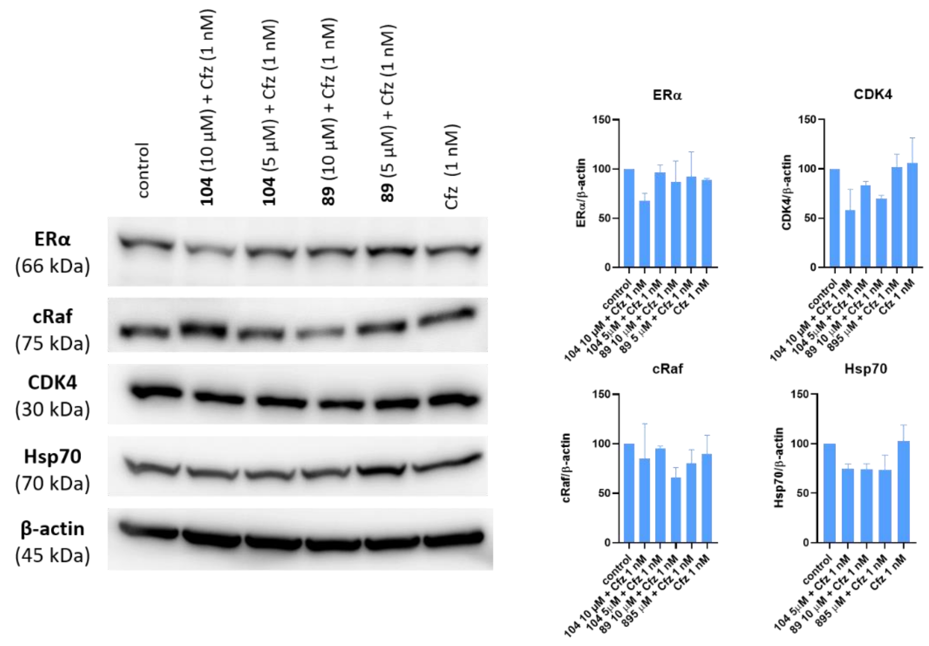

Figure S163. MCF-7 cell line was co- treated with 10 or 5 µM **89** or **104** and proteasome inhibitor 1 nM carfilzomib (Cfz) for 24 h. Exposure of MCF-7 cells to both Hsp90 CTD inhibitor in carfilzomib resulted in insignificant reduction of oncogenic protein levels compared to treatment with Hsp90 CTD inhibitor alone.

References

(1) Tomašič, T.; Durcik, M.; Keegan, B. M.; Skledar, D. G.; Zajec, Ž.; Blagg, B. S. J.; Bryant, S. D. Discovery of Novel Hsp90 C-Terminal Inhibitors Using 3D-Pharmacophores Derived from Molecular Dynamics Simulations. *Int. J. Mol. Sci.* **2020**, *21* (18), 6898. https://doi.org/10.3390/ijms21186898.

(2) Verba, K. A.; Wang, R. Y.-R.; Arakawa, A.; Liu, Y.; Shirouzu, M.; Yokoyama, S.; Agard, D. A. Atomic Structure of Hsp90-Cdc37-Cdk4 Reveals That Hsp90 Traps and Stabilizes an Unfolded Kinase. *Science* **2016**, *352* (6293), 1542–1547. https://doi.org/10.1126/science.aaf5023.

(3) Hawkins, P. C. D.; Skillman, A. G.; Warren, G. L.; Ellingson, B. A.; Stahl, M. T. Conformer Generation with OMEGA: Algorithm and Validation Using High Quality

Structures from the Protein Databank and Cambridge Structural Database. *J. Chem. Inf. Model.* **2010**, *50* (4), 572–584. https://doi.org/10.1021/ci100031x.

(4) McGann, M. FRED and HYBRID Docking Performance on Standardized Datasets. *J. Comput. Aided Mol. Des.* **2012**, *26* (8), 897–906. https://doi.org/10.1007/s10822-012-9584-8.

(5) Phillips, J. C.; Braun, R.; Wang, W.; Gumbart, J.; Tajkhorshid, E.; Villa, E.; Chipot, C.; Skeel, R. D.; Kalé, L.; Schulten, K. Scalable Molecular Dynamics with NAMD. *J. Comput. Chem.* **2005**, *26* (16), 1781–1802. https://doi.org/10.1002/jcc.20289.

(6) Huang, J.; Rauscher, S.; Nawrocki, G.; Ran, T.; Feig, M.; de Groot, B. L.; Grubmüller, H.; MacKerell, A. D. CHARMM36m: An Improved Force Field for Folded and Intrinsically Disordered Proteins. *Nat. Methods* **2017**, *14* (1), 71–73. https://doi.org/10.1038/nmeth.4067.

(7) Vanommeslaeghe, K.; Hatcher, E.; Acharya, C.; Kundu, S.; Zhong, S.; Shim, J.; Darian, E.; Guvench, O.; Lopes, P.; Vorobyov, I.; Mackerell Jr., A. D. CHARMM General Force Field: A Force Field for Drug-like Molecules Compatible with the CHARMM All-Atom Additive Biological Force Fields. *J. Comput. Chem.* **2010**, *31* (4), 671–690. https://doi.org/10.1002/jcc.21367.

(8) Vanommeslaeghe, K.; MacKerell, A. D. Automation of the CHARMM General Force Field (CGenFF) I: Bond Perception and Atom Typing. *J. Chem. Inf. Model.* **2012**, *52* (12), 3144–3154. https://doi.org/10.1021/ci300363c.

(9) Vanommeslaeghe, K.; Raman, E. P.; MacKerell, A. D. Jr. Automation of the CHARMM General Force Field (CGenFF) II: Assignment of Bonded Parameters and Partial Atomic Charges. *J. Chem. Inf. Model.* **2012**, *52* (12), 3155–3168. https://doi.org/10.1021/ci3003649.

(10) Humphrey, W.; Dalke, A.; Schulten, K. VMD: Visual Molecular Dynamics. *J. Mol. Graph.* **1996**, *14* (1), 33–38, 27–28. https://doi.org/10.1016/0263-7855(96)00018-5.

(11) Essmann, U.; Perera, L.; Berkowitz, M. L.; Darden, T.; Lee, H.; Pedersen, L. G. A Smooth Particle Mesh Ewald Method. *J. Chem. Phys.* **1995**, *103* (19), 8577–8593. https://doi.org/10.1063/1.470117.

(12) Ryckaert, J.-P.; Ciccotti, G.; Berendsen, H. J. C. Numerical Integration of the Cartesian Equations of Motion of a System with Constraints: Molecular Dynamics of n-

Alkanes. *J. Comput. Phys.* **1977**, *23* (3), 327–341. https://doi.org/10.1016/0021-9991(77)90098-5.

(13) Wolber, G.; Langer, T. LigandScout: 3-D Pharmacophores Derived from Protein-Bound Ligands and Their Use as Virtual Screening Filters. *J. Chem. Inf. Model.* **2005**, *45* (1), 160–169. https://doi.org/10.1021/ci049885e.
